# Efficacy and safety of the innovative monoclonal antibodies in adults with generalized myasthenia gravis: a Bayesian network analysis

**DOI:** 10.3389/fimmu.2023.1280226

**Published:** 2023-11-08

**Authors:** Huiru Chen, Youjia Qiu, Ziqian Yin, Zilan Wang, Yanbing Tang, Hanyu Ni, Jiaye Lu, Zhouqing Chen, Yan Kong, Zhong Wang

**Affiliations:** ^1^ Department of Neurosurgery & Brain and Nerve Research Laboratory, The First Affiliated Hospital of Soochow University, Suzhou, Jiangsu, China; ^2^ Department of Neurology, The First Affiliated Hospital of Soochow University, Suzhou, Jiangsu, China; ^3^ Suzhou Medical College of Soochow University, Suzhou, Jiangsu, China

**Keywords:** generalized myasthenia gravis, monoclonal antibody, FcRn inhibitor, complement inhibitor, B-cell targeting therapy, meta-analysis

## Abstract

**Background:**

A series of clinical trials support the effectiveness of monoclonal antibodies for generalized myasthenia gravis (MG) compared to the placebo, but the priority among drugs remains unclear. Therefore, we conduct a frequentist network meta-analysis (NMA) to compare the relative effects of different drugs for generalized MG.

**Methods:**

PubMed, Embase, Cochrane Library, and clinicaltrials.gov were systematically searched for eligible studies up to 1 June 2023. The primary outcome was efficacy (Myasthenia Gravis Activities of Daily Living [MG-ADL] score and Quantitative Myasthenia Gravis [QMG] score) and safety (adverse events [AEs]). Mean difference (MD) and risk ratio (RR) with their 95% credible intervals (95%CrIs) were used to show the effect size of continuous and categorical variables, respectively. The quality of evidence was assessed using the Grading of Recommendations Assessment, Development and Evaluation (GRADE) approach.

**Results:**

Thirteen studies involving 1167 individuals were identified for NMA. For efficacy outcomes, belimumab, efgartigimod, mezagitamab 600mg, and nipocalimab 60mg/kg were inferior to rozanolixzumab 7mg/kg (MD ranged from 2 to 3.69) and rozanolixzumab 10mg/kg (MD ranged from 2.04 to 3.72) in MG-ADL score, and rozanolixzumab had the highest rank probability (83%) according to the subjective surface under the curve ranking area (SUCRA). For QMG score, batoclimab 340mg (MD ranged from 4.32 to 8.52) and batoclimab 680mg (MD ranged from 4.11 to 9.31) were more effective than placebo and other monoclonal antibodies except for rozanolixzumab, with the highest SUCRA value (93% and 97% respectively). For safety outcomes, belimumab achieved the highest SUCRA value (89.8%) with significant statistical difference compared to rozanolixzumab 7mg/kg (RR 0.08, 95%CrI 0.01 to 0.94) and rozanolixzumab 10mg/kg (RR 0.08, 95%CrI 0.01 to 0.86).

**Conclusion:**

While all monoclonal antibodies were superior to the placebo, rozanolixzumab and batoclimab might be the most effective for generalized MG. However, rozanolixzumab was associated with higher incidence of AEs. Given the limitations inherent in indirect comparisons, further head-to-head and extensive observational studies are necessary to confirm our findings.

**Systematic review registration:**

https://inplasy.com/?s=202370112, identifier 202370112.

## 1 Introduction

Myasthenia gravis (MG) is a chronic autoimmune disorder primarily targeting the neuromuscular junction, leading to fluctuating skeletal muscle weakness and fatigue (1). Clinical manifestations of MG range from mild symptoms such as ptosis and diplopia to more severe complications like difficulty swallowing, dysarthria, and weakness of the respiratory, axial, and limb muscles (2). The primary pathogenesis of MG is pathogenic immunoglobin G (IgG) antibodies inhibiting neuromuscular transmission by binding to various proteins, most notably the receptors in the postsynaptic membrane (3). Pathogenic antibodies targeting the nicotinic acetylcholine receptor (AChR) can be detected in up to 80% of patients, while those against muscle-specific kinase (MuSK) are found in about 6%. More rarely, anti-lipoprotein receptor-related protein 4 can also be detected without detectable antibodies and is called seronegative (10-15%) (1, 4, 5). Although MG was a relatively rare disease with incidence ranging from 0.15 to 61.33 per million person-years, there was a rapid increase in its pooled incidence rate of MG since 1976 (6, 7). Notably, approximately 75% of patients with ocular MG might progress to generalized MG within the initial 2-3 years following diagnosis, and 15%-20% of patients were likely to experience at least one myasthenic crisis (8–10).

There are several conventional drug treatments for MG, including acetylcholinesterase medications, corticosteroids, immunosuppressive agents (such as azathioprine, ciclosporin, methotrexate, mycophenolate, or tacrolimus), intravenous immunoglobulin, and plasmapheresis (11, 12). Acetylcholinesterase medications were the first-line therapeutic drugs for MG, followed by corticosteroids and immunosuppressive agents. Plasmapheresis and intravenous immunoglobulin were used as short-term treatment for patients with life-threatening signs or myasthenic crisis (11). MG treatment aimed to maintain minimal manifestations of disease or continue a low dose of oral corticosteroids long-term (11, 12). Although many patients with MG may experience temporary or permanent relief from muscle weakness, approximately 10-15% of patients responded inadequately to current treatment or cannot tolerate the complication of immunosuppressive agents (13, 14). Therefore, innovative treatment options for MG were significantly needed to reduce the disease’s severity and burden.

Nowadays, several clinical studies have demonstrated the improvement of clinical symptoms and better tolerance of new target-specific immunological agents in generalized MG. These innovative immunological agents could be mainly classified into three categories according to their mechanisms: neonatal Fc receptor inhibitors (FcRn) (such as rozanolixizumab, efgartigimod, batoclimab, and nipocalimab), complement inhibitors (such as eculizumab, ravulizumab, and zilucoplan), and B-Cell therapies (such as rituximab, belimumab, and iscalimab) (15, 16). Eculizumab, ravulizumab, efgartigimod, and rozanolixizumab have gained Food and Drug Administration (FDA) approval in treating generalized MG (17–20). However, the superiority and inferiority among monoclonal antibodies still need to be clarified due to limited evidence on drug comparison.

A Bayesian network meta-analysis (NMA) was performed to compare the effectiveness of multiple interventions quantitatively, pool the evidence results of direct and indirect comparison, and rank the interventions targeting different outcomes (21). Therefore, in the absence of data from head-to-head randomized trials, we pooled data from previous randomized controlled trials (RCTs) and conducted an NMA to investigate the priority of new therapeutic agents for generalized MG.

## 2 Methods

### 2.1 Methods and materials

This NMA complies with the Preferred Reporting Items for Systematic Reviews and Meta-Analysis Statement (PRISMA) checklist (22). The protocol of this systematic review has been registered on INPLASY (registration ID: 202370112), an international platform for registering systematic reviews and meta-analysis protocols (23). No ethical review is required since all analysis was based on previously published research.

### 2.2 Literature search

A comprehensive search was performed by two independent reviewers (HRC and YJQ) in PubMed, Embase, Cochrane Library, and clinicaltrials.gov from their inception to 1 June 2023. Medical MeSH terms and general terms were combined to identify relevant studies. In addition, we also reviewed previous meta-analyses and a bibliography of published reviews and included studies for supplementary search. A detailed description of search strategies and the extraction of each database can be found in the supplementary materials (**Supplementary Table S1**).

### 2.3 Eligibility criteria

Studies meeting the PICOS criteria were enrolled: (1) participants: adult patients diagnosed as generalized MG and met a Myasthenia Gravis Foundation of America clinical classification of class II to V at screening; (2) intervention: patients received monoclonal antibodies by intravenous or subcutaneous injection. Different dosages of the same monoclonal antibody were treated as distinct intervention groups in the NMA; (3) comparison: patients received placebo; (4) outcomes: the efficacy outcomes were Myasthenia Gravis Activities of Daily Living (MG-ADL) score, Quantitative Myasthenia Gravis (QMG) score, Myasthenia Gravis Composite (MGC) score, 15-item revised version of the Myasthenia Gravis Quality of Life (MG-QoL 15r) score. Safety outcomes were adverse events (AEs), serious adverse events (SAEs), and all-cause mortality. Moreover, the more frequently reported AEs among the included studies were headache, diarrhea, and nausea, which were included in the quantitative analysis of the NMA;(5) study type: only studies with randomized controlled design were enrolled.

Studies matching one of the following items were excluded: (1) reviews, observational studies, case reports, letters, and conference abstracts; (2) studies not written in English; (3) studies not providing original data. In order to avoid overlapping populations, we exclusively included phase III clinical trials for the same monoclonal antibody in the NMA. Phase II clinical trials were excluded as they were deemed to exceed inclusion criteria in the flow diagram, and details of these specific trials were available in **Supplementary Table S2**.

### 2.4 Study selection and data extraction

Two reviewers (HRC and YJQ) independently screened titles and abstracts according to the inclusion criteria. Then, the full text was reviewed for eligibility. Divergences were settled by discussing with a senior professional reviewer (ZQY) who did not participate in the extraction period. The following data were extracted: (1) last name of the first author and publication year; (2) demographic data such as sample size, mean age, and gender ratio (female); (3) comparison of interventions; (4) detailed dosage regimens (including dose, frequency, way of administration); (5) outcome measures and follow-up durations. In addition, we transformed data presented as medians and interquartile ranges to mean and standard deviations using the method described by Hozo et al. (24).

### 2.5 Quality assessment and risk of bias

We accessed the certainty of the evidence for each paired comparison using the methodologies outlined by the Grading of Recommendations, Assessment, Development, and Evaluation (GRADE) Working Group. The evidence of confidence rating in direct comparisons and indirect comparisons for different outcomes was evaluated using the Confidence in Network Meta-Analysis (CINeMA) framework (25). The certainty of evidence for each comparison of different outcomes was rated high, moderate, low, and very low, based on the overall risk of bias, publication bias, inconsistency, indirectness, and imprecisions (26). The risk of bias for enrolled studies was estimated by two independent reviewers using the Cochrane Collaboration tool (27), with discrepancies resolved through discussion with the third reviewer. Bias was evaluated either as low, high, or unclear using Review Manager 5.4.

### 2.6 Statistical analysis

Before performing NMA, we conducted a pairwise meta-analysis of direct evidence using Review Manager 5.4. For continuous variables, mean difference (MD) with 95% confidence intervals (95% CIs) was a statistical measure to quantify the effect size. At the same time, risk ratio (RR) with 95%CIs was used to show the effect size of categorical variables. *I^2^* statistics was used to estimate statistical heterogeneity between trials in NMA. If the heterogeneity was low (*I^2^* < 50%), a fixed-effect model was adopted for analysis; otherwise, a random-effect model was used.

NMA was conducted using a Bayesian framework using the ‘gemtc’ package of the R software environment version 4.2.2 (28). Similarly, we estimated the MD with 95% CrIs for continuous variables and the RR for dichotomous variables with 95% CrIs. The track and density plot and the Brooks-Gelman-Rubin diagnosis plot evaluated the convergence of the model. The fixed or random model effect selection was based on the outcome of *I^2^* statistics. The network comparisons of different interventions were shown in network maps where each node represented an intervention, and the breadth of the connecting lines indicated the number of trials between the two interventions.

The global inconsistency by comparing the deviance information criterion (DIC) of the random-effect or fixed-effect model and the DIC less than 11 indicated better global consistency (29). Since all comparisons were monoclonal antibodies versus placebo or different drug dosages, and there was a lack of direct comparison of different monoclonal antibodies and no closed loops in the network plots, the node-splitting method examined the consistency was not performed (30). Besides, we performed heterogeneity analysis for each outcome in our NMA, and sensitivity analyses were further performed by excluding possible low evidence or possible high heterogeneity trials to evaluate the robustness of our findings. The surface under the cumulative ranking curve (SUCRA) ranged from 0 to 1 and was applied to calculate the ranking probabilities of each intervention, with a higher score indicating a better rank. In addition, the hierarchical cluster ranking based on SUCRA values of efficacy (MG-ADL score) and safety outcomes (severe AEs) was constructed to visually compare different interventions on a graph where the value in the upper and right quadrant indicated the more effective and safe interventions (31). All comparisons were performed using a two-sided t-test, and a cut-off point of 0.05 was considered statistically significant.

The publication bias was checked by generating funnel plots using STATA 17.0 (32), and an asymmetric distribution of the funnel plot indicates a significant publication bias.

## 3 Results

### 3.1 Study characteristics

**Figure 1:**
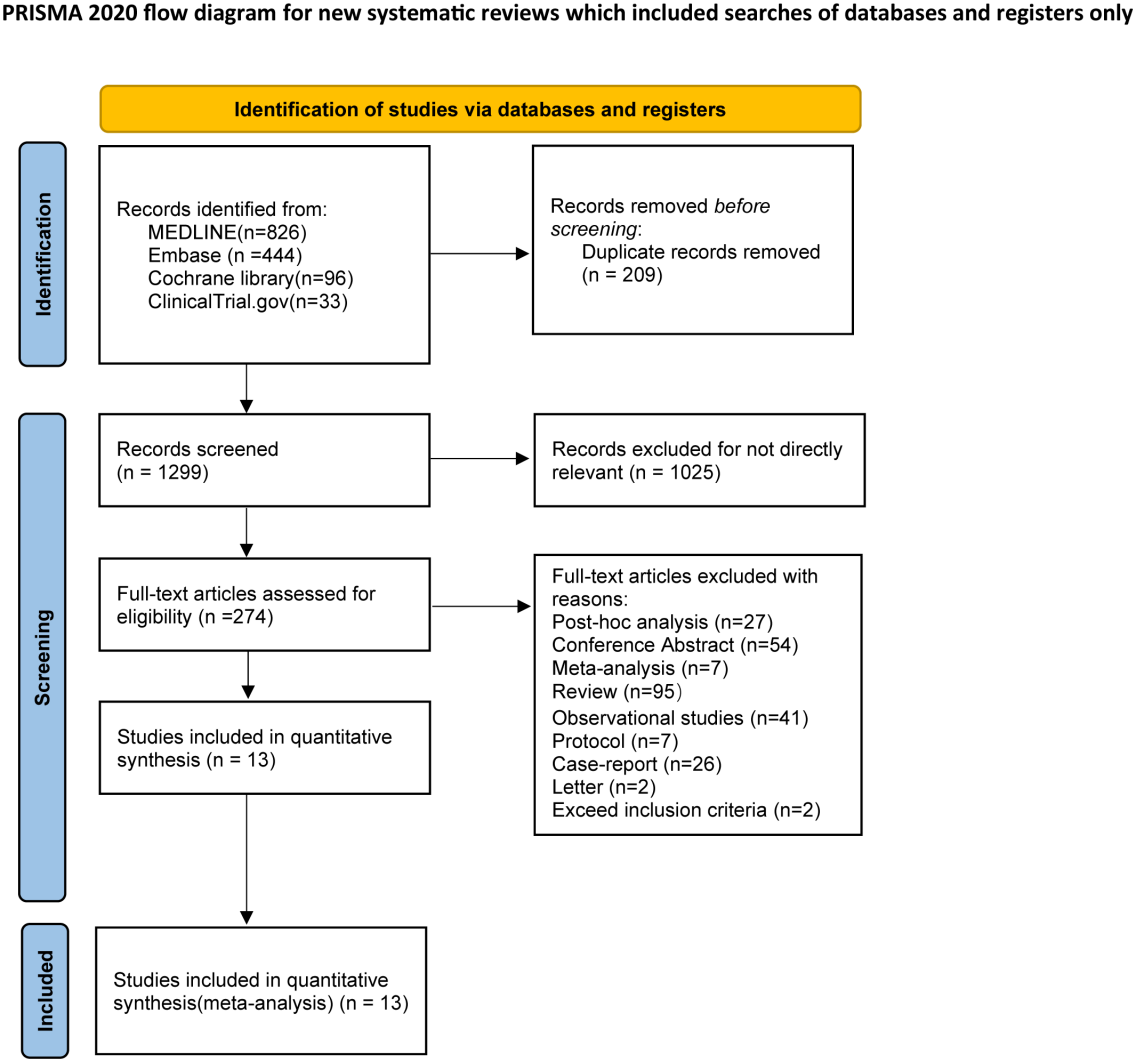
Flow diagram of study selection.

A total of 1399 studies from three databases and one clinical trial were identified for eligibility. The two reviewers excluded duplications and irrelevant studies by screening titles and abstracts. Then, 274 studies were reviewed in full text, and 41 were excluded for non-RCT studies. Finally, 13 studies were enrolled for NMA analysis. Three trials contained three intervention groups, while one contained four intervention groups, and the remaining studies had two intervention groups. The flow chart of the search program is shown in **Figure 1**.

Overall, 1167 patients diagnosed with generalized MG were enrolled. The mean age was 51.59 ± 16.28, and the proportion of females was 59.21%. The duration of the studies ranged from 8 to 52 weeks. The following monoclonal antibodies were identified, including rozanolixzumab, batoclimab, zilucoplan, nipocalimab, eculizumab, ravulizumab, iscalimab, rituximab, efgartigimod, and belimumab. Of note, two dosages of razanolixzumab (7 mg/kg and 10 mg/kg) and batoclimab (340mg and 680mg), and three dosages of nipocalimab (5 mg/kg, 30 mg/kg, and 60 mg/kg) were identified as different regimens. The included studies’ characteristics are shown in **Table 1**, and the details of studies’ inclusion and exclusion are shown in **Supplementary Table S3**.

**Table 1:** characteristic of included randomized controlled trails for patients.

Study	Countries	Center	Publications	Treatment group (No. of participants)	Female (%)	Mean age ± SD(years)	Duration of disease(years)	AChR antibody positive (%)	Study period	Outcome Events
FcRn inhibitors										
Bril et al. (33) NCT03971422	Asia, Europe, North America	81	The Lancet Neurology	ROZ 7mg/kg (66) vs. ROZ 10mg/kg (67) vs. PLA (67)	ROZ 7mg: 59 ROZ 10mg: 52 PLA: 70	ROZ 7mg: 53.2 ± 14.7 ROZ 10mg: 51.9 ± 16.5 PLA: 50.4 ± 17.7	ROZ 7mg: 5.2 ± 4.7 ROZ 10mg: 7.7 ± 8.8 PLA: 7.6 ± 8.2	ROZ 7mg: 90.9 ROZ 10mg:89.5 PLA: 88.1	43 days	a.b.c.d.e.f.g
Yan et al. (34) NCT04346888	China	1	Neurology and Therapy	BAT 340mg (10) vs. BAT 680mg (11) vs. PLA (9)	BAT 340mg: 81.8 BAT 680mg: 80.0 PLA: 77.8	BAT 340mg: 36.4 ± 9.8 BAT680mg: 40.6 ± 16.8 PLA: 40.2 ± 9.3	BAT340mg: 9.8 ± 10.8 BAT680mg: 6.4 ± 5.7 PLA: 6.0 ± 6.8	BAT340mg: 90.0 BAT680mg: 100 PLA: 88.9	43 days	a.b.c.d.e.f.g
Howard et al. (35) NCT 02965573	United States, Belgium, Canada, Italy, Netherlands, Poland, Spain, Sweden	18	American Academy of Neurology	EFG (12) vs. PLA (12)	EFG 10mg: 58.3 PLA: 66.7	EFG: 55.3 ± 13.6 PLA: 43.5 ± 19.3	EFG: 8.2 ± 9.0 PLA: 13.3 ± 11.2	EFG: 100 PLA: 100	78 days	a.b.c.d.e.f.g
Howard et al. (36) NCT03669588	Japan, Europe, North America	56	Journal of Neurology	EFG (84) vs. PLA (83)	EFG: 75 PLA: 66	EFG: 45.9 ± 14.4 PLA: 48.2 ± 15.0	EFG: 10.1 ± 9.0 PLA: 8.8 ± 7.6	EFG: 77.1 PLA: 77.4	8 weeks	a.b.c.d.e.f.g
NCT03772587	United States, Belgium, Canada, Germany, Italy, Poland, Spain, United Kingdom	61	NA	NIP 5mg (14) vs. NIP 30mg (13) vs. NIP 60mg (13) vs. PLA (14)	NIP 5mg: 19.54 NIP 30mg: 15.4 NIP 60mg: 15.03 PLA: 17.64	NIP 5mg: 49 ± 19.54 NIP 30mg: 53.1 ± 15.4 NIP 60mg: 59.9 ± 15.03 PLA: 54.8 ± 17.64	NA	NA	57 days	a.b.d.e.f.g
Complement inhibitors										
Howard et al. (37) NCT01997229	North America, Latin America, Europe, Asia	76	The Lancet Neurology	ECU (62) vs. PLA (63)	ECU: 66 PLA: 65	ECU: 47.5 ± 15.66 PLA: 46.9 ± 17.98	ECU: 9.9 ± 8.1 PLA: 9.2 ± 8.4	NA	26 weeks	a.b.c.d.f.g
Howard et al. (38) NCT04115293	Europe, Japan, North America	75	The Lancet Neurology	ZIL (86) vs. PLA (88)	ZIL: 60 PLA: 53	ZIL: 52.6 ± 14.6 PLA: 53.3 ± 15.7	ZIL: 9.3 ± 9.5 PLA: 9.0 ± 10.4	ZIL: 100 PLA: 100	12 weeks	a.b.c.d.e.f.g
NCT03920293	United States, Austria, Canda, Italy, France, Germany, Israel, Japan, Korea	Multicenter	NA	RAV (86) vs. PLA (89)	RAV: 48.8 PLA: 50.6	RAV: 58.0 ± 13.82 PLA: 53.3 ± 16.05	NA	NA	26 weeks	a.b.d.e.f.g
B-Cells inhibitors										
Hewett et al. (39) NCT01480596	Canada, United States, Germany, Italy	13	Neurology	BEL (18) vs. PLA (21)	BEL: 67 PLA: 56	BEL: 52.7 ± 17.32 PLA: 59.0 ± 13.88	BEL: 6.95 ± 9.03 PLA: 8.3 ± 8.06	BEL: 100 PLA: 90.4	24 weeks	a.b.c.e.f.g
Nowak et al. (40) NCT02110706	United States	26	Neurology	RIT (25) vs. PLA (27)	RIT: 44 PLA: 44.4	RIT: 53.2 ± 17.5 PLA: 56.8 ± 7	NA	RIT: 68 PLA: 85.2	52 weeks	a.b.c.d.e.f.g
Piehl et al. (41) NCT 02950155	Swedish	7	JAMA Neurology	RIT (25) vs. PLA (22)	RIT: 28 PLA: 31.8	RIT: 67.4 ± 13.4 PLA: 58 ± 18.6	RIT: 132.4 ± 91.5(day) PLA:143.0 ± 93.3(day)	RIT: 92 PLA:100	16 weeks	a.b.e.f.g
NCT02565576	Canada, Denmark, Germany, Russian Federation, Taiwan	15	NA	ISC (22) vs. PLA (22)	ISC: 54.5 PLA: 72.7	ISC: 44.7 ± 13.54 PLA: 43.3 ± 13.92	NA	NA	25 weeks	a.b.c.d.f.g
NCT04159805	United States, Canada, Italy, Poland, Serbia, Spain	50	NA	MEZ 300mg (12) vs. MEZ 600mg (12) vs. PLA (12)	TAK 300mg: 50 TAK 600mg: 58.3 PLA: 75	TAK 300mg: 45.3 ± 14.42 TAK 600mg: 56.3 ± 12.47 PLA46.5 ± 18.03	NA	NA	16 weeks	a.b.c.d.e.fg

a. Myasthenia Gravis Activities of Daily Living (MG-ADL) score.

b. Quantitative Myasthenia Gravis (QMG) score.

c. Myasthenia Gravis Composite (MGC) score.

d. 15-item revised version of the Myasthenia Gravis Quality of Life (MG-QoL 15r) score.

e. adverse effects (AEs).

f. several adverse effects (SAEs).

g. all-cause mortality.

ROZ, rozanolixzumab; BAT, batoclimab; EFG, efgartigimod; NIP, nipocalimab; ECU, eculizumab; ZIL, zilucoplan; RAV, ravulizumab; BEL, belimumab; RIT, rituximab; ISC, iscalimab; MEZ, mezagitamab; PLA, placebo; NA, not applicable; AChR, nicotinic acetylcholine receptor.

### 3.2 Network meta-analysis

**Figure 2:**
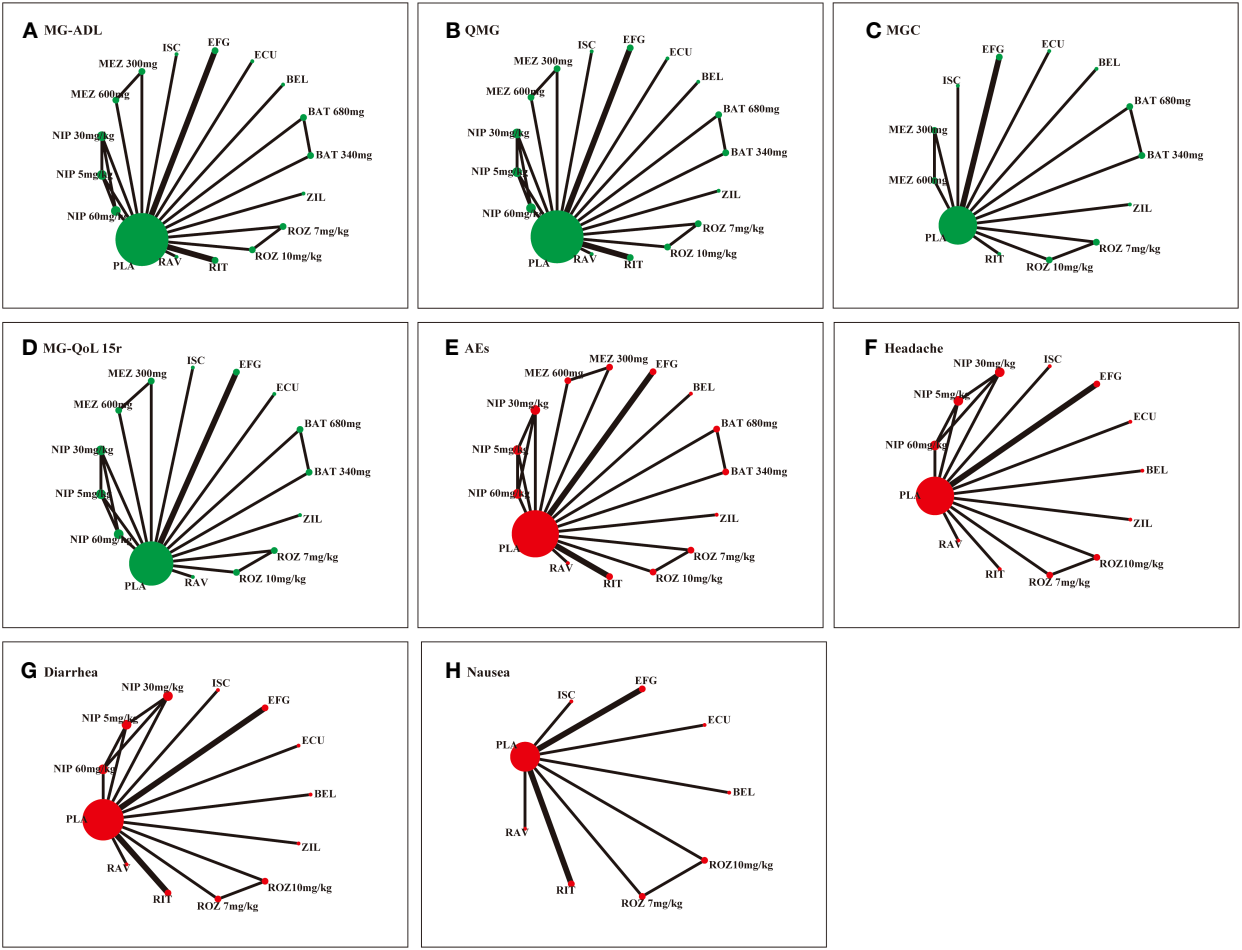
Network of RCTs comparing different monoclonal antibodies in the patients with generalized MG. Green color and red color represent the efficacy and safety, respectively. **(A)** Myasthenia Gravis Activities of Daily Living (MG-ADL) score. **(B)** Quantitative Myasthenia Gravis (QMG) score. **(C)** Myasthenia Gravis Composite (MGC) score. **(D)** 15-item revised version of the Myasthenia Gravis Quality of Life (MG-QoL 15r) score. **(E)** adverse effects (AEs). **(F)** Headache. **(G)** Diarrhea. **(D)** Nausea. ROZ, rozanolixzumab; BAT, batoclimab; EFG, efgartigimod; NIP, nipocalimab; ECU, eculizumab; ZIL, zilucoplan; RAV, ravulizumab; BEL, belimumab; RIT, rituximab; ISC, iscalimab; MEZ, mezagitamab; PLA, placebo;.

**Figure 2** illustrates the network map of different monoclonal antibodies regarding efficacy and safety outcomes. Each node represents an intervention, the size of the nodes indicates the number of participants, and the thickness of the edges indicates the number of trials between the two strategies.

#### 3.2.1 MG-ADL score network

**Figure 3:**
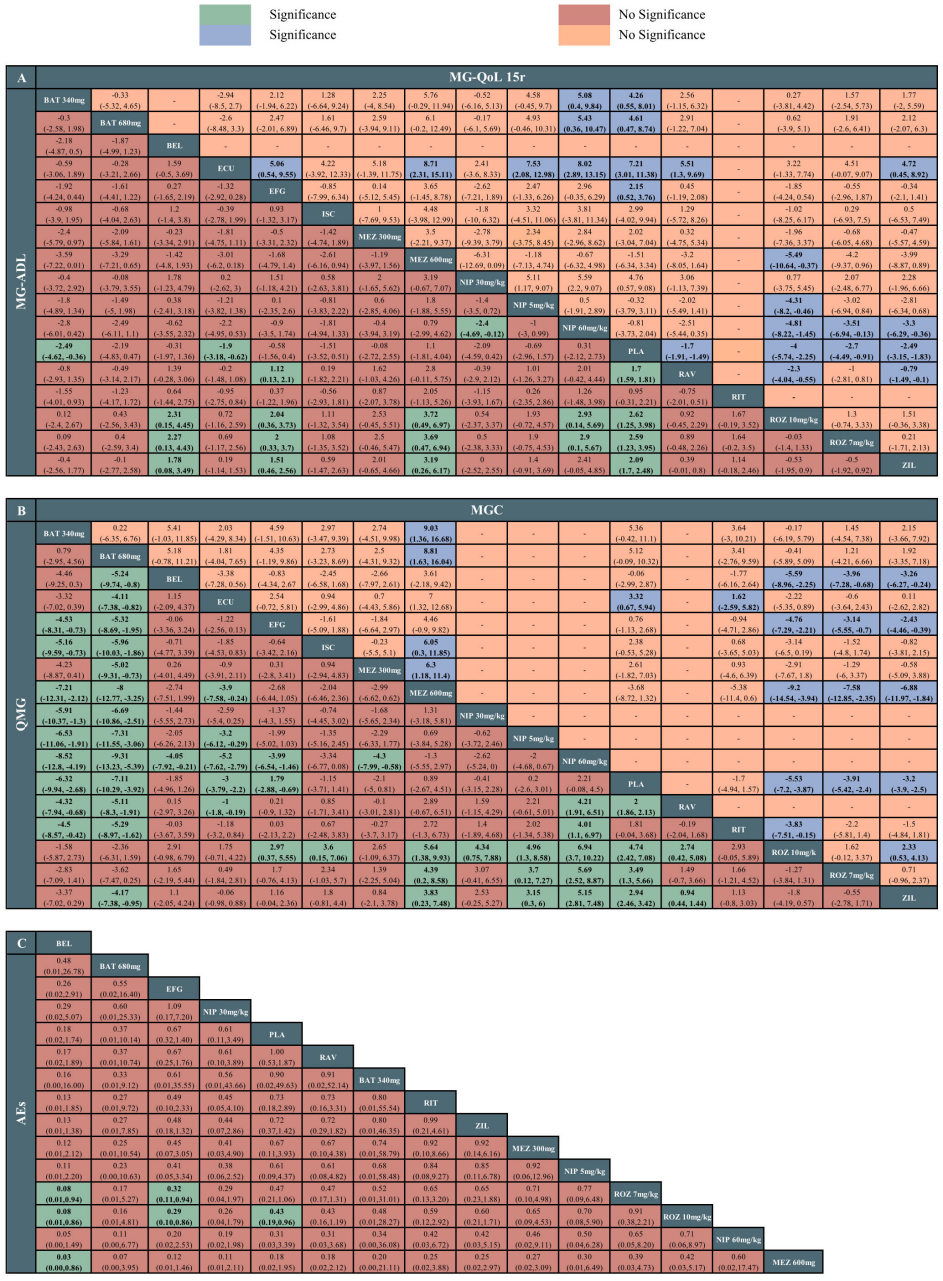
League tables of the outcomes of the efficacy and safety. **(A)** Myasthenia Gravis Activities of Daily Living (MG-ADL) score and 15-item revised version of the Myasthenia Gravis Quality of Life (MG-QoL 15r) score. **(B)** Quantitative Myasthenia Gravis (QMG) score and Myasthenia Gravis Composite (MGC) score. **(C)** adverse effects (AEs). ROZ, rozanolixzumab; BAT, batoclimab; EFG, efgartigimod; NIP, nipocalimab; ECU, eculizumab; ZIL, zilucoplan; RAV, ravulizumab; BEL, belimumab; RIT, rituximab; ISC, iscalimab; MEZ, mezagitamab; PLA, placebo;.

The network meta-analysis of MG-ADL included 13 studies with 17 interventions. Batoclimab 340mg (MD -3.46, 95% CrI -4.62 to -0.36), eculizumab (MD -1.9, 95% CrI -3.18 to -0.62), ravulizumab (MD -1.7, 95% CrI -1.91 to -1.49), rozanolixzumab 10mg/kg (MD -4.00, 95% CrI -5.74 to -2.55), rozanolixzumab 7mg/kg (MD -2.7, 95% CrI -4.49 to -0.91), zilucoplan (MD -2.49, 95% CrI -3.15 to -1.83) showed superiority to placebo. Among monoclonal antibodies, rozanolixzumab 10mg/kg and rozanolixzumab 7mg/kg were superior to belimumab, efgartigimod, mezagitamab 600mg, and nipocalimab 60mg/kg (MDs ranging between 2.00 and 3.72, very low to low certainty). Zilucoplan demonstrated superiority to belimumab, efgartigimod, mezagitamab 600mg (MDs ranging between 1.51 and 3.19, very low certainty). Additionally, there was a statistical difference between ravulizumab and efgartigimod (MD 1.12, 95% CrI 0.13 to 2.1, low certainty), as well as nipocalimab 60mg/kg and nipocalimab 30mg/kg (MD -2.4, 95% CrI -4.69 to -0.12, low certainty). According to SUCRA, rozanolixzumab 10mg/kg, rozanolixzumab 7mg/kg ranked first (83%), followed by batoclimab (78%) and zilucoplan (74%). In comparison, mezagitamab 600mg (11%), nipocalimab 60mg/kg (18%), and placebo (18%) were the worst therapies. The detailed results are showed in **Figure 3A**. Furthermore, our assessment of the certainty of evidence was presented in the CINeMA diagram in **Supplementary Table S4**.

#### 3.2.2 QMG score network

The network meta-analysis of QMG included 13 studies with 17 treatments. Batoclimab 340mg (MD -8.52, 95% CrI -12.8 to -4.19), batoclimab 680mg (MD -9.31, 95% CrI -13.23 to -5.39), eculizumab (MD -5.2, 95% CrI -7.62 to -2.79), efgartigimod (MD -3.99, 95% CrI -6.54 to -1.46), ravulizumab (MD 2, 95% CrI 1.86 to 2.13), rozanolixzumab 10mg/kg (MD 4.74, 95% CrI 2.42 to 7.08), rozanolixzumab 7mg/kg (MD 3.49, 95% CrI 1.30 to 5.66), zilucoplan (MD 2.94, 95% CrI 2.46 to 3.42) were statistically superior to the placebo. Among drugs, batoclimab 680mg was statistically superior to other treatments except for two dosages of rozanolixzumab (MDs ranging between -9.31 and -4.11, very low to low certainty), with the highest SUCRA value (97%); this was followed by batoclimab 340mg (93%) and rozanolixzumab 10mg/kg (86%). Batoclimab 340mg and rozanolixzumab 10mg/kg were statistically superior to efgartigimod, iscalimab, mezagitamab 600mg, ravulizumab, and three dosages of nipocalimab (MDs ranging between -8.52 and 6.94, very low to low certainty). In contrast, nipocalimab 60mg/kg appeared to be the worst treatment according to the SUCRA value (3%) (**Figure 3B**).

#### 3.2.3 MGC score network

The network meta-analysis of MGC included 10 studies with 13 therapies. Rozanolixzumab 10mg/kg, rozanolixzumab 7mg/kg, and zilucoplan were statistically superior to efgartigimod (MDs ranging between -4.76 and -2.43, very low to low certainty), mezagitamab 600mg (MDs ranging between -9.2 and -6.88, very low to low certainty), and placebo (MDs ranging between -5.53 and -3.2, low to high certainty). Among these three drugs, rozanolixzumab 10mg/kg demonstrated superiority to zilucoplan (MD 2.3, 95% CrI -0.53 to 4.13), while there was no significant difference between the two doses of the rozanolixzumab. In addition, mezagitamab 600mg was statistically inferior to other therapies except for belimumab, efgartigimod, and rituximab. According to SUCRA, rozanolixzumab 10mg/kg might be the most effective therapy (90%), while mezagitamab 600mg was the worst (3%) (**Figure 3B**).

#### 3.2.4 MG-QoL 15r score network

The network meta-analysis of MG-QoL 15r included 10 studies with 15 treatments. All treatments demonstrated superiority to the placebo group except for iscalimab, mezagitamab 300mg, and mezagitamab 600mg (MDs ranging between -4.0 and -7.21, very low to low certainty). For drugs comparisons, eculizumab had the highest ranking probability (93%). It demonstrated a significant difference when compared to efgartigimod, mezagitamab 600mg, nipocalimab 5mg/kg, nipocalimab 60mg/kg, ravulizumab, and zilucoplan (MDs ranging between 4.72 and 8.71, very low to moderate certainty). In addition, different dosages of batoclimab, rozanolixzumab, and zilucoplan showed statistical differences with nipocalimab 60mg/kg. Among various dosages of nipocalimab, nipocalimab 30mg/kg was superior to other dosages. Meanwhile, mezagitamab 600mg (12%), and nipocalimab 60mg/kg (12%) appeared to be the worst treatments (**Figure 3A**).

#### 3.2.5 AEs network

The network meta-analysis of AEs included 11 studies with 15 therapies. Two dosages of rozanolixzumab were associated with a higher risk of AEs compared with belimumab, efgartigimod, and placebo. According to SUCRA, belimumab ranked first (89.8%), while mezagitamab 300mg ranked last (15.4%). Among AEs, headache, diarrhea, and nausea were the most reported. For headache, rozanolixzumab 7mg/kg demonstrated more incidence of headache than eculizumab, efgartigimod, ravulizumab, zilucoplan, and placebo, while rozanolixzumab 10mg/kg had more incidence of headache than ravulizumab and placebo. For diarrhea, a statistical difference was observed between rituximab and belimumab. In addition, zilucoplan demonstrated more incidences of diarrhea than belimumab, efgartigimod, and placebo. For nausea, no statistical difference was observed among monoclonal antibodies. According to SUCRA, belimumab ranked first in headache (89%) and diarrhea (89%), while efgartigimod ranked first in nausea (71%). **Figure 3C** and **Supplementary Figure S19** illustrate the detailed results of network estimates.

The proportion of patients who experienced SAEs related to the monoclonal antibodies was similar between the drugs and placebo groups, with no notable differences. As a result, instead of conducting a quantitative analysis, the SAEs were listed between the two groups for comparison. A similar proportion of patients across the included studies reported death. Among the causes of death reported in the studies, one patient died from severe sepsis (belimumab), and 3 patients died from cerebral hemorrhage (placebo), COVID-19 (zilucoplan), and cardiac event (rituximab). These deaths were not deemed to be associated with the monoclonal antibody drugs. Detailed descriptions of SAEs and morality are shown in **Supplementary Table S5**. Safety outcomes of our NMA showed that the monoclonal antibodies were well tolerated and safe.

### 3.3 Pairwise meta-analysis

The results of pairwise meta-analysis are presented in **Supplementary Figures S10**-**S18**. Since most trials focused on comparing monoclonal antibodies to placebo and various dosages of single monoclonal antibodies, the results generally remained consistent with those obtained from the NMAs.

### 3.4 Additional analysis

**Figure 4:**
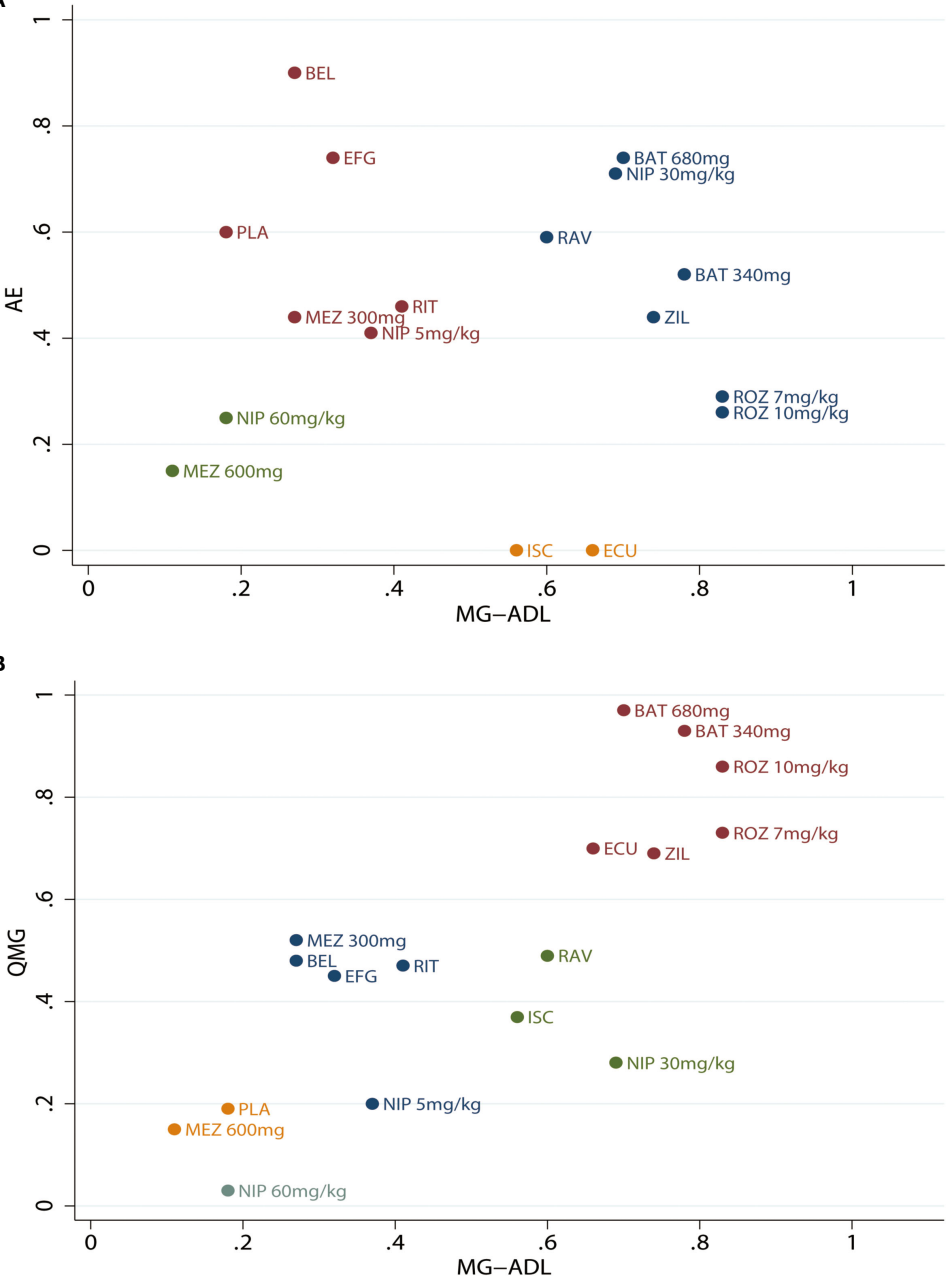
Clustered ranking plot for different monoclonal antibodies. **(A)** Clustered ranking plot according to the Myasthenia Gravis Activities of Daily Living (MG-ADL) score and Quantitative Myasthenia Gravis (QMG) score. **(B)** Myasthenia Gravis Activities of Daily Living (MG-ADL) score and adverse effects (AEs). ROZ, rozanolixzumab; BAT, batoclimab; EFG, efgartigimod; NIP, nipocalimab; ECU, eculizumab; ZIL, zilucoplan; RAV, ravulizumab; BEL, belimumab; RIT, rituximab; ISC, iscalimab; MEZ, mezagitamab; PLA, placebo;.

Cluster ranking was performed based on the MG-ADL score, QMG score, and AEs in **Figure 4**. Batoclimab and rozanolixzumab were the top 2 monoclonal antibodies when considering MG-ADL and QMG scores. However, the cluster ranking for MG-ADL score and AEs demonstrated that batoclimab 680mg might be the optimal regimen balancing efficacy and safety. Otherwise, the ranking probabilities of different monoclonal antibodies regarding efficacy and safety outcomes were present in **Supplementary Figure S20** and **Figure S21**.

### 3.5 Risk of bias, convergence, heterogeneity, and sensitivity analysis

The results of the risk of bias are illustrated in **Supplementary Figure S1**. Most enrolled studies were deemed to have a low risk of bias. However, two studies remained unclear regarding selective reporting bias; one study exhibited blinding of participants and personnel bias, while five had unclear and high other bias. Publication bias was assessed for the outcomes using a funnel plot, and the funnel plot was symmetrically distributed, indicating no publication bias in our study in **Supplementary Figures S46-52**.

The convergence diagnostics for the calculated model were provided in **Supplementary Figures S22**-**S37**. All potential scale reduction factor values were constrained to 1, no noticeable fluctuation was observed, and both trace and density graphs exhibited normal distribution, all pointing to satisfactory and excellent convergence efficacy of our NMA. Global inconsistency was assessed by constructing the consistency model and inconsistency model. The minor differences in DIC and other parameters between fixed and random-effects models indicated minimal inconsistency in the model, which also demonstrated reliability and stability of our findings (**Supplement Table S6**).

Furthermore, we conducted the heterogeneity analysis on various outcomes. The results demonstrated that most comparisons were associated with lower heterogeneity, except the studies by Nowak et al. and Piehl et al. (**Supplement Figures S38-45**). Sensitivity analyses were further performed by individually removing the trials with high heterogeneity that could threaten validity and comparing the results with the primary analysis. The sensitivity results primarily aligned with the primary analysis. However, batoclimab was not superior to placebo and other monoclonal antibodies in terms of QMG score after removing Piehl et al., which was an RCT included patients of lower age and higher AChR antibody levels in baseline and possibly leading to increased heterogeneity (**Supplement Tables S7** **-** **18**). Overall, the low level of heterogeneity across comparisons of different monoclonal drugs suggested the robustness of our NMA results.

## 4 Discussion

Our network meta-analysis incorporated the most comprehensive data available and performed a synthetic analysis of the efficacy of monoclonal antibodies in the treatment of generalized MG. Eculizumab, rozanolixzumab 7mg/kg, rozanolixzumab 10mg/kg, and zilucoplan all demonstrated effectiveness compared to placebo across all efficacy outcomes, including the MG-ADL, QMG, MGC, and MG-QoL 15r scores. Based on the SUCRA values, rozanolixzumab 10mg/kg and batoclimab 680mg ranked first in MG-ADL score and QMG score, respectively. Additionally, belimumab demonstrated a significant statistical difference in AEs compared to rozanolixzumab and mezagitamab.

### 4.1 FcRn inhibitors

In generalized MG, pathogenic IgG autoantibodies target specific proteins on the post-synaptic membrane and impair synaptic transmission at the neuromuscular junction, thereby preventing muscle contraction (42). FcRn inhibitors bind to FcRn with high affinity and inhibit the interaction between FcRn and lgG, accelerating the catabolism and reducing the concentration of pathogenic IgG autoantibodies (43). However, it is important that FcRn inhibitors may increase the risk of infections due to reduced IgG levels.

In our network meta-analysis, FcRn inhibitors mainly included rozanolixzumab, batoclimab, efgartigimod, and nipocalimab. Batoclimab was the first subcutaneous drug to reduce pathogenic antibodies with the advantage of self-administration, which can improve patient compliance (44). In the present study, batoclimab 340mg and batoclimab 680mg were more effective than the placebo in improving QMG score and MG-QoL 15r score. Additionally, batoclimab 340mg and batoclimab 680mg were more effective than other monoclonal antibodies in improving the QMG score, except for rozanolixzumab. Importantly, batoclimab exhibited good tolerability, with no higher incidence of AEs than other drugs. It was noted that batoclimab was associated with hypercholesterolemia. However, patients often experience a quick recovery after medication without causing related cardiovascular events (34). A randomized trial investigating the efficacy of batoclimab for thyroid eye disease reported an increase in serum cholesterol, which subsequently decreased upon discontinuation (45). The results of our network meta-analysis supported the evidence of phase III clinical trials, further confirming the efficacy and safety of batoclimab for patients with generalized MG (NCT05403541).

Rozanolixzumab was the latest FDA-approved FcRn inhibitor in the United States for treating generalized MG in adult patients with positive AChR and MuSK antibodies in 2023 (20). As mentioned earlier, rozanolixzumab proved to be more effective than placebo in all efficacy measures. It also demonstrated superior efficacy compared to belimumab, efgartigimod, mezagitamab 600mg in reducing MG-ADL and MGC scores. Recently, a network meta-analysis systematically compared FcRn inhibitors with complement inhibitors and found that the FcRn inhibitors had a higher reduction in QMG changes, with no significant difference in MG-ADL score change. Furthermore, rozanolixzumab 10mg/kg dosage was more effective than rozanolixzumab 7mg/kg in primary efficacy outcomes (46), consistent with our findings. Regarding AEs, rozanolixzumab did not demonstrate superiority over other monoclonal antibodies and had more AEs than batoclimab.

Additionally, it was associated with a higher incidence of headaches. However, most of these headaches ranged from mild to moderate and could be controlled by non-opioid analgesics (47). Although the decrease in IgG levels caused by FcRn inhibition was associated with a potential increase in susceptibility to infections, no severe infections were observed.

Efgartigimod was the first intravenous FcRn inhibitor approved by the United States FDA, Japan, and the European Union for treating generalized MG (48, 49). In the present study, efgartigimod showed better effectiveness than the placebo in improving both the QMG score and MG-QoL 15r score. Patients with positive MuSK antibodies, or those who are seronegative, responded similarly to patients with positive AChR antibodies in generalized MG. Consequently, efgartigimod can effectively reduce the levels of pathogenic IgG autoantibody regardless of autoantibody status, making it broader applications for treating generalized MG than monoclonal antibodies approved exclusively for the positive AChR antibody. Moreover, lower IgG levels may increase the risk of infections. Our NMA found no significant difference in total or individual AEs.

### 4.2 Complement inhibitors

Complement activation and the subsequent formation of membrane attack complexes damaged the postsynaptic membrane and impaired neuromuscular transmission, leading to skeletal muscle weakness (50). Complement inhibitors, mainly C5 inhibitors, exhibited a high affinity for C5, inhibiting its cleavage into C5a and C5b, ultimately preventing the formation of membrane attack complexes (51). Our network meta-analysis evaluated the effectiveness of three complement inhibitors including zilucoplan, eculizumab, and ravulizumab.

In 2017, the United States FDA approved eculizumab, the first approved complement inhibitor for the treatment of generalized MG in adult patients with positive AChR antibodies (17). In our NMA, eculizumab demonstrated more significant clinical improvement than other complement inhibitors in MG-ADL, MGC, and QMG scores. An observational study further supported the effectiveness of eculizumab by showing improved outcomes in terms of changes from baseline QMG score and a higher rate of minimal manifestation compared with rituximab in patients to treat generalized anti-AChR-antibodies positive MG (52). However, it is essential to acknowledge that complement inhibitors increase the risk of Neisseria meningitides infection, and patients with generalized MG treated with complement inhibitors must receive vaccination (53). Ravulizumab was developed to extend the dosing interval to 8 weeks by prolonging the antibody half-life, eliminating the need for the two-weekly intravenous dosing schedule of eculizumab, which received approval for the treatment of generalized MG with positive AChR in 2022 (18). Zilucoplan was developed as an alternative for eculizumab resistance in Paroxysmal Nocturnal Hemoglobinuria and obtained new drug application and validation for the treatment of generalized MG (54, 55). The previously mentioned complement inhibitors exhibited superiority over the placebo in MG-ADL, QMG, and MG-QoL 15r scores. In addition, eculizumab demonstrated superiority over ravulizumab and zilucoplan in QMG and MG-QoL 15r scores. While eculizumab showed superiority to other complement inhibitors, its high cost exceeded traditional cost-effectiveness thresholds. Moreover, the inadequate clinical evidence to distinguish the benefits of eculizumab from conventional therapies posed significant challenges to its widespread adoption (56).

### 4.3 B-cell targeting therapies

B cells can secrete antibodies, including pathogenic antibodies, and were considered the primary effector cells in the pathogenesis of generalized MG (57). B-cell targeting therapies mainly included direct B-cell inhibitors and indirect B-cell inhibitors. Rituximab is an anti-CD20 glycoprotein monoclonal antibody (58). It can diminish the short-lived plasma cells secreting MuSK antibodies rather than the long-lived plasma cells secreting AChR antibodies (59). Additionally, two studies demonstrated that patients with positive MuSK antibodies had a more favorable response to rituximab than those with anti-AChR antibody-positive generalized MG (60, 61). In the present studies, there were no significant differences in clinical improvement as measured by the MG-ADL and QMG scores. Notably, most patients in the study who received rituximab treatment were positive for AChR antibodies.

Furthermore, different types of plasma cells (short-lived and long-lived) exhibited distinct responses to rituximab treatment, which could explain our study’s observed outcomes. The 2020 International Consensus Guideline stated that the effectiveness of rituximab in refractory AChR antibody-positive MG remained uncertain, and it should be considered as an alternative when the side effects of immunosuppressive agents become intolerable (12). Moreover, rituximab was well-tolerated, and the incidence of AEs did not show a statistical difference from the placebo, as indicated by the lower SUCRA value.

Our network meta-analysis included two drugs classified as indirect B-cell inhibitors. Belimumab, which targets the B-cell activating factor, inhibited the suppression of the B-cell activating factor and led to the depletion of B cell lineage cells (62). Iscalimab was an anti-CD 40 monoclonal antibody that binds to CD154 expressed in activated T cells, effectively blocking the CD40 signaling pathway (63). Although there was no statistical difference in the rate of AEs compared to placebo, significant clinical improvement in all efficacy measures was not reached. These newer biological agents offered the advantage of being fully humanized with fever side-effect and better tolerance. Therefore, large and long-term randomized clinical trials are needed to explore their efficacy further.

This study had several limitations. Firstly, the limited numbers of RCTs were included, pooling data from 13 RCTs and 1167 patients, which could be a source of the non-statistically significant differences in outcome measures. Secondly, the confidence rating for many comparisons in our study was rated as low or very low evidence due to higher levels of indirectness and incoherence according to the results of CINeMA, which may be associated with the indirect comparisons among the different monoclonal antibodies included in the trials. Based on the indirect evidence, this may limit the reliability of our study. Thirdly, the small size of several phase II clinical trials and the length of follow-up were also limitations, which will be addressed by the phase III clinical trials and the ongoing open-label extension studies. Lastly, the data on factors, such as the percentage of antibodies-negative patients, changes in serum IgG levels, and antibody levels from baseline, were unavailable in some literature. Additionally, variations in the study design, inclusion and exclusion criteria, and baseline characteristics (e.g., mean age, sex ratio, and disease duration) may have contributed to discrepant statistics.

## 5 Conclusion

In conclusion, our findings showed that most monoclonal antibodies were more effective than placebo in the efficacy outcomes. Rozanolixzumab and batoclimab may have the highest probability of being the most effective treatment for generalized MG. However, rozanolixzumab was associated with a higher incidence of AEs. Although our network meta-analysis provided the evidence base for the following phase III clinical trials and open-label extension studies, considering the limitations of indirect comparison of NMA, more head-to-head RCTs and extensive observational studies are needed to confirm our findings.

## Author contributions

HC: Data curation, Methodology, Writing – original draft, Software. YQ: Writing – original draft, Methodology, Software. ZY: Methodology, Software, Writing – review & editing, Data curation. ZiW: Methodology, Writing – review & editing. YT: Data curation, Writing – review & editing. HN: Data curation, Writing – review & editing. JL: Data curation, Writing – review & editing. ZC: Supervision, Writing – review & editing. YK: Writing – review & editing, Supervision. ZhW: Writing – review & editing, Supervision.
