# Supplementary material for: Efficacy and safety of the innovative monoclonal antibodies in adults with generalized myasthenia gravis: a Bayesian network analysis
Source: Front Immunol. 2023 Nov 8;14:1280226. doi: 10.3389/fimmu.2023.1280226 (PMC10663412; doi:10.3389/fimmu.2023.1280226)
Supplement: Supplementary file 1 [file DataSheet_1.docx]

**Efficacy and Safety of the Innovative Monoclonal Antibodies in Adults with Generalized Myasthenia Gravis: a Bayesian Network Analysis**

**Huiru Chen^1,2†^, Youjia Qiu^1,†^ , Ziqian Yin^1,†^, Zilan Wang^1^ , Yanbing Tang^3^, Hanyu Ni^3^, Jiaye Lu^1^, Zhouqing Chen^1, *^, Yan Kong^2, *^, Zhong Wang^1, *^**

*^1^* *Department of Neurosurgery & Brain and Nerve Research Laboratory, The First Affiliated Hospital of Soochow University, Suzhou, Jiangsu Province, 215006, China*

*^2^ Department of Neurology, The First Affiliated Hospital of Soochow University, Suzhou, Jiangsu Province, 215006, China*

*^3^* *Suzhou Medical College of Soochow University, Suzhou, Jiangsu Province, 215002, China*

†Huiru Chen, Youjia Qiu and Ziqian Yin contribute equally to this work.

*** Correspondence:**

Zhong Wang, the First Affiliated Hospital of Soochow University, 188 Shizi Street, Suzhou, Jiangsu Province, 215006, China. E-mail address: wangzhong761@163.com

Yan Kong, the First Affiliated Hospital of Soochow University, 899 Pinghai road, Suzhou, Jiangsu Province, 215006, China. E-mail address: [kong0919@163.com](mailto:kong0919@163.com)

Zhouqing Chen, the First Affiliated Hospital of Soochow University, 188 Shizi Street, Suzhou, Jiangsu Province, 215006, China. E-mail address: [zqchen6@163.com](mailto:zqchen6@163.com)

**Table legends**

Table S1: The detailed search strategy.

Table S2: Appendix of detailed excluded studies.

Table S3: Inclusion, Exclusion criteria, Study design and Outcome assessments of the included studies.

Table S4: Certainty of evidence for primary outcomes and efficacy outcome in league table.

Table S5: The events in the SAEs and all-cause Mortality of the included studies.

Table S6: The deviance information criteria (DIC) and I^2^ values of fixed-effects model and random-effects model.

Table S7: Network meta-analysis results of MG-ADL without studies of Nowak 2021.

Table S8: Network meta-analysis results of QMG without studies of Nowak 2021.

Table S9: Network meta-analysis results of MGC without studies of Nowak 2021.

Table S10: Network meta-analysis results of AEs without studies of Nowak 2021.

Table S11: Network meta-analysis results of Headache without studies of Nowak 2021.

Table S12: Network meta-analysis results of Diarrhea without studies of Nowak 2021.

Table S13: Network meta-analysis results of Nausea without studies of Nowak 2021.

Table S14: Network meta-analysis results of MG-ADL without studies of Piehl 2022.

Table S15: Network meta-analysis results of QMG without studies of Piehl 2022.

Table S16: Network meta-analysis results of AEs without studies of Piehl 2022.

Table S17: Network meta-analysis results of Diarrhea without studies of Piehl 2022.

Table S18: Network meta-analysis results of Nausea without studies of Piehl 2022.

**Figure legends**

Figure S1 Risk of bias.

Figure S2 Forest plots of the network meta-analysis: MG-ADL.

Figure S3 Forest plots of the network meta-analysis: QMG.

Figure S4 Forest plots of the network meta-analysis: MGC.

Figure S5 Forest plots of the network meta-analysis: MG-QoL 15r.

Figure S6 Forest plots of the network meta-analysis: AEs.

Figure S7 Forest plots of the network meta-analysis: Headache.

Figure S8 Forest plots of the network meta-analysis: Diarrhea.

Figure S9 Forest plots of the network meta-analysis: Nausea.

Figure S10: Pairwise meta-analysis of efficacy: MG-ADL.

Figure S11: Pairwise meta-analysis of efficacy: QMG.

Figure S12: Pairwise meta-analysis of efficacy: MGC.

Figure S13: Pairwise meta-analysis of efficacy: MG-QoL 15r.

Figure S14: Pairwise meta-analysis of safety: AEs.

Figure S15: Pairwise meta-analysis of safety: SAEs.

Figure S16: Pairwise meta-analysis of safety: Headache.

Figure S17: Pairwise meta-analysis of safety: Diarrhea.

Figure S18: Pairwise meta-analysis of safety: Nausea.

Figure S19: League tables of the headache, diarrhea, and Nausea.

Figure S20: Cumulative probability of different monoclonal antibodies for efficacy outcome.

Figure S21: Cumulative probability of different monoclonal antibodies for safety outcome.

Figure S22: Convergence diagnostics of the network meta-analysis: MG-ADL.

Figure S23: Convergence diagnostics of the network meta-analysis: QMG.

Figure S24: Convergence diagnostics of the network meta-analysis: MGC.

Figure S25: Convergence diagnostics of the network meta-analysis: MG-QoL 15r.

Figure S26: Convergence diagnostics of the network meta-analysis: AEs.

Figure S27: Convergence diagnostics of the network meta-analysis: Headache.

Figure S28: Convergence diagnostics of the network meta-analysis: Diarrhea.

Figure S29: Convergence diagnostics of the network meta-analysis: Nausea.

Figure S30: Trance and density of the network meta-analysis: MG-ADL.

Figure S31: Trance and density of the network meta-analysis: QMG.

Figure S32: Trance and density of the network meta-analysis: MGC.

Figure S33: Trance and density of the network meta-analysis: MG-QoL 15r.

Figure S34: Trance and density of the network meta-analysis: AEs.

Figure S35: Trance and density of the network meta-analysis: Headache.

Figure S36: Trance and density of the network meta-analysis: Diarrhea.

Figure S37: Trance and density of the network meta-analysis: Nausea.

Figure S38: Forest plots for the heterogeneity: MG-ADL.

Figure S39: Forest plots for the heterogeneity: QMG.

Figure S40: Forest plots for the heterogeneity: MGC.

Figure S41: Forest plots for the heterogeneity: MG-QoL 15r.

Figure S42: Forest plots for the heterogeneity: AEs.

Figure S43: Forest plots for the heterogeneity: Headache.

Figure S44: Forest plots for the heterogeneity: Diarrhea.

Figure S45: Forest plots for the heterogeneity: Nausea.

Figure S46: Funnel plots for MG-ADL.

Figure S47: Funnel plots for QMG.

Figure S48: Funnel plots for MGC.

Figure S49: Funnel plots for MG-QoL 15r.

Figure S50: Funnel plots for Headache.

Figure S51: Funnel plots for Diarrhea.

Figure S52: Funnel plots for Nausea.

**Table S1: Detailed Search Strategy**

**PubMed:**

| Search | Query | Results |
| --- | --- | --- |
| #1 | "Myasthenia Gravis"[Mesh] | 16,950 |
| #2 | (((((((((Ocular Myasthenia Gravis[Title/Abstract]) OR (Generalized Myasthenia Gravis[Title/Abstract])) OR (Muscle-Specific Receptor Tyrosine Kinase Myasthenia Gravis[Title/Abstract])) OR (Muscle Specific Receptor Tyrosine Kinase Myasthenia Gravis[Title/Abstract])) OR (Muscle-Specific Tyrosine Kinase Antibody Positive Myasthenia Gravis[Title/Abstract])) OR (Muscle Specific Tyrosine Kinase Antibody Positive Myasthenia Gravis[Title/Abstract])) OR (MuSK MG[Title/Abstract])) OR (MuSK Myasthenia Gravis[Title/Abstract])) OR (Anti-MuSK Myasthenia Gravis[Title/Abstract])) OR (Anti MuSK Myasthenia Gravis[Title/Abstract]) | 1,091 |
| #3 | #1 OR #2 | 17,144 |
| #4 | "Antibodies, Monoclonal" [MeSH Terms] | 276,149 |
| #5 | Monoclonal Antibody [Title/Abstract] | 115,185 |
| #6 | #4 OR #5 | 322,487 |
| #7 | "Fc receptor, neonatal" [MeSH Terms] | 454 |
| #8 | (((((((((((Fc receptors, neonatal[Title/Abstract]) OR (receptor, Fc neonatal[Title/Abstract])) OR (FcRn neonatal transfer protein[Title/Abstract])) OR (neonatal Fc receptor[Title/Abstract])) OR (Fcgrt protein, mouse[Title/Abstract])) OR (FcRn protein, mouse[Title/Abstract])) OR (Fc receptor, IgG, alpha chain transporter, mouse[Title/Abstract])) OR (neonatal Fc receptor, mouse[Title/Abstract])) OR (FCGRT protein, human[Title/Abstract])) OR (FcRn protein, human[Title/Abstract])) OR (Neonatal Fc receptor, human[Title/Abstract])) OR (Fc fragment of IgG, receptor, transporter, alpha protein, human[Title/Abstract]) | 1,274 |
| #9 | #7 OR #8 | 1,531 |
| #10 | "Complement Inactivating Agents" [MeSH Terms] | 1,344 |
| #11 | (((Complement Inhibitor [Title/Abstract]) OR (Complement Inhibitors [Title/Abstract])) OR (Complement Inhibiting Agents [Title/Abstract])) OR (Complement Cytolysis Inhibiting Agents [Title/Abstract]) | 1,693 |
| #12 | #10 OR #11 | 2,748 |
| #13 | "Antigens, CD" [MeSH Terms] | 92,586 |
| #14 | ((((((((CD Antigen [Title/Abstract]) OR (Cluster of Differentiation Antigens [Title/Abstract])) OR (Cluster of Differentiation Antigen [Title/Abstract])) OR (Cluster of Differentiation Marker [Title/Abstract])) OR (Cluster of Differentiation Markers [Title/Abstract])) OR (Antigen Cluster, Differentiation [Title/Abstract])) OR (Differentiation Antigen Cluster [Title/Abstract])) OR (Differentiation Marker Cluster [Title/Abstract])) OR (Leukocyte Differentiation Antigens, Human [Title/Abstract]) | 265 |
| #15 | #13 OR #14 | 92,779 |
| #16 | #6 OR #9 OR #12 OR #15 | 406,504 |
| #17 | #3 AND #16 | **826** |
| #18 |  |  |

**Embase:**

| Search | Query | Results |
| --- | --- | --- |
| #1 | 'myasthenia gravis'/exp | 28,466 |
| #2 | 'ocular myasthenia gravis':ab,ti | 607 |
| #3 | 'generalized myasthenia gravis':ab,ti | 749 |
| #4 | 'muscle specific receptor tyrosine kinase myasthenia gravis':ab,ti | 1 |
| #5 | 'muscle specific tyrosine kinase antibody positive myasthenia gravis':ab,ti | 14 |
| #6 | 'musk mg':ab,ti | 317 |
| #7 | 'musk myasthenia gravis':ab,ti | 131 |
| #8 | 'anti-musk myasthenia gravis':ab,ti | 31 |
| #9 | #1 OR #2 OR #3 OR #4 OR #5 OR #6 OR #7 OR #8 | 28,541 |
| #10 | 'antibodies, monoclonal'/exp | 747,601 |
| #11 | 'monoclonal antibody':ab,ti | 147,267 |
| #12 | #10 OR #11 | 783,157 |
| #13 | 'neonatal fc receptor'/exp | 99 |
| #14 | 'receptor, fc neonatal':ab,ti | 1 |
| #15 | 'fc receptor, neonatal ':ab,ti | 3 |
| #16 | #13 OR #14 OR #15 | 103 |
| #17 | 'complement inactivating agents'/exp | 2,857 |
| #18 | 'complement inhibitor':ab,ti | 1,528 |
| #19 | 'complement inhibitors':ab,ti | 1,228 |
| #20 | 'complement inhibiting agents':ab,ti | 11 |
| #21 | #17 OR #18 OR #19 OR #20 | 4,109 |
| #22 | 'antigens, cd'/exp | 1,055,676 |
| #23 | 'cd antigen':ab,ti | 99 |
| #24 | 'cluster of differentiation antigens':ab,ti | 22 |
| #25 | 'cluster of differentiation antigen':ab,ti | 70 |
| #26 | 'cluster of differentiation marker':ab,ti | 20 |
| #27 | 'cluster of differentiation markers':ab,ti | 36 |
| #28 | 'antigen cluster, differentiation':ab,ti | 1 |
| #29 | 'differentiation antigen cluster':ab,ti | 4 |
| #30 | #22 OR #23 OR #24 OR #25 OR #26 OR #27 OR #28 OR #29 | 1,055,770 |
| #31 | #12 OR #16 OR #21 OR #30 | 1,711,324 |
| #32 | #9 AND #31 | 4,041 |
| #33 | 'random':ab,ti OR 'control':ab,ti OR 'double-blind':ab,ti | 4,519,982 |
| #34 | #32 AND #33 | **444** |

**Cochrane:**

| Search | Query | Results |
| --- | --- | --- |
| #1 | MeSH descriptor: [Myasthenia Gravis] explode all trees | 336 |
| #2 | (Ocular Myasthenia Gravis OR Generalized Myasthenia Gravis OR MuSK Myasthenia Gravis OR Muscle-Specific Receptor Tyrosine Kinase Myasthenia Gravis OR Anti-MuSK Myasthenia Gravis OR MuSK MG OR Muscle Specific Tyrosine Kinase Antibody Positive Myasthenia Gravis):ti,ab,kw | 337 |
| #3 | #1 or #2 | 552 |
| #4 | MeSH descriptor: [Antibodies, Monoclonal] explode all trees | 18,796 |
| #5 | (Monoclonal Antibody):ti,ab,kw | 12,500 |
| #6 | #4 or #5 | 27,087 |
| #7 | MeSH descriptor: [Fc Receptors] explode all trees | 909 |
| #8 | (Receptor, Fc Fc Receptors):ti,ab,kw | 296 |
| #9 | #7 OR #8 | 376 |
| #10 | MeSH descriptor: [Complement Inactivating Agents] explode all trees | 252 |
| #11 | (Complement Cytolysis Inhibiting Agents OR Complement Inhibiting Agents OR Complement Inhibitors OR Complement Inhibitor) :ti,ab,kw | 934 |
| #12 | #10 OR #11 | 1,186 |
| #13 | MeSH descriptor: [Antigens, CD] explode all trees | 1,121 |
| #14 | (Antigen Cluster, Differentiation OR CD Antigen OR Cluster of Differentiation Antigens OR Cluster of Differentiation Antigen OR Cluster of Differentiation Markers OR Differentiation Antigen Cluster OR Cluster of Differentiation Marker OR Differentiation Marker Cluster OR Leukocyte Differentiation Antigens, Human):ti,ab,kw | 535 |
| #15 | #13 OR #14 | 1,439 |
| #16 | #6 OR #9 OR #12 OR #15 | 29,354 |
| #17 | #3 AND #16 | **96** |

**Clinicaltrials.gov**

| Search | Query | Results |
| --- | --- | --- |
| #1 | Status: All studies, condition or disease: Myasthenia Gravis, study Results: with results | 33 |
| Total |  | **33** |

**TableS2: Appendix of detailed excluded studies.**

| **(1). Post-hoc analysis (n=27)**   1. Vissing J, O'Brien F, Wang JJ, Howard JF, Jr. Correlation between myasthenia gravis-activities of daily living (MG-ADL) and quantitative myasthenia gravis (QMG) assessments of anti-acetylcholine receptor antibody-positive refractory generalized myasthenia gravis in the phase 3 regain study. Muscle Nerve (2018) 58: E21-e22.doi.org/10.1002/mus.26152. 2. Vanoli F, Mantegazza R. Ravulizumab for the treatment of myasthenia gravis. Expert Opin Biol Ther (2023) 23: 235-241.doi.org/10.1080/14712598.2023.2185131. 3. Uzawa A, Juel V, Vu T, Casasnovas C, Aguzzi R, Frick G, et al. Efficacy of ravulizumab across sex and age subgroups of patients with generalized myasthenia gravis: A post hoc analysis of the CHAMPION MG study. Clinical and Experimental Neuroimmunology (2023) 14: 72.doi.org/10.1111/cen3.12738. 4. Suzuki Y, Genge A, Hussain Y, Kaminski HJ, Leite MI, Mantegazza R, et al. Efficacy and safety of zilucoplan in myasthenia gravis: Responder analysis from the randomized Phase 3 RAISE trial. Clinical and Experimental Neuroimmunology (2023) 14: 70.doi.org/10.1111/cen3.12738. 5. Singh N, Goyal V. Rituximab as induction therapy in refractory myasthenia gravis: 18 month follow-up study. J Neurol (2019) 266: 1596-1600.doi.org/10.1007/s00415-019-09296-y. 6. Saccà F, Barnett C, Vu T, Peric S, Phillips GA, Zhao S, et al. Efgartigimod improved health-related quality of life in generalized myasthenia gravis: results from a randomized, double-blind, placebo-controlled, phase 3 study (ADAPT). J Neurol (2023) 270: 2096-2105.doi.org/10.1007/s00415-022-11517-w. 7. Ramchandren S, Guptill J, Antozzi C, Bril V, Gamez J, Meuth S, et al. Serum Autoantibody Lowering by the Anti-FcRn Monoclonal Antibody, Nipocalimab, Correlates With Clinical Improvement in Generalized Myasthenia Gravis Patients. Neurology (2022) 93: S35‐S37.doi.org/10.1212/01.wnl.0000903616.08384.e6. 8. Nowak RJ, Muppidi S, Beydoun SR, O'Brien FL, Yountz M, Howard JF, Jr. Concomitant Immunosuppressive Therapy Use in Eculizumab-Treated Adults With Generalized Myasthenia Gravis During the REGAIN Open-Label Extension Study. Front Neurol (2020) 11: 556104.doi.org/10.3389/fneur.2020.556104. 9. Murai H, Uzawa A, Suzuki Y, Imai T, Shiraishi H, Tsuda E, et al. Open label extension trial of regain to evaluate the safety and efficacy of eculizumab in patients from Japan with refractory generalized myasthenia gravis. Journal of the Neurological Sciences (2017) 381: 132-133.doi.org/10.1016/j.jns.2017.08.397. 10. Murai H, Uzawa A, Suzuki Y, Imai T, Shiraishi H, Suzuki H, et al. Long-term efficacy and safety of eculizumab in Japanese patients with generalized myasthenia gravis: A subgroup analysis of the REGAIN open-label extension study. Journal of the Neurological Sciences (2019) 407.doi.org/10.1016/j.jns.2019.08.004. 11. Murai H, Suzuki S, Hasebe M, Fukamizu Y, Rodrigues E, Utsugisawa K. Safety and effectiveness of eculizumab in Japanese patients with generalized myasthenia gravis: interim analysis of post-marketing surveillance. Therapeutic Advances in Neurological Disorders (2021) 14.doi.org/10.1177/17562864211001995. 12. Murai H, Suzuki S, Fukamizu Y, Osawa T, Kikui H, Utsugisawa K. Safety and effectiveness of eculizumab in Japanese patients with generalized myasthenia gravis: Analysis of 1-year postmarketing surveillance. Clinical and Experimental Neuroimmunology (2022) 13: 280-289.doi.org/10.1111/cen3.12716. 13. Muppidi S, Utsugisawa K, Benatar M, Murai H, Barohn RJ, Illa I, et al. Long-term safety and efficacy of eculizumab in generalized myasthenia gravis. Muscle and Nerve (2019) 60: 14-24.doi.org/10.1002/mus.26447. 14. Mantegazza R, Wolfe GI, Muppidi S, Wiendl H, Fujita KP, O'Brien FL, et al. Post-intervention Status in Patients With Refractory Myasthenia Gravis Treated With Eculizumab During REGAIN and Its Open-Label Extension. Neurology (2021) 96: e610-e618.doi.org/10.1212/wnl.0000000000011207. 15. Mantegazza R, O'Brien FL, Yountz M, Howard JF, Jr. Consistent improvement with eculizumab across muscle groups in myasthenia gravis. Ann Clin Transl Neurol (2020) 7: 1327-1339.doi.org/10.1002/acn3.51121. 16. Keung B, Robeson KR, DiCapua DB, Rosen JB, O'Connor KC, Goldstein JM, et al. Long-term benefit of rituximab in MuSK autoantibody myasthenia gravis patients. J Neurol Neurosurg Psychiatry (2013) 84: 1407-1409.doi.org/10.1136/jnnp-2012-303664. 17. Howard JF, Jr., Karam C, Yountz M, O'Brien FL, Mozaffar T. Long-term efficacy of eculizumab in refractory generalized myasthenia gravis: responder analyses. Ann Clin Transl Neurol (2021) 8: 1398-1407.doi.org/10.1002/acn3.51376. 18. Guptill J, Antozzi C, Bril V, Gamez J, Meuth S, Nowak R, et al. Serum IgGand Autoantibody Lowering by the Anti-FcRn Monoclonal Antibody, Nipocalimab, Correlates with Improvement in MG-ADL in Generalized Myasthenia Patients. Neurology (2022) 98. 19. Chan F, Swayne A, Gillis D, Walsh M, Henderson RD, McCombe PA, et al. Long-term follow-up of patients with myasthenia gravis treated with low-dose rituximab. J Neurol Neurosurg Psychiatry (2019) 90: 955-956.doi.org/10.1136/jnnp-2018-319410. 20. Bril V, Druzdz A, Grosskreutz J, Habib A, Mantegazza R, Sacconi S, et al. Rozanolixizumab in Generalized Myasthenia Gravis: Responder Analyses from the Phase 3 MycarinG Study. Journal of Neuromuscular Diseases (2022) 9: S106-S108.doi.org/10.3233/JND-229001. 21. Beecher G, Anderson D, Siddiqi ZA. Rituximab in refractory myasthenia gravis: Extended prospective study results. Muscle Nerve (2018) 58: 452-455.doi.org/10.1002/mus.26156. 22. Andersen H, Mantegazza R, Wang JJ, O'Brien F, Patra K, Howard JF, Jr. Eculizumab improves fatigue in refractory generalized myasthenia gravis. Qual Life Res (2019) 28: 2247-2254.doi.org/10.1007/s11136-019-02148-2. 23. Kaminski H, Zhao C, zu Horste GM, O'Connor K, Klingelschmitt G, Krumova P, et al. P.92 UMINESCE: phase 3 study of satralizumab, a therapeutic recycling antibody targeting the IL-6 receptor, in patients with generalised myasthenia gravis. Neuromuscular disorders (2022) 32: S80‐S81.doi.org/10.1016/j.nmd.2022.07.162. 24. Siddiqi ZA, Nowak RJ, Mozaffar T, O'Brien F, Yountz M, Patti F. Eculizumab in refractory generalized myasthenia gravis previously treated with rituximab: subgroup analysis of REGAIN and its extension study. Muscle Nerve (2021) 64: 662-669.doi.org/10.1002/mus.27422. 25. Howard JF, Jr., Freimer M, O'Brien F, Wang JJ, Collins SR, Kissel JT. QMG and MG-ADL correlations: Study of eculizumab treatment of myasthenia gravis. Muscle Nerve (2017) 56: 328-330.doi.org/10.1002/mus.25529. 26. Ghanima W, McDonald V, Jain S, Carpenedo M, Oliva EN, Hultberg A, et al. Pharmacokinetic / Pharmacodynamic (PK/PD) Simulations Guide Selection of the Dose for Administration of Efgartigimod Subcutaneously in a Phase 3 Clinical Trial in Patients with Primary Immune. Thrombocytopenia Blood (2021) 138: 3165.doi.org/10.1182/blood-2021-153261. 27. Chow V, Pan J, Chien D, Mytych D, Hanes V. Pharmacokinetic and pharmacodynamic similarity of ABP 959 with eculizumab: Results from a randomized, double-blind, single-dose, parallel group study in healthy subjects. HemaSphere (2019) 3: 125. |
| --- |
| **(2). Conference abstract (n=54)**   1. Yan C, Duan R, Yang H, Li H, Zou Z, Zhang H, et al. VP.38 A double-blinded, randomized, placebo-controlled phase II study of FcRn antagonist batoclimab in Chinese generalized myasthenia gravis patients. Neuromuscular Disorders (2022) 32: S82.doi.org/10.1016/j.nmd.2022.07.169. 2. Weston-Davies W, Westwood JP, Nunn M. Phase 1 clinical trial of novel complement C5 inhibitor coversin. Molecular Immunology (2013) 56: 264.doi.org/10.1016/j.molimm.2013.05.072. 3. Vu T, Meisel A, Mantegazza R, Annane D, Katsuno M, Aguzzi R, et al. Efficacy and Safety of Ravulizumab, a Long Acting Terminal Complement Inhibitor, in Adults with Anti-Acetylcholine Receptor Antibody Positive Generalized Myasthenia Gravis: Results from the Phase 3 CHAMPION MG. Study Neurology (2022) 98. 4. Vissing J, Jacob S, Fujita K, O'Brien F, Howard J. 'minimal symptom expression' with eculizumab in myasthenia gravis. Muscle and Nerve (2018) 58: S97. 5. Nyquist KB, Elsais A, Kerty E. Refractory myasthenia gravis successfully treated with Rituximab. European Journal of Neurology (2019) 26: 898.doi.org/10.1111/ene.14019. 6. Nowak RJ, Coffey C, Goldstein JM, Dimachkie M, Benatar M, O'Connor KC, et al. Phase 2 trial of rituximab in myasthenia gravis: Study update. Annals of Neurology (2015) 78: S109.doi.org/10.1002/ana.24498. 7. Nowak RJ, Coffey C, Goldstein JM, Dimachkie M, Benatar M, Huq S, et al. B cell targeted treatment in myasthenia gravis (beatmg) - A phase 2 trial of rituximab in MG: Topline results. Journal of Neuromuscular Diseases (2018) 5: S341.doi.org/10.3233/JND-189001. 8. Nowak R, Coffey C, Goldstein J, Yankey J, Uribe L, Dimachkie M, et al. Rituximab in patients with moderate to severe myasthenia gravis: A subgroup analysis of the beatmg study. Muscle and Nerve (2019) 60: S139. 9. Murai H, Suzuki S, Hayashi T, Fukamizu Y, Okamura K, Utsugisawa K. Interim analysis of post-marketing surveillance of eculizumab in patients with generalized myasthenia gravis in Japan. Journal of Neuromuscular Diseases (2021) 8: S150.doi.org/10.3233/JND-219006. 10. Murai H, Suzuki S, Hasebe M, Fukamizu Y, Rodrigues E, Utsugisawa K. Safety and effectiveness of eculizumab for patients with generalized myasthenia gravis in Japan: Interim analysis of post-marketing surveillance. European Journal of Neurology (2020) 27: 485-486. 11. Murai H, Suzuki S, Fukamizu Y, Osawa T, Kikui H, Utsugisawa K. Real-world effectiveness of eculizumab in generalized myasthenia gravis in Japan:1-year post-marketing surveillance data. Neurology (2022) 98. 12. Murai H, Howard JF, Wang JJ, O'Brien F, Utsugisawa K. Efficacy of eculizumab is maintained beyond 26 weeks in patients with acetylcholine receptor-positive refractory generalized myasthenia gravis. Clinical and Experimental Neuroimmunology (2017) 8: 365. 13. Mantegazza R, Wolfe G, Muppidi S, Wiendl H, Fujita KP, O'Brien F, et al. Achievement of minimal manifestations in eculizumab-treated achrpositive refractory myasthenia gravis patients. Journal of Neuromuscular Diseases (2018) 5: S355-S356.doi.org/10.3233/JND-189001. 14. Mantegazza R, Wolfe G, Muppidi S, Wiendl H, Fujita K, O'Brien F, et al. Minimal manifestations with eculizumab in myasthenia gravis. Muscle and Nerve (2018) 58: S98. 15. Mantegazza R, Fujita KP, O'Brien F, Howard JF. Eculizumab shows consistency of improvement across muscle groups in patients with achr-positive refractory myasthenia gravis. Journal of Neuromuscular Diseases (2018) 5: S354-S355.doi.org/10.3233/JND-189001. 16. Mantegazza R, Fujita K, O'Brien F, Howard J. Eculizumab shows consistent improvement across quantitative myasthenia gravis test muscle groups. Muscle and Nerve (2018) 58: S97. 17. Jacob S, Murai H, Utsugisawa K, Nowak RJ, Wiendl H, Fujita KP, et al. Response to eculizumab in patients with ACHR+ refractory myasthenia gravis recently treated with chronic IVIG. Journal of Neuromuscular Diseases (2018) 5: S333-S334.doi.org/10.3233/JND-189001. 18. Jacob S, Murai H, Utsugisawa K, Nowak R, Wiendl H, Fujita K, et al. Response to eculizumab in myasthenia gravis patients recently treated with chronic IVIG. Muscle and Nerve (2018) 58: S102 19. Jacob S, Guptill JT, Meisel A, Fujita KP, Patra K, Howard JF. Eculizumab reduces exacerbation rates in patients with ACHR+ refractory generalized myasthenia gravis. Journal of Neuromuscular Diseases (2018) 5: S334.doi.org/10.3233/JND-189001. 20. Jacob S, Guptill J, Meisel A, Fujita K, Patra K, Howard J. Eculizumab reduces myasthenia gravis exacerbation rates. Muscle and Nerve (2018) 58: S98. 21. Howard JF, Utsugisawa K, Benatar M, Murai H, Barohn RJ, Sendra II, et al. Regain: A randomized, double-blind, placebocontrolled multi-center phase 3 study of the safety and efficacy of eculizumab in subjects with refractory generalized myasthenia gravis. Journal of Neuromuscular Diseases (2016) 3: S5-S6.doi.org/10.3233/JND-160001. 22. Howard J, Wang JJ, O'Brien F, Mantegazza R. Efficacy of eculizumab is maintained beyond 26 weeks in patients with ACHR+ refractory generalized myasthenia gravis. Muscle and Nerve (2017) 56: 649 23. Howard J, Wang JJ, O'Brien F, Mantegazza R. Efficacy of eculizumab on myasthenia gravis-activities of daily living and its respiratory, bulbar, limb and ocular domains in patients with ACHR+ refractory generalized myasthenia gravis. Muscle and Nerve (2017) 56: 649 24. Howard J, Wang J, O'Brien F, Mantegazza R. Efficacy of eculizumab is sustained over 52 weeks in patients with ACHR+ refractory generalized myasthenia gravis: Interim results from the open-label extension of regain. Muscle and Nerve (2017) 56: 657 25. Howard J, O'Brien F, Wang JJ, Mantegazza R. Dual responder analyses of both muscle strength and activities of daily living, eculizumab versus placebo, in refractory generalized myasthenia gravis (gMG) Patients: Results from the REGAIN Study. Neurology (2017) 89: e101-e102.doi.org/10.1212/WNL.0000000000004380. 26. Howard J, Karam C, Yountz M, O'Brien F, Mozaffar T. Long-term efficacy of eculizumab in refractory generalized yasthenia gravis: Responder analyses. Muscle and Nerve (2019) 60: S133 27. Howard J, Jacob S, Guptill J, Meisel A, Wiendl H, Murai H, et al. Relieving the burden of myasthenia gravis: Eculizumab reduces exacerbation, hospitalization and rescue therapy rates. Muscle and Nerve (2018) 58: S115 28. Frenay CL, Bourg V, Cohen M, Rosenthal A, Desnuelle C, Ticchioni M. Therapeutic target of memory b cells depletion helps to tailor administration frequency of rituximab in myasthenia gravis. Neurology (2016) 86 29. Bril V, Benatar M, Brock M, Greve B, Kiessling P, Woltering F, et al. Proof-of-concept and safety of the anti-FCRN antibody rozanolixizumab in patients with moderate-to-severe generalized myasthenia gravis (GMG): a phase 2a study. Neurology (2019) 92 30. Bernitsas E, Bao F, Sriwastava S, Memon A, Tselis A, Khan O. Long-term safety of rituximab in MS and other autoimmune disorders. Multiple Sclerosis (2014) 20: 447.doi.org/10.1177/1352458514547846. 31. Antozzi C, Guptill J, Bril V, Gamez J, Meuth SG, Blanco JLM, et al. VIVACITY-MG: A PHASE 2, MULTICENTER, RANDOMIZED, DOUBLE-BLIND, PLACEBO-CONTROLLED STUDY TO EVALUATE THE SAFETY, TOLERABILITY, EFFICACY, PHARMACOKINETICS, PHARMACODYNAMICS, AND IMMUNOGENICITY OF NIPOCALIMAB ADMINISTERED TO ADULTS WITH GENERALIZED MYASTHENIA GRAVIS. Clinical and Experimental Rheumatology (2023) 41: 498 32. Andersen H, Mantegazza R, Derosier F, Wang JJ, Zhang J, Howard J. Correlation of neuro-QOL with MG-ADL, QMG, and MG-QOL15 in assessing the spectrum of disease in patients with refractory generalised myasthenia gravis in the REGAIN study. European Journal of Neurology (2017) 24: 498-499.doi.org/10.1111/ene.13368. 33. Andersen H, Mantegazza R, Derosier F, Wang JJ, Zhang J, Howard J. Eculizumab improves fatigue in patients with refractory generalized myasthenia gravis in the REGAIN study. European Journal of Neurology (2017) 24: 498.doi.org/10.1111/ene.13368. 34. Macisaac J, Sidiqqi R, Jamula E, Li N, Baker S, Webert KE, et al. Anti-CD20 monoclonal antibody therapy for immune modulation across a range of autoimmune diseases. Blood (2017) 130 35. Topakian R, Zimprich F, Iglseder S, Embacher N, Guger M, Stieglbauer K, et al. High efficacy and safety of rituximab for myasthenia gravis: A nationwide study by austrian adult neurologists. Journal of Neuromuscular Diseases (2018) 5: S338-S339.doi.org/10.3233/JND-189001. 36. Suh J, Guidon A. Successful rituximab desensitization in two patients with myasthenia gravis. Neurology (2019) 92 37. Sayegh C, Arata M, DeMarco S, Duda P, Farzaneh-Far R, Ma Z, et al. MECHANISTIC AND PHARMACOLOGICAL DIFFERENTIATION OF ZILUCOPLAN, A MACROCYCLIC PEPTIDE INHIBITOR OF COMPLEMENT COMPONENT 5 (C5), FROM ANTI-C5 MONOCLONAL ANTIBODIES. Molecular Immunology (2019) 114: 447-448.doi.org/10.1016/j.molimm.2019.08.016. 38. González Toledo G, Pérez Pérez H, Hernández García M, Hernández Javier C, Crespo Rodríguez M, Lobato González M, et al. Treatment of myasthenia gravis: Experience of our hospital. Journal of Neuromuscular Diseases (2021) 8: S15.doi.org/10.3233/JND-219006. 39. Lee HA, Jang H, Kim Y, Jeong D, Lee J, Jung JA, et al. A Randomized, Double-Blind, Single-Dose Phase 1 Comparative Pharmacokinetic Study Comparing SB12 (Eculizumab Biosimilar) with Reference Eculizumab in Healthy Volunteers. Blood (2021) 138: 929‐.doi.org/10.1182/blood-2021-148197. 40. Kiessling P, Lledo-Garcia R, Watanabe S, Langdon G, Tran D, Bari M, et al. Safety, pharmacokinetics and pharmacodynamics of the FcRn inhibitor UCB7665: a phase I study. Journal of the peripheral nervous system (2017) 22: 315.doi.org/10.1111/jns.12225. 41. Howard J, Nowak R, Wolfe G, Benatar M, Duda P, MacDougall J, et al. Zilucoplan, a self-administered subcutaneous peptide inhibitor of complement component 5 (C5) for the treatment of generalized myasthenia gravis: phase 2 results. Muscle and Nerve (2019) 60: S129 42. Guptill J, Antozzi C, Bril V, Gamez J, Meuth SG, Blanco JLM, et al. Vivacity-MG: A phase 2, multicenter, randomized, double-blind, placebo-controlled study to evaluate the safety, tolerability, efficacy, pharmacokinetics, pharmacodynamics, and immunogenicity of nipocalimab administered to adults with generalized myasthenia gravis. Neurology (2021) 96 43. Guptill J, Antozzi C, Bril V, Gamez J, Meuth S, Muñoz Blanco J, et al. A phase 2, multicenter, randomized, doubleblind, placebo-controlled study to evaluate the safety, tolerability, efficacy, PK, and PD of nipocalimab (m281) in adults with generalized myasthenia gravis. Muscle and Nerve (2020) 62: S112-S113.doi.org/10.1007/s00259-020-04988-4. 44. Benatar M, Breiner A, Bril V, Nowak R, Dunn I, Jacobs A. Topline results of a phase 2 study of subcutaneous IMVT-1401 in patients with generalized myasthenia gravis. Neurology (2021) 96 45. Vissing J, Zhang J, Derosier F, Wang JJ, Howard J. Correlation of assessments of activities of daily living and muscle strength in refractory myasthenia gravis in the REGAIN study. European Journal of Neurology (2017) 24: 584-585.doi.org/10.1111/ene.13368. 46. Utsugisawa K, Howard JF, Wang JJ, O'Brien F, Murai H. Long-term efficacy of eculizumab on the MG-ADL scale, and its respiratory, bulbar, limb and ocular domains in patients with acetylcholine receptor-positive refractory generalized myasthenia gravis. Clinical and Experimental Neuroimmunology (2017) 8: 363-364 47. Suzuki S, Howard JF, Vu T, Mantegazza R, Kushlaf H, Wiendl H, et al. Ravulizumab efficacy in generalized myasthenia gravis by the time from diagnosis. Clinical and Experimental Neuroimmunology (2023) 14: 72-73.doi.org/10.1111/cen3.12738. 48. Mantegazza RE, Levine TD, Oreja-Guevara C, Carrillo-Infante C, Shang S, Kaprielian R, et al. No change in risk of infection among NMOSD and refractory gMG patients treated with eculizumab: Findings from two phase 3 studies and their extensions. European Journal of Neurology (2020) 27: 381-382 49. Vaikunthanathan T, Murakhovskaya I, Fattizzo B, Ebrahim T, Sweet K, Shu C. Designing ENERGY: A phase 2/3 study of Nipocalimab in warm autoimmune haemolytic Anaemia British. Journal of Haematology (2023) 201: 43.doi.org/10.1111/bjh.18720. 50. Hubbard J, Campbell K, Sivils K, Hoffman R, Lo K, Lee Y, et al. Design of a phase 2/multicenter/randomized/placebo-controlled/ double-blind study to assess the efficacy and safety of Nipocalimab in adults with primary Sjögren's syndrome. International Journal of Rheumatic Diseases (2023) 26: 207-208.doi.org/10.1111/1756-185X.14505. 51. Vu T, Meisel A, Mantegazza R, Annane D, Katsuno M, Aguzzi R, et al. Long-term efficacy and safety of ravulizumab in generalized myasthenia gravis: Phase 3 CHAMPION MG study open-label extension. Clinical and Experimental Neuroimmunology (2023) 14: 73.doi.org/10.1111/cen3.12738. 52. Sugimoto T, Druzdz A, Grosskreutz J, Habib AA, Mantegazza R, Sacconi S, et al. Safety and tolerability of rozanolixizumab in the randomized Phase 3 MycarinG study. Clinical and Experimental Neuroimmunology (2023) 14: 69.doi.org/10.1111/cen3.12738. 53. Ramchandren S, Sanga P, Burcklen M, Sun H. Vivacity MG Phase 3 Study: Clinical Trial of Nipocalimab Administered to Adults With Generalized Myasthenia Gravis. Neurology (2022) 93: S41.doi.org/10.1212/01.wnl.0000903616.08384.e6. 54. Howard J, Barohn R, Freimer M, Juel V, Mozaffar T, Mellion M, et al. Randomized, double-blind, placebo-controlled, crossover, multicenter, phase II study of eculizumab in patients with refractory generalized myasthenia gravis (GMG). Neurology (2012) 78.doi.org/10.1212/WNL.78.1. |
| **(3). Meta-analysis (n=7)**   1. Zhao C, Pu M, Chen D, Shi J, Li Z, Guo J, et al. Effectiveness and Safety of Rituximab for Refractory Myasthenia Gravis: A Systematic Review and Single-Arm Meta-Analysis. Front Neurol (2021) 12:736190. Epub 2021/11/02. doi: 10.3389/fneur.2021.736190. 2. Saccà F, Pane C, Espinosa PE, Sormani MP, Signori A. Efficacy of Innovative Therapies in Myasthenia Gravis: A Systematic Review, Meta-Analysis and Network Meta-Analysis. Eur J Neurol (2023). Epub 2023/05/19. doi: 10.1111/ene.15872. 3. Feng X, Song Z, Wu M, Liu Y, Luo S, Zhao C, et al. Efficacy and Safety of Immunotherapies in Refractory Myasthenia Gravis: A Systematic Review and Meta-Analysis. Front Neurol (2021) 12:725700. Epub 2021/12/21. doi: 10.3389/fneur.2021.725700. 4. Iorio R, Damato V, Alboini PE, Evoli A. Efficacy and Safety of Rituximab for Myasthenia Gravis: A Systematic Review and Meta-Analysis. J Neurol (2015) 262(5):1115-9. Epub 2014/10/14. doi: 10.1007/s00415-014-7532-3. 5. Wang L, Huan X, Xi JY, Wu H, Zhou L, Lu JH, et al. Immunosuppressive and Monoclonal Antibody Treatment for Myasthenia Gravis: A Network Meta-Analysis. CNS Neurosci Ther (2019) 25(5):647-58. Epub 2019/02/28. doi: 10.1111/cns.13110. 6. Song Z, Zhang J, Meng J, Jiang G, Yan Z, Yang Y, et al. Different Monoclonal Antibodies in Myasthenia Gravis: A Bayesian Network Meta-Analysis. Front Pharmacol (2021) 12:790834. Epub 2022/02/05. doi: 10.3389/fphar.2021.790834. 7. Li T, Zhang GQ, Li Y, Dong SA, Wang N, Yi M, et al. Efficacy and Safety of Different Dosages of Rituximab for Refractory Generalized Achr Myasthenia Gravis: A Meta-Analysis. J Clin Neurosci (2021) 85:6-12. Epub 2021/02/15. doi: 10.1016/j.jocn.2020.11.043. |
| **(4). Review (n=95）**   1. Gable KL, Guptill JT. Antagonism of the Neonatal Fc Receptor as an Emerging Treatment for Myasthenia Gravis. Front Immunol (2019) 10:3052. Epub 2020/01/31. doi: 10.3389/fimmu.2019.03052. 2. Uzawa A, Utsugisawa K. Biological Therapies for Myasthenia Gravis. Expert Opin Biol Ther (2023) 23(3):253-60. Epub 2023/03/01. doi: 10.1080/14712598.2023.2184257. 3. Miyamoto K. [C5: Eculizumab]. Brain Nerve (2014) 66(10):1191-9. Epub 2014/10/10. doi: 10.11477/mf.1416200008. 4. Li Y, Yi JS, Howard JF, Jr., Chopra M, Russo MA, Guptill JT. Cellular Changes in Eculizumab Early Responders with Generalized Myasthenia Gravis. Clin Immunol (2021) 231:108830. Epub 2021/08/28. doi: 10.1016/j.clim.2021.108830. 5. Xiao H, Wu K, Liang X, Li R, Lai KP. Clinical Efficacy and Safety of Eculizumab for Treating Myasthenia Gravis. Front Immunol (2021) 12:715036. Epub 2021/08/31. doi: 10.3389/fimmu.2021.715036. 6. Ahlberg R, Yi Q, Pirskanen R, Matell G, Swerup C, Rieber P, et al. Clinical Improvement of Myasthenia Gravis by Treatment with a Chimeric Anti-Cd4 Monoclonal Antibody. Ann N Y Acad Sci (1993) 681:552-5. Epub 1993/06/21. doi: 10.1111/j.1749-6632.1993.tb22938.x. 7. Mantegazza R, Vanoli F, Frangiamore R, Cavalcante P. Complement Inhibition for the Treatment of Myasthenia Gravis. Immunotargets Ther (2020) 9:317-31. Epub 2020/12/29. doi: 10.2147/itt.S261414. 8. Albazli K, Kaminski HJ, Howard JF, Jr. Complement Inhibitor Therapy for Myasthenia Gravis. Front Immunol (2020) 11:917. Epub 2020/06/26. doi: 10.3389/fimmu.2020.00917. 9. Baek WS, Bashey A, Sheean GL. Complete Remission Induced by Rituximab in Refractory, Seronegative, Muscle-Specific, Kinase-Positive Myasthenia Gravis. J Neurol Neurosurg Psychiatry (2007) 78(7):771. Epub 2007/06/19. doi: 10.1136/jnnp.2006.093567. 10. Evoli A, Damato V. Conventional and Emerging Treatments and Controversies in Myasthenia Gravis. Expert Rev Neurother (2023) 23(5):445-56. Epub 2023/04/27. doi: 10.1080/14737175.2023.2207739. 11. Mantegazza R, Bonanno S, Camera G, Antozzi C. Current and Emerging Therapies for the Treatment of Myasthenia Gravis. Neuropsychiatric Disease and Treatment (2011) 7(1):151-60. doi: 10.2147/NDT.S8915. 12. Barnett C, Tabasinejad R, Bril V. Current Pharmacotherapeutic Options for Myasthenia Gravis. Expert Opin Pharmacother (2019) 20(18):2295-303. Epub 2019/11/02. doi: 10.1080/14656566.2019.1682548. 13. Alhaidar MK, Abumurad S, Soliven B, Rezania K. Current Treatment of Myasthenia Gravis. Journal of Clinical Medicine (2022) 11(6). doi: 10.3390/jcm11061597. 14. Guptill JT, Soni M, Meriggioli MN. Current Treatment, Emerging Translational Therapies, and New Therapeutic Targets for Autoimmune Myasthenia Gravis. Neurotherapeutics (2016) 13(1):118-31. Epub 2015/10/30. doi: 10.1007/s13311-015-0398-y. 15. Mantegazza R, Cavalcante P. Diagnosis and Treatment of Myasthenia Gravis. Curr Opin Rheumatol (2019) 31(6):623-33. Epub 2019/08/07. doi: 10.1097/bor.0000000000000647. 16. Lekova E, Zelek WM, Gower D, Spitzfaden C, Osuch IH, John-Morris E, et al. Discovery of Functionally Distinct Anti-C7 Monoclonal Antibodies and Stratification of Anti-Nicotinic Achr Positive Myasthenia Gravis Patients. Front Immunol (2022) 13:968206. Epub 2022/09/24. doi: 10.3389/fimmu.2022.968206. 17. Caetano R, Cordeiro Dias Villela Correa M, Villardi P, Almeida Rodrigues PH, Garcia Serpa Osorio-de-Castro C. Dynamics of Patents, Orphan Drug Designation, Licensing, and Revenues from Drugs for Rare Diseases: The Market Expansion of Eculizumab. PLoS One (2021) 16(3):e0247853. Epub 2021/03/06. doi: 10.1371/journal.pone.0247853. 18. Patriquin CJ, Kuo KHM. Eculizumab and Beyond: The Past, Present, and Future of Complement Therapeutics. Transfus Med Rev (2019) 33(4):256-65. Epub 2019/11/11. doi: 10.1016/j.tmrv.2019.09.004. 19. Vélez-Santamaría V, Nedkova V, Díez L, Homedes C, Alberti MA, Casasnovas C. Eculizumab as a Promising Treatment in Thymoma-Associated Myasthenia Gravis. Ther Adv Neurol Disord (2020) 13:1756286420932035. Epub 2020/07/14. doi: 10.1177/1756286420932035. 20. Yeo CJJ, Pleitez MY. Eculizumab in Refractory Myasthenic Crisis. Muscle Nerve (2018) 58(2):E13-e5. Epub 2018/03/11. doi: 10.1002/mus.26124. 21. Monteleone JPR, Gao X, Kleijn HJ, Bellanti F, Pelto R. Eculizumab Pharmacokinetics and Pharmacodynamics in Patients with Generalized Myasthenia Gravis. Frontiers in neurology (2021) 12. doi: 10.3389/fneur.2021.696385. 22. Jiao L, Li H, Guo S. Eculizumab Treatment for Myasthenia Gravis Subgroups: 2021 Update. J Neuroimmunol (2022) 362:577767. Epub 2021/11/26. doi: 10.1016/j.jneuroim.2021.577767. 23. Uzawa A, Kuwabara S. [Eculizumab Treatment for Refractory Generalized Myasthenia Gravis]. Brain Nerve (2019) 71(6):565-70. Epub 2019/06/07. doi: 10.11477/mf.1416201317. 24. Edmundson C, Guidon AC. Eculizumab: A Complementary Addition to Existing Long-Term Therapies for Myasthenia Gravis. Muscle Nerve (2019) 60(1):7-9. Epub 2019/05/11. doi: 10.1002/mus.26512. 25. Dhillon S. Eculizumab: A Review in Generalized Myasthenia Gravis. Drugs (2018) 78(3):367-76. Epub 2018/02/13. doi: 10.1007/s40265-018-0875-9. 26. Gilhus NE. Eculizumab: A Treatment Option for Myasthenia Gravis? Lancet Neurol (2017) 16(12):947-8. Epub 2017/10/27. doi: 10.1016/s1474-4422(17)30363-0. 27. Efgartigimod Alfa (Vyvgart) for Myasthenia Gravis. Med Lett Drugs Ther (2022) 64(1648):62-3. Epub 2022/04/19. 28. Heo YA. Efgartigimod Alfa in Generalised Myasthenia Gravis: A Profile of Its Use. CNS Drugs (2023) 37(5):467-73. Epub 2023/04/01. doi: 10.1007/s40263-023-01000-z. 29. Suzuki S, Uzawa A, Murai H. Efgartigimod for Generalized Myasthenia Gravis with or without Anti-Acetylcholine Receptor Antibodies: A Worldwide and Japanese Perspective. Expert Rev Clin Immunol (2022) 18(12):1207-15. Epub 2022/10/14. doi: 10.1080/1744666x.2022.2136167. 30. Heo YA. Efgartigimod: First Approval. Drugs (2022) 82(3):341-8. Epub 2022/02/19. doi: 10.1007/s40265-022-01678-3. 31. Menon D, Urra Pincheira A, Bril V. Emerging Drugs for the Treatment of Myasthenia Gravis. Expert Opin Emerg Drugs (2021) 26(3):259-70. Epub 2021/07/07. doi: 10.1080/14728214.2021.1952982. 32. Evoli A. The Emerging Treatment Landscape for Myasthenia Gravis. Journal of Neuromuscular Diseases (2022) 9:S23. doi: 10.3233/JND-229001. 33. Heidenreich F, Vincent A, Roberts A, Newsom-Davis J. Epitopes on Human Acetylcholine Receptor Defined by Monoclonal Antibodies and Myasthenia Gravis Sera. Autoimmunity (1988) 1(4):285-97. Epub 1988/01/01. doi: 10.3109/08916938809010682. 34. Keller CW, Pawlitzki M, Wiendl H, Lünemann JD. Fc-Receptor Targeted Therapies for the Treatment of Myasthenia Gravis. Int J Mol Sci (2021) 22(11). Epub 2021/06/03. doi: 10.3390/ijms22115755. 35. Psaridi-Linardaki L, Mamalaki A, Tzartos SJ. Future Therapeutic Strategies in Autoimmune Myasthenia Gravis. Ann N Y Acad Sci (2003) 998:539-48. Epub 2003/11/01. doi: 10.1196/annals.1254.071. 36. Bond A, Corlett L, Nagvekar N, Jacobson L, Pantic N, Beeson D, et al. Heterogeneity and Immunotherapy of Specific T-Cells in Myasthenia Gravis. Biochem Soc Trans (1997) 25(2):665-70. Epub 1997/05/01. doi: 10.1042/bst0250665. 37. Kushlaf H, Li Y. How Does Rituximab Fit in the Treatment Algorithm of Myasthenia Gravis? Muscle Nerve (2021) 64(6):635-6. Epub 2021/09/22. doi: 10.1002/mus.27421. 38. Lefvert AK, Holm G. Idiotypic Network in Myasthenia Gravis Demonstrated by Human Monoclonal B-Cell Lines. Scand J Immunol (1987) 26(5):573-8. Epub 1987/11/01. doi: 10.1111/j.1365-3083.1987.tb02291.x. 39. Wolfe GI, Ward ES, de Haard H, Ulrichts P, Mozaffar T, Pasnoor M, et al. Igg Regulation through Fcrn Blocking: A Novel Mechanism for the Treatment of Myasthenia Gravis. J Neurol Sci (2021) 430:118074. Epub 2021/09/27. doi: 10.1016/j.jns.2021.118074. 40. Biesecker G, Koffler D. Immunology of Myasthenia Gravis. Hum Pathol (1983) 14(5):419-23. Epub 1983/05/01. doi: 10.1016/s0046-8177(83)80286-x. 41. Zhang C, Bu B, Yang H, Wang L, Liu W, Duan RS, et al. Immunotherapy Choice and Maintenance for Generalized Myasthenia Gravis in China. CNS Neurosci Ther (2020) 26(12):1241-54. Epub 2020/10/27. doi: 10.1111/cns.13468. 42. Maselli RA, Jow B, Richman DP, Nelson DJ. In Vitro Blockade of Neuromuscular Transmission by Monoclonal Anti-Acetylcholine Receptor Antibodies. Ann N Y Acad Sci (1988) 540:523-4. Epub 1988/01/01. doi: 10.1111/j.1749-6632.1988.tb27158.x. 43. Huda R. Inflammation and Autoimmune Myasthenia Gravis. Front Immunol (2023) 14:1110499. Epub 2023/02/17. doi: 10.3389/fimmu.2023.1110499. 44. Narayanaswami P, Sanders DB, Wolfe G, Benatar M, Cea G, Evoli A, et al. International Consensus Guidance for Management of Myasthenia Gravis: 2020 Update. Neurology (2021) 96(3):114-22. Epub 2020/11/05. doi: 10.1212/wnl.0000000000011124. 45. Díaz-Manera J, Martínez-Hernández E, Querol L, Klooster R, Rojas-García R, Suárez-Calvet X, et al. Long-Lasting Treatment Effect of Rituximab in Musk Myasthenia. Neurology (2012) 78(3):189-93. Epub 2012/01/06. doi: 10.1212/WNL.0b013e3182407982. 46. Muto K, Matsui N, Unai Y, Sakai W, Haji S, Udaka K, et al. Memory B Cell Resurgence Requires Repeated Rituximab in Myasthenia Gravis. Neuromuscul Disord (2017) 27(10):918-22. Epub 2017/07/12. doi: 10.1016/j.nmd.2017.06.012. 47. Klotz L, Wiendl H. Monoclonal Antibodies in Neuroinflammatory Diseases. Expert Opin Biol Ther (2013) 13(6):831-46. Epub 2013/03/26. doi: 10.1517/14712598.2013.767329. 48. Alabbad S, AlGaeed M, Sikorski P, Kaminski HJ. Monoclonal Antibody-Based Therapies for Myasthenia Gravis. BioDrugs (2020) 34(5):557-66. Epub 2020/09/12. doi: 10.1007/s40259-020-00443-w. 49. Cai XJ, Li ZW, Xi JY, Song HZ, Liu J, Zhu WH, et al. Myasthenia Gravis and Specific Immunotherapy: Monoclonal Antibodies. Ann N Y Acad Sci (2019) 1452(1):18-33. Epub 2019/08/09. doi: 10.1111/nyas.14195. 50. Miñón-Fernández B, Losada-Domingo JM, Sánchez-Horvath MT, Bárcena-Llona J. [Myasthenia Gravis Associated with Nivolumab]. Rev Neurol (2020) 70(2):72-3. Epub 2020/01/14. doi: 10.33588/rn.7002.2019153. 51. Zhu J, Li Y. Myasthenia Gravis Exacerbation Associated with Pembrolizumab. Muscle Nerve (2016) 54(3):506-7. Epub 2016/01/24. doi: 10.1002/mus.25055. 52. Markéta K, Kamil M, Kamil K. [Myasthenia Gravis--Current Treatment Standards and Emerging Drugs]. Ceska Slov Farm (2011) 60(2):47-53. Epub 2011/06/10. 53. Sieb JP. Myasthenia Gravis: Emerging New Therapy Options. Curr Opin Pharmacol (2005) 5(3):303-7. Epub 2005/05/24. doi: 10.1016/j.coph.2005.01.010. 54. Whiting PJ, Vincent A, Newsom-Davis J. Myasthenia Gravis: Monoclonal Antihuman Acetylcholine Receptor Antibodies Used to Analyze Antibody Specificities and Responses to Treatment. Neurology (1986) 36(5):612-7. Epub 1986/05/01. doi: 10.1212/wnl.36.5.612. 55. Lewis RA. Myasthenia Gravis: New Therapeutic Approaches Based on Pathophysiology. J Neurol Sci (2013) 333(1-2):93-8. Epub 2013/08/14. doi: 10.1016/j.jns.2013.06.018. 56. Souto EB, Lima B, Campos JR, Martins-Gomes C, Souto SB, Silva AM. Myasthenia Gravis: State of the Art and New Therapeutic Strategies. J Neuroimmunol (2019) 337:577080. Epub 2019/11/02. doi: 10.1016/j.jneuroim.2019.577080. 57. Howard JF, Jr. Myasthenia Gravis: The Role of Complement at the Neuromuscular Junction. Ann N Y Acad Sci (2018) 1412(1):113-28. Epub 2017/12/22. doi: 10.1111/nyas.13522. 58. Alfaro A, Peñarrocha M. [Myasthenia Gravis]. Med Clin (Barc) (1984) 82(10):462-9. Epub 1984/03/17. 59. Huda R. New Approaches to Targeting B Cells for Myasthenia Gravis Therapy. Front Immunol (2020) 11:240. Epub 2020/03/11. doi: 10.3389/fimmu.2020.00240. 60. Evoli A. New Therapies for Myastenia Gravis: Just for Refractory Disease. Journal of Neuromuscular Diseases (2014) 1:S57-S8. doi: 10.3233/JND-149001. 61. Dalakas MC. Novel Future Therapeutic Options in Myasthenia Gravis. Autoimmun Rev (2013) 12(9):936-41. Epub 2013/03/30. doi: 10.1016/j.autrev.2013.03.006. 62. Manu P, Rogozea LM, Roman-Filip C. Pharmacological Management of Myasthenia Gravis: A Century of Expert Opinions in Cecil Textbook of Medicine. Am J Ther (2021) 28(6):e631-e7. Epub 2021/11/11. doi: 10.1097/mjt.0000000000001454. 63. Wijnsma KL, Ter Heine R, Moes D, Langemeijer S, Schols SEM, Volokhina EB, et al. Pharmacology, Pharmacokinetics and Pharmacodynamics of Eculizumab, and Possibilities for an Individualized Approach to Eculizumab. Clin Pharmacokinet (2019) 58(7):859-74. Epub 2019/02/14. doi: 10.1007/s40262-019-00742-8. 64. Menon D, Bril V. Pharmacotherapy of Generalized Myasthenia Gravis with Special Emphasis on Newer Biologicals. Drugs (2022) 82(8):865-87. Epub 2022/06/01. doi: 10.1007/s40265-022-01726-y. 65. Waheed W, Newman E, Aboukhatwa M, Moin M, Tandan R. Practical Management for Use of Eculizumab in the Treatment of Severe, Refractory, Non-Thymomatous, Achr + Generalized Myasthenia Gravis: A Systematic Review. Ther Clin Risk Manag (2022) 18:699-719. Epub 2022/07/21. doi: 10.2147/tcrm.S266031. 66. Dalakas MC. Progress in the Therapy of Myasthenia Gravis: Getting Closer to Effective Targeted Immunotherapies. Curr Opin Neurol (2020) 33(5):545-52. Epub 2020/08/25. doi: 10.1097/wco.0000000000000858. 67. Konno S. [Proposal for Rituximab Treatment in Patients with Myasthenia Gravis]. Rinsho Shinkeigaku (2013) 53(11):1312-4. Epub 2013/12/03. doi: 10.5692/clinicalneurol.53.1312. 68. Finsterer J, Scorza FA, Scorza CA. Prospective Studies on the Efficacy of Rituximab for Myasthenia Gravis Are Warranted. Eur J Neurol (2020) 27(12):e95. Epub 2020/08/05. doi: 10.1111/ene.14461. 69. Lagoumintzis G, Zisimopoulou P, Kordas G, Lazaridis K, Poulas K, Tzartos SJ. Recent Approaches to the Development of Antigen-Specific Immunotherapies for Myasthenia Gravis. Autoimmunity (2010) 43(5-6):436-45. Epub 2010/03/02. doi: 10.3109/08916930903518099. 70. Ueno S. [Receptor Function and Monoclonal Antibody]. Nihon Rinsho (1997) 55 Suppl 2:457-62. Epub 1997/04/01. 71. Sudulagunta SR, Sepehrar M, Sodalagunta MB, Settikere Nataraju A, Bangalore Raja SK, Sathyanarayana D, et al. Refractory Myasthenia Gravis - Clinical Profile, Comorbidities and Response to Rituximab. Ger Med Sci (2016) 14:Doc12. Epub 2016/10/30. doi: 10.3205/000239. 72. Mitsune A, Yanagisawa S, Fukuhara T, Miyauchi E, Morita M, Ono M, et al. Relapsed Myasthenia Gravis after Nivolumab Treatment. Intern Med (2018) 57(13):1893-7. Epub 2018/02/13. doi: 10.2169/internalmedicine.9153-17. 73. Bril V, Silvestri NJ, Barnett-Tapia C. A Review of Disease Mechanisms and Current and Emerging Treatment Options for Generalized Myasthenia Gravis. Prim Care Companion CNS Disord (2022) 24(3). Epub 2022/05/28. doi: 10.4088/pcc.Ar21018wc2c. 74. Zingariello CD, Elder ME, Kang PB. Rituximab as Adjunct Maintenance Therapy for Refractory Juvenile Myasthenia Gravis. Pediatr Neurol (2020) 111:40-3. Epub 2020/09/22. doi: 10.1016/j.pediatrneurol.2020.07.002. 75. Hehir MK, Li Y. Rituximab for Late Onset Myasthenia Gravis: Evidence Mounting but Deliberations Continue. Muscle Nerve (2020) 62(3):297-9. Epub 2020/06/13. doi: 10.1002/mus.26998. 76. Di Stefano V, Lupica A, Rispoli MG, Di Muzio A, Brighina F, Rodolico C. Rituximab in Achr Subtype of Myasthenia Gravis: Systematic Review. J Neurol Neurosurg Psychiatry (2020) 91(4):392-5. Epub 2020/02/27. doi: 10.1136/jnnp-2019-322606. 77. Marino M, Bartoccioni E, Alboini PE, Evoli A. Rituximab in Myasthenia Gravis: A "to Be or Not to Be" Inhibitor of T Cell Function. Ann N Y Acad Sci (2018) 1413(1):41-8. Epub 2018/01/26. doi: 10.1111/nyas.13562. 78. Caballero-Ávila M, Álvarez-Velasco R, Moga E, Rojas-Garcia R, Turon-Sans J, Querol L, et al. Rituximab in Myasthenia Gravis: Efficacy, Associated Infections and Risk of Induced Hypogammaglobulinemia. Neuromuscul Disord (2022) 32(8):664-71. Epub 2022/07/11. doi: 10.1016/j.nmd.2022.06.006. 79. Chuquilin M, Barohn R. Rituximab in Newly Diagnosed Generalized Myasthenia Gravis: A New Treatment Paradigm? JAMA Neurol (2022) 79(11):1100-2. Epub 2022/09/20. doi: 10.1001/jamaneurol.2022.2311. 80. Koul R, Al Futaisi A, Abdwani R. Rituximab in Severe Seronegative Juvenile Myasthenia Gravis: Review of the Literature. Pediatr Neurol (2012) 47(3):209-12. Epub 2012/08/14. doi: 10.1016/j.pediatrneurol.2012.05.017. 81. Crusz SM, Radunovic A, Shepherd S, Shah S, Newey V, Phillips M, et al. Rituximab in the Treatment of Pembrolizumab-Induced Myasthenia Gravis. Eur J Cancer (2018) 102:49-51. Epub 2018/08/24. doi: 10.1016/j.ejca.2018.07.125. 82. Silvestri NJ, Wolfe GI. Rituximab in Treatment-Refractory Myasthenia Gravis. JAMA Neurol (2017) 74(1):21-3. Epub 2016/11/29. doi: 10.1001/jamaneurol.2016.4367. 83. Tandan R, Hehir MK, 2nd, Waheed W, Howard DB. Rituximab Treatment of Myasthenia Gravis: A Systematic Review. Muscle Nerve (2017) 56(2):185-96. Epub 2017/02/07. doi: 10.1002/mus.25597. 84. Pescovitz MD. Rituximab, an Anti-Cd20 Monoclonal Antibody: History and Mechanism of Action. Am J Transplant (2006) 6(5 Pt 1):859-66. Epub 2006/04/14. doi: 10.1111/j.1600-6143.2006.01288.x. 85. Dalakas MC. Role of Complement, Anti-Complement Therapeutics, and Other Targeted Immunotherapies in Myasthenia Gravis. Expert Rev Clin Immunol (2022) 18(7):691-701. Epub 2022/06/23. doi: 10.1080/1744666x.2022.2082946. 86. Uzawa A, Ozawa Y, Yasuda M, Kuwabara S. Severe Worsening of Myasthenic Symptoms after the Eculizumab Discontinuation. J Neuroimmunol (2020) 349:577424. Epub 2020/10/18. doi: 10.1016/j.jneuroim.2020.577424. 87. Jiang R, Fichtner ML, Hoehn KB, Pham MC, Stathopoulos P, Nowak RJ, et al. Single-Cell Repertoire Tracing Identifies Rituximab-Resistant B Cells During Myasthenia Gravis Relapses. JCI Insight (2020) 5(14). Epub 2020/06/24. doi: 10.1172/jci.insight.136471. 88. Marchiori PE, Duarte AJ, Birolli MI, Figueiredo CA, Scaff M, De Assis JL. [Study of Circulating Lymphocytes by Monoclonal Antibodies in Myasthenia Gravis]. Arq Neuropsiquiatr (1988) 46(3):248-53. Epub 1988/09/01. doi: 10.1590/s0004-282x1988000300003. 89. Dreyfus DH, Na CR, Randolph CC, Kearney D, Price C, Podell D. Successful Rituximab B Lymphocyte Depletion Therapy for Angioedema Due to Acquired C1 Inhibitor Protein Deficiency: Association with Reduced C1 Inhibitor Protein Autoantibody Titers. Isr Med Assoc J (2014) 16(5):315-6. Epub 2014/07/02. 90. Oyama M, Okada K, Masuda M, Shimizu Y, Yokoyama K, Uzawa A, et al. Suitable Indications of Eculizumab for Patients with Refractory Generalized Myasthenia Gravis. Ther Adv Neurol Disord (2020) 13:1756286420904207. Epub 2020/03/28. doi: 10.1177/1756286420904207. 91. Beecher G, Putko BN, Wagner AN, Siddiqi ZA. Therapies Directed against B-Cells and Downstream Effectors in Generalized Autoimmune Myasthenia Gravis: Current Status. Drugs (2019) 79(4):353-64. Epub 2019/02/15. doi: 10.1007/s40265-019-1065-0. 92. Pénisson-Besnier I. Treatment of Autoimmune Myasthenia. Revue Neurologique (2010) 166(4):400-5. doi: 10.1016/j.neurol.2009.09.006. 93. Bodkin C, Pascuzzi RM. Update in the Management of Myasthenia Gravis and Lambert-Eaton Myasthenic Syndrome. Neurol Clin (2021) 39(1):133-46. Epub 2020/11/24. doi: 10.1016/j.ncl.2020.09.007. 94. Habib AA, Ahmadi Jazi G, Mozaffar T. Update on Immune-Mediated Therapies for Myasthenia Gravis. Muscle Nerve (2020) 62(5):579-92. Epub 2020/05/29. doi: 10.1002/mus.26919. 95. Lindstrom J, Tzartos S, Gullick W, Hochschwender S, Swanson L, Sargent P, et al. Use of Monoclonal Antibodies to Study Acetylcholine Receptors from Electric Organs, Muscle, and Brain and the Autoimmune Response to Receptor in Myasthenia Gravis. Cold Spring Harb Symp Quant Biol (1983) 48 Pt 1:89-99. Epub 1983/01/01. doi: 10.1101/sqb.1983.048.01.012. |
| **(5). Observational studies (n=41)**   1. Katyal N, Narula N, Govindarajan R. Clinical Experience with Eculizumab in Treatment-Refractory Acetylcholine Receptor Antibody-Positive Generalized Myasthenia Gravis. J Neuromuscul Dis (2021) 8(2):287-94. Epub 2020/12/17. doi: 10.3233/jnd-200584. 2. Brauner S, Eriksson-Dufva A, Hietala MA, Frisell T, Press R, Piehl F. Comparison between Rituximab Treatment for New-Onset Generalized Myasthenia Gravis and Refractory Generalized Myasthenia Gravis. JAMA Neurol (2020) 77(8):974-81. Epub 2020/05/05. doi: 10.1001/jamaneurol.2020.0851. 3. Litchman T, Roy B, Kumar A, Sharma A, Njike V, Nowak RJ. Differential Response to Rituximab in Anti-Achr and Anti-Musk Positive Myasthenia Gravis Patients: A Single-Center Retrospective Study. J Neurol Sci (2020) 411:116690. Epub 2020/02/07. doi: 10.1016/j.jns.2020.116690. 4. Nelke C, Schroeter CB, Stascheit F, Pawlitzki M, Regner-Nelke L, Huntemann N, et al. Eculizumab Versus Rituximab in Generalised Myasthenia Gravis. J Neurol Neurosurg Psychiatry (2022) 93(5):548-54. Epub 2022/03/06. doi: 10.1136/jnnp-2021-328665. 5. Göl MF, Kara F, Boz M, Mutlu A, Karakullukçu S, Boz C. [Effects of Rituximab on Prognosis in Myasthenia Gravis: A Single-Center Experience from Turkey]. Ideggyogy Sz (2022) 75(9-10):351-9. Epub 2022/10/12. doi: 10.18071/isz.75.0351. 6. Dos Santos A, Noury JB, Genestet S, Nadaj-Pakleza A, Cassereau J, Baron C, et al. Efficacy and Safety of Rituximab in Myasthenia Gravis: A French Multicentre Real-Life Study. Eur J Neurol (2020) 27(11):2277-85. Epub 2020/06/12. doi: 10.1111/ene.14391. 7. Topakian R, Zimprich F, Iglseder S, Embacher N, Guger M, Stieglbauer K, et al. High Efficacy of Rituximab for Myasthenia Gravis: A Comprehensive Nationwide Study in Austria. J Neurol (2019) 266(3):699-706. Epub 2019/01/17. doi: 10.1007/s00415-019-09191-6. 8. Kerkeni S, Marotte H, Miossec P. Improvement with Rituximab in a Patient with Both Rheumatoid Arthritis and Myasthenia Gravis. Muscle Nerve (2008) 38(4):1343-5. Epub 2008/09/26. doi: 10.1002/mus.21047. 9. Du Y, Li C, Hao YF, Zhao C, Yan Q, Yao D, et al. Individualized Regimen of Low-Dose Rituximab Monotherapy for New-Onset Achr-Positive Generalized Myasthenia Gravis. J Neurol (2022) 269(8):4229-40. Epub 2022/03/05. doi: 10.1007/s00415-022-11048-4. 10. Marino M, Basile U, Spagni G, Napodano C, Iorio R, Gulli F, et al. Long-Lasting Rituximab-Induced Reduction of Specific-but Not Total-Igg4 in Musk-Positive Myasthenia Gravis. Front Immunol (2020) 11:613. Epub 2020/05/21. doi: 10.3389/fimmu.2020.00613. 11. Castiglione JI, Rivero AD, Barroso F, Brand P, Lautre A, Kohler AA. Long-Term Remission with Low-Dose Rituximab in Myasthenia Gravis: A Retrospective Study. J Clin Neuromuscul Dis (2022) 24(1):18-25. Epub 2022/08/26. doi: 10.1097/cnd.0000000000000420. 12. Lu J, Zhong H, Jing S, Wang L, Xi J, Lu J, et al. Low-Dose Rituximab Every 6 Months for the Treatment of Acetylcholine Receptor-Positive Refractory Generalized Myasthenia Gravis. Muscle Nerve (2020) 61(3):311-5. Epub 2019/12/27. doi: 10.1002/mus.26790. 13. Juel VC, Sanders DB, Hobson-Webb LD, Massey JM, Guptill JT, O'Brien F, et al. Marked Clinical and Jitter Improvement after Eculizumab in Refractory Myasthenia. Muscle Nerve (2017) 56(3):E16-e8. Epub 2017/02/19. doi: 10.1002/mus.25620. 14. Lau AY, Chan AY, Mok VC. Refractory Bulbar and Respiratory Dysfunction in a Young Chinese Woman with Seronegative, Muscle-Specific Tyrosine Kinase Antibody-Positive Myasthenia Gravis: Response to Cyclophosphamide and Rituximab Treatment. Hong Kong Med J (2011) 17(1):77-9. Epub 2011/02/02. 15. Afanasiev V, Demeret S, Bolgert F, Eymard B, Laforêt P, Benveniste O. Resistant Myasthenia Gravis and Rituximab: A Monocentric Retrospective Study of 28 Patients. Neuromuscul Disord (2017) 27(3):251-8. Epub 2017/01/14. doi: 10.1016/j.nmd.2016.12.004. 16. Wang J, Wu X, Deng H, Liu Y, Liu H, Fan X, et al. Response to Rituximab in an Anti-Muscle-Specific Receptor Tyrosine Kinase- and Anti-Acetylcholine Recepto-Double-Positive Myasthenia Gravis Patient Concomitant with Follicular Dendritic Cell Sarcoma. Neurosciences (Riyadh) (2016) 21(1):77-8. Epub 2016/01/29. doi: 10.17712/nsj.2016.1.20150483. 17. Jing S, Song Y, Song J, Pang S, Quan C, Zhou L, et al. Responsiveness to Low-Dose Rituximab in Refractory Generalized Myasthenia Gravis. J Neuroimmunol (2017) 311:14-21. Epub 2017/08/10. doi: 10.1016/j.jneuroim.2017.05.021. 18. Datta S, Singh S, Govindarajan R. Retrospective Analysis of Eculizumab in Patients with Acetylcholine Receptor Antibody-Negative Myasthenia Gravis: A Case Series. J Neuromuscul Dis (2020) 7(3):269-77. Epub 2020/05/24. doi: 10.3233/jnd-190464. 19. Doughty CT, Suh J, David WS, Amato AA, Guidon AC. Retrospective Analysis of Safety and Outcomes of Rituximab for Myasthenia Gravis in Patients ≥65 years Old. Muscle Nerve (2021) 64(6):651-6. Epub 2021/08/12. doi: 10.1002/mus.27393. 20. Hehir MK, Hobson-Webb LD, Benatar M, Barnett C, Silvestri NJ, Howard JF, Jr., et al. Rituximab as Treatment for Anti-Musk Myasthenia Gravis: Multicenter Blinded Prospective Review. Neurology (2017) 89(10):1069-77. Epub 2017/08/13. doi: 10.1212/wnl.0000000000004341. 21. Lindberg C, Bokarewa M. Rituximab for Severe Myasthenia Gravis--Experience from Five Patients. Acta Neurol Scand (2010) 122(4):225-8. Epub 2010/03/05. doi: 10.1111/j.1600-0404.2010.01345.x. 22. Martínez-Monte E, Gascón-Giménez F, Domínguez-Morán JA, Láinez-Andres JM. [Rituximab for the Treatment of Generalised Myasthenia Gravis: Experience in Clinical Practice]. Rev Neurol (2021) 73(12):416-20. Epub 2021/12/09. doi: 10.33588/rn.7312.2021166. 23. Collongues N, Casez O, Lacour A, Tranchant C, Vermersch P, de Seze J, et al. Rituximab in Refractory and Non-Refractory Myasthenia: A Retrospective Multicenter Study. Muscle Nerve (2012) 46(5):687-91. Epub 2012/09/04. doi: 10.1002/mus.23412. 24. Govindarajan R, Iyadurai SJ, Connolly A, Zaidman C. Selective Response to Rituximab in a Young Child with Musk-Associated Myasthenia Gravis. Neuromuscul Disord (2015) 25(8):651-2. Epub 2015/05/23. doi: 10.1016/j.nmd.2015.03.014. 25. Zhou Y, Yan C, Gu X, Zhou L, Lu J, Zhu W, et al. Short-Term Effect of Low-Dose Rituximab on Myasthenia Gravis with Muscle-Specific Tyrosine Kinase Antibody. Muscle Nerve (2021) 63(6):824-30. Epub 2021/03/22. doi: 10.1002/mus.27233. 26. Heckmann JM. A Single Low-Dose Rituximab Infusion in Severe Chronic Refractory Myasthenia Gravis in Resource-Limited Settings. J Neurol Sci (2022) 442:120394. Epub 2022/09/04. doi: 10.1016/j.jns.2022.120394. 27. Euctr ES. A Study to Examine the Efficacy and Safety of Pozelimab and Cemdisiran Combination Therapy in Patients with Symptomatic Generalized Myasthenia Gravis. https://trialsearchwhoint/Trial2aspx?TrialID=EUCTR2020-003272-41-ES (2021). 28. Lebrun C, Bourg V, Tieulie N, Thomas P. Successful Treatment of Refractory Generalized Myasthenia Gravis with Rituximab. Eur J Neurol (2009) 16(2):246-50. Epub 2009/01/17. doi: 10.1111/j.1468-1331.2008.02399.x. 29. Estrin A, Ting A, Shah R, Lee E. Treatment Patterns and Healthcare Resource Utilization among Patients with Myasthenia Gravis Starting Acetylcholinesterase Inhibitors, Nonsteroidal Immunosuppressant Therapy, or Biologics: A Retrospective Cohort Study in the United States. Journal of Managed Care and Specialty Pharmacy (2022) 28(10):S78-S9. 30. Blum S, Gillis D, Brown H, Boyle R, Henderson R, Heyworth-Smith D, et al. Use and Monitoring of Low Dose Rituximab in Myasthenia Gravis. J Neurol Neurosurg Psychiatry (2011) 82(6):659-63. Epub 2010/11/13. doi: 10.1136/jnnp.2010.220475. 31. Usman U, Chrisman C, Houston D, Haws CC, Wang A, Muley S. The Use of Eculizumab in Ventilator-Dependent Myasthenia Gravis Patients. Muscle Nerve (2021) 64(2):212-5. Epub 2021/05/20. doi: 10.1002/mus.27326. 32. Sachdeva J, Mahesh KV, Shree R, Jain G, Kapila AT, Shashikala TP, et al. Use of Rituximab in Muscle-Specific Tyrosine Kinase Antibody-Positive Myasthenia Gravis: Preliminary Observations from a Tertiary Care Center in Northern India. Indian J Pharmacol (2020) 52(1):49-52. Epub 2020/03/24. doi: 10.4103/ijp.IJP_333_18. 33. Maddison P, McConville J, Farrugia ME, Davies N, Rose M, Norwood F, et al. The Use of Rituximab in Myasthenia Gravis and Lambert-Eaton Myasthenic Syndrome. J Neurol Neurosurg Psychiatry (2011) 82(6):671-3. Epub 2010/04/16. doi: 10.1136/jnnp.2009.197632. 34. Iwasa K, Furukawa Y, Yoshikawa H, Yamada M, Ono K. Cd59 Expression in Skeletal Muscles and Its Role in Myasthenia Gravis. Neurology(R) neuroimmunology & neuroinflammation (2023) 10(1). doi: 10.1212/NXI.0000000000200057. 35. Wang C, Zeng H, Fang W, Song L. Clinical Characteristics, Treatment and Outcome of Nivolumab-Induced Myasthenia Gravis. Invest New Drugs (2023) 41(2):333-9. Epub 2023/03/30. doi: 10.1007/s10637-023-01347-6. 36. Jing S, Lu J, Song J, Luo S, Zhou L, Quan C, et al. Effect of Low-Dose Rituximab Treatment on T- and B-Cell Lymphocyte Imbalance in Refractory Myasthenia Gravis. J Neuroimmunol (2019) 332:216-23. Epub 2019/05/18. doi: 10.1016/j.jneuroim.2019.05.004. 37. Ruetsch-Chelli C, Bresch S, Seitz-Polski B, Rosenthal A, Desnuelle C, Cohen M, et al. Memory B Cells Predict Relapse in Rituximab-Treated Myasthenia Gravis. Neurotherapeutics (2021) 18(2):938-48. Epub 2021/03/27. doi: 10.1007/s13311-021-01006-9. 38. Zebardast N, Patwa HS, Novella SP, Goldstein JM. Rituximab in the Management of Refractory Myasthenia Gravis. Muscle Nerve (2010) 41(3):375-8. Epub 2009/10/24. doi: 10.1002/mus.21521. 39. Roda RH, Doherty L, Corse AM. Stopping Oral Steroid-Sparing Agents at Initiation of Rituximab in Myasthenia Gravis. Neuromuscul Disord (2019) 29(7):554-61. Epub 2019/07/13. doi: 10.1016/j.nmd.2019.06.002. 40. Illa I, Diaz-Manera J, Rojas-Garcia R, Pradas J, Rey A, Blesa R, et al. Sustained Response to Rituximab in Anti-Achr and Anti-Musk Positive Myasthenia Gravis Patients. J Neuroimmunol (2008) 201-202:90-4. Epub 2008/07/26. doi: 10.1016/j.jneuroim.2008.04.039. 41. Lebrun C, Bourg V, Bresch S, Cohen M, Rosenthal-Allieri MA, Desnuelle C, et al. Therapeutic Target of Memory B Cells Depletion Helps to Tailor Administration Frequency of Rituximab in Myasthenia Gravis. J Neuroimmunol (2016) 298:79-81. Epub 2016/09/10. doi: 10.1016/j.jneuroim.2016.07.009. |
| **(6). Protocol (n=7)**   1. Nct. Rituximab EfFicacy IN MyasthEnia Gravis (REFINE) https://clinicaltrialsgov/show/NCT05868837(2023). 2. Nct. A Study of Nipocalimab Administered to Adults with Generalized Myasthenia Gravis https://clinicaltrialsgov/show/NCT04951622(2021). 3. Nct. A Study Evaluating the Safety and Efficacy of Rituximab in Patients with Myasthenia Gravis https://clinicaltrialsgov/show/NCT02950155(2016). 4. Nct. BeatMG: phase II Trial of Rituximab in Myasthenia Gravis https://clinicaltrialsgov/show/NCT02110706(2014). 5. Euctr NL. Study to evaluate the safety and efficacy of ravulizumab in adult patients with generalized Myasthenia Gravis who have never been treated with a complement inhibitor https://trialsearchwhoint/Trial2aspx?TrialID=EUCTR2018-003243-39-NL(2019). 6. Euctr IT. Phase 2 Study of ALXN2050 in Adult Participants with Generalized Myasthenia Gravis https://trialsearchwhoint/Trial2aspx?TrialID=EUCTR2021-001229-26-IT(2021). 7. Euctr FR. Inebilizumab efficacy and safety in adults with myasthenia gravis https://trialsearchwhoint/Trial2aspx?TrialID=EUCTR2020-000949-14-FR(2020). |
| **(7). Case report (n=26)**   1. Takagi K, Yoshida A, Iwasaki H, Inoue H, Ueda T. Anti-CD20 antibody (Rituximab) therapy in a myasthenia gravis patient with follicular lymphoma Ann Hematol(2005) 84: 548-550.doi.org/10.1007/s00277-005-1050-4. 2. Jonsson DI, Pirskanen R, Piehl F. Beneficial effect of tocilizumab in myasthenia gravis refractory to rituximab Neuromuscul Disord(2017) 27: 565-568.doi.org/10.1016/j.nmd.2017.03.007. 3. Hayashi R, Tahara M, Oeda T, Konishi T, Sawada H. [A case of refractory generalized myasthenia gravis with anti-acetylcholine receptor antibodies treated with rituximab] Rinsho Shinkeigaku(2015) 55: 227-232.doi.org/10.5692/clinicalneurol.55.227. 4. Canino F, Pugliese G, Baldessari C, Greco S, Depenni R, Dominici M. Cemiplimab- and nivolumab-induced myasthenia gravis: two clinical cases Tumori(2021) 107: Np123-np126.doi.org/10.1177/03008916211040559. 5. Catzola V, Battaglia A, Buzzonetti A, Fossati M, Scuderi F, Fattorossi A, et al. Changes in regulatory T cells after rituximab in two patients with refractory myasthenia gravis J Neurol(2013) 260: 2163-2165.doi.org/10.1007/s00415-013-6987-y. 6. Kakoulidou M, Bjelak S, Pirskanen R, Lefvert AK. A clinical and immunological study of a myasthenia gravis patient treated with infliximab Acta Neurol Scand(2007) 115: 279-283.doi.org/10.1111/j.1600-0404.2006.00778.x. 7. Katyal N, Nirola L, Narula N, Govindarajan R. Diffuse Gonococcal Infection (DGI) in a Patient with Treatment-Refractory Acetylcholine Receptor Antibody-Positive (AChR+) Generalized Myasthenia Gravis (gMG) Treated with Eculizumab Case Rep Neurol Med(2021) 2021: 9713413.doi.org/10.1155/2021/9713413. 8. Strano CMM, Sorrenti B, Bosco L, Falzone YM, Fazio R, Filippi M. Eculizumab as a fast-acting rescue therapy in a refractory myasthenic crisis: a case report J Neurol(2022) 269: 6152-6154.doi.org/10.1007/s00415-022-11222-8. 9. Takeuchi E, Kajiyama Y, Ando K, Funaki S, Okuno T, Shintani Y, et al. [The efficacy of eculizumab against post-thymectomy exacerbations in thymoma associated myasthenia gravis (MG)] Rinsho Shinkeigaku(2022) 62: 277-280.doi.org/10.5692/clinicalneurol.cn-001682. 10. Mimori M, Komatsu T, Maku T, Mitsumura H, Iguchi Y. Generalized myasthenia gravis patients infected with COVID-19 should continue eculizumab Neurol Sci(2022) 43: 4081-4083.doi.org/10.1007/s10072-022-05922-2. 11. Russell A, Yaraskavitch M, Fok D, Chhibber S, Street L, Korngut L. Obinutuzumab Plus Chlorambucil in a Patient with Severe Myasthenia Gravis and Chronic Lymphocytic Leukemia J Neuromuscul Dis(2017) 4: 251-257.doi.org/10.3233/jnd-170211. 12. Sari PM, Wijaya A, Aryatama, Djojoatmodjo S, Liman J, Freshimona R. P-PN015. Rituximab therapy in post thymectomy refractory myasthenia gravis: A case report Clinical Neurophysiology(2021) 132: e110-e111.doi.org/10.1016/j.clinph.2021.02.266. 13. Burusnukul P, Brennan TD, Cupler EJ. Prolonged improvement after rituximab: two cases of resistant muscle-specific receptor tyrosine kinase + myasthenia gravis J Clin Neuromuscul Dis(2010) 12: 85-87.doi.org/10.1097/CND.0b013e3181fcc109. 14. Waters MJ, Field D, Ravindran J. Refractory myasthenia gravis successfully treated with ofatumumab Muscle Nerve(2019) 60: E45-e47.doi.org/10.1002/mus.26707. 15. Kuntzer T, Carota A, Novy J, Cavassini M, Du Pasquier RA. Rituximab is successful in an HIV-positive patient with MuSK myasthenia gravis Neurology(2011) 76: 757-758.doi.org/10.1212/WNL.0b013e31820d6290. 16. Koul R, Al-Futaisi A, Abdelrahim R, Mani R, Abdwani R, Al-Asmi A. Rituximab Treatment in Myasthaenia Gravis: Report of two paediatric cases Sultan Qaboos Univ Med J(2018) 18: e223-e227.doi.org/10.18295/squmj.2018.18.02.018. 17. Levine TD. Safety of an Abbreviated Transition Period When Switching From Intravenous Immunoglobulin to Eculizumab in Patients with Treatment-Refractory Myasthenia Gravis: A Case Series Am J Case Rep(2019) 20: 965-970.doi.org/10.12659/ajcr.916424. 18. Greenwood GT, Lynch Z. Successful Transition from Plasma Exchange to Eculizumab in Acetylcholine Receptor Antibody- and Muscle-Specific Kinase (MuSK) Antibody-Negative Myasthenia Gravis: A Case Report Am J Case Rep(2020) 21: e921431.doi.org/10.12659/ajcr.921431. 19. Hain B, Jordan K, Deschauer M, Zierz S. Successful treatment of MuSK antibody-positive myasthenia gravis with rituximab Muscle Nerve(2006) 33: 575-580.doi.org/10.1002/mus.20479. 20. Wylam ME, Anderson PM, Kuntz NL, Rodriguez V. Successful treatment of refractory myasthenia gravis using rituximab: a pediatric case report J Pediatr(2003) 143: 674-677.doi.org/10.1067/s0022-3476(03)00300-7. 21. Al-Shehri E, Al-Mowallad E, Al-Said Y, Cupler EJ. Sustained remission in a case of musk (+) myasthenia gravis treated with i.v. rituximab as primary therapy European journal of neurology(2014) 21: 519 22. Kakoulidou M, Pirskanen-Matell R, Lefvert AK. Treatment of a patient with myasthenia gravis using antibodies against CD25 Acta Neurol Scand(2008) 117: 211-216.doi.org/10.1111/j.1600-0404.2007.00919.x. 23. Zaja F, Russo D, Fuga G, Perella G, Baccarani M. Rituximab for myasthenia gravis developing after bone marrow transplant Neurology(2000) 55: 1062-1063.doi.org/10.1212/wnl.55.7.1062-a. 24. Stieglbauer K, Topakian R, Schäffer V, Aichner FT. Rituximab for myasthenia gravis: three case reports and review of the literature J Neurol Sci(2009) 280: 120-122.doi.org/10.1016/j.jns.2009.02.357. 25. Sahai SK, Maghzi AH, Lewis RA. Rituximab in late-onset myasthenia gravis is safe and effective Muscle Nerve(2020) 62: 377-380.doi.org/10.1002/mus.26876. 26. Sadnicka A, Reilly MM, Mummery C, Brandner S, Hirsch N, Lunn MP. Rituximab in the treatment of three coexistent neurological autoimmune diseases: chronic inflammatory demyelinating polyradiculoneuropathy, Morvan syndrome and myasthenia gravis J Neurol Neurosurg Psychiatry(2011) 82: 230-232.doi.org/10.1136/jnnp.2009.174888. |
| **(8). Letter (n=2)**   1. Zouvelou V, Psimenou E. Double Seropositive Myasthenia Gravis Successfully Treated With Rituximab. J Clin Neuromuscul Dis (2022) 24: 116-117.doi.org/10.1097/cnd.0000000000000410. 2. Dos Santos A, Pereon Y, Wiertlewski S, Magot A. Response to Finsterer and colleagues on 'Prospective studies on the efficacy of rituximab for myasthenia gravis are warranted'. Eur J Neurol (2020) 27: e94.doi.org/10.1111/ene.14460. |
| **(9). Exceed inclusion criteria (n=2)**   1. Howard JF, Jr., Nowak RJ, Wolfe GI, Freimer ML, Vu TH, Hinton JL, et al. Clinical Effects of the Self-administered Subcutaneous Complement Inhibitor Zilucoplan in Patients With Moderate to Severe Generalized Myasthenia Gravis: Results of a Phase 2 Randomized, Double-Blind, Placebo-Controlled, Multicenter Clinical Trial. JAMA Neurol (2020) 77: 582-592.doi.org/10.1001/jamaneurol.2019.5125. 2. Bril V, Benatar M, Andersen H, Vissing J, Brock M, Greve B, et al. Efficacy and Safety of Rozanolixizumab in Moderate to Severe Generalized Myasthenia Gravis: A Phase 2 Randomized Control Trial. Neurology (2021) 96: e853-e865.doi.org/10.1212/wnl.0000000000011108. |

**Table S3: Inclusion, exclusion criteria, study design and outcome assessments of the included studies**

**FcRn inhibitors:**

| **Trials** | **Bril et al. 2023 (1)**  **(NCT03971422)** |
| --- | --- |
| ***Inclusion Criteria*** | ≥18 years old; Myasthenia Gravis Foundation of America (MGFA) Class II to IV; positive for AChR/MuSK antibodies; MG-ADL score ≥3(for non-ocular symptoms); QMG≥11; additional therapy such as intravenous immunoglobulin or plasma exchange; bodyweight at least 35 kg; Permitted concomitant medications were cholinesterase inhibitors (stable dose not required), oral corticosteroids (stable for 4 weeks before baseline), azathioprine, ciclosporin, methotrexate, mycophenolate mofetil, and tacrolimus (all received for the previous 6 months and on a stable dose 2 months before baseline). |
| ***Exclusion Criteria*** | Severe oropharyngeal or respiratory weakness; clinically relevant active infection; recent serious infection, a total IgG concentration of no more than 5.5 g/L; hypersensitivity to any components of the study medication; pregnancy or breastfeeding; Prohibited concomitant medications were intravenous immunoglobulin or plasma exchange (other than when used as rescue therapy), biological agents (including rituximab and eculizumab), cyclophosphamide, pimecrolimus, immunoadsorption, and vinca alkaloids |
| ***Study design*** | Patients were randomly assigned (1:1:1) to receive subcutaneous infusions once a week for 6 weeks of either rozanolixizumab 7 mg/kg, rozanolixizumab 10 mg/kg, or placebo |
| ***Efficacy outcomes*** | Change from baseline for QMG score, MGC score, MG-ADL score, MG QOL-15 and Myasthenia Gravis Symptoms Patient-Reported Outcome scales at day 43; MGC and QMG responses (based on the clinically meaningful improvement of ≥3 points) at day 43; changes in MG-ADL, MGC, QMG excluding ocular items and PRO scales each week; changes in MG-ADL; the proportion of patients with MG-ADL, MGC, and QMG response each week; time to MG-ADL response; minimal symptom expression (MG-ADL score of 0 or 1); change in Myasthenia Gravis Impairment Index (MGII) score; change in MGII ocular and generalised domain subscores; Patient Global Impression of Severity; Patient Global Impression of Change; change in Myasthenia Gravis Quality of Life 15-item scale revised score, European Quality of Life 5 Dimensions 5 Levels (EQ-5D-5L); use of rescue therapy; time to rescue therapy. |
| ***Safety outcomes*** | AEs; SAEs; All-Cause Mortality; more frequently reported AEs; TEAEs leading to discontinuation; change from baseline in vital signs, electrocardiogram, laboratory, and suicidality. |

| **Trials** | **Yan et al. 2022 (2)**  **(NCT04346888)** |
| --- | --- |
| ***Inclusion Criteria*** | ≥18 years old; MGFA Class II a to IV a; positive for AChR/MuSK antibodies; MG-ADL score ≥ 6 and with eye muscle score accounting for < 50%; stable MG treatments at baseline, including acetylcholinesterase inhibitors, corticosteroids, and/or immunosuppressants; The diagnosis of MG was supported by a history of abnormal repetitive nerve stimulation test, a positive edrophonium chloride test, or improvement with acetylcholinesterase inhibitors, and positivity of AChR-Ab or MuSK-Ab. |
| ***Exclusion Criteria*** | History of malignancy; MGFA Class IV b or V; thymectomy or radiation therapy for<12 months; intravenous administration of IgG, or plasmapheresis/plasma exchange<4 weeks before the screening; immunosuppressive monoclonal antibodies ((e.g., rituximab, bevacizumab, eculizumab, etc.) administered for <6 months prior to screening; other uncontrolled autoimmune diseases potentially interfering with the study course; |
| ***Study design*** | In the double-blinded treatment period, eligible patients received batoclimab (680 mg), batoclimab (340 mg), or placebo on days 1, 8, 15, 22, 29, and 36. In the open-label treatment period, patients received batoclimab (340 mg) on days 50, 64, and 78. In the follow-up period, patients were examined on days 92, 106, and 120. |
| ***Efficacy outcomes*** | Change from baseline for QMG score, MGC score, MG-ADL score, MG QOL-15 at day 43; Change from baseline for QMG score, MGC score, MG-ADL score, MG QOL-15 at day 120; the percentage of patients with clinically significant improvement based on MG-ADL (defined as 2 or more points improvement) and QMG (defined as 3 or more points improvement) scores on day 43 compared with the baseline; the percentage of patients with sustained improvement from baseline to day 120 (improvement in MG-ADL score≥2 or in QMG score≥3 for 4 consecutive weeks); Minimal symptom expression was defined as a total MG-ADL score of 0-1. |
| ***Safety outcomes*** | AEs; SAEs; All-Cause Mortality; More frequently reported AEs; Changes in albumin levels from baseline; Pharmacodynamic endpoints included serum total IgG level changes from baseline to day 120. |

| **Trials** | **Howard et al. 2019 (3)**  **(NCT02965573)** |
| --- | --- |
| ***Inclusion Criteria*** | ≥18 years old; MGFA Class II to IV a; positive for AChR antibodies; MG-ADL score≥5; baseline with more than 50% of the score attributable to nonocular items; a stable dose of their standard-of-care MG treatment prior to randomization. |
| ***Exclusion Criteria*** | Malignant thymoma; MGFA Class I, IV b or V; thymectomy performed <3 months; monoclonal antibody for immunomodulation within 6 months prior to first dosing (or in case of prior rituximab treatment with CD19 counts below the normal range); take any biological therapy or investigational drug within 3 months or 5 half-lives of the drug before screening; received IV or intramuscular immunoglobulin or plasmapheresis/plasma exchange within 4 weeks before screening |
| ***Study design*** | A phase 2, exploratory, randomized, double-blind, placebo-controlled, 15-center study is described. Eligible patients were randomly assigned (1:1) to receive 4 doses over a 3-week period of either 10 mg/kg IV efgartigimod or matched placebo combined with their standard-of-care therapy. |
| ***Efficacy outcomes*** | Change from baseline for QMG score, MGC score, MG-ADL score, MG QOL-15 at day 78; total immunoglobulin G (IgG) and anti-AChR autoantibody levels. |
| ***Safety outcomes*** | AEs; SAEs; All-Cause Mortality; more frequently reported AEs; Vital signs, ECGs, clinical laboratory. |

| **Trials** | **Howard et al. 2021 (4)**  **(NCT03669588)** |
| --- | --- |
| ***Inclusion Criteria*** | ≥18 years old; MGFA Class II to IV; MG-ADL score≥5(with >50% of the MG-ADL score due to non-ocular symptoms); Diagnosis was supported by a history of abnormal neuromuscular transmission tests, a positive edrophonium chloride test, or improvement with acetylcholinesterase inhibitors; a stable dose of at least one treatment for gMG (ie, acetylcholinesterase inhibitors, corticosteroids, or NSISTs) before screening and throughout the trial. |
| ***Exclusion Criteria*** | Received rituximab or eculizumab in the 6 months before screening, undergone thymectomy within 3 months; intravenous immunoglobulin or plasma exchange within 1 month of screening; active hepatitis B; seropositive for hepatitis C, seropositive for HIV with low CD4 count; serum IgG levels less than 6 g/L at screening; pregnant. |
| ***Study design*** | Patients were randomly assigned by interactive response technology (1:1) to efgartigimod (10 mg/kg) or matching placebo, administered as four infusions per cycle (one infusion per week), repeated as needed depending on clinical response no sooner than 8 weeks after initiation of the previous cycle. |
| ***Efficacy outcomes*** | Minimal symptom expression; Change from baseline for QMG score, MGC score, MG-ADL score, MG QOL-15 at day 43; MG-ADL responder (at least a 2-point improvement in MG-ADL score, sustained for at least 4 consecutive weeks, with the first improvement occurring by week 4 of the cycle); proportion of QMG responders (defined as a ≥3 point improvement in the total QMG score for ≥4 consecutive weeks with the first improvement occurring by week 4 of cycle 1) in the acetylcholine receptor antibody-positive population; percentage of MG-ADL responders in cycle 1 in the overall population; proportion of time patients showed a clinically meaningful improvement in MG-ADL score in the AChR positive, up to day 126; time from day 28 to not having clinically meaningful improvement in the AChR-positive population; proportion of early MG-ADL responders in cycle 1 in AChR-positive population. |
| ***Safety outcomes*** | AEs; SAEs; All-Cause Mortality; more frequently reported AEs; changes in clinical laboratory values and vital signs, and on electrocardiograms. |

| **Trials** | **NCT03772587 (5)** |
| --- | --- |
| ***Inclusion Criteria*** | ≥18 years old; documented history of Generalized Myasthenia Gravis (gMG) and clinical signs/symptoms of gMG. |
| ***Exclusion Criteria*** | pregnant or breastfeeding; history of any neurologic disorder other than MG that might interfere with the accuracy of study assessments. |
| ***Study design*** | the placebo group: Participants received intravenous (IV) infusion of placebo matching to nipocalimab once every 2 weeks (Q2W) starting Day 1 up to Day 57; in the Nipocalimab 5mg/kg group: Participants received IV infusion of 5 mg/kg nipocalimab once every 4 weeks (Q4W) starting Day 1 up to Day 57. To maintain blinding, participants received matching placebo on Days 15 and 43; in the Nipocalimab 30mg/kg group: Participants received IV infusion of 30 mg/kg nipocalimab once Q4W starting Day 1 up to Day 57. To maintain blinding, participants received matching placebo on Days 15 and 43; in the Nipocalimab 60mg/kg group: Participants received IV infusion of 60 mg/kg nipocalimab single dose on Day 1. To maintain blinding, participants received matching placebo on Days 15, 29, 43 and 57. |
| ***Efficacy outcomes*** | change from baseline for QMG score, MG-ADL score, MG QOL-15 at day 57; change from baseline in total MG-ADL score as a function of total serum immunoglobulin G (IgG) at day 57; change from baseline in total MG-ADL score as a response to percent change in total serum IgG for AChR positive patients at Day 57; number of participants with a 2-, 3-, 4-, 5-, 6-, 7-, or greater than or equal to≥8-point improvement in total MG-ADL score and with a 3-, 4-, 5-, 6-, 7-, or≥8-point improvement in total QMG Score at day 57; change from baseline in total serum IgG at day 57; change from baseline for QMG score, MG-ADL score, MG QOL-15 at day 85 and day 113. |
| ***Safety outcomes*** | AEs; SAEs; All-Cause Mortality; more frequently reported AEs; number of participants with treatment-emergent adverse events of special interest. |

**Complement inhibitors**

| **Trials** | **Howard et al. 2023 (6)**  **(NCT04115293)** |
| --- | --- |
| ***Inclusion Criteria*** | MGFA Class II-IV; positive for AChR antibodies; MG-ADL Score ≥ 6; QMG score ≥ 12; no change in corticosteroid dose for at least 30 days prior to Baseline or anticipated to occur during the 12-week Treatment Period; no change in immunosuppressive therapy, including dose, for at least 30 days prior to Baseline or anticipated to occur during the 12-week Treatment Period. |
| ***Exclusion Criteria*** | Thymectomy within 12 months prior to baseline or scheduled to occur during the 12week treatment period; History of meningococcal disease; current or recent systemic infection within 2 weeks prior to baseline or injection requiring intravenous (IV) antibiotics within 4 weeks prior to baseline; previous treatment with a complement inhibitor, treatment with rituximab within 12 months, or treatment with intravenous immunoglobulin, subcutaneous immunoglobulin, or plasma exchange within 4 weeks before baseline; participating in a clinical trial of an experimental intervention. |
| ***Study design*** | We randomly assigned (1:1) participants to receive zilucoplan or matched placebo within permuted blocks of four using an interactive response technology. Randomization was stratified by baseline MG-ADL score (≤9 vs ≥10), QMG score (≤17 vs ≥18), and geographical region (Europe, Japan, or North America). The sponsor, investigators, clinical evaluators, and participants were masked to treatment allocation. Subcutaneous doses of zilucoplan 0.3 mg/kg or matched placebo were administered daily at home. |
| ***Efficacy outcomes*** | change from baseline for QMG score, MGC score, MG-ADL score, MG QOL-15 at Week 12; A 2-point change in MG-ADL score; a 3-point change in QMG score,30 and a 3-point change in MGC Score; the proportion of participants achieving a reduction of at least 3 points in MG-ADL score at week 12 without rescue therapy; proportion of patients achieving a reduction of at least 5 points in QMG score at week 12 without rescue therapy; time to first receipt of rescue therapy over 12 weeks; proportion of participants achieving minimal symptom expression (defined as an MG-ADL score of 0 or 1 without rescue therapy) at week 12. |
| ***Safety outcomes*** | AEs; SAEs; All-Cause Mortality; more frequently reported AEs; clinical laboratory tests, electrocardiograms, immunogenicity, vital signs, physical  examinations, and the Columbia-Suicide Severity Rating Scale. |

| **Trials** | **Howard et al. 2017 (7)**  **(NCT01997229)** |
| --- | --- |
| ***Inclusion Criteria*** | ≥18 years old; MGFA Class II to IV; MG-ADL total score ≥6; history of positive anticholinesterase test; improvement in MG signs on oral cholinesterase inhibitors; Failed treatment with at least two immunosuppressive agents; Failed treatment with at least one immunosuppressive agent and require chronic plasma exchange or IVIg. |
| ***Exclusion Criteria*** | History of thymoma or other neoplasms of the thymus; History of thymectomy within 12 months; MGFA Class I or V); rituximab within 6 months prior to screening; IVIg or PE within 4 weeks prior to Randomization. |
| ***Study design*** | We randomly assigned participants (1:1) to 26 weeks of either intravenous eculizumab or intravenous placebo. Placebo was matched to eculizumab in appearance and supplied in identical containers to preserve masking. The schedule for eculizumab was induction dosing 900 mg on day 1 and weeks 1, 2, and 3; 1200 mg at week 4; and maintenance dosing 1200 mg every second week thereafter. Placebo was given on the same schedule. |
| ***Efficacy outcomes*** | Change from baseline for QMG score, MGC score, MG-ADL score, MG QOL-15 at Week 26; responder analysis of the MG-ADL score (≥3-point  improvement); responder analysis of the QMG score (≥5-point improvement). |
| ***Safety outcomes*** | SAEs; All-Cause Mortality; more frequently reported AEs; admissions to hospital, protocol-defined clinical deterioration with or without rescue medication use; changes in vital signs; electrocardiography; clinical; laboratory variables. |

| **Trials** | **TuanVu et al. 2022 (8)**  **(NCT03920293)** |
| --- | --- |
| ***Inclusion Criteria*** | Diagnosed with Myasthenia Gravis at least 6 months; MGFA Class II to IV; MG-ADL score≥ 6; Vaccinated against meningococcal infections within 3 years prior to, or at the time of, initiating study drug. |
| ***Exclusion Criteria*** | Active or untreated thymoma; History of thymic carcinoma or thymic malignancy with no evidence of recurrence for ≥ 5 years; History of thymectomy within the 12 months; History of N meningitidis infection; IV immunoglobulin within 4 weeks; Use of plasma exchange within 4 weeks; Use of rituximab within 6 months; Participants who have received previous treatment with complement inhibitors. |
| ***Study design*** | patients were randomized 1:1 to intravenous ravulizumab or matching placebo. Ravulizumab dosing during the study was based on patients’ body weight. Patients received an initial loading dose of 2400, 2700, or 3000 mg on Day 1, followed by a maintenance dose of 3000, 3300, or 3600 mg starting on Day 15 and every 8 weeks thereafter. |
| ***Efficacy outcomes*** | Change from baseline for QMG score, MG-ADL score, MG QOL-15, Neurological Quality of Life (Neuro-QoL) Fatigue Score at Week 26; percentage of patients of MG-ADL total score reduction of at least 3 Points At Week 26; |
| ***Safety outcomes*** | SAEs; All-Cause Mortality; more frequently reported AEs. |

**B-Cells inhibitors**

| **Trials** | **Piehl et al. 2022 (9)**  **(NCT02950155)** |
| --- | --- |
| ***Inclusion Criteria*** | ≥18 years old; onset of gMG symptoms 12 months or less prior to inclusion; QMG≥6; MGFA Class II-IV; The MG diagnosis had to be confirmed by at least 2: a positive AChR antibody test result, an abnormal electrophysiological test result (repetitive nerve stimulation and/or single fiber electromyography) consistent with MG, and/or a clinically significant response to an oral or intravenous AChEI test. |
| ***Exclusion Criteria*** | MGFA class I or V; prior thymectomy or suspected thymoma; pulsed high-dose corticosteroids, rituximab, azathioprine, ciclosporin, and mycophenolate for any condition for 12 months or less prior to inclusion. Neither treatment with prednisolone, 40 mg/d or less, for a maximum of 3 months nor intravenous immunoglobulins or plasma exchange within 12 months of screening. |
| ***Study design*** | Eligible participants were randomly assigned 1:1 without stratification to receive an intravenous infusion of 500 mg of rituximab or matched placebo. |
| ***Efficacy outcomes*** | change from baseline for QMG score, MG-ADL score at Week 16; Minimal disease manifestations (defined as a QMG score of 4 or less and a daily dose of prednisolone of 10 mg/d or less at week 16, with no need of rescue treatment procedure(s) during study weeks 9 to 16);Tertiary outcomes included the primary end point evaluated at 24 weeks, proportion having received rescue treatment by week 24; change in QMG, MG-ADL, and MG-QoL 15r scores at each study visit; hospitalization for worsened MG symptoms, and AChR antibody concentrations at week 24. |
| ***Safety outcomes*** | AEs; SAEs; more frequently reported AEs. |

| **Trials** | **Nowak et al. 2021 (10)**  **(NCT02110706)** |
| --- | --- |
| ***Inclusion Criteria*** | 21-90 years old; positive for AChR antibodies; MGFA Class II-IV; a stable dose of either prednisone alone (≥15 mg/day) for 4-weeks, or prednisone and a stable dose of another IST for ≥6 months prior to baseline. Azathioprine, mycophenolate mofetil, cyclosporine, tacrolimus and methotrexate were permitted. |
| ***Exclusion Criteria*** | active or untreated thymoma; a history of thymic carcinoma or thymic malignancy (unless deemed cured by adequate treatment with no evidence of recurrence for 5 years or more before screening); a history of thymectomy in the 12 months before screening; a history of Neisseria meningitidis infection, use of intravenous immunoglobulin or plasma exchange in the 4 weeks before randomization, use of rituximab in the 6 months before screening, or previous treatment with a complement inhibitor (e.g., eculizumab). |
| ***Study design*** | eligible patients were randomly assigned (1:1) at baseline (day 1) to receive either ravulizumab or placebo for 26 weeks. Ravulizumab dosing was based on the patient’s body weight. Patients received an initial loading dose of ravulizumab (2400, 2700, or 3000 mg) or placebo at baseline (day 1), followed by maintenance doses of ravulizumab (3000, 3300, or 3600 mg) or placebo on day 15 (week 2) and every 8 weeks thereafter. |
| ***Efficacy outcomes*** | Change from baseline for QMG score, MGC score, MG-ADL score at Week 52; AChR-Ab levels were measured at baseline, week-24 and 52 (reference range: positive >0.02 nmol/L); Total B-cell counts (CD19+/CD20- plus CD19+/CD20+ cells) were measured at baseline, week-24 and 52. |
| ***Safety outcomes*** | AEs; SAEs; more frequently reported AEs. |

| **Trials** | **Hewett et al. 2018 (11)**  **(NCT01480596)** |
| --- | --- |
| ***Inclusion Criteria*** | MGFA class II-Iva inclusive; positive for AChR/MuSK antibodies; QMG score ≥8, of which ≥4 score from signs other than ocular; must receive one or more the treatment: a cholinesterase inhibitor (min 2 week), prednisone (min 1 month), one of the following immunosuppressants: cyclosporine (min 3 months), methotrexate (min 3 months), azathioprine (min 6 months), or mycophenolate (min 6 months); not pregnant or nursing and agreed to several predefined criteria designed to prevent pregnancy from enrollment to 16 weeks post last dose of study treatment. |
| ***Exclusion Criteria*** | History of thymoma, thymectomy within 12 months; IVIg or PLEX within 4 weeks; rituximab or eculizumab within 12 months; any B cell targeted therapy (including belimumab) at any time; treatment with any immunosuppressive agent other than those permitted within the inclusion criteria within 6 months; a history of recurrent or chronic infection or currently active systemic infection. |
| ***Study design*** | Eligible participants with MG were randomized 1:1 to receive IV belimumab 10 mg/kg or placebo in this phase II, placebo-controlled, multicenter, double-blind study. Participants received standard of care therapies throughout the 24-week treatment phase and 12-week follow-up period. |
| ***Efficacy outcomes*** | change from baseline for QMG score, MGC score, MG-ADL score at Week 24; proportion of participants with ≥3-point QMG/MGC improvement or worsening from baseline by week 24 and with a sustained improvement of ≥3 points from baseline at week 12 maintained to week 24; mean changes from baseline in QMG, MGC, and MG-ADL scores during the follow-up period at weeks 28, 32, and 36. |
| ***Safety outcomes*** | AEs; SAEs; Treatment discontinuation due to TEAE; potential clinical concern (vital sign, hematology and clinical chemistry parameters). |

| **Trials** | **NCT02565576 (12)** |
| --- | --- |
| ***Inclusion Criteria*** | MGFA class II-IVa inclusive; QMG score ≥10; positive for AChR/MuSK antibodies; Only one immunosuppressant or immunomodulatory drug at a stable dose: azathioprine and mycophenolate mofetil (min 4 months), cyclosporine (min 3 months); oral corticosteroids, methotrexate or tacrolimus (min 1 month), cholinesterase inhibitors (min 2 weeks) prior to randomization; Women of child-bearing potential use contraception during the study and for 12 weeks after study treatment. |
| ***Exclusion Criteria*** | MGFA grade I, IVb, or V disease; unresected thymoma; thymectomy within 6 months of screening; any of the following treatments prior to randomization: IVIg or plasma exchange within 8 weeks; oral or IV cyclosphosphamide treatment within 3 months; IV corticosteroid bolus (dose higher than 1 mg/kg) within 3 months; Belimumab within 6 months; Rituximab within 12 months; Live vaccines within 4 weeks; any other biologic or an investigational drug within 1 month or five times the half-life;  History of either thrombosis or 3 or more spontaneous abortions with or without the presence of anti-cardiolipin autoantibodies; Presence of prolonged partial thromboplastin time (PTT). |
| ***Study design*** | Eligible participants with MG were randomized 1:1 to receive IV Iscalimab 10 mg/kg or placebo in this phase II, placebo-controlled, multicenter, double-blind study. |
| ***Efficacy outcomes*** | change from baseline for QMG score, MGC score, MG-ADL score, MG QOL-15 at Week 25; proportion of patients with improvement or worsening by ≥ 3 Points in the QMG Score at week 49; proportion of patients intolerant to steroid taper at week 49; proportion of patients who discontinued due to inefficacy or worsening to week 49; Mean changes from baseline in the QMG score, MGC score. |
| ***Safety outcomes*** | SAEs; All-Cause Mortality; more frequently reported AEs; |

| **Trials** | **NCT04159805 (13)** |
| --- | --- |
| ***Inclusion Criteria*** | Diagnosis of Myasthenia Gravis (MG); positive for AChR/MuSK antibodies; MGFA class II-IV inclusive; QMG score ≥6, of which ≥4 score from signs other than ocular; If receiving immunosuppressive drugs, therapy must be ongoing for at least 6 months, with stable dosing ongoing for at least 3 months before screening. Participants receiving azathioprine must be on a stable dose for at least 6 months before screening; If receiving oral corticosteroids, therapy must be ongoing for at least 3 months, with a stable dose at least 1 month before screening. Corticosteroids, including dexamethasone, must be given as oral, daily or every-other-day therapy, as opposed to pulse therapy; If receiving cholinesterase inhibitors, therapy with a stable dose is required at least 2 weeks before screening; |
| ***Exclusion Criteria*** | Presence of a thymoma; history of invasive thymic malignancy unless deemed cured by adequate treatment with no evidence of recurrence for ≥ 5 years before screening; History of thymectomy within 12 months before screening; MGFA class I or V; received intravenous immunoglobulin (IVIg), subcutaneous immunoglobulin (SCIg), or plasmapheresis/plasma exchange within 4 weeks before screening; Received rituximab, belimumab, eculizumab, or any monoclonal antibody for immunomodulation within 6 months before first dosing; Known autoimmune disease other than MG that could interfere with the course and conduct of the study; Received a live vaccine within 4 weeks before screening or has any live vaccination planned during the study; Opportunistic infection ≤12 weeks before initial study dosing or currently receiving treatment for a chronic opportunistic infection; |
| ***Study design*** | A Phase 2, Randomized, Placebo-Controlled Study to Evaluate Safety, Tolerability, and Efficacy of Mezagitamab in Patients with Generalized Myasthenia Gravis; Mezagitamab 300 mg or 600 mg injection, SC, once weekly in combination with standard background therapy for 8 weeks; |
| ***Efficacy outcomes*** | change from baseline for QMG score, MGC score, MG-ADL score, MG QOL-15 at Week 32; change from baseline in AChR antibody levels to week 32; change from baseline in MuSK antibody levels to week 32; percentage of participants with 2-point reduction in MG-ADL total score, 3-point reduction in QMG total score, 3-point reduction in MGC total score, at week 4, 6, 8, 10, 12, 14, 16, 20, 24, 28 and 32. |
| ***Safety outcomes*** | AEs; SAEs; All-Cause Mortality; more frequently reported AEs; |

Abbreviation: MG: Myasthenia Gravis; AChR: Acetylcholine receptor; MuSK: Muscle-specific kinase; MGFA: Myasthenia Gravis Foundation of America; MG-ADL: Myasthenia Gravis Activities of Daily Living score; QMG: Quantitative Myasthenia Gravis score; MGC: Myasthenia Gravis Composite score;

MG-QoL 15r: 15-item revised version of the Myasthenia Gravis Quality of Life score; AEs: adverse effects; SAEs: serious adverse events;

**Figure S4: Certainty of evidence for primary outcomes and efficacy outcome in league table.**

| Comparison | Number of studies | Within-study bias | Reporting bias | Indirectness | Imprecision | Heterogeneity | Incoherence | Confidence rating |
| --- | --- | --- | --- | --- | --- | --- | --- | --- |
| **1. MG-ADL** | | | | | | | | |
| Batoclimab_340mg: Batoclimab_680mg | 1 | No concerns | Low risk | No concerns | Major concerns | No concerns | Major concerns | High |
| Batoclimab_340mg: Placebo | 1 | No concerns | Low risk | No concerns | No concerns | Some concerns | Major concerns | low |
| Batoclimab_680mg: Placebo | 1 | No concerns | Low risk | No concerns | No concerns | Major concerns | Major concerns | low |
| Belimumab: Placebo | 1 | No concerns | Low risk | No concerns | Major concerns | No concerns | Major concerns | High |
| Eculizumab: Placebo | 1 | No concerns | Low risk | No concerns | No concerns | Major concerns | Major concerns | low |
| Efgartigimod_10mg_kg: Placebo | 2 | No concerns | Low risk | No concerns | No concerns | Major concerns | Major concerns | low |
| Iscalimab: Placebo | 1 | No concerns | Low risk | No concerns | Major concerns | No concerns | Major concerns | High |
| Mezagitamab_300mg: Mezagitamab_600mg | 1 | No concerns | Low risk | No concerns | Some concerns | Some concerns | Major concerns | Moderate |
| Mezagitamab_300mg: Placebo | 1 | No concerns | Low risk | No concerns | Major concerns | No concerns | Major concerns | High |
| Mezagitamab_600mg: Placebo | 1 | No concerns | Low risk | No concerns | Major concerns | No concerns | Major concerns | High |
| Nipocalimab_30mg_kg: Nipocalimab_5mg_kg | 1 | No concerns | Low risk | No concerns | Major concerns | No concerns | Major concerns | High |
| Nipocalimab_30mg_kg: Nipocalimab_60mg_kg | 1 | No concerns | Low risk | No concerns | No concerns | No concerns | Major concerns | Moderate |
| Nipocalimab_30mg_kg: Placebo | 1 | No concerns | Low risk | No concerns | Major concerns | No concerns | Major concerns | High |
| Nipocalimab_5mg_kg: Nipocalimab_60mg_kg | 1 | No concerns | Low risk | No concerns | Some concerns | Some concerns | Major concerns | Moderate |
| Nipocalimab_5mg_kg: Placebo | 1 | No concerns | Low risk | No concerns | Major concerns | No concerns | Major concerns | High |
| Nipocalimab_60mg_kg: Placebo | 1 | No concerns | Low risk | No concerns | Some concerns | Some concerns | Major concerns | Moderate |
| Placebo: Ravulizumab | 1 | No concerns | Low risk | No concerns | No concerns | No concerns | Major concerns | High |
| Placebo: Rituximab | 2 | No concerns | Low risk | No concerns | Some concerns | Some concerns | Major concerns | Moderate |
| Placebo: Rozanolixzumb_10mg_kg | 1 | Some concerns | Low risk | No concerns | No concerns | Major concerns | Major concerns | Very low |
| Placebo: Rozanolixzumb_7mg_kg | 1 | Some concerns | Low risk | No concerns | No concerns | Major concerns | Major concerns | Very low |
| Placebo: Zilucoplan | 1 | No concerns | Low risk | No concerns | No concerns | Some concerns | Major concerns | Moderate |
| Rozanolixzumb_10mg_kg: Rozanolixzumb_7mg_kg | 1 | Some concerns | Low risk | No concerns | Major concerns | No concerns | Major concerns | Moderate |
| Batoclimab_340mg: Belimumab | 0 | No concerns | Low risk | No concerns | Some concerns | Some concerns | Major concerns | Moderate |
| Batoclimab_340mg: Eculizumab | 0 | No concerns | Low risk | No concerns | Some concerns | Some concerns | Major concerns | Moderate |
| Batoclimab_340mg: Efgartigimod_10mg_kg | 0 | No concerns | Low risk | No concerns | No concerns | Major concerns | Major concerns | Very low |
| Batoclimab_340mg: Iscalimab | 0 | No concerns | Low risk | No concerns | No concerns | Major concerns | Major concerns | Very low |
| Batoclimab_340mg: Mezagitamab_300mg | 0 | No concerns | Low risk | No concerns | Some concerns | Some concerns | Major concerns | Very low |
| Batoclimab_340mg: Mezagitamab_600mg | 0 | No concerns | Low risk | No concerns | No concerns | Major concerns | Major concerns | Very low |
| Batoclimab_340mg: Nipocalimab_30mg_kg | 0 | No concerns | Low risk | No concerns | No concerns | Major concerns | Major concerns | Very low |
| Batoclimab_340mg: Nipocalimab_5mg_kg | 0 | No concerns | Low risk | No concerns | No concerns | Major concerns | Major concerns | Very low |
| Batoclimab_340mg: Nipocalimab_60mg_kg | 0 | No concerns | Low risk | No concerns | No concerns | Major concerns | Major concerns | Very low |
| Batoclimab_340mg: Ravulizumab | 0 | No concerns | Low risk | No concerns | No concerns | Major concerns | Major concerns | Very low |
| Batoclimab_340mg: Rituximab | 0 | No concerns | Low risk | No concerns | No concerns | Major concerns | Major concerns | Very low |
| Batoclimab_340mg: Rozanolixzumb_10mg_kg | 0 | No concerns | Low risk | No concerns | Major concerns | No concerns | Major concerns | Low |
| Batoclimab_340mg: Rozanolixzumb_7mg_kg | 0 | No concerns | Low risk | No concerns | Major concerns | No concerns | Major concerns | Low |
| Batoclimab_340mg: Zilucoplan | 0 | No concerns | Low risk | No concerns | Some concerns | Some concerns | Major concerns | Moderate |
| Batoclimab_680mg: Belimumab | 0 | No concerns | Low risk | No concerns | No concerns | Major concerns | Major concerns | Very low |
| Batoclimab_680mg: Eculizumab | 0 | No concerns | Low risk | No concerns | No concerns | Major concerns | Major concerns | Very low |
| Batoclimab_680mg: Efgartigimod_10mg_kg | 0 | No concerns | Low risk | No concerns | No concerns | Major concerns | Major concerns | Very low |
| Batoclimab_680mg: Iscalimab | 0 | No concerns | Low risk | No concerns | No concerns | Major concerns | Major concerns | Very low |
| Batoclimab_680mg: Mezagitamab_300mg | 0 | No concerns | Low risk | No concerns | No concerns | Major concerns | Major concerns | Very low |
| Batoclimab_680mg: Mezagitamab_600mg | 0 | No concerns | Low risk | No concerns | No concerns | Major concerns | Major concerns | Very low |
| Batoclimab_680mg: Nipocalimab_30mg_kg | 0 | No concerns | Low risk | No concerns | No concerns | Major concerns | Major concerns | Very low |
| Batoclimab_680mg: Nipocalimab_5mg_kg | 0 | No concerns | Low risk | No concerns | No concerns | Major concerns | Major concerns | Very low |
| Batoclimab_680mg: Nipocalimab_60mg_kg | 0 | No concerns | Low risk | No concerns | No concerns | Major concerns | Major concerns | Very low |
| Batoclimab_680mg: Ravulizumab | 0 | No concerns | Low risk | No concerns | No concerns | Major concerns | Major concerns | Very low |
| Batoclimab_680mg: Rituximab | 0 | No concerns | Low risk | No concerns | No concerns | Major concerns | Major concerns | Very low |
| Batoclimab_680mg: Rozanolixzumb_10mg_kg | 0 | Some concerns | Low risk | No concerns | Major concerns | No concerns | Major concerns | Moderate |
| Batoclimab_680mg: Rozanolixzumb_7mg_kg | 0 | Some concerns | Low risk | No concerns | Some concerns | Some concerns | Major concerns | Low |
| Batoclimab_680mg: Zilucoplan | 0 | No concerns | Low risk | No concerns | No concerns | Major concerns | Major concerns | Very low |
| Belimumab: Eculizumab | 0 | No concerns | Low risk | No concerns | Major concerns | No concerns | Major concerns | Very low |
| Belimumab: Efgartigimod_10mg_kg | 0 | No concerns | Low risk | No concerns | Major concerns | No concerns | Major concerns | Very low |
| Belimumab: Iscalimab | 0 | No concerns | Low risk | No concerns | Major concerns | No concerns | Major concerns | Very low |
| Belimumab: Mezagitamab_300mg | 0 | No concerns | Low risk | No concerns | Major concerns | No concerns | Major concerns | Very low |
| Belimumab: Mezagitamab_600mg | 0 | No concerns | Low risk | No concerns | Major concerns | No concerns | Major concerns | Very low |
| Belimumab: Nipocalimab_30mg_kg | 0 | No concerns | Low risk | No concerns | Major concerns | No concerns | Major concerns | Very low |
| Belimumab: Nipocalimab_5mg_kg | 0 | No concerns | Low risk | No concerns | Major concerns | No concerns | Major concerns | Very low |
| Belimumab: Nipocalimab_60mg_kg | 0 | No concerns | Low risk | No concerns | No concerns | Major concerns | Major concerns | Very low |
| Belimumab: Ravulizumab | 0 | No concerns | Low risk | No concerns | Major concerns | No concerns | Major concerns | Very low |
| Belimumab: Rituximab | 0 | No concerns | Low risk | No concerns | Major concerns | No concerns | Major concerns | Very low |
| Belimumab: Rozanolixzumb_10mg_kg | 0 | Some concerns | Low risk | No concerns | Major concerns | No concerns | Major concerns | Low |
| Belimumab: Rozanolixzumb_7mg_kg | 0 | Some concerns | Low risk | No concerns | Major concerns | No concerns | Major concerns | Low |
| Belimumab: Zilucoplan | 0 | No concerns | Low risk | No concerns | Major concerns | No concerns | Major concerns | Very low |
| Eculizumab: Efgartigimod_10mg_kg | 0 | No concerns | Low risk | No concerns | Some concerns | Some concerns | Major concerns | Low |
| Eculizumab: Iscalimab | 0 | No concerns | Low risk | No concerns | Some concerns | Some concerns | Major concerns | Low |
| Eculizumab: Mezagitamab_300mg | 0 | No concerns | Low risk | No concerns | Major concerns | No concerns | Major concerns | Very low |
| Eculizumab: Mezagitamab_600mg | 0 | No concerns | Low risk | No concerns | No concerns | Major concerns | Major concerns | Very low |
| Eculizumab: Nipocalimab_30mg_kg | 0 | No concerns | Low risk | No concerns | Some concerns | Some concerns | Major concerns | Very low |
| Eculizumab: Nipocalimab_5mg_kg | 0 | No concerns | Low risk | No concerns | No concerns | Major concerns | Major concerns | Very low |
| Eculizumab: Nipocalimab_60mg_kg | 0 | No concerns | Low risk | No concerns | No concerns | Major concerns | Major concerns | Very low |
| Eculizumab: Ravulizumab | 0 | No concerns | Low risk | No concerns | No concerns | Major concerns | Major concerns | Very low |
| Eculizumab: Rituximab | 0 | No concerns | Low risk | No concerns | Major concerns | No concerns | Major concerns | Moderate |
| Eculizumab: Rozanolixzumb_10mg_kg | 0 | Some concerns | Low risk | No concerns | Some concerns | Some concerns | Major concerns | Very low |
| Eculizumab: Rozanolixzumb_7mg_kg | 0 | Some concerns | Low risk | No concerns | Major concerns | No concerns | Major concerns | Low |
| Eculizumab: Zilucoplan | 0 | No concerns | Low risk | No concerns | Major concerns | No concerns | Major concerns | Low |
| Efgartigimod_10mg_kg: Iscalimab | 0 | No concerns | Low risk | No concerns | Major concerns | No concerns | Major concerns | Low |
| Efgartigimod_10mg_kg: Mezagitamab_300mg | 0 | No concerns | Low risk | No concerns | Major concerns | No concerns | Major concerns | Low |
| Efgartigimod_10mg_kg: Mezagitamab_600mg | 0 | No concerns | Low risk | No concerns | Major concerns | No concerns | Major concerns | Low |
| Efgartigimod_10mg_kg: Nipocalimab_30mg_kg | 0 | No concerns | Low risk | No concerns | Major concerns | No concerns | Major concerns | Low |
| Efgartigimod_10mg_kg: Nipocalimab_5mg_kg | 0 | No concerns | Low risk | No concerns | Major concerns | No concerns | Major concerns | Low |
| Efgartigimod_10mg_kg: Nipocalimab_60mg_kg | 0 | No concerns | Low risk | No concerns | No concerns | Major concerns | Major concerns | Very low |
| Efgartigimod_10mg_kg: Ravulizumab | 0 | No concerns | Low risk | No concerns | Major concerns | No concerns | Major concerns | Low |
| Efgartigimod_10mg_kg: Rituximab | 0 | No concerns | Low risk | No concerns | Major concerns | No concerns | Major concerns | Low |
| Efgartigimod_10mg_kg: Rozanolixzumb_10mg_kg | 0 | Some concerns | Low risk | No concerns | No concerns | Major concerns | Major concerns | Very low |
| Efgartigimod_10mg_kg: Rozanolixzumb_7mg_kg | 0 | Some concerns | Low risk | No concerns | Some concerns | Some concerns | Major concerns | Very low |
| Efgartigimod_10mg_kg: Zilucoplan | 0 | No concerns | Low risk | No concerns | Some concerns | Some concerns | Major concerns | Very low |
| Iscalimab: Mezagitamab_300mg | 0 | No concerns | Low risk | No concerns | Major concerns | No concerns | Major concerns | Low |
| Iscalimab: Mezagitamab_600mg | 0 | No concerns | Low risk | No concerns | Major concerns | No concerns | Major concerns | Low |
| Iscalimab: Nipocalimab_30mg_kg | 0 | No concerns | Low risk | No concerns | Major concerns | No concerns | Major concerns | Low |
| Iscalimab: Nipocalimab_5mg_kg | 0 | No concerns | Low risk | No concerns | Major concerns | No concerns | Major concerns | Low |
| Iscalimab: Nipocalimab_60mg_kg | 0 | No concerns | Low risk | No concerns | Some concerns | Some concerns | Major concerns | Very low |
| Iscalimab: Ravulizumab | 0 | No concerns | Low risk | No concerns | Major concerns | No concerns | Major concerns | Low |
| Iscalimab: Rituximab | 0 | No concerns | Low risk | No concerns | Major concerns | No concerns | Major concerns | Low |
| Iscalimab: Rozanolixzumb_10mg_kg | 0 | Some concerns | Low risk | No concerns | No concerns | Major concerns | Major concerns | Very low |
| Iscalimab: Rozanolixzumb_7mg_kg | 0 | Some concerns | Low risk | No concerns | Major concerns | No concerns | Major concerns | Low |
| Iscalimab: Zilucoplan | 0 | No concerns | Low risk | No concerns | Some concerns | Some concerns | Major concerns | Very low |
| Mezagitamab_300mg: Nipocalimab_30mg_kg | 0 | No concerns | Low risk | No concerns | Major concerns | No concerns | Major concerns | Low |
| Mezagitamab_300mg: Nipocalimab_5mg_kg | 0 | No concerns | Low risk | No concerns | Major concerns | No concerns | Major concerns | Low |
| Mezagitamab_300mg: Nipocalimab_60mg_kg | 0 | No concerns | Low risk | No concerns | No concerns | Major concerns | Major concerns | Very low |
| Mezagitamab_300mg: Ravulizumab | 0 | No concerns | Low risk | No concerns | Major concerns | No concerns | Major concerns | Low |
| Mezagitamab_300mg: Rituximab | 0 | No concerns | Low risk | No concerns | Major concerns | No concerns | Major concerns | Low |
| Mezagitamab_300mg: Rozanolixzumb_10mg_kg | 0 | Some concerns | Low risk | No concerns | Major concerns | No concerns | Major concerns | Very low |
| Mezagitamab_300mg: Rozanolixzumb_7mg_kg | 0 | Some concerns | Low risk | No concerns | Major concerns | No concerns | Major concerns | Very low |
| Mezagitamab_300mg: Zilucoplan | 0 | No concerns | Low risk | No concerns | Major concerns | No concerns | Major concerns | Low |
| Mezagitamab_600mg: Nipocalimab_30mg_kg | 0 | No concerns | Low risk | No concerns | Major concerns | No concerns | Major concerns | Low |
| Mezagitamab_600mg: Nipocalimab_5mg_kg | 0 | No concerns | Low risk | No concerns | Major concerns | No concerns | Major concerns | Low |
| Mezagitamab_600mg: Nipocalimab_60mg_kg | 0 | No concerns | Low risk | No concerns | Major concerns | No concerns | Major concerns | Low |
| Mezagitamab_600mg: Ravulizumab | 0 | No concerns | Low risk | No concerns | Some concerns | Some concerns | Major concerns | Very low |
| Mezagitamab_600mg: Rituximab | 0 | No concerns | Low risk | No concerns | Major concerns | No concerns | Major concerns | Low |
| Mezagitamab_600mg: Rozanolixzumb_10mg_kg | 0 | No concerns | Low risk | No concerns | No concerns | Major concerns | Major concerns | Very low |
| Mezagitamab_600mg: Rozanolixzumb_7mg_kg | 0 | No concerns | Low risk | No concerns | No concerns | Major concerns | Major concerns | Very low |
| Mezagitamab_600mg: Zilucoplan | 0 | No concerns | Low risk | No concerns | No concerns | Major concerns | Major concerns | Very low |
| Nipocalimab_30mg_kg: Ravulizumab | 0 | No concerns | Low risk | No concerns | Major concerns | No concerns | Major concerns | Low |
| Nipocalimab_30mg_kg: Rituximab | 0 | No concerns | Low risk | No concerns | Major concerns | No concerns | Major concerns | Low |
| Nipocalimab_30mg_kg: Rozanolixzumb_10mg_kg | 0 | No concerns | Low risk | No concerns | No concerns | Major concerns | Major concerns | Very low |
| Nipocalimab_30mg_kg: Rozanolixzumb_7mg_kg | 0 | No concerns | Low risk | No concerns | Some concerns | Some concerns | Major concerns | Very low |
| Nipocalimab_30mg_kg: Zilucoplan | 0 | No concerns | Low risk | No concerns | Some concerns | Some concerns | Major concerns | Very low |
| Nipocalimab_5mg_kg: Ravulizumab | 0 | No concerns | Low risk | No concerns | Some concerns | Some concerns | Major concerns | Very low |
| Nipocalimab_5mg_kg: Rituximab | 0 | No concerns | Low risk | No concerns | Major concerns | No concerns | Major concerns | Low |
| Nipocalimab_5mg_kg: Rozanolixzumb_10mg_kg | 0 | No concerns | Low risk | No concerns | No concerns | Major concerns | Major concerns | Very low |
| Ravulizumab: Rituximab | 0 | No concerns | Low risk | No concerns | No concerns | Major concerns | Major concerns | Very low |
| Ravulizumab: Rozanolixzumb_10mg_kg | 0 | No concerns | Low risk | No concerns | No concerns | Major concerns | Major concerns | Very low |
| Ravulizumab: Rozanolixzumb_7mg_kg | 0 | No concerns | Low risk | No concerns | No concerns | Major concerns | Major concerns | Very low |
| Ravulizumab: Zilucoplan | 0 | No concerns | Low risk | No concerns | No concerns | Major concerns | Major concerns | Very low |
| Rituximab: Rozanolixzumb_10mg_kg | 0 | No concerns | Low risk | No concerns | No concerns | Major concerns | Major concerns | Very low |
| Rituximab: Rozanolixzumb_7mg_kg | 0 | No concerns | Low risk | No concerns | No concerns | Major concerns | Major concerns | Very low |
| Rituximab: Zilucoplan | 0 | No concerns | Low risk | No concerns | No concerns | Major concerns | Major concerns | Very low |
| Rozanolixzumb_10mg_kg: Zilucoplan | 0 | No concerns | Low risk | No concerns | Major concerns | No concerns | Major concerns | Low |
| Rozanolixzumb_7mg_kg: Zilucoplan | 0 | Some concerns | Low risk | No concerns | No concerns | Major concerns | Major concerns | Very low |
| **2. QMG** | | | | | | | | |
| Batoclimab_340mg: Batoclimab_680mg | 1 | No concerns | Low risk | No concerns | Some concerns | Some concerns | Major concerns | High |
| Batoclimab_340mg: Placebo | 1 | No concerns | Low risk | No concerns | No concerns | Major concerns | Major concerns | Low |
| Batoclimab_680mg: Placebo | 1 | No concerns | Low risk | No concerns | No concerns | Major concerns | Major concerns | Low |
| Belimumab: Placebo | 1 | No concerns | Low risk | No concerns | Some concerns | Some concerns | Major concerns | High |
| Eculizumab: Placebo | 1 | No concerns | Low risk | No concerns | No concerns | Some concerns | Major concerns | High |
| Efgartigimod_10mg_kg: Placebo | 2 | No concerns | Low risk | No concerns | No concerns | Major concerns | Major concerns | Low |
| Iscalimab: Placebo | 1 | No concerns | Low risk | No concerns | Some concerns | Some concerns | Major concerns | Moderate |
| Mezagitamab_300mg: Mezagitamab_600mg | 1 | No concerns | Low risk | No concerns | Some concerns | Some concerns | Major concerns | Moderate |
| Mezagitamab_300mg: Placebo | 1 | No concerns | Low risk | No concerns | Some concerns | Some concerns | Major concerns | Moderate |
| Mezagitamab_600mg: Placebo | 1 | No concerns | Low risk | No concerns | Some concerns | Some concerns | Major concerns | Moderate |
| Nipocalimab_30mg_kg: Nipocalimab_5mg_kg | 1 | No concerns | Low risk | No concerns | Some concerns | Some concerns | Major concerns | Moderate |
| Nipocalimab_30mg_kg: Nipocalimab_60mg_kg | 1 | No concerns | Low risk | No concerns | No concerns | Major concerns | Major concerns | Low |
| Nipocalimab_30mg_kg: Placebo | 1 | No concerns | Low risk | No concerns | Some concerns | Some concerns | Major concerns | Moderate |
| Nipocalimab_5mg_kg: Nipocalimab_60mg_kg | 1 | No concerns | Low risk | No concerns | Some concerns | Some concerns | Major concerns | Moderate |
| Nipocalimab_5mg_kg: Placebo | 1 | No concerns | Low risk | No concerns | No concerns | Major concerns | Major concerns | Low |
| Nipocalimab_60mg_kg: Placebo | 1 | No concerns | Low risk | No concerns | Some concerns | Some concerns | Major concerns | Moderate |
| Placebo: Ravulizumab | 1 | No concerns | Low risk | No concerns | No concerns | No concerns | Major concerns | High |
| Placebo: Rituximab | 2 | No concerns | Low risk | No concerns | Some concerns | Some concerns | Major concerns | High |
| Placebo: Rozanolixzumb_10mg_kg | 1 | Some concerns | Low risk | No concerns | No concerns | Major concerns | Major concerns | Low |
| Placebo: Rozanolixzumb_7mg_kg | 1 | Some concerns | Low risk | No concerns | No concerns | Major concerns | Major concerns | Low |
| Placebo: Zilucoplan | 1 | No concerns | Low risk | No concerns | No concerns | Some concerns | Major concerns | Moderate |
| Rozanolixzumb_10mg_kg: Rozanolixzumb_7mg_kg | 1 | Some concerns | Low risk | No concerns | Some concerns | Some concerns | Major concerns | Low |
| Batoclimab_340mg: Belimumab 10mg/kg | 0 | No concerns | Low risk | No concerns | Some concerns | Some concerns | Major concerns | Moderate |
| Batoclimab_340mg: Eculizumab | 0 | No concerns | Low risk | No concerns | Some concerns | Some concerns | Major concerns | Moderate |
| Batoclimab_340mg: Efgartigimod_10mg_kg | 0 | No concerns | Low risk | No concerns | No concerns | Major concerns | Major concerns | Very low |
| Batoclimab_340mg: Iscalimab | 0 | No concerns | Low risk | No concerns | No concerns | Major concerns | Major concerns | Very low |
| Batoclimab_340mg: Mezagitamab_300mg | 0 | No concerns | Low risk | No concerns | Some concerns | Some concerns | Major concerns | Moderate |
| Batoclimab_340mg: Mezagitamab_600mg | 0 | No concerns | Low risk | No concerns | No concerns | Major concerns | Major concerns | Very low |
| Batoclimab_340mg: Nipocalimab_30mg/kg | 0 | No concerns | Low risk | No concerns | No concerns | Major concerns | Major concerns | Very low |
| Batoclimab_340mg: Nipocalimab_5mg/kg | 0 | No concerns | Low risk | No concerns | No concerns | Major concerns | Major concerns | Very low |
| Batoclimab_340mg: Nipocalimab_60mg/kg | 0 | No concerns | Low risk | No concerns | No concerns | Major concerns | Major concerns | Very low |
| Batoclimab_340mg: Ravulizumab | 0 | No concerns | Low risk | No concerns | No concerns | Major concerns | Major concerns | Very low |
| Batoclimab_340mg: Rituximab | 0 | No concerns | Low risk | No concerns | No concerns | Major concerns | Major concerns | Very low |
| Batoclimab_340mg: Rozanolixzumb_10mg_kg | 0 | No concerns | Low risk | No concerns | Some concerns | Some concerns | Major concerns | Moderate |
| Batoclimab_340mg: Rozanolixzumb_7mg_kg | 0 | No concerns | Low risk | No concerns | Some concerns | Some concerns | Major concerns | Moderate |
| Batoclimab_340mg: Zilucoplan | 0 | No concerns | Low risk | No concerns | Some concerns | Some concerns | Major concerns | Moderate |
| Batoclimab_680mg: Belimumab | 0 | No concerns | Low risk | No concerns | No concerns | Major concerns | Major concerns | Very low |
| Batoclimab_680mg: Eculizumab | 0 | No concerns | Low risk | No concerns | No concerns | Major concerns | Major concerns | Very low |
| Batoclimab_680mg: Efgartigimod_10mg_kg | 0 | No concerns | Low risk | No concerns | No concerns | Major concerns | Major concerns | Very low |
| Batoclimab_680mg: Iscalimab | 0 | No concerns | Low risk | No concerns | No concerns | Major concerns | Major concerns | Very low |
| Batoclimab_680mg: Mezagitamab_300mg | 0 | No concerns | Low risk | No concerns | No concerns | Major concerns | Major concerns | Very low |
| Batoclimab_680mg: Mezagitamab_600mg | 0 | No concerns | Low risk | No concerns | No concerns | Major concerns | Major concerns | Very low |
| Batoclimab_680mg: Nipocalimab_30mg/kg | 0 | No concerns | Low risk | No concerns | No concerns | Major concerns | Major concerns | Very low |
| Batoclimab_680mg: Nipocalimab_5mg/kg | 0 | No concerns | Low risk | No concerns | No concerns | Major concerns | Major concerns | Very low |
| Batoclimab_680mg: Nipocalimab_60mg/kg | 0 | No concerns | Low risk | No concerns | No concerns | Major concerns | Major concerns | Very low |
| Batoclimab_680mg: Ravulizumab | 0 | No concerns | Low risk | No concerns | No concerns | Major concerns | Major concerns | Very low |
| Batoclimab_680mg: Rituximab | 0 | No concerns | Low risk | No concerns | No concerns | Major concerns | Major concerns | Very low |
| Batoclimab_680mg: Rozanolixzumb_10mg_kg | 0 | Some concerns | Low risk | No concerns | Some concerns | Some concerns | Major concerns | Low |
| Batoclimab_680mg: Rozanolixzumb_7mg_kg | 0 | Some concerns | Low risk | No concerns | Some concerns | Some concerns | Major concerns | Low |
| Batoclimab_680mg: Zilucoplan | 0 | No concerns | Low risk | No concerns | No concerns | Major concerns | Major concerns | Very low |
| Belimumab: Eculizumab | 0 | No concerns | Low risk | No concerns | Some concerns | Some concerns | Major concerns | Moderate |
| Belimumab: Efgartigimod 10mg_kg | 0 | No concerns | Low risk | No concerns | Major concerns | No concerns | Major concerns | Low |
| Belimumab: Iscalimab10mg_kg | 0 | No concerns | Low risk | No concerns | Major concerns | No concerns | Major concerns | Low |
| Belimumab: Mezagitamab 300mg | 0 | No concerns | Low risk | No concerns | Major concerns | No concerns | Major concerns | Low |
| Belimumab: Mezagitamab 600mg | 0 | No concerns | Low risk | No concerns | Some concerns | Some concerns | Major concerns | Low |
| Belimumab: Nipocalimab 30mg_kg | 0 | No concerns | Low risk | No concerns | Some concerns | Some concerns | Major concerns | Low |
| Belimumab: Nipocalimab 5mg_kg | 0 | No concerns | Low risk | No concerns | Some concerns | Some concerns | Major concerns | Low |
| Belimumab: Nipocalimab 60mg_kg | 0 | No concerns | Low risk | No concerns | No concerns | Major concerns | Major concerns | Very low |
| Belimumab: Ravulizumab | 0 | No concerns | Low risk | No concerns | Some concerns | Some concerns | Major concerns | Low |
| Belimumab: Rituximab | 0 | No concerns | Low risk | No concerns | Major concerns | No concerns | Major concerns | Very low |
| Belimumab: Rozanolixzumb 10mg_kg | 0 | Some concerns | Low risk | No concerns | Some concerns | Some concerns | Major concerns | Low |
| Belimumab: Rozanolixzumb 7mg_kg | 0 | Some concerns | Low risk | No concerns | Some concerns | Some concerns | Major concerns | Low |
| Belimumab: Zilucoplan | 0 | No concerns | Low risk | No concerns | Some concerns | Some concerns | Major concerns | Low |
| Eculizumab: Efgartigimod 10mg_kg | 0 | No concerns | Low risk | No concerns | No concerns | Major concerns | Major concerns | Very low |
| Eculizumab: Iscalimab | 0 | No concerns | Low risk | No concerns | Some concerns | Some concerns | Major concerns | Low |
| Eculizumab: Mezagitamab_300mg | 0 | No concerns | Low risk | No concerns | Some concerns | Some concerns | Major concerns | Low |
| Eculizumab: Mezagitamab_600mg | 0 | No concerns | Low risk | No concerns | No concerns | Major concerns | Major concerns | Very low |
| Eculizumab: Nipocalimab_30mg/kg | 0 | No concerns | Low risk | No concerns | Some concerns | Some concerns | Major concerns | Low |
| Eculizumab: Nipocalimab_5mg/kg | 0 | No concerns | Low risk | No concerns | No concerns | Major concerns | Major concerns | Very low |
| Eculizumab: Nipocalimab_60mg/kg | 0 | No concerns | Low risk | No concerns | No concerns | Major concerns | Major concerns | Very low |
| Eculizumab: Ravulizumab | 0 | No concerns | Low risk | No concerns | No concerns | Major concerns | Major concerns | Very low |
| Eculizumab: Rituximab | 0 | No concerns | Low risk | No concerns | Some concerns | Some concerns | Major concerns | Low |
| Eculizumab: Rozanolixzumb_10mg_kg | 0 | Some concerns | Low risk | No concerns | Some concerns | Some concerns | Major concerns | Low |
| Eculizumab: Rozanolixzumb_7mg_kg | 0 | Some concerns | Low risk | No concerns | No concerns | Major concerns | Major concerns | Very low |
| Eculizumab: Zilucoplan | 0 | No concerns | Low risk | No concerns | No concerns | Major concerns | Major concerns | Very low |
| Efgartigimod 10mg_kg: Iscalimab | 0 | No concerns | Low risk | No concerns | Some concerns | Some concerns | Major concerns | Low |
| Efgartigimod_10mg_kg: Mezagitamab_300mg | 0 | No concerns | Low risk | No concerns | Some concerns | Some concerns | Major concerns | Low |
| Efgartigimod_10mg_kg: Mezagitamab_600mg | 0 | No concerns | Low risk | No concerns | Some concerns | Some concerns | Major concerns | Low |
| Efgartigimod_10mg_kg: Nipocalimab_30mg/kg | 0 | No concerns | Low risk | No concerns | Some concerns | Some concerns | Major concerns | Low |
| Efgartigimod_10mg_kg: Nipocalimab_5mg/kg | 0 | No concerns | Low risk | No concerns | Some concerns | Some concerns | Major concerns | Low |
| Efgartigimod_10mg_kg: Nipocalimab_60mg/kg | 0 | No concerns | Low risk | No concerns | No concerns | Major concerns | Major concerns | Very low |
| Efgartigimod_10mg_kg: Ravulizumab | 0 | No concerns | Low risk | No concerns | No concerns | Major concerns | Major concerns | Very low |
| Efgartigimod_10mg_kg: Rituximab | 0 | No concerns | Low risk | No concerns | No concerns | Major concerns | Major concerns | Very low |
| Efgartigimod_10mg_kg: Rozanolixzumb_10mg_kg | 0 | Some concerns | Low risk | No concerns | No concerns | Major concerns | Major concerns | Very low |
| Efgartigimod_10mg_kg: Rozanolixzumb_7mg_kg | 0 | Some concerns | Low risk | No concerns | Some concerns | Some concerns | Major concerns | Low |
| Efgartigimod_10mg_kg: Zilucoplan | 0 | No concerns | Low risk | No concerns | No concerns | Major concerns | Major concerns | Very low |
| Iscalimab: Mezagitamab_300mg | 0 | No concerns | Low risk | No concerns | Some concerns | Some concerns | Major concerns | Low |
| Iscalimab: Mezagitamab_600mg | 0 | No concerns | Low risk | No concerns | Some concerns | Some concerns | Major concerns | Low |
| Iscalimab: Nipocalimab_30mg/kg | 0 | No concerns | Low risk | No concerns | Some concerns | Some concerns | Major concerns | Low |
| Iscalimab: Nipocalimab_5mg/kg | 0 | No concerns | Low risk | No concerns | Some concerns | Some concerns | Major concerns | Low |
| Iscalimab: Nipocalimab_60mg/kg | 0 | No concerns | Low risk | No concerns | Some concerns | Some concerns | Major concerns | Low |
| Iscalimab: Ravulizumab | 0 | No concerns | Low risk | No concerns | Some concerns | Some concerns | Major concerns | Low |
| Iscalimab: Rituximab | 0 | No concerns | Low risk | No concerns | Some concerns | Some concerns | Major concerns | Low |
| Iscalimab: Rozanolixzumb_10mg_kg | 0 | Some concerns | Low risk | No concerns | No concerns | Major concerns | Major concerns | Very low |
| Iscalimab: Rozanolixzumb_7mg_kg | 0 | Some concerns | Low risk | No concerns | Some concerns | Some concerns | Major concerns | Low |
| Iscalimab: Zilucoplan | 0 | No concerns | Low risk | No concerns | Some concerns | Some concerns | Major concerns | Low |
| Mezagitamab_300mg: Nipocalimab_30mg/kg | 0 | No concerns | Low risk | No concerns | Some concerns | Some concerns | Major concerns | Low |
| Mezagitamab_300mg: Nipocalimab_5mg/kg | 0 | No concerns | Low risk | No concerns | Some concerns | Some concerns | Major concerns | Low |
| Mezagitamab_300mg: Nipocalimab_60mg/kg | 0 | No concerns | Low risk | No concerns | No concerns | Major concerns | Major concerns | Very low |
| Mezagitamab_300mg: Ravulizumab | 0 | No concerns | Low risk | No concerns | Some concerns | Some concerns | Major concerns | Low |
| Mezagitamab_300mg: Rituximab | 0 | No concerns | Low risk | No concerns | Major concerns | No concerns | Major concerns | Moderate |
| Mezagitamab_300mg: Rozanolixzumb_10mg_kg | 0 | Some concerns | Low risk | No concerns | Some concerns | Some concerns | Major concerns | Low |
| Mezagitamab_300mg: Rozanolixzumb_7mg_kg | 0 | Some concerns | Low risk | No concerns | Some concerns | Some concerns | Major concerns | Low |
| Mezagitamab_300mg: Zilucoplan | 0 | No concerns | Low risk | No concerns | Some concerns | Some concerns | Major concerns | Low |
| Mezagitamab_600mg: Nipocalimab_30mg/kg | 0 | No concerns | Low risk | No concerns | Major concerns | No concerns | Major concerns | Very low |
| Mezagitamab_600mg: Nipocalimab_5mg/kg | 0 | No concerns | Low risk | No concerns | Major concerns | No concerns | Major concerns | Very low |
| Mezagitamab_600mg: Nipocalimab_60mg/kg | 0 | No concerns | Low risk | No concerns | Some concerns | Some concerns | Major concerns | Low |
| Mezagitamab_600mg: Ravulizumab | 0 | No concerns | Low risk | No concerns | Some concerns | Some concerns | Major concerns | Low |
| Mezagitamab_600mg: Rituximab | 0 | No concerns | Low risk | No concerns | Some concerns | Some concerns | Major concerns | Low |
| Mezagitamab_600mg: Rozanolixzumb_10mg_kg | 0 | No concerns | Low risk | No concerns | No concerns | Major concerns | Major concerns | Low |
| Mezagitamab_600mg: Rozanolixzumb_7mg_kg | 0 | No concerns | Low risk | No concerns | No concerns | Major concerns | Major concerns | Low |
| Mezagitamab_600mg: Zilucoplan | 0 | No concerns | Low risk | No concerns | No concerns | Major concerns | Major concerns | Low |
| Nipocalimab_30mg/kg: Ravulizumab | 0 | No concerns | Low risk | No concerns | Some concerns | Some concerns | Major concerns | Low |
| Nipocalimab_30mg/kg: Rituximab | 0 | No concerns | Low risk | No concerns | Some concerns | Some concerns | Major concerns | Low |
| Nipocalimab_30mg/kg: Rozanolixzumb_10mg_kg | 0 | No concerns | Low risk | No concerns | No concerns | Major concerns | Major concerns | Very low |
| Nipocalimab_30mg/kg: Rozanolixzumb_7mg_kg | 0 | No concerns | Low risk | No concerns | Some concerns | Some concerns | Major concerns | Low |
| Nipocalimab_30mg/kg: Zilucoplan | 0 | No concerns | Low risk | No concerns | Some concerns | Some concerns | Major concerns | Low |
| Nipocalimab_5mg/kg: Ravulizumab | 0 | No concerns | Low risk | No concerns | Some concerns | Some concerns | Major concerns | Low |
| Nipocalimab_5mg/kg: Rituximab | 0 | No concerns | Low risk | No concerns | Some concerns | Some concerns | Major concerns | Low |
| Nipocalimab_5mg/kg: Rozanolixzumb_10mg_kg | 0 | No concerns | Low risk | No concerns | No concerns | Major concerns | Major concerns | Very low |
| Nipocalimab_5mg/kg: Rozanolixzumb_7mg_kg | 0 | No concerns | Low risk | No concerns | No concerns | Major concerns | Major concerns | Very low |
| Nipocalimab_5mg/kg: Zilucoplan | 0 | No concerns | Low risk | No concerns | No concerns | Major concerns | Major concerns | Very low |
| Nipocalimab_60mg/kg: Ravulizumab | 0 | No concerns | Low risk | No concerns | No concerns | Major concerns | Major concerns | Very low |
| Nipocalimab_60mg/kg: Rituximab | 0 | No concerns | Low risk | No concerns | No concerns | Major concerns | Major concerns | Very low |
| Nipocalimab_60mg/kg: Rozanolixzumb_10mg_kg | 0 | No concerns | Low risk | No concerns | No concerns | Major concerns | Major concerns | Very low |
| Nipocalimab_60mg/kg: Rozanolixzumb_7mg_kg | 0 | No concerns | Low risk | No concerns | No concerns | Major concerns | Major concerns | Very low |
| Nipocalimab_60mg/kg: Zilucoplan | 0 | No concerns | Low risk | No concerns | No concerns | Major concerns | Major concerns | Very low |
| Ravulizumab: Rituximab | 0 | No concerns | Low risk | No concerns | No concerns | Major concerns | Major concerns | Very low |
| Ravulizumab: Rozanolixzumb_10mg_kg | 0 | Some concerns | Low risk | No concerns | No concerns | Major concerns | Major concerns | Very low |
| Ravulizumab: Rozanolixzumb_7mg_kg | 0 | Some concerns | Low risk | No concerns | Some concerns | Some concerns | Major concerns | Low |
| Ravulizumab: Zilucoplan | 0 | No concerns | Low risk | No concerns | No concerns | Some concerns | Major concerns | Moderate |
| Rituximab: Rozanolixzumb_10mg_kg | 0 | Some concerns | Low risk | No concerns | Some concerns | Some concerns | Major concerns | Low |
| Rituximab: Rozanolixzumb_7mg_kg | 0 | Some concerns | Low risk | No concerns | Some concerns | Some concerns | Major concerns | Low |
| Rituximab: Zilucoplan | 0 | No concerns | Low risk | No concerns | Some concerns | Some concerns | Major concerns | Moderate |
| Rozanolixzumb_10mg_kg: Zilucoplan | 0 | Some concerns | Low risk | No concerns | Some concerns | Some concerns | Major concerns | Low |
| Rozanolixzumb_7mg_kg: Zilucoplan | 0 | Some concerns | Low risk | No concerns | No concerns | Major concerns | Major concerns | Very low |
| **3. MGC** |  |  |  |  |  |  |  |  |
| Batoclimab_340mg: Batoclimab_680mg | 1 | No concerns | Low risk | No concerns | Some concerns | Some concerns | Major concerns | Moderate |
| Batoclimab_340mg: Placebo | 1 | No concerns | Low risk | No concerns | No concerns | Major concerns | Major concerns | Low |
| Batoclimab_680mg: Placebo | 1 | No concerns | Low risk | No concerns | No concerns | Major concerns | Major concerns | Low |
| Belimumab: Placebo | 1 | No concerns | Low risk | No concerns | Some concerns | Some concerns | Major concerns | High |
| Eculizumab: Placebo | 1 | No concerns | Low risk | No concerns | No concerns | Some concerns | Major concerns | High |
| Efgartigimod_10mg_kg: Placebo | 2 | No concerns | Low risk | No concerns | No concerns | Major concerns | Major concerns | Low |
| Iscalimab: Placebo | 1 | No concerns | Low risk | No concerns | Some concerns | Some concerns | Major concerns | Moderate |
| Mezagitamab_300mg: Mezagitamab_600mg | 1 | No concerns | Low risk | No concerns | Some concerns | Some concerns | Major concerns | Moderate |
| Mezagitamab_300mg: Placebo | 1 | No concerns | Low risk | No concerns | Some concerns | Some concerns | Major concerns | Moderate |
| Mezagitamab_600mg: Placebo | 1 | No concerns | Low risk | No concerns | Some concerns | Some concerns | Major concerns | Moderate |
| Placebo: Rituximab | 1 | No concerns | Low risk | No concerns | Some concerns | Some concerns | Major concerns | Moderate |
| Placebo: Rozanolixzumb_10mg_kg | 1 | Some concerns | Low risk | No concerns | No concerns | Major concerns | Major concerns | Low |
| Placebo: Rozanolixzumb_7mg_kg | 1 | Some concerns | Low risk | No concerns | Some concerns | Some concerns | Major concerns | Moderate |
| Placebo: Zilucoplan | 1 | No concerns | Low risk | No concerns | Some concerns | Some concerns | Major concerns | Moderate |
| Rozanolixzumb_10mg_kg: Rozanolixzumb_7mg_kg | 1 | Some concerns | Low risk | No concerns | No concerns | Major concerns | Major concerns | Low |
| Batoclimab_340mg: Belimumab | 0 | No concerns | Low risk | No concerns | Some concerns | Some concerns | Major concerns | High |
| Batoclimab_340mg: Eculizumab | 0 | No concerns | Low risk | No concerns | Some concerns | No concerns | Major concerns | Very low |
| Batoclimab_340mg: Efgartigimod_10mg_kg | 0 | No concerns | Low risk | No concerns | Some concerns | No concerns | Major concerns | Very low |
| Batoclimab_340mg: Iscalimab | 0 | No concerns | Low risk | No concerns | Some concerns | No concerns | Major concerns | Very low |
| Batoclimab_340mg: Mezagitamab_300mg | 0 | No concerns | Low risk | No concerns | Some concerns | No concerns | Major concerns | Very low |
| Batoclimab_340mg: Mezagitamab_600mg | 0 | No concerns | Low risk | No concerns | Some concerns | No concerns | Major concerns | Very low |
| Batoclimab_340mg: Rituximab | 0 | No concerns | Low risk | No concerns | Major concerns | No concerns | Major concerns | Low |
| Batoclimab_340mg: Rozanolixzumb_10mg_kg | 0 | Some concerns | Low risk | No concerns | Some concerns | No concerns | Major concerns | Very low |
| Batoclimab_340mg: Rozanolixzumb_7mg_kg | 0 | No concerns | Low risk | No concerns | Some concerns | No concerns | Major concerns | low |
| Batoclimab_340mg: Zilucoplan | 0 | No concerns | Low risk | No concerns | Some concerns | Some concerns | Major concerns | Very low |
| Batoclimab_680mg: Belimumab | 0 | No concerns | Low risk | No concerns | Some concerns | No concerns | Major concerns | Very low |
| Batoclimab_680mg: Eculizumab | 0 | No concerns | Low risk | No concerns | Some concerns | No concerns | Major concerns | Very low |
| Batoclimab_680mg: Efgartigimod_10mg_kg | 0 | No concerns | Low risk | No concerns | Some concerns | No concerns | Major concerns | Very low |
| Batoclimab_680mg: Iscalimab | 0 | No concerns | Low risk | No concerns | Some concerns | No concerns | Major concerns | Very low |
| Batoclimab_680mg: Mezagitamab_300mg | 0 | No concerns | Low risk | No concerns | Major concerns | No concerns | Major concerns | Very low |
| Batoclimab_680mg: Mezagitamab_600mg | 0 | No concerns | Low risk | No concerns | Major concerns | No concerns | Major concerns | Very low |
| Batoclimab_680mg: Rituximab | 0 | No concerns | Low risk | No concerns | Some concerns | No concerns | Major concerns | Very low |
| Batoclimab_680mg: Rozanolixzumb_10mg_kg | 0 | Some concerns | Low risk | No concerns | Some concerns | Some concerns | Major concerns | Very low |
| Batoclimab_680mg: Rozanolixzumb_7mg_kg | 0 | Some concerns | Low risk | No concerns | Some concerns | Some concerns | Major concerns | Very low |
| Batoclimab_680mg: Zilucoplan | 0 | No concerns | Low risk | No concerns | Some concerns | No concerns | Major concerns | Very low |
| Belimumab: Eculizumab | 0 | No concerns | Low risk | No concerns | Some concerns | No concerns | Major concerns | Very low |
| Belimumab: Efgartigimod_10mg_kg | 0 | No concerns | Low risk | No concerns | Some concerns | No concerns | Major concerns | Very low |
| Belimumab: Iscalimab | 0 | No concerns | Low risk | No concerns | Major concerns | Some concerns | Major concerns | Very low |
| Belimumab: Mezagitamab_300mg | 0 | No concerns | Low risk | No concerns | Some concerns | Some concerns | Major concerns | Very low |
| Belimumab: Mezagitamab_600mg | 0 | No concerns | Low risk | No concerns | Some concerns | Some concerns | Major concerns | Very low |
| Belimumab: Rituximab | 0 | No concerns | Low risk | No concerns | Some concerns | No concerns | Major concerns | Very low |
| Belimumab: Rozanolixzumb_10mg_kg | 0 | Some concerns | Low risk | No concerns | Some concerns | No concerns | Major concerns | Very low |
| Belimumab: Rozanolixzumb_7mg_kg | 0 | Some concerns | Low risk | No concerns | Some concerns | No concerns | Major concerns | Very low |
| Belimumab: Zilucoplan | 0 | No concerns | Low risk | No concerns | Some concerns | No concerns | Major concerns | Very low |
| Eculizumab: Efgartigimod_10mg_kg | 0 | No concerns | Low risk | No concerns | Some concerns | No concerns | Major concerns | Very low |
| Eculizumab: Iscalimab | 0 | No concerns | Low risk | No concerns | Some concerns | No concerns | Major concerns | Very low |
| Eculizumab: Mezagitamab_300mg | 0 | No concerns | Low risk | No concerns | Major concerns | Some concerns | Major concerns | Very low |
| Eculizumab: Mezagitamab_600mg | 0 | No concerns | Low risk | No concerns | Major concerns | Some concerns | Major concerns | Very low |
| Eculizumab: Rituximab | 0 | No concerns | Low risk | No concerns | Some concerns | No concerns | Major concerns | Very low |
| Eculizumab: Rozanolixzumb_10mg_kg | 0 | Some concerns | Low risk | No concerns | Some concerns | No concerns | Major concerns | Very low |
| Eculizumab: Rozanolixzumb_7mg_kg | 0 | Some concerns | Low risk | No concerns | Some concerns | No concerns | Major concerns | Very low |
| Eculizumab: Zilucoplan | 0 | No concerns | Low risk | No concerns | Some concerns | No concerns | Major concerns | Very low |
| Efgartigimod_10mg_kg: Iscalimab | 0 | No concerns | Low risk | No concerns | Some concerns | Some concerns | Major concerns | Very low |
| Efgartigimod_10mg_kg: Mezagitamab_300mg | 0 | No concerns | Low risk | No concerns | Some concerns | Major concerns | Major concerns | Very low |
| Efgartigimod_10mg_kg: Mezagitamab_600mg | 0 | No concerns | Low risk | No concerns | Some concerns | Some concerns | Major concerns | Very low |
| Efgartigimod_10mg_kg: Rituximab | 0 | No concerns | Low risk | No concerns | Some concerns | No concerns | Major concerns | Very low |
| Efgartigimod_10mg_kg: Rozanolixzumb_10mg_kg | 0 | Some concerns | Low risk | No concerns | Some concerns | Some concerns | Major concerns | Very low |
| Efgartigimod_10mg_kg: Rozanolixzumb_7mg_kg | 0 | Some concerns | Low risk | No concerns | No concerns | No concerns | Major concerns | Very low |
| Efgartigimod_10mg_kg: Zilucoplan | 0 | No concerns | Low risk | No concerns | No concerns | Major concerns | Major concerns | Very low |
| Iscalimab: Mezagitamab_300mg | 0 | No concerns | Low risk | No concerns | No concerns | Major concerns | Major concerns | Very low |
| Iscalimab: Mezagitamab_600mg | 0 | No concerns | Low risk | No concerns | No concerns | Major concerns | Major concerns | Very low |
| Iscalimab: Rituximab | 0 | No concerns | Low risk | No concerns | Some concerns | Some concerns | Major concerns | Very low |
| Iscalimab: Rozanolixzumb_10mg_kg | 0 | Some concerns | Low risk | No concerns | No concerns | Major concerns | Major concerns | Very low |
| Iscalimab: Rozanolixzumb_7mg_kg | 0 | Some concerns | Low risk | No concerns | Some concerns | Some concerns | Major concerns | Very low |
| Iscalimab: Zilucoplan | 0 | No concerns | Low risk | No concerns | Some concerns | Some concerns | Major concerns | Very low |
| Mezagitamab_300mg: Rituximab | 0 | No concerns | Low risk | No concerns | Some concerns | Some concerns | Major concerns | Very low |
| Mezagitamab_300mg: Rozanolixzumb_10mg_kg | 0 | Some concerns | Low risk | No concerns | Some concerns | Some concerns | Major concerns | Very low |
| Mezagitamab_300mg: Rozanolixzumb_7mg_kg | 0 | Some concerns | Low risk | No concerns | Major concerns | No concerns | Major concerns | Very low |
| Mezagitamab_300mg: Zilucoplan | 0 | No concerns | Low risk | No concerns | Major concerns | No concerns | Major concerns | Very low |
| Mezagitamab_600mg: Rituximab | 0 | No concerns | Low risk | No concerns | Major concerns | No concerns | Major concerns | Very low |
| Mezagitamab_600mg: Rozanolixzumb_10mg_kg | 0 | No concerns | Low risk | No concerns | Some concerns | Some concerns | Major concerns | Very low |
| Mezagitamab_600mg: Rozanolixzumb_7mg_kg | 0 | No concerns | Low risk | No concerns | Some concerns | Some concerns | Major concerns | Very low |
| Mezagitamab_600mg: Zilucoplan | 0 | No concerns | Low risk | No concerns | Major concerns | No concerns | Major concerns | Very low |
| Rituximab: Rozanolixzumb_10mg_kg | 0 | Some concerns | Low risk | No concerns | Some concerns | Some concerns | Major concerns | Very low |
| Rituximab: Rozanolixzumb_7mg_kg | 0 | Some concerns | Low risk | No concerns | Some concerns | Some concerns | Major concerns | Very low |
| Rituximab: Zilucoplan | 0 | No concerns | Low risk | No concerns | Some concerns | Some concerns | Major concerns | Very low |
| Rozanolixzumb_10mg_kg: Zilucoplan | 0 | Some concerns | Low risk | No concerns | Major concerns | No concerns | Major concerns | Very low |
| Rozanolixzumb_7mg_kg: Zilucoplan | 0 | Some concerns | Low risk | No concerns | Major concerns | No concerns | Major concerns | Very low |
| **4. MG-QoL 15r** |  |  |  |  |  |  |  |  |
| Batoclimab_340mg: Batoclimab_680mg | 1 | No concerns | Low risk | No concerns | Some concerns | No concerns | Major concerns | Moderate |
| Batoclimab_340mg: Placebo | 1 | No concerns | Low risk | No concerns | No concerns | No concerns | Major concerns | High |
| Batoclimab_680mg: Placebo | 1 | No concerns | Low risk | No concerns | No concerns | No concerns | Major concerns | High |
| Eculizumab: Placebo | 1 | No concerns | Low risk | No concerns | No concerns | No concerns | Major concerns | High |
| Efgartigimod_10mg_kg: Placebo | 2 | No concerns | Low risk | No concerns | No concerns | No concerns | Major concerns | High |
| Iscalimab: Placebo | 1 | No concerns | Low risk | No concerns | No concerns | No concerns | Major concerns | High |
| Mezagitamab_300mg: Mezagitamab_600mg | 1 | No concerns | Low risk | No concerns | Some concerns | No concerns | Major concerns | Moderate |
| Mezagitamab_300mg: Placebo | 1 | No concerns | Low risk | No concerns | No concerns | No concerns | Major concerns | High |
| Mezagitamab_600mg: Placebo | 1 | No concerns | Low risk | No concerns | No concerns | No concerns | Major concerns | High |
| Nipocalimab_30mg/kg: Nipocalimab_5mg/kg | 1 | No concerns | Low risk | No concerns | Some concerns | No concerns | Major concerns | Moderate |
| Nipocalimab_30mg/kg: Nipocalimab_60mg/kg | 1 | No concerns | Low risk | No concerns | Some concerns | No concerns | Major concerns | Moderate |
| Nipocalimab_30mg/kg: Placebo | 1 | No concerns | Low risk | No concerns | No concerns | No concerns | Major concerns | High |
| Nipocalimab_5mg/kg: Nipocalimab_60mg/kg | 1 | No concerns | Low risk | No concerns | Some concerns | No concerns | Major concerns | Moderate |
| Nipocalimab_5mg/kg: Placebo | 1 | No concerns | Low risk | No concerns | No concerns | No concerns | Major concerns | High |
| Nipocalimab_60mg/kg: Placebo | 1 | No concerns | Low risk | No concerns | No concerns | No concerns | Major concerns | High |
| Placebo: Ravulizumab | 1 | No concerns | Low risk | No concerns | No concerns | No concerns | Major concerns | High |
| Placebo: Rituximab | 1 | No concerns | Low risk | No concerns | No concerns | No concerns | Major concerns | High |
| Placebo: Rozanolixzumb_10mg_kg | 1 | Some concerns | Low risk | No concerns | No concerns | No concerns | Major concerns | High |
| Placebo: Rozanolixzumb_7mg_kg | 1 | Some concerns | Low risk | No concerns | No concerns | No concerns | Major concerns | High |
| Placebo: Zilucoplan | 1 | No concerns | Low risk | No concerns | No concerns | No concerns | Major concerns | High |
| Rozanolixzumb_10mg_kg: Rozanolixzumb_7mg_kg | 1 | Some concerns | Low risk | No concerns | Some concerns | No concerns | Major concerns | low |
| Batoclimab_340mg: Eculizumab | 0 | No concerns | Low risk | No concerns | Some concerns | No concerns | Major concerns | Very low |
| Batoclimab_340mg: Efgartigimod_10mg_kg | 0 | No concerns | Low risk | No concerns | Major concerns | No concerns | Major concerns | Very low |
| Batoclimab_340mg: Iscalimab | 0 | No concerns | Low risk | No concerns | Major concerns | No concerns | Major concerns | Very low |
| Batoclimab_340mg: Mezagitamab_300mg | 0 | No concerns | Low risk | No concerns | Major concerns | Some concerns | Major concerns | Very low |
| Batoclimab_340mg: Mezagitamab_600mg | 0 | No concerns | Low risk | No concerns | Major concerns | No concerns | Major concerns | Very low |
| Batoclimab_340mg: Nipocalimab_30mg/kg | 0 | No concerns | Low risk | No concerns | Major concerns | No concerns | Major concerns | Very low |
| Batoclimab_340mg: Nipocalimab_5mg/kg | 0 | No concerns | Low risk | No concerns | Major concerns | No concerns | Major concerns | Very low |
| Batoclimab_340mg: Nipocalimab_60mg/kg | 0 | No concerns | Low risk | No concerns | Major concerns | No concerns | Major concerns | Very low |
| Batoclimab_340mg: Ravulizumab | 0 | No concerns | Low risk | No concerns | Some concerns | No concerns | Major concerns | Very low |
| Batoclimab_340mg: Rituximab | 0 | No concerns | Low risk | No concerns | Some concerns | No concerns | Major concerns | Very low |
| Batoclimab_340mg: Rozanolixzumb_10mg_kg | 0 | Some concerns | Low risk | No concerns | Some concerns | No concerns | Major concerns | Very low |
| Batoclimab_340mg: Rozanolixzumb_7mg_kg | 0 | Some concerns | Low risk | No concerns | Some concerns | No concerns | Major concerns | Very low |
| Batoclimab_340mg: Zilucoplan | 0 | No concerns | Low risk | No concerns | Some concerns | No concerns | Major concerns | Very low |
| Batoclimab_680mg: Eculizumab | 0 | No concerns | Low risk | No concerns | Major concerns | No concerns | Major concerns | Very low |
| Batoclimab_680mg: Efgartigimod_10mg_kg | 0 | No concerns | Low risk | No concerns | Some concerns | No concerns | Major concerns | low |
| Batoclimab_680mg: Iscalimab | 0 | No concerns | Low risk | No concerns | Major concerns | No concerns | Major concerns | Very low |
| Batoclimab_680mg: Mezagitamab_300mg | 0 | No concerns | Low risk | No concerns | Major concerns | No concerns | Major concerns | Very low |
| Batoclimab_680mg: Mezagitamab_600mg | 0 | No concerns | Low risk | No concerns | Major concerns | No concerns | Major concerns | Very low |
| Batoclimab_680mg: Nipocalimab_30mg/kg | 0 | No concerns | Low risk | No concerns | Some concerns | Some concerns | Major concerns | Very low |
| Batoclimab_680mg: Nipocalimab_5mg/kg | 0 | No concerns | Low risk | No concerns | Some concerns | Some concerns | Major concerns | Very low |
| Batoclimab_680mg: Nipocalimab_60mg/kg | 0 | No concerns | Low risk | No concerns | Some concerns | Some concerns | Major concerns | Very low |
| Batoclimab_680mg: Ravulizumab | 0 | No concerns | Low risk | No concerns | Some concerns | No concerns | Major concerns | low |
| Batoclimab_680mg: Rituximab | 0 | No concerns | Low risk | No concerns | Some concerns | No concerns | Major concerns | low |
| Batoclimab_680mg: Rozanolixzumb_10mg_kg | 0 | No concerns | Low risk | No concerns | Some concerns | No concerns | Major concerns | low |
| Batoclimab_680mg: Rozanolixzumb_7mg_kg | 0 | Some concerns | Low risk | No concerns | Some concerns | No concerns | Major concerns | Very low |
| Batoclimab_680mg: Zilucoplan | 0 | No concerns | Low risk | No concerns | Major concerns | No concerns | Major concerns | Very low |
| Eculizumab: Efgartigimod_10mg_kg | 0 | No concerns | Low risk | No concerns | Major concerns | No concerns | Major concerns | Very low |
| Eculizumab: Iscalimab | 0 | No concerns | Low risk | No concerns | Major concerns | No concerns | Major concerns | Very low |
| Eculizumab: Mezagitamab_300mg | 0 | No concerns | Low risk | No concerns | Major concerns | No concerns | Major concerns | Very low |
| Eculizumab: Mezagitamab_600mg | 0 | No concerns | Low risk | No concerns | Major concerns | No concerns | Major concerns | Very low |
| Eculizumab: Nipocalimab_30mg/kg | 0 | No concerns | Low risk | No concerns | Major concerns | No concerns | Major concerns | Very low |
| Eculizumab: Nipocalimab_5mg/kg | 0 | No concerns | Low risk | No concerns | Major concerns | No concerns | Major concerns | Very low |
| Eculizumab: Nipocalimab_60mg/kg | 0 | No concerns | Low risk | No concerns | Major concerns | No concerns | Major concerns | Very low |
| Eculizumab: Ravulizumab | 0 | No concerns | Low risk | No concerns | Some concerns | No concerns | Major concerns | Very low |
| Eculizumab: Rituximab | 0 | No concerns | Low risk | No concerns | Some concerns | No concerns | Major concerns | low |
| Eculizumab: Rozanolixzumb_10mg_kg | 0 | Some concerns | Low risk | No concerns | Some concerns | No concerns | Major concerns | Very low |
| Eculizumab: Rozanolixzumb_7mg_kg | 0 | Some concerns | Low risk | No concerns | Some concerns | No concerns | Major concerns | Very low |
| Eculizumab: Zilucoplan | 0 | No concerns | Low risk | No concerns | Major concerns | No concerns | Major concerns | Very low |
| Efgartigimod_10mg_kg: Iscalimab | 0 | No concerns | Low risk | No concerns | Major concerns | No concerns | Major concerns | Very low |
| Efgartigimod_10mg_kg: Mezagitamab_300mg | 0 | No concerns | Low risk | No concerns | Major concerns | Some concerns | Major concerns | Very low |
| Efgartigimod_10mg_kg: Mezagitamab_600mg | 0 | No concerns | Low risk | No concerns | Major concerns | No concerns | Major concerns | Very low |
| Efgartigimod_10mg_kg: Nipocalimab_30mg/kg | 0 | No concerns | Low risk | No concerns | Major concerns | No concerns | Major concerns | Very low |
| Efgartigimod_10mg_kg: Nipocalimab_5mg/kg | 0 | No concerns | Low risk | No concerns | Major concerns | No concerns | Major concerns | Very low |
| Efgartigimod_10mg_kg: Nipocalimab_60mg/kg | 0 | No concerns | Low risk | No concerns | Major concerns | No concerns | Major concerns | Very low |
| Efgartigimod_10mg_kg: Ravulizumab | 0 | No concerns | Low risk | No concerns | Major concerns | No concerns | Major concerns | Very low |
| Efgartigimod_10mg_kg: Rituximab | 0 | No concerns | Low risk | No concerns | Major concerns | No concerns | Major concerns | Very low |
| Efgartigimod_10mg_kg: Rozanolixzumb_10mg_kg | 0 | Some concerns | Low risk | No concerns | Major concerns | No concerns | Major concerns | Very low |
| Efgartigimod_10mg_kg: Rozanolixzumb_7mg_kg | 0 | Some concerns | Low risk | No concerns | Major concerns | No concerns | Major concerns | Very low |
| Efgartigimod_10mg_kg: Zilucoplan | 0 | No concerns | Low risk | No concerns | Major concerns | No concerns | Major concerns | Very low |
| Iscalimab: Mezagitamab_300mg | 0 | No concerns | Low risk | No concerns | Major concerns | No concerns | Major concerns | Very low |
| Iscalimab: Mezagitamab_600mg | 0 | No concerns | Low risk | No concerns | Major concerns | No concerns | Major concerns | Very low |
| Iscalimab: Nipocalimab_30mg/kg | 0 | No concerns | Low risk | No concerns | Major concerns | No concerns | Major concerns | Very low |
| Iscalimab: Nipocalimab_5mg/kg | 0 | No concerns | Low risk | No concerns | Major concerns | No concerns | Major concerns | Very low |
| Iscalimab: Nipocalimab_60mg/kg | 0 | No concerns | Low risk | No concerns | Major concerns | No concerns | Major concerns | Very low |
| Iscalimab: Ravulizumab | 0 | No concerns | Low risk | No concerns | No concerns | Major concerns | Major concerns | Very low |
| Iscalimab: Rituximab | 0 | No concerns | Low risk | No concerns | Some concerns | Some concerns | Major concerns | Very low |
| Iscalimab: Rozanolixzumb_10mg_kg | 0 | Some concerns | Low risk | No concerns | Major concerns | No concerns | Major concerns | Very low |
| Iscalimab: Rozanolixzumb_7mg_kg | 0 | Some concerns | Low risk | No concerns | Major concerns | No concerns | Major concerns | Very low |
| Iscalimab: Zilucoplan | 0 | No concerns | Low risk | No concerns | Major concerns | No concerns | Major concerns | Very low |
| Mezagitamab_300mg: Nipocalimab_30mg/kg | 0 | No concerns | Low risk | No concerns | Major concerns | No concerns | Major concerns | Very low |
| Mezagitamab_300mg: Nipocalimab_5mg/kg | 0 | No concerns | Low risk | No concerns | Major concerns | No concerns | Major concerns | Very low |
| Mezagitamab_300mg: Nipocalimab_60mg/kg | 0 | No concerns | Low risk | No concerns | Major concerns | No concerns | Major concerns | Very low |
| Mezagitamab_300mg: Ravulizumab | 0 | No concerns | Low risk | No concerns | Major concerns | No concerns | Major concerns | Very low |
| Mezagitamab_300mg: Rituximab | 0 | No concerns | Low risk | No concerns | Major concerns | No concerns | Major concerns | Very low |
| Mezagitamab_300mg: Rozanolixzumb_10mg_kg | 0 | No concerns | Low risk | No concerns | Major concerns | No concerns | Major concerns | Very low |
| Mezagitamab_300mg: Rozanolixzumb_7mg_kg | 0 | No concerns | Low risk | No concerns | Major concerns | No concerns | Major concerns | Very low |
| Mezagitamab_300mg: Zilucoplan | 0 | No concerns | Low risk | No concerns | Major concerns | No concerns | Major concerns | Very low |
| Mezagitamab_600mg: Nipocalimab_30mg/kg | 0 | No concerns | Low risk | No concerns | Major concerns | No concerns | Major concerns | Very low |
| Mezagitamab_600mg: Nipocalimab_5mg/kg | 0 | No concerns | Low risk | No concerns | Major concerns | No concerns | Major concerns | Very low |
| Mezagitamab_600mg: Nipocalimab_60mg/kg | 0 | No concerns | Low risk | No concerns | Major concerns | No concerns | Major concerns | Very low |
| Mezagitamab_600mg: Ravulizumab | 0 | No concerns | Low risk | No concerns | Major concerns | No concerns | Major concerns | Very low |
| Mezagitamab_600mg: Rituximab | 0 | No concerns | Low risk | No concerns | Major concerns | No concerns | Major concerns | Very low |
| Mezagitamab_600mg: Rozanolixzumb_10mg_kg | 0 | No concerns | Low risk | No concerns | Major concerns | No concerns | Major concerns | Very low |
| Mezagitamab_600mg: Rozanolixzumb_7mg_kg | 0 | No concerns | Low risk | No concerns | Major concerns | No concerns | Major concerns | Very low |
| Mezagitamab_600mg: Zilucoplan | 0 | No concerns | Low risk | No concerns | Major concerns | No concerns | Major concerns | Very low |
| Nipocalimab_30mg/kg: Ravulizumab | 0 | No concerns | Low risk | No concerns | Major concerns | No concerns | Major concerns | Very low |
| Nipocalimab_30mg/kg: Rituximab | 0 | No concerns | Low risk | No concerns | Major concerns | No concerns | Major concerns | Very low |
| Nipocalimab_30mg/kg: Rozanolixzumb_10mg_kg | 0 | No concerns | Low risk | No concerns | Major concerns | No concerns | Major concerns | Very low |
| Nipocalimab_30mg/kg: Rozanolixzumb_7mg_kg | 0 | No concerns | Low risk | No concerns | Major concerns | No concerns | Major concerns | Very low |
| Nipocalimab_30mg/kg: Zilucoplan | 0 | No concerns | Low risk | No concerns | Some concerns | Some concerns | Major concerns | Very low |
| Nipocalimab_5mg/kg: Ravulizumab | 0 | No concerns | Low risk | No concerns | Some concerns | Some concerns | Major concerns | Very low |
| Nipocalimab_5mg/kg: Rituximab | 0 | No concerns | Low risk | No concerns | Major concerns | Some concerns | Major concerns | Very low |
| Nipocalimab_5mg/kg: Rozanolixzumb_10mg_kg | 0 | No concerns | Low risk | No concerns | Major concerns | Major concerns | Major concerns | Very low |
| Nipocalimab_5mg/kg: Rozanolixzumb_7mg_kg | 0 | No concerns | Low risk | No concerns | Major concerns | Major concerns | Major concerns | Very low |
| Nipocalimab_5mg/kg: Zilucoplan | 0 | No concerns | Low risk | No concerns | Major concerns | Major concerns | Major concerns | Very low |
| Nipocalimab_60mg/kg: Ravulizumab | 0 | No concerns | Low risk | No concerns | Major concerns | Some concerns | Major concerns | Very low |
| Nipocalimab_60mg/kg: Rituximab | 0 | No concerns | Low risk | No concerns | Major concerns | Some concerns | Major concerns | Very low |
| Nipocalimab_60mg/kg: Rozanolixzumb_10mg_kg | 0 | No concerns | Low risk | No concerns | Major concerns | Major concerns | Major concerns | Very low |
| Nipocalimab_60mg/kg: Rozanolixzumb_7mg_kg | 0 | No concerns | Low risk | No concerns | Major concerns | Some concerns | Major concerns | Very low |
| Nipocalimab_60mg/kg: Zilucoplan | 0 | No concerns | Low risk | No concerns | Major concerns | Some concerns | Major concerns | Very low |
| Ravulizumab: Rituximab | 0 | No concerns | Low risk | No concerns | Some concerns | Some concerns | Major concerns | Very low |
| Ravulizumab: Rozanolixzumb_10mg_kg | 0 | Some concerns | Low risk | No concerns | Some concerns | Some concerns | Major concerns | Very low |
| Ravulizumab: Rozanolixzumb_7mg_kg | 0 | Some concerns | Low risk | No concerns | Some concerns | Major concerns | Major concerns | Very low |
| Ravulizumab: Zilucoplan | 0 | No concerns | Low risk | No concerns | Some concerns | Some concerns | Major concerns | Very low |
| Rituximab: Rozanolixzumb_10mg_kg | 0 | Some concerns | Low risk | No concerns | No concerns | Some concerns | Major concerns | Very low |
| Rituximab: Rozanolixzumb_7mg_kg | 0 | Some concerns | Low risk | No concerns | Some concerns | Some concerns | Major concerns | Very low |
| Rituximab: Zilucoplan | 0 | No concerns | Low risk | No concerns | Some concerns | Some concerns | Major concerns | low |
| Rozanolixzumb_10mg_kg: Zilucoplan | 0 | Some concerns | Low risk | No concerns | Some concerns | Some concerns | Major concerns | low |
| Rozanolixzumb_7mg_kg: Zilucoplan | 0 | Some concerns | Low risk | No concerns | Some concerns | Some concerns | Major concerns | low |
| **3. AEs** | | | | | | | | |
| Batoclimab__340mg vs. Batoclimab__680mg | 1 | No concerns | Low risk | No concerns | Major concerns | No concerns | Major concerns | Low |
| Batoclimab__340mg vs. Placebo | 1 | No concerns | Low risk | No concerns | Major concerns | No concerns | Major concerns | Low |
| Batoclimab__680mg vs. Placebo | 1 | No concerns | Low risk | No concerns | Major concerns | No concerns | Major concerns | Low |
| Belimumab vs. Placebo | 1 | No concerns | Low risk | No concerns | Major concerns | No concerns | Major concerns | Low |
| Efgartigimod 10mg/kg vs. Placebo | 2 | No concerns | Low risk | No concerns | Major concerns | No concerns | Major concerns | Low |
| Mezagitamab_300mg vs. Mezagitamab_600mg | 1 | No concerns | Low risk | No concerns | Major concerns | No concerns | Major concerns | Low |
| Mezagitamab_300mg vs. Placebo | 1 | No concerns | Low risk | No concerns | Major concerns | No concerns | Major concerns | Low |
| Mezagitamab_600mg vs. Placebo | 1 | No concerns | Low risk | No concerns | Major concerns | No concerns | Major concerns | Low |
| Nipocalimab_30mg/kg vs. Nipocalimab_5mg/kg | 1 | No concerns | Low risk | No concerns | Major concerns | No concerns | Major concerns | Low |
| Nipocalimab_30mg/kg vs. Nipocalimab_60mg/kg | 1 | No concerns | Low risk | No concerns | Major concerns | No concerns | Major concerns | Low |
| Nipocalimab_30mg/kg vs. Placebo | 1 | No concerns | Low risk | No concerns | Major concerns | No concerns | Major concerns | Low |
| Nipocalimab_5mg/kg vs. Nipocalimab_60mg/kg | 1 | No concerns | Low risk | No concerns | Major concerns | No concerns | Major concerns | Low |
| Nipocalimab_5mg/kg vs. Placebo | 1 | No concerns | Low risk | No concerns | Major concerns | No concerns | Major concerns | Low |
| Nipocalimab_60mg/kg vs. Placebo | 1 | No concerns | Low risk | No concerns | Major concerns | No concerns | Major concerns | Low |
| Placebo vs. Ravulizumab | 1 | No concerns | Low risk | No concerns | Major concerns | No concerns | Major concerns | Low |
| Placebo vs. Rituximab | 2 | No concerns | Low risk | No concerns | Major concerns | No concerns | Major concerns | Low |
| Placebo vs. Rozanolixzumb_10mg/kg | 1 | Some concerns | Low risk | No concerns | No concerns | Major concerns | Major concerns | Very low |
| Placebo vs. Rozanolixzumb_7mg/kg | 1 | Some concerns | Low risk | No concerns | Some concerns | Some concerns | Major concerns | Low |
| Placebo vs. Zilucoplan | 1 | No concerns | Low risk | No concerns | Major concerns | No concerns | Major concerns | Low |
| Rozanolixzumb_10mg/kg vs. Rozanolixzumb_7mg/kg | 1 | Some concerns | Low risk | No concerns | Major concerns | No concerns | Major concerns | Low |
| Batoclimab_340mg vs. Belimumab | 0 | No concerns | Low risk | No concerns | Major concerns | No concerns | Major concerns | Low |
| Batoclimab_340mg vs. Efgartigimod_10mg/kg | 0 | No concerns | Low risk | No concerns | Major concerns | No concerns | Major concerns | Low |
| Batoclimab_340mg vs. Mezagitamab_300mg | 0 | No concerns | Low risk | No concerns | Major concerns | No concerns | Major concerns | Low |
| Batoclimab_340mg vs. Mezagitamab_600mg | 0 | No concerns | Low risk | No concerns | Major concerns | No concerns | Major concerns | Low |
| Batoclimab_340mg vs. Nipocalimab_0mg/kg | 0 | No concerns | Low risk | No concerns | Major concerns | No concerns | Major concerns | Low |
| Batoclimab_340mg vs. Nipocalimab_5mg/kg | 0 | No concerns | Low risk | No concerns | Major concerns | No concerns | Major concerns | Low |
| Batoclimab_340mg vs. Nipocalimab_60mg/kg | 0 | No concerns | Low risk | No concerns | Major concerns | No concerns | Major concerns | Low |
| Batoclimab_340mg vs. Ravulizumab | 0 | No concerns | Low risk | No concerns | Major concerns | No concerns | Major concerns | Low |
| Batoclimab_340mg vs. Rituximab | 0 | No concerns | Low risk | No concerns | Major concerns | No concerns | Major concerns | Low |
| Batoclimab_340mg vs. Rozanolixzumb_10mg/kg | 0 | No concerns | Low risk | No concerns | Major concerns | No concerns | Major concerns | Low |
| Batoclimab_340mg vs. Rozanolixzumb_7mg/kg | 0 | No concerns | Low risk | No concerns | Major concerns | No concerns | Major concerns | Low |
| Batoclimab_340mg vs. Zilucoplan | 0 | No concerns | Low risk | No concerns | Major concerns | No concerns | Major concerns | Low |
| Batoclimab_680mg vs. Belimumab | 0 | No concerns | Low risk | No concerns | Major concerns | No concerns | Major concerns | Low |
| Batoclimab_680mg vs. Efgartigimod_10mg/kg | 0 | No concerns | Low risk | No concerns | Major concerns | No concerns | Major concerns | Low |
| Batoclimab_680mg vs. Mezagitamab_300mg | 0 | No concerns | Low risk | No concerns | Major concerns | No concerns | Major concerns | Low |
| Batoclimab_680mg vs. Mezagitamab_600mg | 0 | No concerns | Low risk | No concerns | Major concerns | No concerns | Major concerns | Low |
| Batoclimab_680mg vs. Nipocalimab_30mg/kg | 0 | No concerns | Low risk | No concerns | Major concerns | No concerns | Major concerns | Low |
| Batoclimab_680mg vs. Nipocalimab_5mg/kg | 0 | No concerns | Low risk | No concerns | Major concerns | No concerns | Major concerns | Low |
| Batoclimab_680mg vs. Nipocalimab_60mg/kg | 0 | No concerns | Low risk | No concerns | Major concerns | No concerns | Major concerns | Low |
| Batoclimab_680mg vs. Ravulizumab | 0 | No concerns | Low risk | No concerns | Major concerns | No concerns | Major concerns | Low |
| Batoclimab_680mg vs. Rituximab | 0 | No concerns | Low risk | No concerns | Major concerns | No concerns | Major concerns | Low |
| Batoclimab_680mg vs. Rozanolixzumb_10mg/kg | 0 | Some concerns | Low risk | No concerns | Major concerns | No concerns | Major concerns | Very low |
| Batoclimab__680mg vs. Rozanolixzumb_7mg/kg | 0 | Some concerns | Low risk | No concerns | Major concerns | No concerns | Major concerns | Very low |
| Batoclimab__680mg vs. Zilucoplan | 0 | No concerns | Low risk | No concerns | Major concerns | No concerns | Major concerns | Low |
| Belimumab vs. Efgartigimod_10mg/kg | 0 | No concerns | Low risk | No concerns | Major concerns | No concerns | Major concerns | Low |
| Belimumab vs. Mezagitamab_300mg | 0 | No concerns | Low risk | No concerns | Major concerns | No concerns | Major concerns | Low |
| Belimumab vs. Mezagitamab_600mg | 0 | No concerns | Low risk | No concerns | No concerns | Major concerns | Major concerns | Very low |
| Belimumab vs. Nipocalimab__30mg/kg | 0 | No concerns | Low risk | No concerns | Major concerns | No concerns | Major concerns | Low |
| Belimumab vs. Nipocalimab__5mg/kg | 0 | No concerns | Low risk | No concerns | Major concerns | No concerns | Major concerns | Low |
| Belimumab vs. Nipocalimab__60mg/kg | 0 | No concerns | Low risk | No concerns | Major concerns | No concerns | Major concerns | Low |
| Belimumab vs. Ravulizumab | 0 | No concerns | Low risk | No concerns | Major concerns | No concerns | Major concerns | Low |
| Belimumab vs. Rituximab | 0 | No concerns | Low risk | No concerns | Major concerns | No concerns | Major concerns | Low |
| Belimumab vs. Rozanolixzumb_10mg/kg | 0 | Some concerns | Low risk | No concerns | No concerns | Major concerns | Major concerns | Very low |
| Belimumab vs. Rozanolixzumb_7mg/kg | 0 | Some concerns | Low risk | No concerns | No concerns | Major concerns | Major concerns | Very low |
| Belimumab vs. Zilucoplan | 0 | No concerns | Low risk | No concerns | Major concerns | No concerns | Major concerns | Low |
| Efgartigimod_10mg/kg vs. Mezagitamab_300mg | 0 | No concerns | Low risk | No concerns | Major concerns | No concerns | Major concerns | Low |
| Efgartigimod_10mg/kg vs. Mezagitamab_600mg | 0 | No concerns | Low risk | No concerns | Major concerns | No concerns | Major concerns | Low |
| Efgartigimod_10mg/kg vs. Nipocalimab__30mg/kg | 0 | No concerns | Low risk | No concerns | Major concerns | No concerns | Major concerns | Low |
| Efgartigimod_10mg/kg vs. Nipocalimab__5mg/kg | 0 | No concerns | Low risk | No concerns | Major concerns | No concerns | Major concerns | Low |
| Efgartigimod_10mg/kg vs. Nipocalimab__60mg/kg | 0 | No concerns | Low risk | No concerns | Major concerns | No concerns | Major concerns | Low |
| Efgartigimod_10mg/kg vs. Ravulizumab | 0 | No concerns | Low risk | No concerns | Major concerns | No concerns | Major concerns | Low |
| Efgartigimod_10mg/kg vs. Rituximab | 0 | No concerns | Low risk | No concerns | Major concerns | No concerns | Major concerns | Low |
| Efgartigimod_10mg/kg vs. Rozanolixzumb_10mg/kg | 0 | Some concerns | Low risk | No concerns | No concerns | Major concerns | Major concerns | Very low |
| Efgartigimod_10mg/kg vs. Rozanolixzumb_7mg/kg | 0 | Some concerns | Low risk | No concerns | No concerns | Major concerns | Major concerns | Very low |
| Efgartigimod vs. Zilucoplan | 0 | No concerns | Low risk | No concerns | Major concerns | No concerns | Major concerns | Low |
| Mezagitamab_300mg vs. Nipocalimab_30mg/kg | 0 | No concerns | Low risk | No concerns | Major concerns | No concerns | Major concerns | Low |
| Mezagitamab_300mg vs. Nipocalimab_5mg/kg | 0 | No concerns | Low risk | No concerns | Major concerns | No concerns | Major concerns | Low |
| Mezagitamab_300mg vs. Nipocalimab_60mg/kg | 0 | No concerns | Low risk | No concerns | Major concerns | No concerns | Major concerns | Low |
| Mezagitamab_300mg vs. Ravulizumab | 0 | No concerns | Low risk | No concerns | Major concerns | No concerns | Major concerns | Low |
| Mezagitamab_300mg vs. Rituximab | 0 | No concerns | Low risk | No concerns | Major concerns | No concerns | Major concerns | Low |
| Mezagitamab_300mg vs. Rozanolixzumb_10mg/kg | 0 | Some concerns | Low risk | No concerns | Major concerns | No concerns | Major concerns | Very low |
| Mezagitamab_300mg vs. Rozanolixzumb_7mg/kg | 0 | Some concerns | Low risk | No concerns | Major concerns | No concerns | Major concerns | Very low |
| Mezagitamab_300mg vs. Zilucoplan | 0 | No concerns | Low risk | No concerns | Major concerns | No concerns | Major concerns | Low |
| Mezagitamab_600mg vs. Nipocalimab__30mg/kg | 0 | No concerns | Low risk | No concerns | Major concerns | No concerns | Major concerns | Low |
| Mezagitamab_600mg vs. Nipocalimab__5mg/kg | 0 | No concerns | Low risk | No concerns | Major concerns | No concerns | Major concerns | Low |
| Mezagitamab_600mg vs. Nipocalimab__60mg/kg | 0 | No concerns | Low risk | No concerns | Major concerns | No concerns | Major concerns | Low |
| Mezagitamab_600mg vs. Ravulizumab | 0 | No concerns | Low risk | No concerns | Major concerns | No concerns | Major concerns | Low |
| Mezagitamab_600mg vs. Rituximab | 0 | No concerns | Low risk | No concerns | Major concerns | No concerns | Major concerns | Low |
| Mezagitamab_600mg vs. Rozanolixzumb_10mg/kg | 0 | No concerns | Low risk | No concerns | Major concerns | No concerns | Major concerns | Low |
| Mezagitamab_600mg vs. Rozanolixzumb_7mg/kg | 0 | No concerns | Low risk | No concerns | Major concerns | No concerns | Major concerns | Low |
| Mezagitamab_600mg vs. Zilucoplan | 0 | No concerns | Low risk | No concerns | Major concerns | No concerns | Major concerns | Low |
| Nipocalimab_30mg/kg vs. Ravulizumab | 0 | No concerns | Low risk | No concerns | Major concerns | No concerns | Major concerns | Low |
| Nipocalimab_30mg/kg vs. Rituximab | 0 | No concerns | Low risk | No concerns | Major concerns | No concerns | Major concerns | Low |
| Nipocalimab_30mg/kg vs. Rozanolixzumb_10mg/kg | 0 | No concerns | Low risk | No concerns | Major concerns | No concerns | Major concerns | Low |
| Nipocalimab_30mg/kg vs. Rozanolixzumb_7mg/kg | 0 | No concerns | Low risk | No concerns | Major concerns | No concerns | Major concerns | Low |
| Nipocalimab_30mg/kg vs. Zilucoplan | 0 | No concerns | Low risk | No concerns | Major concerns | No concerns | Major concerns | Low |
| Nipocalimab_5mg/kg vs. Ravulizumab | 0 | No concerns | Low risk | No concerns | Major concerns | No concerns | Major concerns | Low |
| Nipocalimab_5mg/kg vs. Rituximab | 0 | No concerns | Low risk | No concerns | Major concerns | No concerns | Major concerns | Low |
| Nipocalimab_5mg/kg vs. Rozanolixzumb_10mg/kg | 0 | No concerns | Low risk | No concerns | Major concerns | No concerns | Major concerns | Low |
| Nipocalimab_5mg/kg vs. Rozanolixzumb_7mg/kg | 0 | No concerns | Low risk | No concerns | Major concerns | No concerns | Major concerns | Low |
| Nipocalimab_5mg/kg vs. Zilucoplan | 0 | No concerns | Low risk | No concerns | Major concerns | No concerns | Major concerns | Low |
| Nipocalimab_60mg/kg vs. Ravulizumab | 0 | No concerns | Low risk | No concerns | Major concerns | No concerns | Major concerns | Low |
| Nipocalimab_60mg/kg vs. Rituximab | 0 | No concerns | Low risk | No concerns | Major concerns | No concerns | Major concerns | Low |
| Nipocalimab_60mg/kg vs. Rozanolixzumb_10mg/kg | 0 | No concerns | Low risk | No concerns | Major concerns | No concerns | Major concerns | Low |
| Nipocalimab_60mg/kg vs. Rozanolixzumb_7mg/kg | 0 | No concerns | Low risk | No concerns | Major concerns | No concerns | Major concerns | Low |
| Nipocalimab_60mg/kg vs. Zilucoplan | 0 | No concerns | Low risk | No concerns | Major concerns | No concerns | Major concerns | Low |
| Ravulizumab vs. Rituximab | 0 | No concerns | Low risk | No concerns | Major concerns | No concerns | Major concerns | Low |
| Ravulizumab vs. Rozanolixzumb_10mg/kg | 0 | Some concerns | Low risk | No concerns | Some concerns | Some concerns | Major concerns | Very low |
| Ravulizumab vs. Rozanolixzumb_7mg/kg | 0 | Some concerns | Low risk | No concerns | Major concerns | No concerns | Major concerns | Very low |
| Ravulizumab vs. Zilucoplan | 0 | No concerns | Low risk | No concerns | Major concerns | No concerns | Major concerns | Low |
| Rituximab vs. Rozanolixzumb_10mg/kg | 0 | Some concerns | Low risk | No concerns | Major concerns | No concerns | Major concerns | Very low |
| Mezagitamab_600mg vs. Rozanolixzumb_10mg/kg | 0 | No concerns | Low risk | No concerns | Major concerns | No concerns | Major concerns | Low |
| Mezagitamab_600mg vs. Rozanolixzumb_7mg/kg | 0 | No concerns | Low risk | No concerns | Major concerns | No concerns | Major concerns | Low |
| Rituximab vs. Rozanolixzumb 7mg/kg | 0 | Some concerns | Low risk | No concerns | Major concerns | No concerns | Major concerns | Very low |
| Rituximab vs. Zilucoplan | 0 | No concerns | Low risk | No concerns | Major concerns | No concerns | Major concerns | Low |
| Rozanolixzumb 10mg/kg vs. Zilucoplan | 0 | Some concerns | Low risk | No concerns | Major concerns | No concerns | Major concerns | Very low |
| Rozanolixzumb 7mg/kg vs. Zilucoplan | 0 | Some concerns | Low risk | No concerns | Major concerns | No concerns | Major concerns | Very low |
| **4. SAEs** | | | | | | | | |
| Batoclimab_340mg: Batoclimab_680mg | 1 | No concerns | Low risk | No concerns | Major concerns | No concerns | Major concerns | Low |
| Batoclimab_340mg: Placebo | 1 | No concerns | Low risk | No concerns | Major concerns | No concerns | Major concerns | Low |
| Batoclimab_680mg: Placebo | 1 | No concerns | Low risk | No concerns | Major concerns | No concerns | Major concerns | Low |
| Belimumab: Placebo | 1 | No concerns | Low risk | No concerns | Major concerns | No concerns | Major concerns | Low |
| Eculizumab: Placebo | 1 | No concerns | Low risk | No concerns | Major concerns | No concerns | Major concerns | Low |
| Efgartigimod_10mg_kg: Placebo | 2 | No concerns | Low risk | No concerns | Major concerns | No concerns | Major concerns | Low |
| Iscalimab: Placebo | 1 | No concerns | Low risk | No concerns | Major concerns | No concerns | Major concerns | Low |
| Mezagitamab_300mg: Mezagitamab_600mg | 1 | No concerns | Low risk | No concerns | Major concerns | No concerns | Major concerns | Low |
| Mezagitamab_300mg: Placebo | 1 | No concerns | Low risk | No concerns | Major concerns | No concerns | Major concerns | Low |
| Mezagitamab_600mg: Placebo | 1 | No concerns | Low risk | No concerns | Major concerns | No concerns | Major concerns | Low |
| Nipocalimab_30mg_kg: Nipocalimab_5mg_kg | 1 | No concerns | Low risk | No concerns | Major concerns | No concerns | Major concerns | Low |
| Nipocalimab_30mg_kg: Nipocalimab_60mg_kg | 1 | No concerns | Low risk | No concerns | Major concerns | No concerns | Major concerns | Low |
| Nipocalimab_30mg_kg: Placebo | 1 | No concerns | Low risk | No concerns | Major concerns | No concerns | Major concerns | Low |
| Nipocalimab_5mg_kg: Nipocalimab_60mg_kg | 1 | No concerns | Low risk | No concerns | Major concerns | No concerns | Major concerns | Low |
| Nipocalimab_5mg_kg: Placebo | 1 | No concerns | Low risk | No concerns | Major concerns | No concerns | Major concerns | Low |
| Nipocalimab_60mg_kg: Placebo | 1 | No concerns | Low risk | No concerns | Major concerns | No concerns | Major concerns | Low |
| Placebo: Ravulizumab | 1 | No concerns | Low risk | No concerns | Major concerns | No concerns | Major concerns | Low |
| Placebo: Rituximab | 2 | No concerns | Low risk | No concerns | Major concerns | No concerns | Major concerns | Low |
| Placebo: Rozanolixzumb_10mg_kg | 1 | Some concerns | Low risk | No concerns | Major concerns | No concerns | Major concerns | Very low |
| Placebo: Rozanolixzumb_7mg_kg | 1 | Some concerns | Low risk | No concerns | Major concerns | No concerns | Major concerns | Very low |
| Placebo: Zilucoplan | 1 | No concerns | Low risk | No concerns | Major concerns | No concerns | Major concerns | Low |
| Rozanolixzumb_10mg_kg: Rozanolixzumb_7mg_kg | 1 | Some concerns | Low risk | No concerns | Major concerns | No concerns | Major concerns | Very low |
| Batoclimab_340mg: Belimumab | 0 | No concerns | Low risk | No concerns | Major concerns | No concerns | Major concerns | Low |
| Batoclimab_340mg: Eculizumab | 0 | No concerns | Low risk | No concerns | Major concerns | No concerns | Major concerns | Low |
| Batoclimab_340mg: Efgartigimod_10mg_kg | 0 | No concerns | Low risk | No concerns | Major concerns | No concerns | Major concerns | Low |
| Batoclimab_340mg: Iscalimab | 0 | No concerns | Low risk | No concerns | Major concerns | No concerns | Major concerns | Low |
| Batoclimab_340mg: Mezagitamab_300mg | 0 | No concerns | Low risk | No concerns | Major concerns | No concerns | Major concerns | Low |
| Batoclimab_340mg: Mezagitamab_600mg | 0 | No concerns | Low risk | No concerns | Major concerns | No concerns | Major concerns | Low |
| Batoclimab_340mg: Nipocalimab_30mg_kg | 0 | No concerns | Low risk | No concerns | Major concerns | No concerns | Major concerns | Low |
| Batoclimab_340mg: Nipocalimab_5mg_kg | 0 | No concerns | Low risk | No concerns | Major concerns | No concerns | Major concerns | Low |
| Batoclimab_340mg: Nipocalimab_60mg_kg | 0 | No concerns | Low risk | No concerns | Major concerns | No concerns | Major concerns | Low |
| Batoclimab_340mg: Ravulizumab | 0 | No concerns | Low risk | No concerns | Major concerns | No concerns | Major concerns | Low |
| Batoclimab_340mg: Rituximab | 0 | No concerns | Low risk | No concerns | Major concerns | No concerns | Major concerns | Low |
| Batoclimab_340mg: Rozanolixzumb_10mg_kg | 0 | No concerns | Low risk | No concerns | Major concerns | No concerns | Major concerns | Low |
| Batoclimab_340mg: Rozanolixzumb_7mg_kg | 0 | No concerns | Low risk | No concerns | Major concerns | No concerns | Major concerns | Very low |
| Batoclimab_340mg: Zilucoplan | 0 | No concerns | Low risk | No concerns | Major concerns | No concerns | Major concerns | Low |
| Batoclimab_680mg: Belimumab | 0 | No concerns | Low risk | No concerns | Major concerns | No concerns | Major concerns | Low |
| Batoclimab_680mg: Eculizumab | 0 | No concerns | Low risk | No concerns | Major concerns | No concerns | Major concerns | Low |
| Batoclimab_680mg: Efgartigimod_10mg_kg | 0 | No concerns | Low risk | No concerns | Major concerns | No concerns | Major concerns | Low |
| Batoclimab_680mg: Iscalimab | 0 | No concerns | Low risk | No concerns | Major concerns | No concerns | Major concerns | Low |
| Batoclimab_680mg: Mezagitamab_300mg | 0 | No concerns | Low risk | No concerns | Major concerns | No concerns | Major concerns | Low |
| Batoclimab_680mg: Mezagitamab_600mg | 0 | No concerns | Low risk | No concerns | Major concerns | No concerns | Major concerns | Low |
| Batoclimab_680mg: Nipocalimab_30mg_kg | 0 | No concerns | Low risk | No concerns | Major concerns | No concerns | Major concerns | Low |
| Batoclimab_680mg: Nipocalimab_5mg_kg | 0 | No concerns | Low risk | No concerns | Major concerns | No concerns | Major concerns | Low |
| Batoclimab_680mg: Nipocalimab_60mg_kg | 0 | No concerns | Low risk | No concerns | Major concerns | No concerns | Major concerns | Low |
| Batoclimab_680mg: Ravulizumab | 0 | No concerns | Low risk | No concerns | Major concerns | No concerns | Major concerns | Low |
| Batoclimab_680mg: Rituximab | 0 | No concerns | Low risk | No concerns | Major concerns | No concerns | Major concerns | Low |
| Batoclimab_680mg: Rozanolixzumb_10mg_kg | 0 | Some concerns | Low risk | No concerns | Major concerns | No concerns | Major concerns | Low |
| Batoclimab_680mg: Rozanolixzumb_7mg_kg | 0 | Some concerns | Low risk | No concerns | Major concerns | No concerns | Major concerns | Very low |
| Batoclimab_680mg: Zilucoplan | 0 | No concerns | Low risk | No concerns | Major concerns | No concerns | Major concerns | Low |
| Belimumab: Eculizumab | 0 | No concerns | Low risk | No concerns | Major concerns | No concerns | Major concerns | Low |
| Belimumab: Efgartigimod_10mg_kg | 0 | No concerns | Low risk | No concerns | Major concerns | No concerns | Major concerns | Low |
| Belimumab: Iscalimab | 0 | No concerns | Low risk | No concerns | Major concerns | No concerns | Major concerns | Low |
| Belimumab: Mezagitamab_300mg | 0 | No concerns | Low risk | No concerns | Major concerns | No concerns | Major concerns | Low |
| Belimumab: Mezagitamab_600mg | 0 | No concerns | Low risk | No concerns | Major concerns | No concerns | Major concerns | Low |
| Belimumab: Nipocalimab_30mg_kg | 0 | No concerns | Low risk | No concerns | Major concerns | No concerns | Major concerns | Low |
| Belimumab: Nipocalimab_5mg_kg | 0 | No concerns | Low risk | No concerns | Major concerns | No concerns | Major concerns | Low |
| Belimumab: Nipocalimab_60mg_kg | 0 | No concerns | Low risk | No concerns | Major concerns | No concerns | Major concerns | Low |
| Belimumab: Ravulizumab | 0 | No concerns | Low risk | No concerns | Major concerns | No concerns | Major concerns | Low |
| Belimumab: Rituximab | 0 | No concerns | Low risk | No concerns | Major concerns | No concerns | Major concerns | Low |
| Belimumab: Rozanolixzumb_10mg_kg | 0 | Some concerns | Low risk | No concerns | Major concerns | No concerns | Major concerns | Very low |
| Belimumab: Rozanolixzumb_7mg_kg | 0 | Some concerns | Low risk | No concerns | Major concerns | No concerns | Major concerns | Very low |
| Belimumab: Zilucoplan | 0 | No concerns | Low risk | No concerns | Major concerns | No concerns | Major concerns | Low |
| Eculizumab: Efgartigimod_10mg_kg | 0 | No concerns | Low risk | No concerns | Major concerns | No concerns | Major concerns | Low |
| Eculizumab: Iscalimab | 0 | No concerns | Low risk | No concerns | Major concerns | No concerns | Major concerns | Low |
| Eculizumab: Mezagitamab_300mg | 0 | No concerns | Low risk | No concerns | Major concerns | No concerns | Major concerns | Low |
| Eculizumab: Mezagitamab_600mg | 0 | No concerns | Low risk | No concerns | Major concerns | No concerns | Major concerns | Low |
| Eculizumab: Nipocalimab_30mg_kg | 0 | No concerns | Low risk | No concerns | Major concerns | No concerns | Major concerns | Low |
| Eculizumab: Nipocalimab_5mg_kg | 0 | No concerns | Low risk | No concerns | Major concerns | No concerns | Major concerns | Low |
| Eculizumab: Nipocalimab_60mg_kg | 0 | No concerns | Low risk | No concerns | Major concerns | No concerns | Major concerns | Low |
| Eculizumab: Ravulizumab | 0 | No concerns | Low risk | No concerns | Major concerns | No concerns | Major concerns | Low |
| Eculizumab: Rituximab | 0 | No concerns | Low risk | No concerns | Major concerns | No concerns | Major concerns | Low |
| Eculizumab: Rozanolixzumb_10mg_kg | 0 | Some concerns | Low risk | No concerns | Major concerns | No concerns | Major concerns | Very low |
| Eculizumab: Rozanolixzumb_7mg_kg | 0 | Some concerns | Low risk | No concerns | Major concerns | No concerns | Major concerns | Very low |
| Eculizumab: Zilucoplan | 0 | No concerns | Low risk | No concerns | Major concerns | No concerns | Major concerns | Low |
| Efgartigimod_10mg_kg: Iscalimab | 0 | No concerns | Low risk | No concerns | Major concerns | No concerns | Major concerns | Low |
| Efgartigimod_10mg_kg: Mezagitamab_300mg | 0 | No concerns | Low risk | No concerns | Major concerns | No concerns | Major concerns | Low |
| Efgartigimod_10mg_kg: Mezagitamab_600mg | 0 | No concerns | Low risk | No concerns | Major concerns | No concerns | Major concerns | Low |
| Efgartigimod_10mg_kg: Nipocalimab_30mg_kg | 0 | No concerns | Low risk | No concerns | Major concerns | No concerns | Major concerns | Low |
| Efgartigimod_10mg_kg: Nipocalimab_5mg_kg | 0 | No concerns | Low risk | No concerns | Major concerns | No concerns | Major concerns | Low |
| Efgartigimod_10mg_kg: Nipocalimab_60mg_kg | 0 | No concerns | Low risk | No concerns | Major concerns | No concerns | Major concerns | Low |
| Efgartigimod_10mg_kg: Ravulizumab | 0 | No concerns | Low risk | No concerns | Major concerns | No concerns | Major concerns | Low |
| Efgartigimod_10mg_kg: Rituximab | 0 | No concerns | Low risk | No concerns | Major concerns | No concerns | Major concerns | Low |
| Efgartigimod_10mg_kg: Rozanolixzumb_10mg_kg | 0 | Some concerns | Low risk | No concerns | Major concerns | No concerns | Major concerns | Very low |
| Efgartigimod_10mg_kg: Rozanolixzumb_7mg_kg | 0 | Some concerns | Low risk | No concerns | Major concerns | No concerns | Major concerns | Very low |
| Efgartigimod_10mg_kg: Zilucoplan | 0 | No concerns | Low risk | No concerns | Major concerns | No concerns | Major concerns | Low |
| Iscalimab: Mezagitamab_300mg | 0 | No concerns | Low risk | No concerns | Major concerns | No concerns | Major concerns | Low |
| Iscalimab: Mezagitamab_600mg | 0 | No concerns | Low risk | No concerns | Major concerns | No concerns | Major concerns | Low |
| Iscalimab: Nipocalimab_30mg_kg | 0 | No concerns | Low risk | No concerns | Major concerns | No concerns | Major concerns | Low |
| Iscalimab: Nipocalimab_5mg_kg | 0 | No concerns | Low risk | No concerns | Major concerns | No concerns | Major concerns | Low |
| Iscalimab: Nipocalimab_60mg_kg | 0 | No concerns | Low risk | No concerns | Major concerns | No concerns | Major concerns | Low |
| Iscalimab: Ravulizumab | 0 | No concerns | Low risk | No concerns | Major concerns | No concerns | Major concerns | Low |
| Iscalimab: Rituximab | 0 | No concerns | Low risk | No concerns | Major concerns | No concerns | Major concerns | Low |
| Iscalimab: Rozanolixzumb_10mg_kg | 0 | Some concerns | Low risk | No concerns | Major concerns | No concerns | Major concerns | Very low |
| Iscalimab: Rozanolixzumb_7mg_kg | 0 | Some concerns | Low risk | No concerns | Major concerns | No concerns | Major concerns | Very low |
| Iscalimab: Zilucoplan | 0 | No concerns | Low risk | No concerns | Major concerns | No concerns | Major concerns | Low |
| Mezagitamab_300mg: Nipocalimab_30mg_kg | 0 | No concerns | Low risk | No concerns | Major concerns | No concerns | Major concerns | Low |
| Mezagitamab_300mg: Nipocalimab_5mg_kg | 0 | No concerns | Low risk | No concerns | Major concerns | No concerns | Major concerns | Low |
| Mezagitamab_300mg: Nipocalimab_60mg_kg | 0 | No concerns | Low risk | No concerns | Major concerns | No concerns | Major concerns | Low |
| Mezagitamab_300mg: Ravulizumab | 0 | No concerns | Low risk | No concerns | Major concerns | No concerns | Major concerns | Low |
| Mezagitamab_600mg: Rituximab | 0 | No concerns | Low risk | No concerns | Major concerns | No concerns | Major concerns | Low |
| Mezagitamab_600mg: Rozanolixzumb_10mg_kg | 0 | No concerns | Low risk | No concerns | Major concerns | No concerns | Major concerns | Low |
| Mezagitamab_600mg: Rozanolixzumb_7mg_kg | 0 | Some concerns | Low risk | No concerns | Major concerns | No concerns | Major concerns | Very low |
| Mezagitamab_600mg: Zilucoplan | 0 | No concerns | Low risk | No concerns | Major concerns | No concerns | Major concerns | Low |
| Nipocalimab_30mg_kg: Ravulizumab | 0 | No concerns | Low risk | No concerns | Major concerns | No concerns | Major concerns | Low |
| Nipocalimab_30mg_kg: Rituximab | 0 | No concerns | Low risk | No concerns | Major concerns | No concerns | Major concerns | Low |
| Nipocalimab_30mg_kg: Rozanolixzumb_10mg_kg | 0 | No concerns | Low risk | No concerns | Major concerns | No concerns | Major concerns | Low |
| Nipocalimab_30mg_kg: Rozanolixzumb_7mg_kg | 0 | Some concerns | Low risk | No concerns | Major concerns | No concerns | Major concerns | Low |
| Nipocalimab_30mg_kg: Zilucoplan | 0 | No concerns | Low risk | No concerns | Major concerns | No concerns | Major concerns | Low |
| Nipocalimab_5mg_kg: Ravulizumab | 0 | No concerns | Low risk | No concerns | Major concerns | No concerns | Major concerns | Low |
| Nipocalimab_5mg_kg: Rituximab | 0 | No concerns | Low risk | No concerns | Major concerns | No concerns | Major concerns | Very low |
| Nipocalimab_5mg_kg: Rozanolixzumb_10mg_kg | 0 | No concerns | Low risk | No concerns | Major concerns | No concerns | Major concerns | Low |
| Nipocalimab_5mg_kg: Rozanolixzumb_7mg_kg | 0 | No concerns | Low risk | No concerns | Major concerns | No concerns | Major concerns | Low |
| Nipocalimab_5mg_kg: Zilucoplan | 0 | No concerns | Low risk | No concerns | Major concerns | No concerns | Major concerns | Low |
| Nipocalimab_60mg_kg: Ravulizumab | 0 | No concerns | Low risk | No concerns | Major concerns | No concerns | Major concerns | Low |
| Nipocalimab_60mg_kg: Rituximab | 0 | No concerns | Low risk | No concerns | Major concerns | No concerns | Major concerns | Low |
| Nipocalimab_60mg_kg: Rozanolixzumb_10mg_kg | 0 | No concerns | Low risk | No concerns | Major concerns | No concerns | Major concerns | Low |
| Nipocalimab_60mg_kg: Rozanolixzumb_7mg_kg | 0 | No concerns | Low risk | No concerns | Major concerns | No concerns | Major concerns | Low |
| Nipocalimab_60mg_kg: Zilucoplan | 0 | No concerns | Low risk | No concerns | Major concerns | No concerns | Major concerns | Low |
| Ravulizumab: Rituximab | 0 | No concerns | Low risk | No concerns | Major concerns | No concerns | Major concerns | Low |
| Ravulizumab: Rozanolixzumb_10mg_kg | 0 | Some concerns | Low risk | No concerns | Major concerns | No concerns | Major concerns | Low |
| Ravulizumab: Rozanolixzumb_7mg_kg | 0 | Some concerns | Low risk | No concerns | Major concerns | No concerns | Major concerns | Low |
| Ravulizumab: Zilucoplan | 0 | No concerns | Low risk | No concerns | Major concerns | No concerns | Major concerns | Low |
| Rituximab: Rozanolixzumb_10mg_kg | 0 | Some concerns | Low risk | No concerns | Major concerns | No concerns | Major concerns | Low |
| Rituximab: Rozanolixzumb_7mg_kg | 0 | Some concerns | Low risk | No concerns | Major concerns | No concerns | Major concerns | Low |
| Rituximab: Zilucoplan | 0 | No concerns | Low risk | No concerns | Major concerns | No concerns | Major concerns | Low |
| Rozanolixzumb_10mg_kg: Zilucoplan | 0 | Some concerns | Low risk | No concerns | Major concerns | No concerns | Major concerns | Low |
| Rozanolixzumb_7mg_kg: Zilucoplan | 0 | Some concerns | Low risk | No concerns | Major concerns | No concerns | Major concerns | Very low |

**Table S5: The events in the SAEs and all-cause Mortality of the included studies.**

| Study |  | SAEs  n/N | Specific Events | Mortality  n/N | Specific Events |
| --- | --- | --- | --- | --- | --- |
| **FcRn inhibitors** | | | | | |
| Bril et al. (2023) | placebo | 6/67 | COVID-19 pneumonia (1), myasthenia gravis crises (2) | 0/67 | - |
|  | Rozanolixzumb_7mg/kg | 5/64 | Severe vomiting (2), myasthenia gravis worsening (1), arthralgia, headache | 0/64 | - |
|  | Rozanolixzumb 10mg/kg | 7/69 | Severe diarrhea (2), myasthenia gravis worsening (2), upper abdominal pain, vomiting, oral herpes, metastatic squamous cell carcinoma, pruritus, deep vein thrombosis | 0/69 | - |
| Yan et al. (2022) | Placebo | 0/9 | - | 0/9 | - |
|  | Batoclimab_340mg | 0/10 | - | 0/11 | - |
|  | Batoclimab_680mg | 0/11 | - | 0/11 | - |
| Howard et al. (2019) | Placebo | 0/12 | - | 0/12 | - |
|  | Efgartigimod | 0/12 | - | 0/12 | - |
| Howard et al. (2021) | Placebo | 7/83 | Atrial fibrillation (1), Myocardial ischemia (1), Therapeutic product ineffective (1), Upper respiratory tract infection (1), Procedural pain (1), Spinal compression fracture (1), Spinal ligament ossification (1), Myasthenia gravis (1), Myasthenia gravis crisis (2) | 0/83 | - |
|  | Efgartigimod | 4/84 | Thrombocytosis (1), Rectal adenocarcinoma (1), Myasthenia gravis (1), depression (1) | 0/84 | - |
| NCT03772587 | Placebo | 2/14 | Ischemic stroke (1), Myasthenia gravis (1) | 0/14 | - |
|  | Nipocalimab_5mg/kg | 0/14 | - | 0/14 | - |
|  | Nipocalimab_30mg/kg | 1/13 | Musculoskeletal pain (1) | 0/13 | - |
|  | Nipocalimab_60mg/kg | 0/13 | - | 0/13 | - |
| **Complement inhibitors** | | | | | |
| Howard et al. (2017) | Placebo | 18/63 | Gastritis (1), General physical health deterioration (1), Cholecystitis acute (1), Gastroenteritis (1), Tonsillitis (1), Upper respiratory tract infection (2), Urinary tract infection bacterial (1), Varicella (1), Intentional overdose (1), Lymphocyte count decrease (1), Hyperglycemia (1), Myasthenia gravis (8), Apnea (1), Pulmonary embolism (1), Deep vein thrombosis (1) | 0/63 | - |
|  | Eculizumab | 9/62 | Lymphopenia (1), Intestinal perforation (1), Pyrexia (2), Bacteraemia (1), Diverticulitis (1), Endocarditis (1), Metastases to bone (1), Prostate cancer (1), Myasthenia gravis (5), Myasthenia gravis crisis (1) | 0/62 | - |
| Howard et al. (2023) | Placebo | 13/88 | Vomiting (1), COVID-19 (2), COVID-19 pneumonia (2), Herpes simplex meningoencephalitis (2), Metastases to meninges (1), Cerebral hemorrhage (1), Cerebrovascular accident (1), Myasthenia gravis (6), Hyperemesis gravidarum (1), Chronic obstructive pulmonary disease (1) | 1/88 | Cerebral hemorrhage (1) |
|  | Zilucoplan | 11/86 | Anemia (1), Aphthous ulcer (1), Oesophageal candidiasis (1), Oral candidiasis (1), COVID-19 (1), COVID-19 pneumonia (1), Pneumonia (1), Sepsis (1), Lipase increased (1), Basal cell carcinoma (1), Myasthenia gravis (3), Pulmonary embolism (1), Angioedema (1) | 1/86 | COVID-19(1) |
| NCT03920293 | Placebo | 14/89 | Enteritis (1), Herpes zoster (1), COVID-19(1), Cellulitis (1), Infusion-related reaction (1), Diabetic ketoacidosis (1), Spinal stenosis (1), Facial paresis (1), Myasthenia gravis (3), Trigeminal neuralgia (1), Nephrotic syndrome (1), Renal failure (1), Granuloma skin (1) | 0/89 | - |
|  | Ravulizumab | 20/86 | Congestive cardiomyopathy(1), Visual impairment(1), Dysphagia (1), Nausea (1), Asthenia (1), General physical health deterioration (1),Non-cardiac chest pain(1), COVID-19 pneumonia(3), Arthritis bacterial(1), Diverticulitis(1), Gangrene(1), Gastroenteritis viral(1), Herpes zoster(1), Infected skin ulcer(1), Pneumonia respiratory syncytial vira (1), Staphylococcal sepsis(1), Multiple fractures(1), Steroid diabetes(1), Intervertebral disc protrusi(1), Tendonitis(1), Squamous cell carcinoma of skin(1), Ureteral neoplasm(1), Transient ischemic attack(2), Cerebral hemorrhage(2), Myasthenia gravis crisis(1), Syncope(1), Suicide attempt(1), Dyspnea(1), Dyspnea exertional(1), Lung infiltration(1) | 2/86 | NA |
| **B-Cells inhibitors** |  |  |  |  |  |
| Hewett et al. (2018) | Placebo | 4/21 | Cholelithiasis (1), Nephrolithiasis (1), Sepsis (1), Myasthenia gravis (1), Aortic dissection rupture (1) | 1/21 | Severe sepsis (1) |
|  | Belimumab | 0/18 | - | 0/18 | - |
| Nowak et al. (2021) | Placebo | 14/27 | Anemia (1), Cardiac failure congestive (1), Coronary artery disease (2), Colonic obstruction (1), Diverticulum intestinal (1), Small intestinal obstruction (1), Chest pain (1), Pyrexia (1), Abscess neck (2), Cellulitis (1), Clostridium difficile colitis (1), Pneumonia (1), Sepsis (1), Spinal compression fracture (1), c (1), Prostate cancer (1), Worsening of Myasthenia gravis (4), Dyspnea (1), Pulmonary embolism (1), Micrographic skin surgery (1), Thrombosis (1) | 0/27 | - |
|  | Rituximab | 9/25 | Leukopenia (1), Hypersensitivity (1), Diverticulitis (1), Septic shock (1), Vascular pseudoaneurysm (1), Platelet count decreased (1), Hyper-glycaemia (1), Worsening of Myasthenia gravis (1), Psychotic disorder (1), Nephrolithiasis (1), Menorrhagia (1), Pulmonary embolism (1), Hypotension (1), Venous thrombosis limb (1) | 0/25 | - |
| Piehl et al. (2022) | Placebo | 4/22 | myocardial infarction with cardiac arrest in context of an MG exacerbation (1), bacterial septicemia during an MG crisis (1) | 0/22 | - |
|  | Rituximab | 6/25 | NA | 1/15 | Cardiac event (1) |
| NCT02565576 | Placebo | 4/22 | Myocardial ischemia (1), Hepatitis toxic (1), Brachial plexopathy (1), Myasthenia gravis (1), Radial nerve palsy (1) | 2/22 | NA |
|  | Iscalimab | 7/22 | Febrile neutropenia (1), Glaucoma (1), Abdominal pain upper (1), Constipation (1), Pyrexia (1), Influenza (2), Pneumonia (1), Myasthenia gravis (2), Myasthenia gravis crisis (1) | 0/22 | - |
| NCT04159805 | Placebo | 1/12 | Enteritis (1), Gastroenteritis (1) | 0/12 | - |
|  | Mezagitamab 300mg | 1/12 | Suicidal ideation (1) | 0/12 | - |
|  | Mezagitamab 600mg | 1/12 | Myasthenia gravis (1) | 0/12 | - |

**Table S6: The deviance information criteria (DIC) and I^2^ values of fixed-effects model and random-effects model.**

| Outcome measure | Estimate from network meta-analysis | | | | | | | |
| --- | --- | --- | --- | --- | --- | --- | --- | --- |
|  | Fixed-effects models | | | | Random-effects models | | | |
|  | Dbar | pD | DIC | I^2^(%) | Dbar | pD | DIC | I^2^(%) |
| MG-ADL | 16.79 | 16.76 | 33.55 | 5 | 16.72 | 16.69 | 33.42 | 4 |
| QMG | 16.72 | 16.68 | 33.40 | 4 | 16.81 | 16.78 | 33.58 | 5 |
| MGC | 16.75 | 16.71 | 33.45 | 4 | 16.85 | 16.82 | 33.67 | 5 |
| MG-QoL 15r | 24.37 | 24.08 | 48.45 | 2 | 24.75 | 24.70 | 49.46 | 3 |
| AEs | 26.51 | 19.09 | 45.60 | 2 | 26.95 | 19.72 | 46.67 | 4 |
| Headache | 26.60 | 19.10 | 46.70 | 2 | 26.72 | 19.60 | 46.32 | 3 |
| Diarrhea | 25.58 | 25.38 | 50.95 | 6 | 25.98 | 25.81 | 51.80 | 8 |
| Nausea | 25.49 | 25.29 | 50.79 | 6 | 25.68 | 25.53 | 51.21 | 7 |

Abbreviation: MG-ADL: Myasthenia Gravis Activities of Daily Living score; QMG: Quantitative Myasthenia Gravis score; MGC: Myasthenia Gravis Composite score; MG-QoL 15r: 15-item revised version of the Myasthenia Gravis Quality of Life score; AEs: adverse effects.

**Table S7: Network meta-analysis results of MG-ADL without studies of Nowak 2021.**

| **BAT 340mg** | 0.31 (-2, 2.6) | 2.18 (-0.52, 4.87) | 0.6 (-1.89, 3.08) | 1.92 (-0.44, 4.26) | 1 (-1.91, 3.94) | 2.39 (-0.99, 5.74) | 3.58 (-0.02, 7.19) | 0.41 (-2.95, 3.72) |
| --- | --- | --- | --- | --- | --- | --- | --- | --- |
| -0.31 (-2.6, 2) | **BAT 680mg** | 1.88 (-1.23, 4.99) | 0.3 (-2.64, 3.21) | 1.62 (-1.19, 4.43) | 0.7 (-2.6, 4.04) | 2.08 (-1.58, 5.82) | 3.28 (-0.62, 7.23) | 0.11 (-3.62, 3.79) |
| -2.18 (-4.87, 0.52) | -1.88 (-4.99, 1.23) | **BEL** | -1.58 (-3.66, 0.5) | -0.27 (-2.19, 1.64) | -1.18 (-3.76, 1.42) | 0.21 (-2.89, 3.29) | 1.41 (-1.94, 4.76) | -1.77 (-4.83, 1.29) |
| -0.6 (-3.08, 1.89) | -0.3 (-3.21, 2.64) | 1.58 (-0.5, 3.66) | **ECU** | 1.32 (-0.31, 2.92) | 0.4 (-1.97, 2.79) | 1.79 (-1.13, 4.75) | 2.99 (-0.18, 6.19) | -0.19 (-3.07, 2.71) |
| -1.92 (-4.26, 0.44) | -1.62 (-4.43, 1.19) | 0.27 (-1.64, 2.19) | -1.32 (-2.92, 0.31) | **EFG** | -0.92 (-3.15, 1.34) | 0.47 (-2.34, 3.3) | 1.67 (-1.39, 4.77) | -1.51 (-4.3, 1.25) |
| -1 (-3.94, 1.91) | -0.7 (-4.04, 2.6) | 1.18 (-1.42, 3.76) | -0.4 (-2.79, 1.97) | 0.92 (-1.34, 3.15) | **ISC** | 1.38 (-1.91, 4.69) | 2.59 (-0.95, 6.14) | -0.59 (-3.87, 2.66) |
| -2.39 (-5.74, 0.99) | -2.08 (-5.82, 1.58) | -0.21 (-3.29, 2.89) | -1.79 (-4.75, 1.13) | -0.47 (-3.3, 2.34) | -1.38 (-4.69, 1.91) | **MEZ 300mg** | 1.2 (-1.57, 3.95) | -1.98 (-5.69, 1.68) |
| -3.58 (-7.19, 0.02) | -3.28 (-7.23, 0.62) | -1.41 (-4.76, 1.94) | -2.99 (-6.19, 0.18) | -1.67 (-4.77, 1.39) | -2.59 (-6.14, 0.95) | -1.2 (-3.95, 1.57) | **MEZ 600mg** | -3.18 (-7.08, 0.7) |
| -0.41 (-3.72, 2.95) | -0.11 (-3.79, 3.62) | 1.77 (-1.29, 4.83) | 0.19 (-2.71, 3.07) | 1.51 (-1.25, 4.3) | 0.59 (-2.66, 3.87) | 1.98 (-1.68, 5.69) | 3.18 (-0.7, 7.08) | **NIP 30mg** |
| -1.8 (-4.93, 1.33) | -1.51 (-5.01, 2.01) | 0.38 (-2.44, 3.2) | -1.21 (-3.83, 1.44) | 0.11 (-2.38, 2.6) | -0.8 (-3.86, 2.26) | 0.58 (-2.91, 4.1) | 1.78 (-1.91, 5.5) | -1.4 (-3.52, 0.73) |
| -2.79 (-6.02, 0.42) | -2.5 (-6.12, 1.08) | -0.62 (-3.57, 2.33) | -2.21 (-4.94, 0.58) | -0.89 (-3.52, 1.77) | -1.8 (-4.94, 1.35) | -0.42 (-3.99, 3.22) | 0.79 (-3, 4.57) | **-2.39 (-4.7, -0.1)** |
| **-2.5 (-4.61, -0.36)** | -2.2 (-4.83, 0.46) | -0.31 (-1.95, 1.33) | **-1.9 (-3.17, -0.61)** | -0.58 (-1.56, 0.4) | -1.49 (-3.49, 0.53) | -0.11 (-2.72, 2.54) | 1.09 (-1.81, 4.01) | -2.09 (-4.67, 0.5) |
| -0.79 (-2.92, 1.34) | -0.5 (-3.13, 2.16) | 1.39 (-0.26, 3.03) | -0.2 (-1.47, 1.1) | **1.12 (0.13, 2.11)** | 0.21 (-1.8, 2.23) | 1.59 (-1.02, 4.24) | 2.79 (-0.11, 5.72) | -0.39 (-2.97, 2.2) |
| -1.29 (-4.07, 1.5) | -0.99 (-4.18, 2.22) | 0.89 (-1.56, 3.33) | -0.69 (-2.91, 1.5) | 0.62 (-1.45, 2.68) | -0.3 (-3, 2.42) | 1.1 (-2.08, 4.32) | 2.3 (-1.12, 5.72) | -0.89 (-4.02, 2.24) |
| 0.13 (-2.38, 2.63) | 0.43 (-2.55, 3.39) | **2.3 (0.17, 4.45)** | 0.71 (-1.13, 2.59) | **2.03 (0.36, 3.72)** | 1.12 (-1.28, 3.56) | 2.52 (-0.44, 5.48) | **3.71 (0.51, 6.92)** | 0.52 (-2.38, 3.45) |
| 0.1 (-2.41, 2.61) | 0.4 (-2.57, 3.36) | **2.27 (0.15, 4.41)** | 0.69 (-1.18, 2.58) | **2.01 (0.34, 3.69)** | 1.1 (-1.32, 3.53) | 2.48 (-0.46, 5.44) | **3.68 (0.47, 6.9)** | 0.5 (-2.42, 3.41) |
| -0.4 (-2.56, 1.76) | -0.11 (-2.77, 2.56) | **1.78 (0.09, 3.47)** | 0.19 (-1.14, 1.54) | **1.51 (0.46, 2.57)** | 0.59 (-1.45, 2.67) | 1.98 (-0.66, 4.66) | **3.18 (0.26, 6.14)** | 0 (-2.62, 2.62) |

| 1.8 (-1.33, 4.93) | 2.79 (-0.42, 6.02) | 2.5 (0.36, 4.61) | 0.79 (-1.34, 2.92) | 1.29 (-1.5, 4.07) | -0.13 (-2.63, 2.38) | -0.1 (-2.61, 2.41) |
| --- | --- | --- | --- | --- | --- | --- |
| 1.51 (-2.01, 5.01) | 2.5 (-1.08, 6.12) | 2.2 (-0.46, 4.83) | 0.5 (-2.16, 3.13) | 0.99 (-2.22, 4.18) | -0.43 (-3.39, 2.55) | -0.4 (-3.36, 2.57) |
| -0.38 (-3.2, 2.44) | 0.62 (-2.33, 3.57) | 0.31 (-1.33, 1.95) | -1.39 (-3.03, 0.26) | -0.89 (-3.33, 1.56) | -2.3 (-4.45, -0.17) | -2.27 (-4.41, -0.15) |
| 1.21 (-1.44, 3.83) | 2.21 (-0.58, 4.94) | 1.9 (0.61, 3.17) | 0.2 (-1.1, 1.47) | 0.69 (-1.5, 2.91) | -0.71 (-2.59, 1.13) | -0.69 (-2.58, 1.18) |
| -0.11 (-2.6, 2.38) | 0.89 (-1.77, 3.52) | 0.58 (-0.4, 1.56) | -1.12 (-2.11, -0.13) | -0.62 (-2.68, 1.45) | -2.03 (-3.72, -0.36) | -2.01 (-3.69, -0.34) |
| 0.8 (-2.26, 3.86) | 1.8 (-1.35, 4.94) | 1.49 (-0.53, 3.49) | -0.21 (-2.23, 1.8) | 0.3 (-2.42, 3) | -1.12 (-3.56, 1.28) | -1.1 (-3.53, 1.32) |
| -0.58 (-4.1, 2.91) | 0.42 (-3.22, 3.99) | 0.11 (-2.54, 2.72) | -1.59 (-4.24, 1.02) | -1.1 (-4.32, 2.08) | -2.52 (-5.48, 0.44) | -2.48 (-5.44, 0.46) |
| -1.78 (-5.5, 1.91) | -0.79 (-4.57, 3) | -1.09 (-4.01, 1.81) | -2.79 (-5.72, 0.11) | -2.3 (-5.72, 1.12) | -3.71 (-6.92, -0.51) | -3.68 (-6.9, -0.47) |
| 1.4 (-0.73, 3.52) | 2.39 (0.1, 4.7) | 2.09 (-0.5, 4.67) | 0.39 (-2.2, 2.97) | 0.89 (-2.24, 4.02) | -0.52 (-3.45, 2.38) | -0.5 (-3.41, 2.42) |
| **NIP 5mg** | 1 (-1, 2.98) | 0.69 (-1.59, 2.98) | -1.01 (-3.3, 1.29) | -0.51 (-3.41, 2.39) | -1.93 (-4.6, 0.72) | -1.91 (-4.56, 0.77) |
| -1 (-2.98, 1) | **NIP 60mg** | -0.31 (-2.75, 2.14) | -2.01 (-4.46, 0.44) | -1.51 (-4.53, 1.53) | -2.92 (-5.72, -0.13) | -2.9 (-5.7, -0.11) |
| -0.69 (-2.98, 1.59) | 0.31 (-2.14, 2.75) | **PLA** | -1.7 (-1.81, -1.59) | -1.2 (-3, 0.6) | -2.62 (-3.98, -1.27) | -2.59 (-3.94, -1.23) |
| 1.01 (-1.29, 3.3) | 2.01 (-0.44, 4.46) | 1.7 (1.59, 1.81) | **RAV** | 0.5 (-1.3, 2.3) | -0.92 (-2.28, 0.44) | -0.89 (-2.25, 0.47) |
| 0.51 (-2.39, 3.41) | 1.51 (-1.53, 4.53) | 1.2 (-0.6, 3) | -0.5 (-2.3, 1.3) | **RIT** | -1.41 (-3.69, 0.85) | -1.39 (-3.67, 0.88) |
| 1.93 (-0.72, 4.6) | **2.92 (0.13, 5.72)** | **2.62 (1.27, 3.98)** | 0.92 (-0.44, 2.28) | 1.41 (-0.85, 3.69) | **ROZ 10mg** | 0.03 (-1.33, 1.38) |
| 1.91 (-0.77, 4.56) | **2.9 (0.11, 5.7)** | **2.59 (1.23, 3.94)** | 0.89 (-0.47, 2.25) | 1.39 (-0.88, 3.67) | -0.03 (-1.38, 1.33) | **ROZ 7mg** |
| 1.4 (-0.93, 3.72) | 2.4 (-0.08, 4.87) | **2.09 (1.7, 2.48)** | 0.39 (-0.01, 0.79) | 0.89 (-0.95, 2.73) | -0.53 (-1.95, 0.88) | -0.5 (-1.91, 0.91) |

**Table S8: Network meta-analysis results of QMG without studies of Nowak 2021.**

| **BAT 340mg** | -0.81 (-4.6, 2.95) | 4.47 (-0.36, 9.24) | 3.3 (-0.46, 7.01) | 4.52 (0.68, 8.32) | 5.18 (0.71, 9.62) | 4.19 (-0.47, 8.88) | 7.19 (2.06, 12.36) | 5.88 (1.3, 10.46) |
| --- | --- | --- | --- | --- | --- | --- | --- | --- |
| 0.81 (-2.95, 4.6) | **BAT 680mg** | 5.28 (0.83, 9.74) | 4.11 (0.81, 7.37) | 5.33 (1.94, 8.69) | 5.99 (1.88, 10.07) | 5.01 (0.66, 9.34) | 8.02 (3.17, 12.8) | 6.7 (2.49, 10.91) |
| -4.47 (-9.24, 0.36) | **-5.28 (-9.74, -0.83)** | **BEL** | -1.17 (-4.37, 2.05) | 0.04 (-3.25, 3.37) | 0.7 (-3.32, 4.76) | -0.28 (-4.52, 3.97) | 2.73 (-2.02, 7.48) | 1.42 (-2.71, 5.56) |
| -3.3 (-7.01, 0.46) | **-4.11 (-7.37, -0.81)** | 1.17 (-2.05, 4.37) | **ECU** | 1.22 (-0.13, 2.56) | 1.88 (-0.79, 4.56) | 0.89 (-2.13, 3.95) | 3.91 (0.22, 7.57) | 2.58 (-0.23, 5.46) |
| **-4.52 (-8.32, -0.68)** | **-5.33 (-8.69, -1.94)** | -0.04 (-3.37, 3.25) | -1.22 (-2.56, 0.13) | **EFG** | 0.66 (-2.09, 3.44) | -0.32 (-3.44, 2.8) | 2.68 (-1.06, 6.44) | 1.37 (-1.59, 4.33) |
| **-5.18 (-9.62, -0.71)** | **-5.99 (-10.07, -1.88)** | -0.7 (-4.76, 3.32) | -1.88 (-4.56, 0.79) | -0.66 (-3.44, 2.09) | **ISC** | -0.98 (-4.86, 2.9) | 2.03 (-2.37, 6.44) | 0.71 (-3.07, 4.47) |
| -4.19 (-8.88, 0.47) | **-5.01 (-9.34, -0.66)** | 0.28 (-3.97, 4.52) | -0.89 (-3.95, 2.13) | 0.32 (-2.8, 3.44) | 0.98 (-2.9, 4.86) | **MEZ 300mg** | 3 (-0.65, 6.67) | 1.7 (-2.28, 5.66) |
| **-7.19 (-12.36, -2.06)** | **-8.02 (-12.8, -3.17)** | -2.73 (-7.48, 2.02) | **-3.91 (-7.57, -0.22)** | -2.68 (-6.44, 1.06) | -2.03 (-6.44, 2.37) | -3 (-6.67, 0.65) | **MEZ 600mg** | -1.32 (-5.84, 3.16) |
| **-5.88 (-10.46, -1.3)** | **-6.7 (-10.91, -2.49)** | -1.42 (-5.56, 2.71) | -2.58 (-5.46, 0.23) | -1.37 (-4.33, 1.59) | -0.71 (-4.47, 3.07) | -1.7 (-5.66, 2.28) | 1.32 (-3.16, 5.84) | **NIP 30mg** |
| **-6.49 (-11.1, -1.88)** | **-7.29 (-11.56, -3.07)** | -2.03 (-6.22, 2.18) | **-3.19 (-6.11, -0.27)** | -1.97 (-4.98, 1.04) | -1.33 (-5.14, 2.5) | -2.3 (-6.32, 1.75) | 0.72 (-3.84, 5.27) | -0.59 (-3.68, 2.46) |
| **-8.49 (-12.8, -4.17)** | **-9.3 (-13.23, -5.37)** | **-4.03 (-7.89, -0.14)** | **-5.2 (-7.62, -2.77)** | **-3.98 (-6.52, -1.43)** | -3.32 (-6.74, 0.11) | **-4.31 (-8.02, -0.59)** | -1.29 (-5.53, 2.95) | **-2.61 (-5.2, 0)** |
| **-6.3 (-9.95, -2.62)** | **-7.12 (-10.29, -3.9)** | -1.82 (-4.95, 1.27) | **-3 (-3.8, -2.2)** | **-1.78 (-2.88, -0.69)** | -1.12 (-3.66, 1.44) | -2.1 (-5.01, 0.83) | 0.9 (-2.69, 4.49) | -0.42 (-3.14, 2.35) |
| **-4.3 (-7.95, -0.62)** | **-5.11 (-8.29, -1.9)** | 0.18 (-2.95, 3.27) | **-1 (-1.8, -0.19)** | 0.22 (-0.88, 1.31) | 0.88 (-1.66, 3.44) | -0.1 (-3.01, 2.84) | 2.91 (-0.69, 6.51) | 1.59 (-1.15, 4.35) |
| **-4.98 (-9.54, -0.45)** | **-5.8 (-9.95, -1.62)** | -0.53 (-4.66, 3.61) | -1.7 (-4.5, 1.11) | -0.48 (-3.37, 2.42) | 0.18 (-3.56, 3.89) | -0.8 (-4.76, 3.2) | 2.21 (-2.27, 6.67) | 0.89 (-2.94, 4.72) |
| -1.55 (-5.86, 2.78) | -2.37 (-6.32, 1.61) | 2.93 (-0.98, 6.79) | 1.74 (-0.72, 4.21) | **2.96 (0.38, 5.54)** | **3.62 (0.17, 7.09)** | 2.64 (-1.08, 6.36) | **5.65 (1.38, 9.94)** | **4.33 (0.74, 7.94)** |
| -2.82 (-7.07, 1.44) | -3.65 (-7.52, 0.25) | 1.65 (-2.19, 5.44) | 0.48 (-1.88, 2.79) | 1.69 (-0.76, 4.14) | 2.35 (-1.02, 5.74) | 1.36 (-2.25, 4.99) | **4.38 (0.17, 8.57)** | 3.07 (-0.46, 6.56) |
| -3.36 (-7.04, 0.36) | **-4.16 (-7.38, -0.94)** | 1.12 (-2.04, 4.24) | -0.06 (-0.99, 0.87) | 1.16 (-0.03, 2.35) | 1.82 (-0.76, 4.42) | 0.84 (-2.1, 3.8) | **3.85 (0.24, 7.45)** | 2.53 (-0.24, 5.33) |

| 6.49 (1.88, 11.1) | 8.49 (4.17, 12.8) | 6.3 (2.62, 9.95) | 4.3 (0.62, 7.95) | 4.98 (0.45, 9.54) | 1.55 (-2.78, 5.86) | 2.82 (-1.44, 7.07) |
| --- | --- | --- | --- | --- | --- | --- |
| 7.29 (3.07, 11.56) | 9.3 (5.37, 13.23) | 7.12 (3.9, 10.29) | 5.11 (1.9, 8.29) | 5.8 (1.62, 9.95) | 2.37 (-1.61, 6.32) | 3.65 (-0.25, 7.52) |
| 2.03 (-2.18, 6.22) | 4.03 (0.14, 7.89) | 1.82 (-1.27, 4.95) | -0.18 (-3.27, 2.95) | 0.53 (-3.61, 4.66) | -2.93 (-6.79, 0.98) | -1.65 (-5.44, 2.19) |
| 3.19 (0.27, 6.11) | 5.2 (2.77, 7.62) | 3 (2.2, 3.8) | 1 (0.19, 1.8) | 1.7 (-1.11, 4.5) | -1.74 (-4.21, 0.72) | -0.48 (-2.79, 1.88) |
| 1.97 (-1.04, 4.98) | 3.98 (1.43, 6.52) | 1.78 (0.69, 2.88) | -0.22 (-1.31, 0.88) | 0.48 (-2.42, 3.37) | -2.96 (-5.54, -0.38) | -1.69 (-4.14, 0.76) |
| 1.33 (-2.5, 5.14) | 3.32 (-0.11, 6.74) | 1.12 (-1.44, 3.66) | -0.88 (-3.44, 1.66) | -0.18 (-3.89, 3.56) | -3.62 (-7.09, -0.17) | -2.35 (-5.74, 1.02) |
| 2.3 (-1.75, 6.32) | 4.31 (0.59, 8.02) | 2.1 (-0.83, 5.01) | 0.1 (-2.84, 3.01) | 0.8 (-3.2, 4.76) | -2.64 (-6.36, 1.08) | -1.36 (-4.99, 2.25) |
| -0.72 (-5.27, 3.84) | 1.29 (-2.95, 5.53) | -0.9 (-4.49, 2.69) | -2.91 (-6.51, 0.69) | -2.21 (-6.67, 2.27) | -5.65 (-9.94, -1.38) | -4.38 (-8.57, -0.17) |
| 0.59 (-2.46, 3.68) | 2.61 (0, 5.2) | 0.42 (-2.35, 3.14) | -1.59 (-4.35, 1.15) | -0.89 (-4.72, 2.94) | -4.33 (-7.94, -0.74) | -3.07 (-6.56, 0.46) |
| **NIP 5mg** | 2 (-0.67, 4.67) | -0.19 (-3.01, 2.62) | -2.19 (-5.02, 0.62) | -1.49 (-5.39, 2.37) | -4.94 (-8.58, -1.26) | -3.67 (-7.23, -0.07) |
| -2 (-4.67, 0.67) | **NIP 60mg** | -2.19 (-4.49, 0.1) | -4.2 (-6.5, -1.89) | -3.5 (-7.05, 0.03) | -6.94 (-10.22, -3.67) | -5.67 (-8.83, -2.5) |
| 0.19 (-2.62, 3.01) | 2.19 (-0.1, 4.49) | **PLA** | -2 (-2.13, -1.86) | -1.3 (-4.01, 1.38) | -4.74 (-7.07, -2.41) | -3.48 (-5.66, -1.27) |
| 2.19 (-0.62, 5.02) | **4.2 (1.89, 6.5)** | **2 (1.86, 2.13)** | **RAV** | 0.69 (-2.02, 3.38) | -2.74 (-5.08, -0.4) | -1.48 (-3.66, 0.74) |
| 1.49 (-2.37, 5.39) | 3.5 (-0.03, 7.05) | 1.3 (-1.38, 4.01) | -0.69 (-3.38, 2.02) | **RIT** | -3.43 (-6.99, 0.1) | -2.16 (-5.65, 1.29) |
| **4.94 (1.26, 8.58)** | **6.94 (3.67, 10.22)** | **4.74 (2.41, 7.07)** | **2.74 (0.4, 5.08)** | 3.43 (-0.1, 6.99) | **ROZ 10mg** | 1.27 (-1.31, 3.84) |
| **3.67 (0.07, 7.23)** | **5.67 (2.5, 8.83)** | **3.48 (1.27, 5.66)** | 1.48 (-0.74, 3.66) | 2.16 (-1.29, 5.65) | -1.27 (-3.84, 1.31) | **ROZ 7mg** |
| **3.14 (0.27, 5.99)** | **5.14 (2.79, 7.49)** | **2.94 (2.46, 3.42)** | **0.94 (0.44, 1.44)** | 1.64 (-1.11, 4.36) | -1.8 (-4.18, 0.59) | -0.53 (-2.76, 1.73) |

**Table S9: Network meta-analysis results of MGC without studies of Nowak 2021.**

| **BAT 340mg** | -0.81 (-4.6, 2.95) | 4.47 (-0.36, 9.24) | 3.3 (-0.46, 7.01) | 4.52 (0.68, 8.32) | 5.18 (0.71, 9.62) | 4.19 (-0.47, 8.88) | 7.19 (2.06, 12.36) | 5.88 (1.3, 10.46) |
| --- | --- | --- | --- | --- | --- | --- | --- | --- |
| 0.81 (-2.95, 4.6) | **BAT 680mg** | 5.28 (0.83, 9.74) | 4.11 (0.81, 7.37) | 5.33 (1.94, 8.69) | 5.99 (1.88, 10.07) | 5.01 (0.66, 9.34) | 8.02 (3.17, 12.8) | 6.7 (2.49, 10.91) |
| -4.47 (-9.24, 0.36) | **-5.28 (-9.74, -0.83)** | **BEL** | -1.17 (-4.37, 2.05) | 0.04 (-3.25, 3.37) | 0.7 (-3.32, 4.76) | -0.28 (-4.52, 3.97) | 2.73 (-2.02, 7.48) | 1.42 (-2.71, 5.56) |
| -3.3 (-7.01, 0.46) | **-4.11 (-7.37, -0.81)** | 1.17 (-2.05, 4.37) | **ECU** | 1.22 (-0.13, 2.56) | 1.88 (-0.79, 4.56) | 0.89 (-2.13, 3.95) | 3.91 (0.22, 7.57) | 2.58 (-0.23, 5.46) |
| **-4.52 (-8.32, -0.68)** | **-5.33 (-8.69, -1.94)** | -0.04 (-3.37, 3.25) | -1.22 (-2.56, 0.13) | **EFG** | 0.66 (-2.09, 3.44) | -0.32 (-3.44, 2.8) | 2.68 (-1.06, 6.44) | 1.37 (-1.59, 4.33) |
| **-5.18 (-9.62, -0.71)** | **-5.99 (-10.07, -1.88)** | -0.7 (-4.76, 3.32) | -1.88 (-4.56, 0.79) | -0.66 (-3.44, 2.09) | **ISC** | -0.98 (-4.86, 2.9) | 2.03 (-2.37, 6.44) | 0.71 (-3.07, 4.47) |
| -4.19 (-8.88, 0.47) | **-5.01 (-9.34, -0.66)** | 0.28 (-3.97, 4.52) | -0.89 (-3.95, 2.13) | 0.32 (-2.8, 3.44) | 0.98 (-2.9, 4.86) | **MEZ 300mg** | 3 (-0.65, 6.67) | 1.7 (-2.28, 5.66) |
| **-7.19 (-12.36, -2.06)** | **-8.02 (-12.8, -3.17)** | -2.73 (-7.48, 2.02) | **-3.91 (-7.57, -0.22)** | -2.68 (-6.44, 1.06) | -2.03 (-6.44, 2.37) | -3 (-6.67, 0.65) | **MEZ 600mg** | -1.32 (-5.84, 3.16) |
| **-5.88 (-10.46, -1.3)** | **-6.7 (-10.91, -2.49)** | -1.42 (-5.56, 2.71) | -2.58 (-5.46, 0.23) | -1.37 (-4.33, 1.59) | -0.71 (-4.47, 3.07) | -1.7 (-5.66, 2.28) | 1.32 (-3.16, 5.84) | **NIP 30mg** |
| **-6.49 (-11.1, -1.88)** | **-7.29 (-11.56, -3.07)** | -2.03 (-6.22, 2.18) | **-3.19 (-6.11, -0.27)** | -1.97 (-4.98, 1.04) | -1.33 (-5.14, 2.5) | -2.3 (-6.32, 1.75) | 0.72 (-3.84, 5.27) | -0.59 (-3.68, 2.46) |
| **-8.49 (-12.8, -4.17)** | **-9.3 (-13.23, -5.37)** | **-4.03 (-7.89, -0.14)** | **-5.2 (-7.62, -2.77)** | **-3.98 (-6.52, -1.43)** | -3.32 (-6.74, 0.11) | **-4.31 (-8.02, -0.59)** | -1.29 (-5.53, 2.95) | -2.61 (-5.2, 0) |
| **-6.3 (-9.95, -2.62)** | **-7.12 (-10.29, -3.9)** | -1.82 (-4.95, 1.27) | **-3 (-3.8, -2.2)** | **-1.78 (-2.88, -0.69)** | -1.12 (-3.66, 1.44) | -2.1 (-5.01, 0.83) | 0.9 (-2.69, 4.49) | -0.42 (-3.14, 2.35) |
| **-4.3 (-7.95, -0.62)** | **-5.11 (-8.29, -1.9)** | 0.18 (-2.95, 3.27) | **-1 (-1.8, -0.19)** | 0.22 (-0.88, 1.31) | 0.88 (-1.66, 3.44) | -0.1 (-3.01, 2.84) | 2.91 (-0.69, 6.51) | 1.59 (-1.15, 4.35) |
| **-4.98 (-9.54, -0.45)** | **-5.8 (-9.95, -1.62)** | -0.53 (-4.66, 3.61) | -1.7 (-4.5, 1.11) | -0.48 (-3.37, 2.42) | 0.18 (-3.56, 3.89) | -0.8 (-4.76, 3.2) | 2.21 (-2.27, 6.67) | 0.89 (-2.94, 4.72) |
| -1.55 (-5.86, 2.78) | -2.37 (-6.32, 1.61) | 2.93 (-0.98, 6.79) | 1.74 (-0.72, 4.21) | **2.96 (0.38, 5.54)** | **3.62 (0.17, 7.09)** | 2.64 (-1.08, 6.36) | **5.65 (1.38, 9.94)** | **4.33 (0.74, 7.94)** |
| -2.82 (-7.07, 1.44) | -3.65 (-7.52, 0.25) | 1.65 (-2.19, 5.44) | 0.48 (-1.88, 2.79) | 1.69 (-0.76, 4.14) | 2.35 (-1.02, 5.74) | 1.36 (-2.25, 4.99) | **4.38 (0.17, 8.57)** | 3.07 (-0.46, 6.56) |
| -3.36 (-7.04, 0.36) | **-4.16 (-7.38, -0.94)** | 1.12 (-2.04, 4.24) | -0.06 (-0.99, 0.87) | 1.16 (-0.03, 2.35) | 1.82 (-0.76, 4.42) | 0.84 (-2.1, 3.8) | **3.85 (0.24, 7.45)** | 2.53 (-0.24, 5.33) |

| 6.49 (1.88, 11.1) | 8.49 (4.17, 12.8) | 6.3 (2.62, 9.95) | 4.3 (0.62, 7.95) | 4.98 (0.45, 9.54) | 1.55 (-2.78, 5.86) | 2.82 (-1.44, 7.07) | 3.36 (-0.36, 7.04) |
| --- | --- | --- | --- | --- | --- | --- | --- |
| 7.29 (3.07, 11.56) | 9.3 (5.37, 13.23) | 7.12 (3.9, 10.29) | 5.11 (1.9, 8.29) | 5.8 (1.62, 9.95) | 2.37 (-1.61, 6.32) | 3.65 (-0.25, 7.52) | 4.16 (0.94, 7.38) |
| 2.03 (-2.18, 6.22) | 4.03 (0.14, 7.89) | 1.82 (-1.27, 4.95) | -0.18 (-3.27, 2.95) | 0.53 (-3.61, 4.66) | -2.93 (-6.79, 0.98) | -1.65 (-5.44, 2.19) | -1.12 (-4.24, 2.04) |
| 3.19 (0.27, 6.11) | 5.2 (2.77, 7.62) | 3 (2.2, 3.8) | 1 (0.19, 1.8) | 1.7 (-1.11, 4.5) | -1.74 (-4.21, 0.72) | -0.48 (-2.79, 1.88) | 0.06 (-0.87, 0.99) |
| 1.97 (-1.04, 4.98) | 3.98 (1.43, 6.52) | 1.78 (0.69, 2.88) | -0.22 (-1.31, 0.88) | 0.48 (-2.42, 3.37) | -2.96 (-5.54, -0.38) | -1.69 (-4.14, 0.76) | -1.16 (-2.35, 0.03) |
| 1.33 (-2.5, 5.14) | 3.32 (-0.11, 6.74) | 1.12 (-1.44, 3.66) | -0.88 (-3.44, 1.66) | -0.18 (-3.89, 3.56) | -3.62 (-7.09, -0.17) | -2.35 (-5.74, 1.02) | -1.82 (-4.42, 0.76) |
| 2.3 (-1.75, 6.32) | 4.31 (0.59, 8.02) | 2.1 (-0.83, 5.01) | 0.1 (-2.84, 3.01) | 0.8 (-3.2, 4.76) | -2.64 (-6.36, 1.08) | -1.36 (-4.99, 2.25) | -0.84 (-3.8, 2.1) |
| -0.72 (-5.27, 3.84) | 1.29 (-2.95, 5.53) | -0.9 (-4.49, 2.69) | -2.91 (-6.51, 0.69) | -2.21 (-6.67, 2.27) | -5.65 (-9.94, -1.38) | -4.38 (-8.57, -0.17) | -3.85 (-7.45, -0.24) |
| 0.59 (-2.46, 3.68) | 2.61 (0, 5.2) | 0.42 (-2.35, 3.14) | -1.59 (-4.35, 1.15) | -0.89 (-4.72, 2.94) | -4.33 (-7.94, -0.74) | -3.07 (-6.56, 0.46) | -2.53 (-5.33, 0.24) |
| **NIP 5mg** | 2 (-0.67, 4.67) | -0.19 (-3.01, 2.62) | -2.19 (-5.02, 0.62) | -1.49 (-5.39, 2.37) | -4.94 (-8.58, -1.26) | -3.67 (-7.23, -0.07) | -3.14 (-5.99, -0.27) |
| -2 (-4.67, 0.67) | **NIP 60mg** | -2.19 (-4.49, 0.1) | -4.2 (-6.5, -1.89) | -3.5 (-7.05, 0.03) | -6.94 (-10.22, -3.67) | -5.67 (-8.83, -2.5) | -5.14 (-7.49, -2.79) |
| 0.19 (-2.62, 3.01) | 2.19 (-0.1, 4.49) | **PLA** | -2 (-2.13, -1.86) | -1.3 (-4.01, 1.38) | -4.74 (-7.07, -2.41) | -3.48 (-5.66, -1.27) | -2.94 (-3.42, -2.46) |
| 2.19 (-0.62, 5.02) | **4.2 (1.89, 6.5)** | **2 (1.86, 2.13)** | **RAV** | 0.69 (-2.02, 3.38) | -2.74 (-5.08, -0.4) | -1.48 (-3.66, 0.74) | -0.94 (-1.44, -0.44) |
| 1.49 (-2.37, 5.39) | 3.5 (-0.03, 7.05) | 1.3 (-1.38, 4.01) | -0.69 (-3.38, 2.02) | **RIT** | -3.43 (-6.99, 0.1) | -2.16 (-5.65, 1.29) | -1.64 (-4.36, 1.11) |
| **4.94 (1.26, 8.58)** | **6.94 (3.67, 10.22)** | **4.74 (2.41, 7.07)** | **2.74 (0.4, 5.08)** | 3.43 (-0.1, 6.99) | **ROZ 10mg/kg** | 1.27 (-1.31, 3.84) | 1.8 (-0.59, 4.18) |
| **3.67 (0.07, 7.23)** | **5.67 (2.5, 8.83)** | **3.48 (1.27, 5.66)** | 1.48 (-0.74, 3.66) | 2.16 (-1.29, 5.65) | -1.27 (-3.84, 1.31) | **ROZ 7mg/kg** | 0.53 (-1.73, 2.76) |
| **3.14 (0.27, 5.99)** | **5.14 (2.79, 7.49)** | **2.94 (2.46, 3.42)** | **0.94 (0.44, 1.44)** | 1.64 (-1.11, 4.36) | -1.8 (-4.18, 0.59) | -0.53 (-2.76, 1.73) | **ZIL** |

**Table S10: Network meta-analysis results of AEs without studies of Nowak 2021.**

| **BEL** | 3.83 (0.34,42.62) | 2.10 (0.04,118.62) | 3.51 (0.20,62.35) | 5.71 (0.58,56.71) | 5.72 (0.53,61.72) | 6.67 (0.42,104.69) | 6.31 (0.06,637.75) | 7.91 (0.72,86.57) |
| --- | --- | --- | --- | --- | --- | --- | --- | --- |
| 0.26 (0.02,2.91) | **EFG** | 0.55 (0.02,16.40) | 0.92 (0.14,6.04) | 1.49 (0.72,3.11) | 1.49 (0.57,3.92) | 1.74 (0.32,9.44) | 1.65 (0.03,96.71) | 2.07 (0.76,5.61) |
| 0.48 (0.01,26.78) | 1.82 (0.06,54.25) | **BAT 680mg** | 1.67 (0.04,70.29) | 2.71 (0.10,74.72) | 2.72 (0.09,79.28) | 3.17 (0.08,121.57) | 3.00 (0.11,82.08) | 3.76 (0.13,110.74) |
| 0.29 (0.02,5.07) | 1.09 (0.17,7.20) | 0.60 (0.01,25.33) | **NIP 30mg** | 1.63 (0.29,9.26) | 1.63 (0.26,10.33) | 1.90 (0.19,19.14) | 1.80 (0.02,141.60) | 2.26 (0.35,14.55) |
| 0.18 (0.02,1.74) | 0.67 (0.32,1.40) | 0.37 (0.01,10.14) | 0.61 (0.11,3.49) | **PLA** | 1.00 (0.53,1.87) | 1.17 (0.25,5.35) | 1.10 (0.02,60.61) | 1.38 (0.70,2.73) |
| 0.17 (0.02,1.89) | 0.67 (0.25,1.76) | 0.37 (0.01,10.75) | 0.61 (0.10,3.89) | 1.00 (0.53,1.87) | **RAV** | 1.17 (0.22,6.05) | 1.10 (0.02,63.60) | 1.38 (0.55,3.48) |
| 0.15 (0.01,2.36) | 0.57 (0.11,3.11) | 0.32 (0.01,12.12) | 0.53 (0.05,5.30) | 0.86 (0.19,3.93) | 0.86 (0.17,4.45) | **RIT** | 0.95 (0.01,68.69) | 1.19 (0.22,6.28) |
| 0.16 (0.00,16.00) | 0.61 (0.01,35.56) | 0.33 (0.01,9.12) | 0.56 (0.01,43.67) | 0.91 (0.02,49.65) | 0.91 (0.02,52.15) | 1.06 (0.01,76.59) | **BAT 340mg** | 1.25 (0.02,72.73) |
| 0.13 (0.01,1.38) | 0.48 (0.18,1.32) | 0.27 (0.01,7.85) | 0.44 (0.07,2.86) | 0.72 (0.37,1.42) | 0.72 (0.29,1.82) | 0.84 (0.16,4.46) | 0.80 (0.01,46.36) | **ZIL** |
| 0.12 (0.01,2.12) | 0.45 (0.07,3.05) | 0.25 (0.01,10.55) | 0.41 (0.03,4.90) | 0.67 (0.11,3.93) | 0.67 (0.10,4.38) | 0.78 (0.08,8.06) | 0.74 (0.01,58.80) | 0.92 (0.14,6.16) |
| 0.11 (0.01,2.20) | 0.41 (0.05,3.34) | 0.23 (0.00,10.63) | 0.38 (0.06,2.52) | 0.61 (0.09,4.37) | 0.61 (0.08,4.82) | 0.71 (0.06,8.58) | 0.68 (0.01,58.49) | 0.85 (0.11,6.78) |
| **0.08 (0.01,0.94)** | **0.32 (0.11,0.94)** | 0.17 (0.01,5.27) | 0.29 (0.04,1.97) | 0.47 (0.21,1.06) | 0.47 (0.17,1.31) | 0.55 (0.10,3.09) | 0.52 (0.01,31.02) | 0.65 (0.23,1.88) |
| **0.08 (0.01,0.86)** | **0.29 (0.10,0.86)** | 0.16 (0.01,4.81) | 0.26 (0.04,1.79) | **0.43 (0.19,0.96)** | 0.43 (0.16,1.19) | 0.50 (0.09,2.81) | 0.48 (0.01,28.27) | 0.60 (0.21,1.71) |
| 0.05 (0.00,1.49) | 0.20 (0.02,2.53) | 0.11 (0.00,6.77) | 0.19 (0.02,1.98) | 0.31 (0.03,3.39) | 0.31 (0.03,3.68) | 0.36 (0.02,6.15) | 0.34 (0.00,36.09) | 0.42 (0.03,5.15) |
| **0.03 (0.00,0.86)** | 0.12 (0.01,1.46) | 0.07 (0.00,3.95) | 0.11 (0.01,2.11) | 0.18 (0.02,1.95) | 0.18 (0.02,2.12) | 0.21 (0.01,3.56) | 0.20 (0.00,21.11) | 0.25 (0.02,2.97) |

| 8.57 (0.47,155.90) | 9.35 (0.45,192.15) | 12.10 (1.06,137.97) | 13.27 (1.17,151.03) | 18.70 (0.67,520.07) | 31.42 (1.16,853.12) |
| --- | --- | --- | --- | --- | --- |
| 2.24 (0.33,15.28) | 2.44 (0.30,19.95) | 3.16 (1.06,9.43) | 3.47 (1.17,10.31) | 4.88 (0.39,60.48) | 8.21 (0.68,98.43) |
| 4.07 (0.09,174.85) | 4.44 (0.09,209.74) | 5.75 (0.19,174.44) | 6.30 (0.21,191.03) | 8.88 (0.15,534.10) | 14.93 (0.25,880.15) |
| 2.44 (0.20,29.27) | 2.67 (0.40,17.91) | 3.45 (0.51,23.45) | 3.78 (0.56,25.66) | 5.33 (0.51,56.24) | 8.96 (0.47,169.66) |
| 1.50 (0.25,8.84) | 1.64 (0.23,11.70) | 2.12 (0.94,4.76) | 2.32 (1.04,5.19) | 3.27 (0.29,36.31) | 5.50 (0.51,59.01) |
| 1.50 (0.23,9.84) | 1.64 (0.21,12.89) | 2.12 (0.76,5.89) | 2.32 (0.84,6.44) | 3.27 (0.27,39.32) | 5.50 (0.47,63.98) |
| 1.29 (0.12,13.32) | 1.40 (0.12,16.87) | 1.82 (0.32,10.18) | 1.99 (0.36,11.14) | 2.81 (0.16,48.37) | 4.71 (0.28,79.03) |
| 1.36 (0.02,108.37) | 1.48 (0.02,128.29) | 1.92 (0.03,114.02) | 2.10 (0.04,124.89) | 2.96 (0.03,316.63) | 4.98 (0.05,523.05) |
| 1.08 (0.16,7.24) | 1.18 (0.15,9.47) | 1.53 (0.53,4.40) | 1.68 (0.59,4.81) | 2.36 (0.19,28.82) | 3.97 (0.34,46.89) |
| **MEA 300mg** | 1.09 (0.08,15.43) | 1.41 (0.20,9.93) | 1.55 (0.22,10.86) | 2.18 (0.11,43.38) | 3.67 (0.32,41.59) |
| 0.92 (0.06,12.96) | **NIP 5mg** | 1.29 (0.15,10.86) | 1.42 (0.17,11.89) | 2.00 (0.16,25.11) | 3.36 (0.15,73.31) |
| 0.71 (0.10,4.98) | 0.77 (0.09,6.48) | **ROZ 7mg** | 1.10 (0.45,2.65) | 1.54 (0.12,19.56) | 2.60 (0.21,31.85) |
| 0.65 (0.09,4.53) | 0.70 (0.08,5.90) | 0.91 (0.38,2.21) | **ROZ 10mg** | 1.41 (0.11,17.82) | 2.37 (0.19,29.02) |
| 0.46 (0.02,9.11) | 0.50 (0.04,6.28) | 0.65 (0.05,8.20) | 0.71 (0.06,8.97) | **NIP 60mg** | 1.68 (0.06,49.34) |
| 0.27 (0.02,3.09) | 0.30 (0.01,6.49) | 0.39 (0.03,4.73) | 0.42 (0.03,5.17) | 0.60 (0.02,17.47) | **MEZ 600mg** |

**Table S11: Network meta-analysis results of Nausea without studies of Nowak 2021.**

| **BEL** | 4.53 (0.5, 137.41) | 3.03 (0.33, 91.86) | 1.88 (0.04, 107.5) | 4.99 (0.08, 542.62) | 10.4 (0.18, 1069.12) | 12.65 (0.47, 1107.08) | 4.38 (0.62, 121.05) | 5.57 (0.67, 166.71) |
| --- | --- | --- | --- | --- | --- | --- | --- | --- |
| 0.22 (0.01, 1.99) | **ECU** | 0.67 (0.17, 2.53) | 0.4 (0.01, 5.83) | 1.02 (0.03, 37.11) | 2.16 (0.06, 74.55) | 2.52 (0.19, 71.41) | 0.99 (0.38, 2.56) | 1.23 (0.37, 4.2) |
| 0.33 (0.01, 3.01) | 1.49 (0.4, 5.84) | **EFG** | 0.61 (0.02, 8.77) | 1.54 (0.04, 56.21) | 3.22 (0.09, 112.23) | 3.81 (0.28, 110.64) | 1.47 (0.58, 3.98) | 1.84 (0.56, 6.41) |
| 0.53 (0.01, 25.71) | 2.47 (0.17, 80.51) | 1.65 (0.11, 51.47) | **ISC** | 2.66 (0.03, 292.81) | 5.54 (0.08, 589.26) | 6.83 (0.19, 590.97) | 2.38 (0.21, 69.78) | 3 (0.23, 93.52) |
| 0.2 (0, 11.98) | 0.98 (0.03, 38.83) | 0.65 (0.02, 25.28) | 0.38 (0, 30.82) | **NIP 30mg/kg** | 2.07 (0.06, 74.84) | 2.4 (0.21, 74.57) | 0.97 (0.03, 34.35) | 1.21 (0.03, 46.24) |
| 0.1 (0, 5.68) | 0.46 (0.01, 17.2) | 0.31 (0.01, 11.54) | 0.18 (0, 12.96) | 0.48 (0.01, 16.34) | **NIP 5mg/kg** | 1.15 (0.11, 32.79) | 0.46 (0.01, 15.13) | 0.57 (0.02, 20.88) |
| 0.08 (0, 2.12) | 0.4 (0.01, 5.4) | 0.26 (0.01, 3.62) | 0.15 (0, 5.39) | 0.42 (0.01, 4.73) | 0.87 (0.03, 9.13) | **NIP 60mg/kg** | 0.39 (0.02, 4.47) | 0.49 (0.02, 6.35) |
| 0.23 (0.01, 1.61) | 1.01 (0.39, 2.62) | 0.68 (0.25, 1.72) | 0.42 (0.01, 4.86) | 1.03 (0.03, 34.2) | 2.18 (0.07, 67.82) | 2.54 (0.22, 65.08) | **PLA** | 1.25 (0.59, 2.74) |
| 0.18 (0.01, 1.5) | 0.81 (0.24, 2.73) | 0.54 (0.16, 1.79) | 0.33 (0.01, 4.42) | 0.83 (0.02, 29.63) | 1.74 (0.05, 58) | 2.04 (0.16, 57.45) | 0.8 (0.36, 1.71) | **RAV** |
| **0.07 (0, 0.91)** | 0.34 (0.04, 1.91) | 0.23 (0.03, 1.28) | 0.13 (0, 2.43) | 0.33 (0.01, 14.94) | 0.7 (0.01, 30.23) | 0.84 (0.04, 29.24) | 0.34 (0.05, 1.4) | 0.42 (0.05, 2.15) |
| 0.19 (0.01, 1.62) | 0.84 (0.24, 2.96) | 0.57 (0.15, 1.95) | 0.34 (0.01, 4.73) | 0.86 (0.02, 31.26) | 1.8 (0.05, 63) | 2.12 (0.16, 61.39) | 0.83 (0.36, 1.89) | 1.04 (0.34, 3.22) |
| **0.12 (0, 0.99)** | 0.53 (0.16, 1.78) | 0.36 (0.1, 1.17) | 0.22 (0.01, 2.91) | 0.54 (0.01, 19.6) | 1.14 (0.03, 39.41) | 1.33 (0.11, 37.45) | 0.53 (0.24, 1.09) | 0.66 (0.22, 1.91) |
| **0.04 (0, 0.51)** | 0.19 (0.02, 1.09) | **0.13 (0.01, 0.73)** | 0.07 (0, 1.4) | 0.19 (0, 8.92) | 0.4 (0.01, 17.84) | 0.48 (0.02, 17.53) | **0.19 (0.03, 0.78**) | 0.24 (0.03, 1.22) |

| 14.22 (1.1, 607.2) | 5.37 (0.62, 158.73) | 8.53 (1.01, 250.36) | 25.14 (1.95, 990.02) |
| --- | --- | --- | --- |
| 2.95 (0.52, 25.85) | 1.18 (0.34, 4.2) | 1.88 (0.56, 6.38) | 5.16 (0.92, 46.37) |
| 4.42 (0.78, 38.57) | 1.77 (0.51, 6.54) | 2.79 (0.86, 9.88) | 7.7 (1.38, 70.89) |
| 7.59 (0.41, 328.98) | 2.9 (0.21, 90.66) | 4.61 (0.34, 142.01) | 13.57 (0.71, 590.02) |
| 2.99 (0.07, 155.95) | 1.16 (0.03, 45.33) | 1.85 (0.05, 71.13) | 5.24 (0.11, 301.14) |
| 1.44 (0.03, 71.67) | 0.56 (0.02, 19.96) | 0.88 (0.03, 31.44) | 2.52 (0.06, 133.41) |
| 1.18 (0.03, 25.45) | 0.47 (0.02, 6.13) | 0.75 (0.03, 9.49) | 2.08 (0.06, 46.81) |
| 2.94 (0.72, 21.17) | 1.2 (0.53, 2.79) | 1.89 (0.92, 4.13) | 5.15 (1.28, 38.69) |
| 2.39 (0.46, 19.07) | 0.96 (0.31, 2.98) | 1.52 (0.52, 4.5) | 4.16 (0.82, 36.55) |
| **RIT** | 0.4 (0.05, 2.13) | 0.64 (0.08, 3.24) | 1.76 (0.15, 20.92) |
| 2.48 (0.47, 20.84) | **ROZ 10mg/kg** | 1.58 (0.8, 3.25) | 4.33 (0.83, 38.14) |
| 1.56 (0.31, 12.66) | 0.63 (0.31, 1.25) | **ROZ 7mg/kg** | 2.73 (0.54, 23.19) |
| 0.57 (0.05, 6.6) | 0.23 (0.03, 1.2) | 0.37 (0.04, 1.85) | **ZIL** |

**Table S12: Network meta-analysis results of Diarrhea without studies of Nowak 2021.**

| **BEL** | 4.31 (0.47, 108.75) | 2.9 (0.32, 70.66) | 1.81 (0.04, 87.91) | 5.13 (0.1, 659.29) | 10.61 (0.17, 1223.66) | 12.51 (0.47, 1296.4) | 4.19 (0.59, 93.7) | 5.34 (0.65, 128.38) |
| --- | --- | --- | --- | --- | --- | --- | --- | --- |
| 0.23 (0.01, 2.15) | **ECU** | 0.67 (0.17, 2.56) | 0.41 (0.01, 6.15) | 1.12 (0.03, 53.32) | 2.28 (0.06, 105.38) | 2.66 (0.19, 108.77) | 0.99 (0.38, 2.57) | 1.24 (0.36, 4.3) |
| 0.35 (0.01, 3.14) | 1.49 (0.39, 5.9) | **EFG** | 0.61 (0.02, 9.21) | 1.69 (0.04, 83.6) | 3.43 (0.09, 158.63) | 3.99 (0.28, 163.42) | 1.47 (0.58, 3.99) | 1.86 (0.56, 6.52) |
| 0.55 (0.01, 27.52) | 2.46 (0.16, 83.89) | 1.64 (0.11, 56.54) | **ISC** | 2.92 (0.04, 434.78) | 5.89 (0.07, 780.31) | 7.12 (0.19, 814.34) | 2.39 (0.2, 73.42) | 3.03 (0.22, 98.77) |
| 0.19 (0, 10.13) | 0.89 (0.02, 35.84) | 0.59 (0.01, 22.78) | 0.34 (0, 26.08) | **NIP 30mg/kg** | 2.02 (0.06, 66.82) | 2.31 (0.21, 68.59) | 0.88 (0.02, 30.74) | 1.11 (0.02, 42.1) |
| 0.09 (0, 6.03) | 0.44 (0.01, 17.43) | 0.29 (0.01, 11.39) | 0.17 (0, 13.6) | 0.5 (0.01, 17.66) | **NIP 5mg/kg** | 1.15 (0.11, 33.85) | 0.43 (0.01, 15.8) | 0.55 (0.01, 21.4) |
| 0.08 (0, 2.11) | 0.38 (0.01, 5.28) | 0.25 (0.01, 3.57) | 0.14 (0, 5.31) | 0.43 (0.01, 4.71) | 0.87 (0.03, 9.15) | **NIP 60mg/kg** | 0.38 (0.01, 4.3) | 0.47 (0.01, 6.1) |
| 0.24 (0.01, 1.7) | 1.01 (0.39, 2.64) | 0.68 (0.25, 1.71) | 0.42 (0.01, 5.11) | 1.14 (0.03, 50.03) | 2.31 (0.06, 94.48) | 2.64 (0.23, 96.7) | **PLA** | 1.26 (0.59, 2.74) |
| 0.19 (0.01, 1.53) | 0.81 (0.23, 2.77) | 0.54 (0.15, 1.79) | 0.33 (0.01, 4.54) | 0.9 (0.02, 43.53) | 1.83 (0.05, 78.37) | 2.12 (0.16, 81.54) | 0.8 (0.37, 1.69) | **RAV** |
| **0.08 (0, 0.96)** | 0.35 (0.04, 1.96) | 0.23 (0.03, 1.28) | 0.13 (0, 2.56) | 0.37 (0.01, 20.81) | 0.76 (0.01, 40.1) | 0.9 (0.04, 40.52) | 0.35 (0.05, 1.42) | 0.43 (0.05, 2.22) |
| 0.2 (0.01, 1.67) | 0.84 (0.23, 2.99) | 0.56 (0.15, 1.98) | 0.34 (0.01, 4.91) | 0.95 (0.02, 44.98) | 1.91 (0.05, 85.46) | 2.23 (0.17, 89.4) | 0.83 (0.35, 1.91) | 1.04 (0.33, 3.26) |
| 0.12 (0.01, 1.01) | 0.53 (0.15, 1.77) | 0.36 (0.1, 1.18) | 0.22 (0.01, 3) | 0.6 (0.02, 27.19) | 1.21 (0.03, 53.1) | 1.4 (0.11, 53.1) | 0.53 (0.23, 1.09) | 0.66 (0.22, 1.93) |
| 0.04 (0, 0.54) | 0.19 (0.02, 1.07) | **0.13 (0.01, 0.72)** | 0.07 (0, 1.42) | 0.21 (0, 11.9) | 0.43 (0.01, 22.54) | 0.51 (0.02, 23.26) | **0.19 (0.03, 0.78)** | 0.24 (0.03, 1.21) |

| 13.18 (1.04, 459.24) | 5.11 (0.6, 124.36) | 8.05 (0.99, 197.82) | 23.52 (1.87, 856.12) |
| --- | --- | --- | --- |
| 2.89 (0.51, 24.96) | 1.19 (0.33, 4.27) | 1.88 (0.57, 6.49) | 5.22 (0.93, 44.2) |
| 4.34 (0.78, 37.51) | 1.78 (0.5, 6.49) | 2.81 (0.85, 9.88) | 7.8 (1.4, 67.56) |
| 7.48 (0.39, 334.42) | 2.93 (0.2, 97.89) | 4.65 (0.33, 152.33) | 13.35 (0.7, 647.33) |
| 2.67 (0.05, 144.9) | 1.06 (0.02, 41.07) | 1.67 (0.04, 63.44) | 4.75 (0.08, 251.27) |
| 1.32 (0.02, 71.87) | 0.52 (0.01, 20.51) | 0.83 (0.02, 31.99) | 2.33 (0.04, 131.14) |
| 1.11 (0.02, 24.6) | 0.45 (0.01, 6.01) | 0.71 (0.02, 9.32) | 1.96 (0.04, 45.71) |
| 2.89 (0.7, 20.93) | 1.2 (0.52, 2.83) | 1.89 (0.92, 4.26) | 5.19 (1.29, 36.45) |
| 2.32 (0.45, 18.84) | 0.96 (0.31, 2.99) | 1.52 (0.52, 4.51) | 4.18 (0.82, 33.83) |
| **RIT** | 0.41 (0.05, 2.17) | 0.65 (0.08, 3.36) | 1.8 (0.16, 18.96) |
| 2.43 (0.46, 20.43) | **ROZ 10mg/kg** | 1.58 (0.8, 3.27) | 4.37 (0.84, 35.84) |
| 1.53 (0.3, 12.42) | 0.63 (0.31, 1.25) | **ROZ 7mg/kg** | 2.75 (0.55, 21.67) |
| 0.56 (0.05, 6.26) | 0.23 (0.03, 1.19) | 0.36 (0.05, 1.83) | **ZIL** |

**Table S13: Network meta-analysis results of Nausea without studies of Nowak 2021.**

| **BEL** | 0.19 (0.01, 2.01) | 0.17 (0.01, 1.74) | 0.34 (0.01, 6.76) | 0.22 (0.01, 1.82) | 0.23 (0.01, 2.35) | 0.99 (0.02, 45.16) | 0.34 (0.01, 3.98) | 0.22 (0.01, 2.83) |
| --- | --- | --- | --- | --- | --- | --- | --- | --- |
| 5.16 (0.5, 163.63) | **ECU** | 0.87 (0.24, 3.18) | 1.78 (0.24, 17.26) | 1.1 (0.44, 2.78) | 1.17 (0.33, 4.26) | 5.11 (0.56, 144.87) | 1.78 (0.44, 7.83) | 1.16 (0.25, 5.51) |
| 5.98 (0.58, 182.35) | 1.16 (0.31, 4.21) | **EFG** | 2.04 (0.28, 19.73) | 1.27 (0.52, 3.19) | 1.35 (0.37, 4.96) | 5.88 (0.65, 166.09) | 2.05 (0.5, 9.08) | 1.35 (0.29, 6.36) |
| 2.96 (0.15, 120.53) | 0.56 (0.06, 4.2) | 0.49 (0.05, 3.63) | **ISC** | 0.63 (0.08, 3.72) | 0.67 (0.07, 5.02) | 2.92 (0.16, 114.23) | 1 (0.1, 8.41) | 0.65 (0.06, 5.85) |
| 4.61 (0.55, 130.75) | 0.91 (0.36, 2.26) | 0.79 (0.31, 1.93) | 1.6 (0.27, 13.17) | PLA | 1.06 (0.43, 2.65) | 4.49 (0.64, 116.67) | 1.59 (0.55, 5.26) | 1.05 (0.3, 3.79) |
| 4.44 (0.43, 136.45) | 0.85 (0.23, 3.05) | 0.74 (0.2, 2.67) | 1.5 (0.2, 14.96) | 0.94 (0.38, 2.33) | RAV | 4.32 (0.48, 120.52) | 1.51 (0.38, 6.56) | 0.99 (0.21, 4.69) |
| 1.01 (0.02, 47.8) | 0.2 (0.01, 1.79) | 0.17 (0.01, 1.53) | 0.34 (0.01, 6.16) | 0.22 (0.01, 1.57) | 0.23 (0.01, 2.09) | RIT | 0.35 (0.01, 3.57) | 0.23 (0.01, 2.5) |
| 2.92 (0.25, 95.64) | 0.56 (0.13, 2.29) | 0.49 (0.11, 1.99) | 1 (0.12, 10.48) | 0.63 (0.19, 1.81) | 0.66 (0.15, 2.64) | 2.87 (0.28, 86.35) | ROZ 10mg/kg | 0.66 (0.2, 1.91) |
| 4.54 (0.35, 151.33) | 0.86 (0.18, 4.04) | 0.74 (0.16, 3.49) | 1.54 (0.17, 17.38) | 0.95 (0.26, 3.35) | 1.01 (0.21, 4.72) | 4.42 (0.4, 141.09) | 1.52 (0.52, 4.97) | ROZ 7mg/kg |

**Table S14: Network meta-analysis results of MG-ADL without studies of Piehl 2022.**

| **BAT 340mg** | 0.3 (-1.98, 2.57) | 2.18 (-0.47, 4.87) | 0.6 (-1.88, 3.08) | 1.92 (-0.41, 4.25) | 0.99 (-1.91, 3.94) | 2.41 (-0.98, 5.78) | 3.6 (-0.01, 7.23) | 0.42 (-2.94, 3.68) |
| --- | --- | --- | --- | --- | --- | --- | --- | --- |
| -0.3 (-2.57, 1.98) | **BAT 680mg** | 1.89 (-1.23, 5) | 0.31 (-2.65, 3.24) | 1.63 (-1.2, 4.45) | 0.71 (-2.61, 4.01) | 2.12 (-1.6, 5.83) | 3.31 (-0.65, 7.24) | 0.12 (-3.56, 3.77) |
| -2.18 (-4.87, 0.47) | -1.89 (-5, 1.23) | **BEL** | -1.59 (-3.69, 0.49) | -0.27 (-2.19, 1.63) | -1.19 (-3.78, 1.38) | 0.22 (-2.89, 3.29) | 1.41 (-1.95, 4.77) | -1.78 (-4.79, 1.19) |
| -0.6 (-3.08, 1.88) | -0.31 (-3.24, 2.65) | 1.59 (-0.49, 3.69) | **ECU** | 1.32 (-0.28, 2.91) | 0.4 (-1.98, 2.76) | 1.81 (-1.1, 4.72) | 3 (-0.18, 6.2) | -0.19 (-3.03, 2.63) |
| -1.92 (-4.25, 0.41) | -1.63 (-4.45, 1.2) | 0.27 (-1.63, 2.19) | -1.32 (-2.91, 0.28) | **EFG** | -0.92 (-3.14, 1.3) | 0.49 (-2.31, 3.29) | 1.68 (-1.42, 4.79) | -1.51 (-4.23, 1.18) |
| -0.99 (-3.94, 1.91) | -0.71 (-4.01, 2.61) | 1.19 (-1.38, 3.78) | -0.4 (-2.76, 1.98) | 0.92 (-1.3, 3.14) | **ISC** | 1.41 (-1.9, 4.71) | 2.6 (-0.96, 6.13) | -0.59 (-3.82, 2.65) |
| -2.41 (-5.78, 0.98) | -2.12 (-5.83, 1.6) | -0.22 (-3.29, 2.89) | -1.81 (-4.72, 1.1) | -0.49 (-3.29, 2.31) | -1.41 (-4.71, 1.9) | **MEZ 300mg** | 1.19 (-1.56, 3.96) | -2 (-5.66, 1.63) |
| -3.6 (-7.23, 0.01) | -3.31 (-7.24, 0.65) | -1.41 (-4.77, 1.95) | -3 (-6.2, 0.18) | -1.68 (-4.79, 1.42) | -2.6 (-6.13, 0.96) | -1.19 (-3.96, 1.56) | **MEZ 600mg** | -3.19 (-7.07, 0.66) |
| -0.42 (-3.68, 2.94) | -0.12 (-3.77, 3.56) | 1.78 (-1.19, 4.79) | 0.19 (-2.63, 3.03) | 1.51 (-1.18, 4.23) | 0.59 (-2.65, 3.82) | 2 (-1.63, 5.66) | 3.19 (-0.66, 7.07) | **NIP 30mg** |
| -1.8 (-4.9, 1.3) | -1.51 (-4.98, 2) | 0.39 (-2.4, 3.17) | -1.21 (-3.81, 1.38) | 0.12 (-2.35, 2.58) | -0.79 (-3.84, 2.2) | 0.61 (-2.85, 4.08) | 1.8 (-1.91, 5.48) | -1.39 (-3.52, 0.72) |
| -2.81 (-5.99, 0.41) | -2.51 (-6.09, 1.09) | -0.61 (-3.56, 2.29) | -2.21 (-4.96, 0.53) | -0.89 (-3.5, 1.72) | -1.81 (-4.97, 1.35) | -0.4 (-3.97, 3.19) | 0.79 (-3.01, 4.6) | -2.4 (-4.69, -0.1) |
| **-2.5 (-4.61, -0.38)** | -2.21 (-4.84, 0.43) | -0.31 (-1.94, 1.34) | **-1.9 (-3.17, -0.62)** | -0.58 (-1.55, 0.4) | -1.5 (-3.5, 0.5) | -0.09 (-2.71, 2.53) | 1.11 (-1.83, 4.04) | -2.09 (-4.63, 0.42) |
| -0.8 (-2.91, 1.33) | -0.5 (-3.13, 2.14) | 1.39 (-0.25, 3.04) | -0.2 (-1.48, 1.08) | **1.12 (0.14, 2.11)** | 0.2 (-1.8, 2.21) | 1.61 (-1.01, 4.23) | 2.81 (-0.13, 5.74) | -0.39 (-2.93, 2.12) |
| -1.3 (-4.09, 1.47) | -1 (-4.18, 2.2) | 0.89 (-1.52, 3.33) | -0.7 (-2.9, 1.5) | 0.62 (-1.41, 2.66) | -0.3 (-2.99, 2.38) | 1.12 (-2.11, 4.27) | 2.31 (-1.13, 5.73) | -0.89 (-3.99, 2.17) |
| 0.12 (-2.4, 2.67) | 0.42 (-2.56, 3.39) | **2.31 (0.17, 4.45)** | 0.72 (-1.14, 2.59) | **2.04 (0.37, 3.72)** | 1.12 (-1.3, 3.54) | 2.53 (-0.41, 5.49) | **3.73 (0.49, 6.96)** | 0.53 (-2.36, 3.41) |
| 0.09 (-2.43, 2.62) | 0.39 (-2.59, 3.35) | **2.28 (0.16, 4.41)** | 0.69 (-1.17, 2.55) | **2.01 (0.33, 3.69)** | 1.09 (-1.33, 3.5) | 2.51 (-0.45, 5.45) | **3.69 (0.46, 6.92)** | 0.49 (-2.4, 3.35) |
| -0.42 (-2.56, 1.75) | -0.12 (-2.78, 2.54) | **1.78 (0.1, 3.47)** | 0.19 (-1.15, 1.53) | **1.51 (0.46, 2.57)** | 0.59 (-1.46, 2.64) | 2.01 (-0.65, 4.64) | **3.2 (0.24, 6.15)** | 0.01 (-2.56, 2.54) |

| 1.8 (-1.3, 4.9) | 2.81 (-0.41, 5.99) | 2.5 (0.38, 4.61) | 0.8 (-1.33, 2.91) | 1.3 (-1.47, 4.09) | -0.12 (-2.67, 2.4) | -0.09 (-2.62, 2.43) | 0.42 (-1.75, 2.56) |
| --- | --- | --- | --- | --- | --- | --- | --- |
| 1.51 (-2, 4.98) | 2.51 (-1.09, 6.09) | 2.21 (-0.43, 4.84) | 0.5 (-2.14, 3.13) | 1 (-2.2, 4.18) | -0.42 (-3.39, 2.56) | -0.39 (-3.35, 2.59) | 0.12 (-2.54, 2.78) |
| -0.39 (-3.17, 2.4) | 0.61 (-2.29, 3.56) | 0.31 (-1.34, 1.94) | -1.39 (-3.04, 0.25) | -0.89 (-3.33, 1.52) | -2.31 (-4.45, -0.17) | -2.28 (-4.41, -0.16) | -1.78 (-3.47, -0.1) |
| 1.21 (-1.38, 3.81) | 2.21 (-0.53, 4.96) | 1.9 (0.62, 3.17) | 0.2 (-1.08, 1.48) | 0.7 (-1.5, 2.9) | -0.72 (-2.59, 1.14) | -0.69 (-2.55, 1.17) | -0.19 (-1.53, 1.15) |
| -0.12 (-2.58, 2.35) | 0.89 (-1.72, 3.5) | 0.58 (-0.4, 1.55) | -1.12 (-2.11, -0.14) | -0.62 (-2.66, 1.41) | -2.04 (-3.72, -0.37) | -2.01 (-3.69, -0.33) | -1.51 (-2.57, -0.46) |
| 0.79 (-2.2, 3.84) | 1.81 (-1.35, 4.97) | 1.5 (-0.5, 3.5) | -0.2 (-2.21, 1.8) | 0.3 (-2.38, 2.99) | -1.12 (-3.54, 1.3) | -1.09 (-3.5, 1.33) | -0.59 (-2.64, 1.46) |
| -0.61 (-4.08, 2.85) | 0.4 (-3.19, 3.97) | 0.09 (-2.53, 2.71) | -1.61 (-4.23, 1.01) | -1.12 (-4.27, 2.11) | -2.53 (-5.49, 0.41) | -2.51 (-5.45, 0.45) | -2.01 (-4.64, 0.65) |
| -1.8 (-5.48, 1.91) | -0.79 (-4.6, 3.01) | -1.11 (-4.04, 1.83) | -2.81 (-5.74, 0.13) | -2.31 (-5.73, 1.13) | -3.73 (-6.96, -0.49) | -3.69 (-6.92, -0.46) | -3.2 (-6.15, -0.24) |
| 1.39 (-0.72, 3.52) | 2.4 (0.1, 4.69) | 2.09 (-0.42, 4.63) | 0.39 (-2.12, 2.93) | 0.89 (-2.17, 3.99) | -0.53 (-3.41, 2.36) | -0.49 (-3.35, 2.4) | -0.01 (-2.54, 2.56) |
| **NIP 5mg** | 1.01 (-0.97, 2.97) | 0.7 (-1.58, 2.97) | -1 (-3.28, 1.27) | -0.5 (-3.38, 2.37) | -1.92 (-4.6, 0.74) | -1.89 (-4.55, 0.77) | -1.39 (-3.7, 0.9) |
| -1.01 (-2.97, 0.97) | **NIP 60mg** | -0.31 (-2.75, 2.13) | -2.01 (-4.45, 0.43) | -1.51 (-4.52, 1.52) | -2.93 (-5.73, -0.13) | -2.9 (-5.69, -0.1) | -2.4 (-4.87, 0.06) |
| -0.7 (-2.97, 1.58) | 0.31 (-2.13, 2.75) | **PLA** | -1.7 (-1.81, -1.59) | -1.2 (-3, 0.59) | -2.62 (-3.99, -1.26) | -2.59 (-3.95, -1.23) | -2.09 (-2.48, -1.7) |
| 1 (-1.27, 3.28) | 2.01 (-0.43, 4.45) | **1.7 (1.59, 1.81)** | **RAV** | 0.5 (-1.3, 2.29) | -0.92 (-2.3, 0.44) | -0.89 (-2.26, 0.47) | -0.39 (-0.79, 0.01) |
| 0.5 (-2.37, 3.38) | 1.51 (-1.52, 4.52) | 1.2 (-0.59, 3) | -0.5 (-2.29, 1.3) | **RIT** | -1.42 (-3.68, 0.84) | -1.38 (-3.65, 0.86) | -0.89 (-2.73, 0.95) |
| 1.92 (-0.74, 4.6) | **2.93 (0.13, 5.73)** | **2.62 (1.26, 3.99)** | 0.92 (-0.44, 2.3) | 1.42 (-0.84, 3.68) | **ROZ 10mg** | 0.03 (-1.33, 1.39) | 0.53 (-0.88, 1.96) |
| 1.89 (-0.77, 4.55) | **2.9 (0.1, 5.69)** | **2.59 (1.23, 3.95)** | 0.89 (-0.47, 2.26) | 1.38 (-0.86, 3.65) | -0.03 (-1.39, 1.33) | **ROZ 7mg** | 0.5 (-0.91, 1.92) |
| 1.39 (-0.9, 3.7) | 2.4 (-0.06, 4.87) | **2.09 (1.7, 2.48)** | 0.39 (-0.01, 0.79) | 0.89 (-0.95, 2.73) | -0.53 (-1.96, 0.88) | -0.5 (-1.92, 0.91) | **ZIL** |

**Table S15: Network meta-analysis results of QMG without studies of Piehl 2022.**

| **BAT 340mg** | 0.3 (-1.98, 2.57) | 2.18 (-0.49, 4.88) | 0.59 (-1.88, 3.07) | 1.91 (-0.42, 4.25) | 0.99 (-1.93, 3.9) | 2.4 (-0.96, 5.74) | 3.6 (-0.03, 7.2) | 0.41 (-2.91, 3.73) |
| --- | --- | --- | --- | --- | --- | --- | --- | --- |
| -0.3 (-2.57, 1.98) | **BAT 680mg** | 1.88 (-1.23, 5.01) | 0.28 (-2.66, 3.23) | 1.6 (-1.2, 4.43) | 0.68 (-2.62, 3.99) | 2.09 (-1.64, 5.81) | 3.29 (-0.64, 7.22) | 0.12 (-3.58, 3.77) |
| -2.18 (-4.88, 0.49) | -1.88 (-5.01, 1.23) | **BEL** | -1.6 (-3.7, 0.51) | -0.28 (-2.22, 1.65) | -1.2 (-3.8, 1.42) | 0.21 (-2.9, 3.29) | 1.42 (-1.96, 4.76) | -1.77 (-4.8, 1.28) |
| -0.59 (-3.07, 1.88) | -0.28 (-3.23, 2.66) | 1.6 (-0.51, 3.7) | **ECU** | 1.32 (-0.3, 2.93) | 0.39 (-1.98, 2.8) | 1.81 (-1.1, 4.74) | 3.01 (-0.2, 6.19) | -0.17 (-3.06, 2.67) |
| -1.91 (-4.25, 0.42) | -1.6 (-4.43, 1.2) | 0.28 (-1.65, 2.22) | -1.32 (-2.93, 0.3) | **EFG** | -0.93 (-3.16, 1.32) | 0.49 (-2.31, 3.28) | 1.69 (-1.39, 4.76) | -1.49 (-4.22, 1.26) |
| -0.99 (-3.9, 1.93) | -0.68 (-3.99, 2.62) | 1.2 (-1.42, 3.8) | -0.39 (-2.8, 1.98) | 0.93 (-1.32, 3.16) | **ISC** | 1.41 (-1.89, 4.71) | 2.61 (-0.91, 6.14) | -0.56 (-3.85, 2.7) |
| -2.4 (-5.74, 0.96) | -2.09 (-5.81, 1.64) | -0.21 (-3.29, 2.9) | -1.81 (-4.74, 1.1) | -0.49 (-3.28, 2.31) | -1.41 (-4.71, 1.89) | **MEZ 300mg** | 1.2 (-1.54, 3.97) | -1.98 (-5.65, 1.73) |
| -3.6 (-7.2, 0.03) | -3.29 (-7.22, 0.64) | -1.42 (-4.76, 1.96) | -3.01 (-6.19, 0.2) | -1.69 (-4.76, 1.39) | -2.61 (-6.14, 0.91) | -1.2 (-3.97, 1.54) | **MEZ 600mg** | -3.18 (-7.06, 0.71) |
| -0.41 (-3.73, 2.91) | -0.12 (-3.77, 3.58) | 1.77 (-1.28, 4.8) | 0.17 (-2.67, 3.06) | 1.49 (-1.26, 4.22) | 0.56 (-2.7, 3.85) | 1.98 (-1.73, 5.65) | 3.18 (-0.71, 7.06) | **NIP 30mg** |
| -1.8 (-4.91, 1.31) | -1.5 (-5, 2) | 0.39 (-2.43, 3.21) | -1.21 (-3.82, 1.41) | 0.11 (-2.4, 2.58) | -0.82 (-3.86, 2.24) | 0.59 (-2.92, 4.1) | 1.79 (-1.92, 5.51) | -1.38 (-3.49, 0.73) |
| -2.79 (-6.02, 0.43) | -2.49 (-6.11, 1.11) | -0.61 (-3.56, 2.34) | -2.21 (-4.95, 0.56) | -0.89 (-3.53, 1.74) | -1.82 (-4.94, 1.37) | -0.4 (-4.01, 3.2) | 0.8 (-3.03, 4.62) | **-2.39 (-4.66, -0.08)** |
| **-2.49 (-4.61, -0.37)** | -2.18 (-4.83, 0.44) | -0.3 (-1.96, 1.37) | **-1.9 (-3.17, -0.62)** | -0.58 (-1.56, 0.4) | -1.51 (-3.5, 0.52) | -0.09 (-2.72, 2.54) | 1.11 (-1.81, 4.01) | -2.07 (-4.62, 0.49) |
| -0.79 (-2.91, 1.33) | -0.48 (-3.13, 2.15) | 1.4 (-0.26, 3.08) | -0.2 (-1.48, 1.09) | **1.12 (0.13, 2.11)** | 0.2 (-1.8, 2.23) | 1.61 (-1.03, 4.24) | 2.81 (-0.11, 5.72) | -0.37 (-2.92, 2.19) |
| -1.29 (-4.08, 1.5) | -0.99 (-4.2, 2.21) | 0.9 (-1.54, 3.34) | -0.71 (-2.9, 1.52) | 0.62 (-1.43, 2.64) | -0.3 (-3.01, 2.42) | 1.1 (-2.08, 4.27) | 2.31 (-1.11, 5.74) | -0.88 (-4, 2.25) |
| 0.14 (-2.39, 2.65) | 0.45 (-2.56, 3.4) | **2.33 (0.17, 4.48)** | 0.72 (-1.14, 2.6) | **2.04 (0.35, 3.74)** | 1.12 (-1.3, 3.55) | 2.54 (-0.43, 5.51) | **3.74 (0.5, 6.94)** | 0.55 (-2.33, 3.47) |
| 0.12 (-2.44, 2.61) | 0.42 (-2.57, 3.36) | **2.29 (0.14, 4.45)** | 0.7 (-1.17, 2.56) | **2.01 (0.33, 3.69)** | 1.09 (-1.33, 3.52) | 2.51 (-0.46, 5.46) | **3.71 (0.47, 6.9)** | 0.52 (-2.35, 3.41) |
| -0.4 (-2.57, 1.75) | -0.09 (-2.77, 2.57) | **1.79 (0.08, 3.5)** | 0.19 (-1.15, 1.53) | **1.51 (0.46, 2.57)** | 0.58 (-1.45, 2.65) | 2 (-0.65, 4.65) | **3.2 (0.27, 6.13)** | 0.02 (-2.57, 2.61) |

| 1.8 (-1.31, 4.91) | 2.79 (-0.43, 6.02) | 2.49 (0.37, 4.61) | 0.79 (-1.33, 2.91) | 1.29 (-1.5, 4.08) | -0.14 (-2.65, 2.39) | -0.12 (-2.61, 2.44) | 0.4 (-1.75, 2.57) |
| --- | --- | --- | --- | --- | --- | --- | --- |
| 1.5 (-2, 5) | 2.49 (-1.11, 6.11) | 2.18 (-0.44, 4.83) | 0.48 (-2.15, 3.13) | 0.99 (-2.21, 4.2) | -0.45 (-3.4, 2.56) | -0.42 (-3.36, 2.57) | 0.09 (-2.57, 2.77) |
| -0.39 (-3.21, 2.43) | 0.61 (-2.34, 3.56) | 0.3 (-1.37, 1.96) | -1.4 (-3.08, 0.26) | -0.9 (-3.34, 1.54) | -2.33 (-4.48, -0.17) | -2.29 (-4.45, -0.14) | -1.79 (-3.5, -0.08) |
| 1.21 (-1.41, 3.82) | 2.21 (-0.56, 4.95) | 1.9 (0.62, 3.17) | 0.2 (-1.09, 1.48) | 0.71 (-1.52, 2.9) | -0.72 (-2.6, 1.14) | -0.7 (-2.56, 1.17) | -0.19 (-1.53, 1.15) |
| -0.11 (-2.58, 2.4) | 0.89 (-1.74, 3.53) | 0.58 (-0.4, 1.56) | **-1.12 (-2.11, -0.13)** | -0.62 (-2.64, 1.43) | -2.04 (-3.74, -0.35) | -2.01 (-3.69, -0.33) | -1.51 (-2.57, -0.46) |
| 0.82 (-2.24, 3.86) | 1.82 (-1.37, 4.94) | 1.51 (-0.52, 3.5) | -0.2 (-2.23, 1.8) | 0.3 (-2.42, 3.01) | -1.12 (-3.55, 1.3) | -1.09 (-3.52, 1.33) | -0.58 (-2.65, 1.45) |
| -0.59 (-4.1, 2.92) | 0.4 (-3.2, 4.01) | 0.09 (-2.54, 2.72) | -1.61 (-4.24, 1.03) | -1.1 (-4.27, 2.08) | -2.54 (-5.51, 0.43) | -2.51 (-5.46, 0.46) | -2 (-4.65, 0.65) |
| -1.79 (-5.51, 1.92) | -0.8 (-4.62, 3.03) | -1.11 (-4.01, 1.81) | -2.81 (-5.72, 0.11) | -2.31 (-5.74, 1.11) | -3.74 (-6.94, -0.5) | -3.71 (-6.9, -0.47) | -3.2 (-6.13, -0.27) |
| 1.38 (-0.73, 3.49) | 2.39 (0.08, 4.66) | 2.07 (-0.49, 4.62) | 0.37 (-2.19, 2.92) | 0.88 (-2.25, 4) | -0.55 (-3.47, 2.33) | -0.52 (-3.41, 2.35) | -0.02 (-2.61, 2.57) |
| **NIP 5mg** | 1 (-1, 2.99) | 0.69 (-1.61, 2.97) | -1.01 (-3.31, 1.27) | -0.51 (-3.43, 2.41) | -1.94 (-4.6, 0.74) | -1.91 (-4.58, 0.76) | -1.4 (-3.73, 0.93) |
| -1 (-2.99, 1) | **NIP 60mg** | -0.32 (-2.74, 2.13) | -2.02 (-4.44, 0.44) | -1.51 (-4.54, 1.54) | -2.94 (-5.73, -0.14) | -2.91 (-5.68, -0.13) | -2.4 (-4.88, 0.07) |
| -0.69 (-2.97, 1.61) | 0.32 (-2.13, 2.74) | **PLA** | -1.7 (-1.81, -1.59) | -1.2 (-2.99, 0.6) | -2.63 (-3.98, -1.25) | -2.6 (-3.94, -1.23) | -2.09 (-2.48, -1.7) |
| 1.01 (-1.27, 3.31) | 2.02 (-0.44, 4.44) | **1.7 (1.59, 1.81)** | **RAV** | 0.5 (-1.29, 2.3) | -0.93 (-2.29, 0.45) | -0.9 (-2.25, 0.48) | -0.39 (-0.8, 0.02) |
| 0.51 (-2.41, 3.43) | 1.51 (-1.54, 4.54) | 1.2 (-0.6, 2.99) | -0.5 (-2.3, 1.29) | **RIT** | -1.43 (-3.69, 0.84) | -1.4 (-3.67, 0.85) | -0.89 (-2.73, 0.95) |
| 1.94 (-0.74, 4.6) | **2.94 (0.14, 5.73)** | **2.63 (1.25, 3.98)** | 0.93 (-0.45, 2.29) | 1.43 (-0.84, 3.69) | **ROZ 10mg** | 0.03 (-1.33, 1.39) | 0.54 (-0.89, 1.95) |
| 1.91 (-0.76, 4.58) | **2.91 (0.13, 5.68)** | **2.6 (1.23, 3.94)** | 0.9 (-0.48, 2.25) | 1.4 (-0.85, 3.67) | -0.03 (-1.39, 1.33) | **ROZ 7mg** | 0.51 (-0.91, 1.91) |
| 1.4 (-0.93, 3.73) | 2.4 (-0.07, 4.88) | **2.09 (1.7, 2.48)** | 0.39 (-0.02, 0.8) | 0.89 (-0.95, 2.73) | -0.54 (-1.95, 0.89) | -0.51 (-1.91, 0.91) | **ZIL** |

**Table S16: Network meta-analysis results of AEs without studies of Piehl 2022.**

| **BEL** | 2.10 (0.04,118.65) | 3.83 (0.34,42.62) | 3.51 (0.20,62.35) | 5.71 (0.58,56.71) | 5.72 (0.53,61.72) | 6.31 (0.06,638.27) | 7.91 (0.72,86.57) | 8.57 (0.47,155.90) |
| --- | --- | --- | --- | --- | --- | --- | --- | --- |
| 0.48 (0.01,26.78) | **BAT 680mg** | 1.82 (0.06,54.27) | 1.67 (0.04,70.31) | 2.71 (0.10,74.73) | 2.72 (0.09,79.30) | 3.00 (0.11,82.10) | 3.76 (0.13,110.77) | 4.07 (0.09,174.88) |
| 0.26 (0.02,2.91) | 0.55 (0.02,16.40) | **EFG** | 0.92 (0.14,6.04) | 1.49 (0.72,3.11) | 1.49 (0.57,3.92) | 1.65 (0.03,96.79) | 2.07 (0.76,5.61) | 2.24 (0.33,15.28) |
| 0.29 (0.02,5.07) | 0.60 (0.01,25.34) | 1.09 (0.17,7.20) | **NIP 30mg** | 1.63 (0.29,9.26) | 1.63 (0.26,10.33) | 1.80 (0.02,141.72) | 2.26 (0.35,14.55) | 2.44 (0.20,29.27) |
| 0.18 (0.02,1.74) | 0.37 (0.01,10.14) | 0.67 (0.32,1.40) | 0.61 (0.11,3.49) | **PLA** | 1.00 (0.53,1.87) | 1.10 (0.02,60.67) | 1.38 (0.70,2.73) | 1.50 (0.25,8.84) |
| 0.17 (0.02,1.89) | 0.37 (0.01,10.75) | 0.67 (0.25,1.76) | 0.61 (0.10,3.89) | 1.00 (0.53,1.87) | **RAV** | 1.10 (0.02,63.66) | 1.38 (0.55,3.48) | 1.50 (0.23,9.84) |
| 0.16 (0.00,16.01) | 0.33 (0.01,9.13) | 0.61 (0.01,35.59) | 0.56 (0.01,43.71) | 0.90 (0.02,49.69) | 0.91 (0.02,52.20) | **BAT 340mg** | 1.25 (0.02,72.79) | 1.36 (0.02,108.45) |
| 0.13 (0.01,1.38) | 0.27 (0.01,7.85) | 0.48 (0.18,1.32) | 0.44 (0.07,2.86) | 0.72 (0.37,1.42) | 0.72 (0.29,1.82) | 0.80 (0.01,46.41) | **ZIL** | 1.08 (0.16,7.24) |
| 0.12 (0.01,2.12) | 0.25 (0.01,10.55) | 0.45 (0.07,3.05) | 0.41 (0.03,4.90) | 0.67 (0.11,3.93) | 0.67 (0.10,4.38) | 0.74 (0.01,58.85) | 0.92 (0.14,6.16) | **MEZ 300mg** |
| 0.11 (0.01,2.20) | 0.23 (0.00,10.63) | 0.41 (0.05,3.34) | 0.38 (0.06,2.52) | 0.61 (0.09,4.37) | 0.61 (0.08,4.82) | 0.68 (0.01,58.54) | 0.85 (0.11,6.78) | 0.92 (0.06,12.96) |
| 0.06 (0.00,3.23) | 0.13 (0.00,13.21) | 0.23 (0.01,6.48) | 0.21 (0.01,8.44) | 0.35 (0.01,8.90) | 0.35 (0.01,9.46) | 0.38 (0.00,66.39) | 0.48 (0.02,13.21) | 0.52 (0.01,21.01) |
| **0.08 (0.01,0.94)** | 0.17 (0.01,5.28) | **0.32 (0.11,0.94)** | 0.29 (0.04,1.97) | 0.47 (0.21,1.06) | 0.47 (0.17,1.31) | 0.52 (0.01,31.04) | 0.65 (0.23,1.88) | 0.71 (0.10,4.98) |
| **0.08 (0.01,0.86)** | 0.16 (0.01,4.81) | **0.29 (0.10,0.86)** | 0.26 (0.04,1.79) | **0.43 (0.19,0.96)** | 0.43 (0.16,1.19) | 0.48 (0.01,28.30) | 0.60 (0.21,1.71) | 0.65 (0.09,4.53) |
| 0.05 (0.00,1.49) | 0.11 (0.00,6.77) | 0.20 (0.02,2.53) | 0.19 (0.02,1.98) | 0.31 (0.03,3.39) | 0.31 (0.03,3.68) | 0.34 (0.00,36.12) | 0.42 (0.03,5.15) | 0.46 (0.02,9.11) |
| **0.03 (0.00,0.86)** | 0.07 (0.00,3.95) | 0.12 (0.01,1.46) | 0.11 (0.01,2.11) | 0.18 (0.02,1.95) | 0.18 (0.02,2.12) | 0.20 (0.00,21.13) | 0.25 (0.02,2.97) | 0.27 (0.02,3.09) |

| 9.35 (0.45,192.15) | 16.49 (0.31,879.01) | 12.10 (1.06,137.97) | 13.27 (1.17,151.03) | 18.70 (0.67,520.07) | 31.42 (1.16,853.12) |
| --- | --- | --- | --- | --- | --- |
| 4.44 (0.09,209.78) | 7.84 (0.08,811.48) | 5.75 (0.19,174.48) | 6.30 (0.21,191.08) | 8.88 (0.15,534.20) | 14.93 (0.25,880.32) |
| 2.44 (0.30,19.95) | 4.31 (0.15,120.20) | 3.16 (1.06,9.43) | 3.47 (1.17,10.31) | 4.88 (0.39,60.48) | 8.21 (0.68,98.43) |
| 2.67 (0.40,17.91) | 4.70 (0.12,186.86) | 3.45 (0.51,23.45) | 3.78 (0.56,25.66) | 5.33 (0.51,56.24) | 8.96 (0.47,169.66) |
| 1.64 (0.23,11.70) | 2.89 (0.11,74.19) | 2.12 (0.94,4.76) | 2.32 (1.04,5.19) | 3.27 (0.29,36.31) | 5.50 (0.51,59.01) |
| 1.64 (0.21,12.89) | 2.89 (0.11,78.73) | 2.12 (0.76,5.89) | 2.32 (0.84,6.44) | 3.27 (0.27,39.32) | 5.50 (0.47,63.98) |
| 1.48 (0.02,128.38) | 2.61 (0.02,453.14) | 1.92 (0.03,114.12) | 2.10 (0.04,124.99) | 2.96 (0.03,316.85) | 4.98 (0.05,523.41) |
| 1.18 (0.15,9.47) | 2.09 (0.08,57.50) | 1.53 (0.53,4.40) | 1.68 (0.59,4.81) | 2.36 (0.19,28.82) | 3.97 (0.34,46.89) |
| 1.09 (0.08,15.43) | 1.92 (0.05,77.81) | 1.41 (0.20,9.93) | 1.55 (0.22,10.86) | 2.18 (0.11,43.38) | 3.67 (0.32,41.59) |
| **NIP 5mg** | 1.76 (0.04,78.55) | 1.29 (0.15,10.86) | 1.42 (0.17,11.89) | 2.00 (0.16,25.11) | 3.36 (0.15,73.31) |
| 0.57 (0.01,25.24) | **RIT** | 0.73 (0.03,20.83) | 0.80 (0.03,22.81) | 1.13 (0.02,64.50) | 1.91 (0.03,106.26) |
| 0.77 (0.09,6.48) | 1.36 (0.05,38.67) | **ROZ 7mg** | 1.10 (0.45,2.65) | 1.54 (0.12,19.56) | 2.60 (0.21,31.85) |
| 0.70 (0.08,5.90) | 1.24 (0.04,35.24) | 0.91 (0.38,2.21) | **ROZ 10mg** | 1.41 (0.11,17.82) | 2.37 (0.19,29.02) |
| 0.50 (0.04,6.28) | 0.88 (0.02,50.18) | 0.65 (0.05,8.20) | 0.71 (0.06,8.97) | **NIP 60mg** | 1.68 (0.06,49.34) |
| 0.30 (0.01,6.49) | 0.52 (0.01,29.27) | 0.39 (0.03,4.73) | 0.42 (0.03,5.17) | 0.60 (0.02,17.47) | **MEZ 600mg** |

**Table S17: Network meta-analysis results of Diarrhea without studies of Piehl 2022.**

| **BEL** | 4.37 (0.47, 114.91) | 2.9 (0.31, 80.59) | 1.83 (0.04, 105.47) | 5.06 (0.08, 491.69) | 10.39 (0.17, 1036.79) | 12.49 (0.46, 1109.4) | 4.19 (0.59, 102.05) | 5.37 (0.63, 145.94) |
| --- | --- | --- | --- | --- | --- | --- | --- | --- |
| 0.23 (0.01, 2.11) | **ECU** | 0.67 (0.17, 2.52) | 0.41 (0.01, 6.02) | 1.07 (0.03, 44.03) | 2.22 (0.06, 88.75) | 2.56 (0.18, 91.69) | 0.98 (0.38, 2.54) | 1.24 (0.37, 4.24) |
| 0.34 (0.01, 3.21) | 1.49 (0.4, 5.82) | **EFG** | 0.62 (0.02, 8.94) | 1.61 (0.04, 66.93) | 3.3 (0.08, 136.67) | 3.86 (0.27, 143.04) | 1.46 (0.58, 3.89) | 1.84 (0.56, 6.43) |
| 0.55 (0.01, 28.28) | 2.42 (0.17, 86.15) | 1.62 (0.11, 55.69) | **ISC** | 2.71 (0.03, 350.78) | 5.63 (0.07, 713.18) | 6.76 (0.18, 740.6) | 2.32 (0.19, 73.71) | 2.99 (0.22, 98.4) |
| 0.2 (0, 12.97) | 0.93 (0.02, 37.7) | 0.62 (0.01, 24.53) | 0.37 (0, 30.29) | **NIP 30mg/kg** | 2.08 (0.06, 73.32) | 2.39 (0.2, 73.67) | 0.92 (0.02, 33.12) | 1.17 (0.03, 45.06) |
| 0.1 (0, 5.82) | 0.45 (0.01, 17.93) | 0.3 (0.01, 11.91) | 0.18 (0, 13.61) | 0.48 (0.01, 17.33) | **NIP 5mg/kg** | 1.13 (0.11, 36.09) | 0.45 (0.01, 15.78) | 0.56 (0.01, 21.57) |
| 0.08 (0, 2.19) | 0.39 (0.01, 5.45) | 0.26 (0.01, 3.64) | 0.15 (0, 5.42) | 0.42 (0.01, 4.92) | 0.89 (0.03, 9.47) | **NIP 60mg/kg** | 0.39 (0.01, 4.55) | 0.48 (0.01, 6.33) |
| 0.24 (0.01, 1.69) | 1.02 (0.39, 2.66) | 0.68 (0.26, 1.72) | 0.43 (0.01, 5.14) | 1.09 (0.03, 40.97) | 2.24 (0.06, 82.15) | 2.59 (0.22, 85.19) | **PLA** | 1.26 (0.59, 2.76) |
| 0.19 (0.01, 1.6) | 0.81 (0.24, 2.74) | 0.54 (0.16, 1.79) | 0.33 (0.01, 4.52) | 0.86 (0.02, 35.19) | 1.78 (0.05, 72.66) | 2.06 (0.16, 72.98) | 0.79 (0.36, 1.7) | **RAV** |
| 0.13 (0, 2.05) | 0.6 (0.06, 4.54) | 0.4 (0.04, 3.03) | 0.24 (0.01, 5.31) | 0.64 (0.01, 34.23) | 1.3 (0.02, 73.21) | 1.57 (0.06, 72.28) | 0.6 (0.08, 3.5) | 0.75 (0.08, 5.24) |
| 0.2 (0.01, 1.69) | 0.86 (0.24, 3.01) | 0.58 (0.16, 2) | 0.35 (0.01, 5) | 0.92 (0.02, 37.2) | 1.9 (0.05, 75.19) | 2.21 (0.16, 76.4) | 0.85 (0.36, 1.92) | 1.06 (0.34, 3.31) |
| 0.12 (0, 1.04) | 0.54 (0.16, 1.79) | 0.36 (0.1, 1.18) | 0.22 (0.01, 2.99) | 0.58 (0.01, 22.82) | 1.19 (0.03, 47.38) | 1.38 (0.1, 48.41) | 0.53 (0.24, 1.1) | 0.67 (0.22, 1.95) |
| **0.04 (0, 0.53)** | 0.19 (0.02, 1.06) | **0.13 (0.01, 0.69)** | 0.07 (0, 1.42) | 0.2 (0, 9.86) | 0.41 (0.01, 20.16) | 0.49 (0.02, 20.22) | **0.19 (0.03, 0.76)** | 0.24 (0.03, 1.2) |

| 7.55 (0.49, 317.69) | 5.09 (0.59, 133.26) | 8.03 (0.96, 211.22) | 24.16 (1.89, 872.1) |
| --- | --- | --- | --- |
| 1.67 (0.22, 15.75) | 1.17 (0.33, 4.2) | 1.85 (0.56, 6.41) | 5.24 (0.95, 45.68) |
| 2.5 (0.33, 23.68) | 1.74 (0.5, 6.39) | 2.76 (0.85, 9.76) | 7.82 (1.44, 70.15) |
| 4.22 (0.19, 196.98) | 2.82 (0.2, 97) | 4.48 (0.33, 151.47) | 13.34 (0.7, 657) |
| 1.57 (0.03, 95.17) | 1.09 (0.03, 43.5) | 1.73 (0.04, 68.95) | 5.09 (0.1, 288.46) |
| 0.77 (0.01, 42.61) | 0.53 (0.01, 20.5) | 0.84 (0.02, 31.89) | 2.46 (0.05, 132.16) |
| 0.64 (0.01, 15.71) | 0.45 (0.01, 6.13) | 0.72 (0.02, 9.63) | 2.06 (0.05, 46.47) |
| 1.68 (0.29, 13.22) | 1.18 (0.52, 2.79) | 1.88 (0.91, 4.22) | 5.23 (1.31, 38.71) |
| 1.34 (0.19, 11.98) | 0.94 (0.3, 2.95) | 1.5 (0.51, 4.53) | 4.2 (0.83, 34.77) |
| **RIT** | 0.7 (0.08, 5.04) | 1.12 (0.12, 7.76) | 3.19 (0.26, 43.57) |
| 1.42 (0.2, 12.86) | **ROZ 10mg/kg** | 1.58 (0.8, 3.27) | 4.47 (0.85, 37.81) |
| 0.89 (0.13, 8.01) | 0.63 (0.31, 1.26) | **ROZ 7mg/kg** | 2.79 (0.55, 23.34) |
| 0.31 (0.02, 3.86) | 0.22 (0.03, 1.18) | 0.36 (0.04, 1.81) | **ZIL** |

**Table S18: Network meta-analysis results of Nausea without studies of Piehl 2022.**

| **BEL** | 4.25 (0.47, 111.86) | 2.89 (0.32, 73.89) | 1.81 (0.03, 84.02) | 5.22 (0.11, 505.05) | 10.3 (0.17, 991) | 12.34 (0.49, 1039.05) | 4.1 (0.6, 96.63) | 5.26 (0.64, 127.22) |
| --- | --- | --- | --- | --- | --- | --- | --- | --- |
| 0.24 (0.01, 2.12) | **ECU** | 0.68 (0.17, 2.57) | 0.41 (0.01, 5.91) | 1.11 (0.04, 48.33) | 2.23 (0.06, 91.49) | 2.61 (0.19, 91.01) | 0.99 (0.38, 2.57) | 1.25 (0.37, 4.26) |
| 0.35 (0.01, 3.16) | 1.47 (0.39, 5.82) | **EFG** | 0.61 (0.02, 8.87) | 1.64 (0.05, 71.88) | 3.27 (0.09, 137.17) | 3.86 (0.29, 138.09) | 1.45 (0.58, 3.89) | 1.83 (0.55, 6.43) |
| 0.55 (0.01, 28.9) | 2.42 (0.17, 90.25) | 1.64 (0.11, 61.08) | **ISC** | 2.91 (0.04, 364.4) | 5.73 (0.08, 706.95) | 6.91 (0.2, 756.98) | 2.37 (0.2, 78.04) | 3 (0.23, 109.12) |
| 0.19 (0, 9.23) | 0.9 (0.02, 28.09) | 0.61 (0.01, 19.02) | 0.34 (0, 23.02) | **NIP 30mg/kg** | 1.98 (0.06, 57.4) | 2.31 (0.2, 53.07) | 0.89 (0.02, 24.94) | 1.12 (0.03, 34.38) |
| 0.1 (0, 5.9) | 0.45 (0.01, 16.28) | 0.31 (0.01, 10.92) | 0.17 (0, 13.05) | 0.51 (0.02, 17.06) | **NIP 5mg/kg** | 1.14 (0.11, 32.68) | 0.45 (0.01, 14.41) | 0.56 (0.01, 19.79) |
| 0.08 (0, 2.05) | 0.38 (0.01, 5.17) | 0.26 (0.01, 3.45) | 0.14 (0, 5.07) | 0.43 (0.02, 4.98) | 0.88 (0.03, 9.3) | **NIP 60mg/kg** | 0.38 (0.01, 4.19) | 0.48 (0.01, 5.95) |
| 0.24 (0.01, 1.68) | 1.01 (0.39, 2.64) | 0.69 (0.26, 1.73) | 0.42 (0.01, 5.03) | 1.12 (0.04, 44.43) | 2.24 (0.07, 82.29) | 2.6 (0.24, 82.05) | **PLA** | 1.26 (0.59, 2.75) |
| 0.19 (0.01, 1.56) | 0.8 (0.23, 2.73) | 0.55 (0.16, 1.81) | 0.33 (0.01, 4.42) | 0.9 (0.03, 37.16) | 1.79 (0.05, 69.33) | 2.08 (0.17, 70.58) | 0.8 (0.36, 1.69) | **RAV** |
| 0.13 (0, 2.07) | 0.59 (0.06, 4.52) | 0.4 (0.04, 3.09) | 0.23 (0, 5.26) | 0.65 (0.01, 38.78) | 1.27 (0.02, 71.17) | 1.55 (0.06, 74.45) | 0.58 (0.08, 3.59) | 0.73 (0.08, 5.26) |
| 0.2 (0.01, 1.7) | 0.84 (0.23, 2.97) | 0.57 (0.16, 1.99) | 0.35 (0.01, 4.85) | 0.93 (0.03, 39.94) | 1.86 (0.05, 74.79) | 2.2 (0.17, 74.31) | 0.83 (0.36, 1.9) | 1.05 (0.34, 3.27) |
| 0.13 (0, 1.04) | 0.53 (0.15, 1.77) | 0.36 (0.1, 1.19) | 0.22 (0.01, 2.95) | 0.59 (0.02, 24.79) | 1.18 (0.03, 46.07) | 1.38 (0.11, 45.71) | 0.53 (0.24, 1.09) | 0.66 (0.22, 1.91) |
| **0.04 (0, 0.53)** | 0.19 (0.02, 1.06) | **0.13 (0.02, 0.7)** | 0.08 (0, 1.43) | 0.21 (0, 10.84) | 0.41 (0.01, 19.74) | 0.49 (0.02, 19.84) | **0.19 (0.03, 0.77)** | 0.24 (0.03, 1.21) |

| 7.63 (0.48, 295) | 5.02 (0.59, 129.89) | 7.94 (0.96, 204.37) | 23.37 (1.89, 826.4) |
| --- | --- | --- | --- |
| 1.71 (0.22, 16.16) | 1.19 (0.34, 4.26) | 1.89 (0.56, 6.48) | 5.23 (0.94, 45.36) |
| 2.53 (0.32, 24.27) | 1.76 (0.5, 6.4) | 2.77 (0.84, 9.73) | 7.68 (1.42, 66.58) |
| 4.27 (0.19, 235.35) | 2.87 (0.21, 103.93) | 4.54 (0.34, 163.8) | 13.33 (0.7, 651.46) |
| 1.54 (0.03, 72.58) | 1.07 (0.03, 33.51) | 1.69 (0.04, 51.9) | 4.85 (0.09, 215.17) |
| 0.79 (0.01, 43) | 0.54 (0.01, 19.19) | 0.85 (0.02, 29.9) | 2.44 (0.05, 118.23) |
| 0.64 (0.01, 15.49) | 0.46 (0.01, 5.9) | 0.73 (0.02, 9.19) | 2.04 (0.05, 42.4) |
| 1.72 (0.28, 13.3) | 1.2 (0.53, 2.8) | 1.89 (0.92, 4.17) | 5.17 (1.29, 38.15) |
| 1.37 (0.19, 12.24) | 0.96 (0.31, 2.97) | 1.51 (0.52, 4.49) | 4.15 (0.83, 34.37) |
| **RIT** | 0.69 (0.08, 5.21) | 1.1 (0.13, 8.03) | 3.08 (0.25, 44.26) |
| 1.44 (0.19, 12.91) | **ROZ 10mg/kg** | 1.58 (0.79, 3.3) | 4.35 (0.85, 37.93) |
| 0.91 (0.12, 7.81) | 0.63 (0.3, 1.26) | **ROZ 7mg/kg** | 2.74 (0.55, 23.07) |
| 0.32 (0.02, 4.03) | 0.23 (0.03, 1.17) | 0.37 (0.04, 1.82) | **ZIL** |

**Figure S1: Risk of bias.**


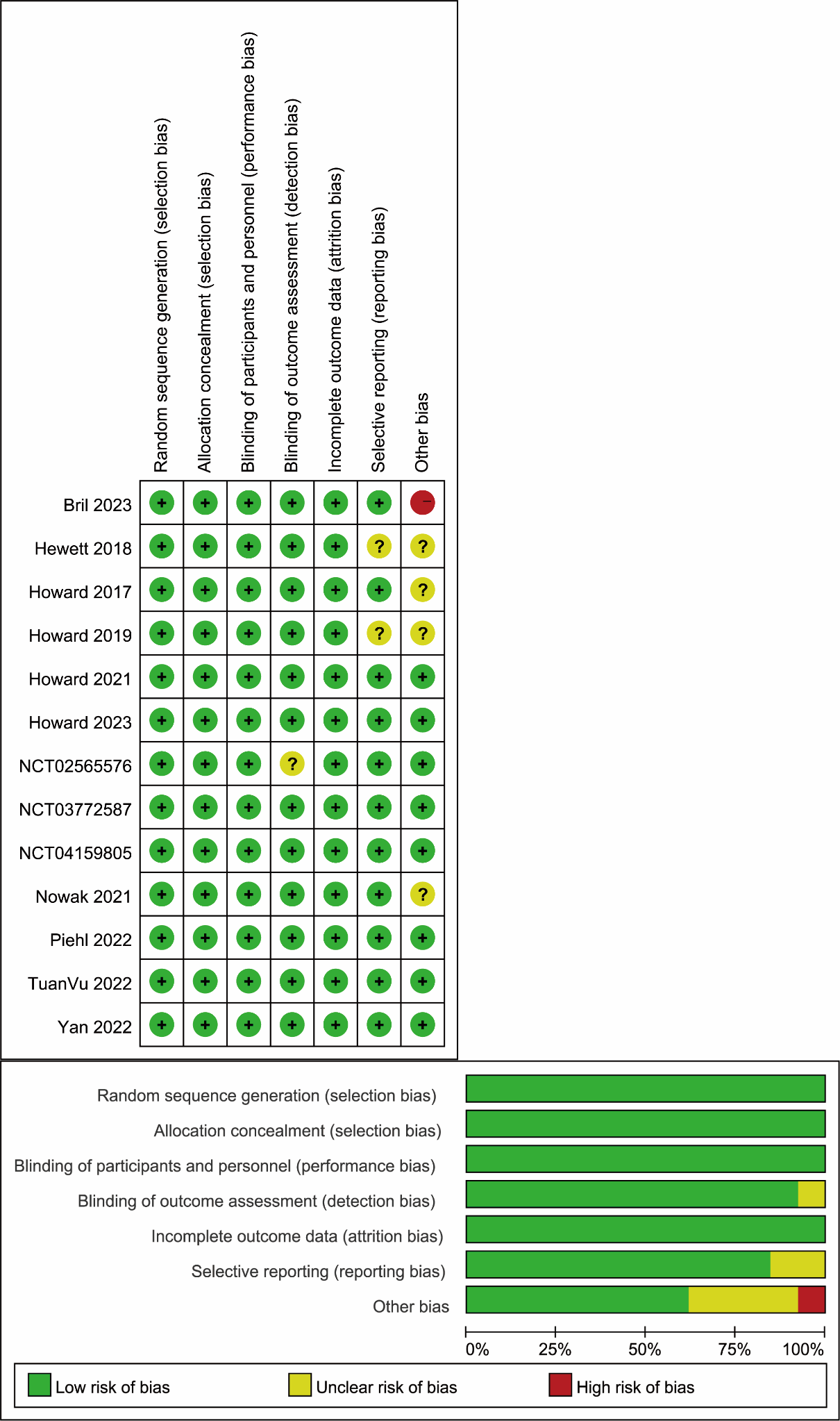


**Figure S2: Forest plots of the network meta-analysis: MG-ADL.**

**
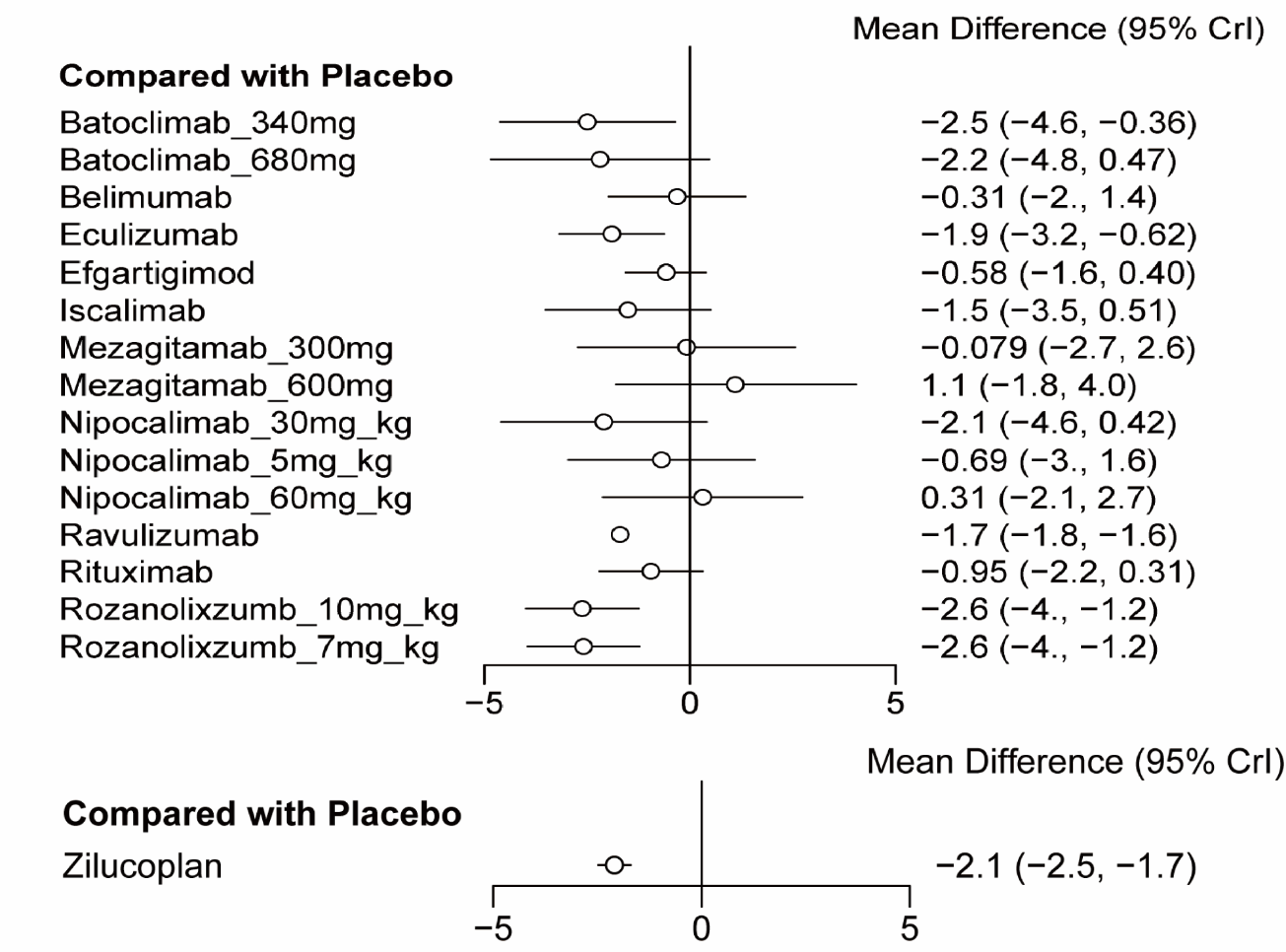
**

**Figure S3: Forest plots of the network meta-analysis: QMG.**

**
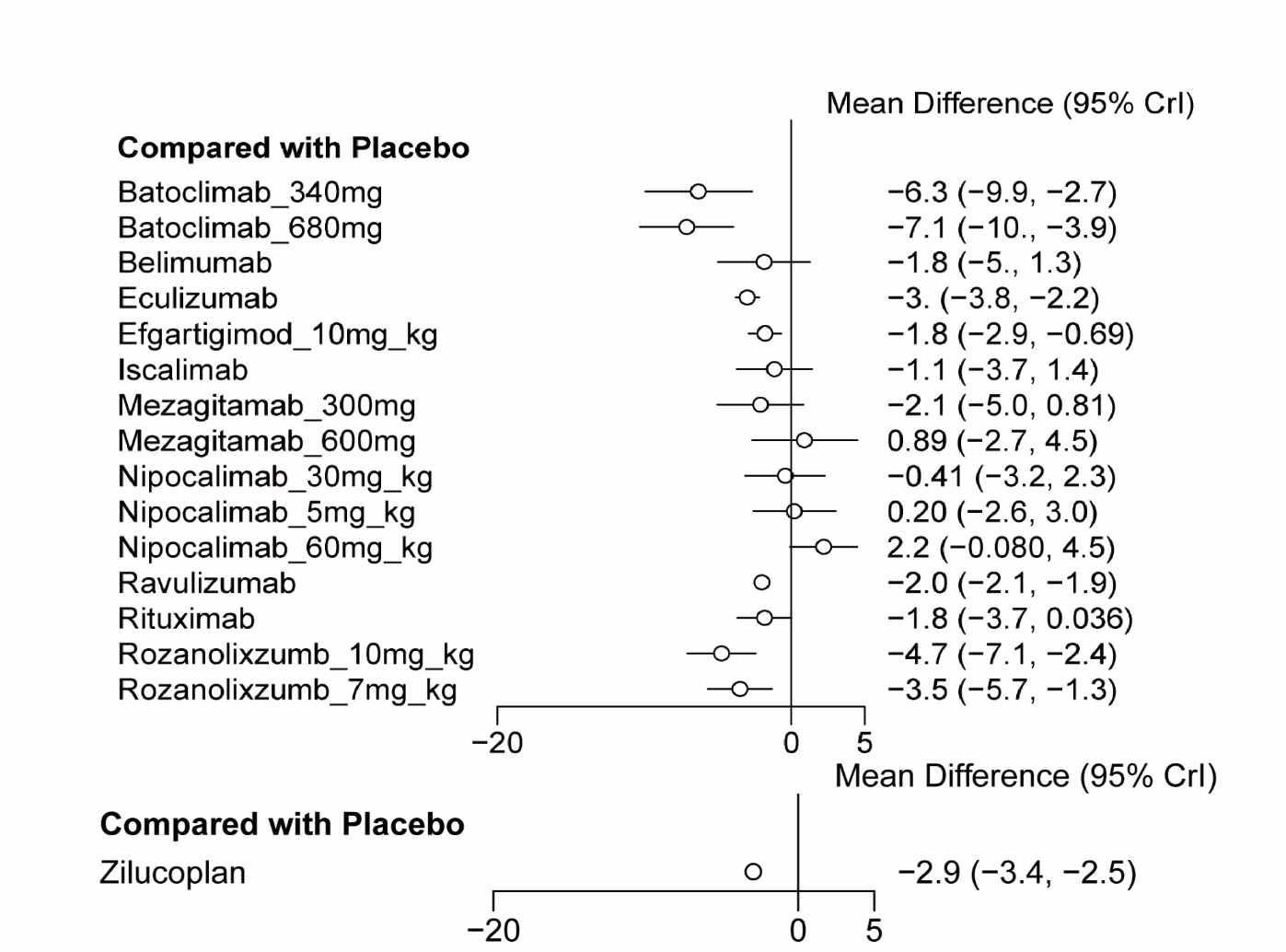
**

**Figure S4: Forest plots of the network meta-analysis: MGC.**

**
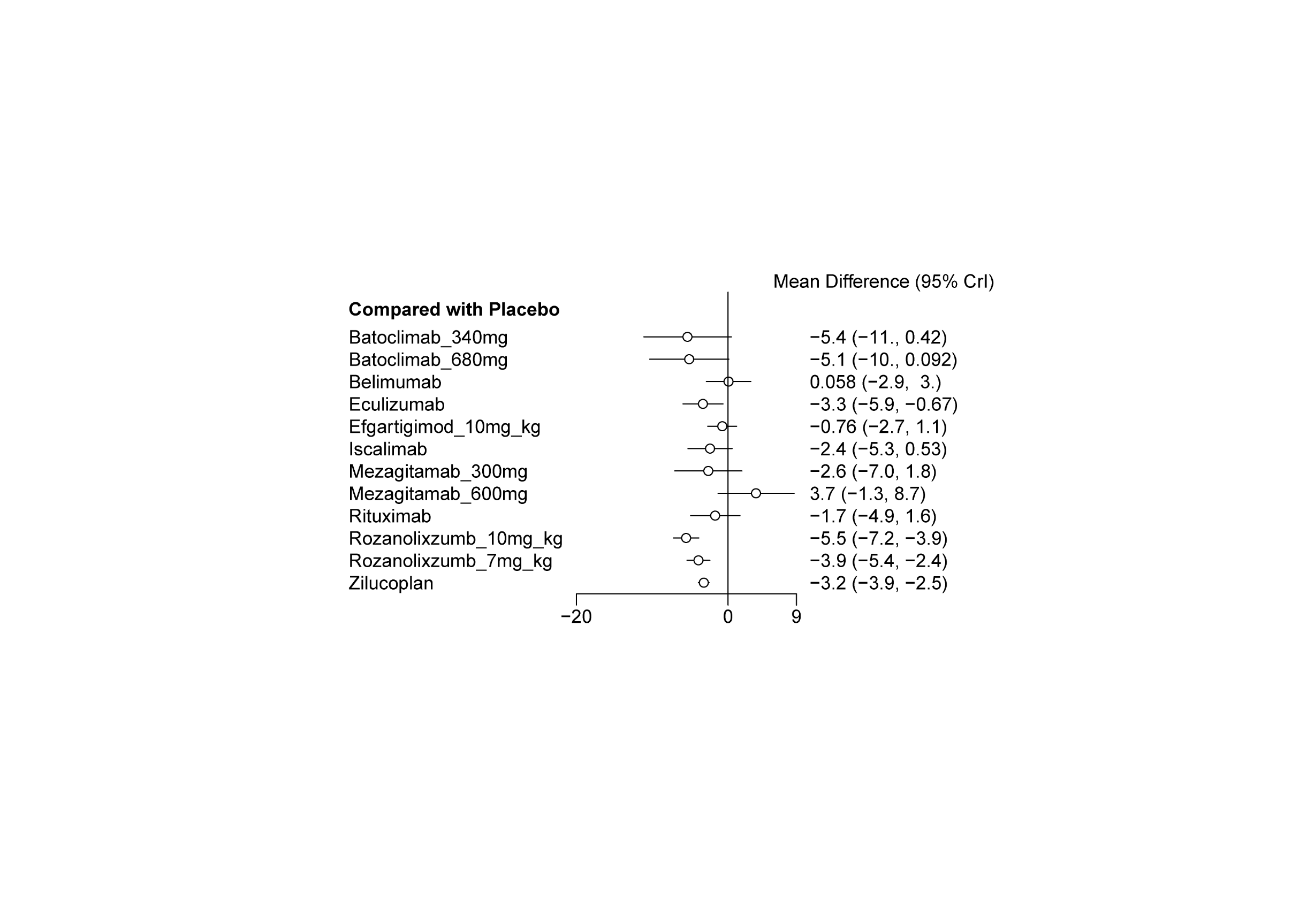
**

**Figure S5: Forest plots of the network meta-analysis: MG-QoL 15r**

**
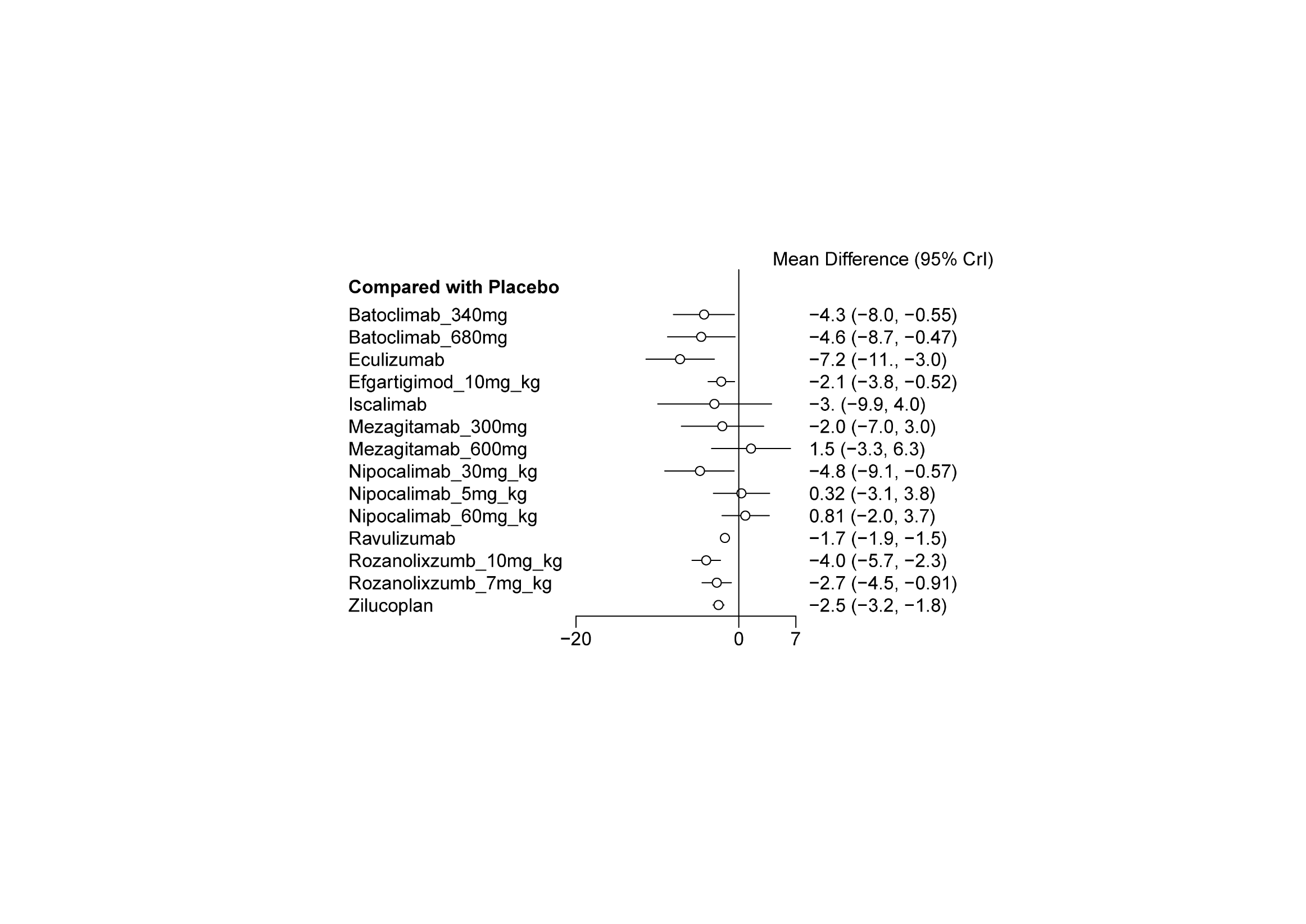
**

**Figure S6: Forest plots of the network meta-analysis: AEs.**

**
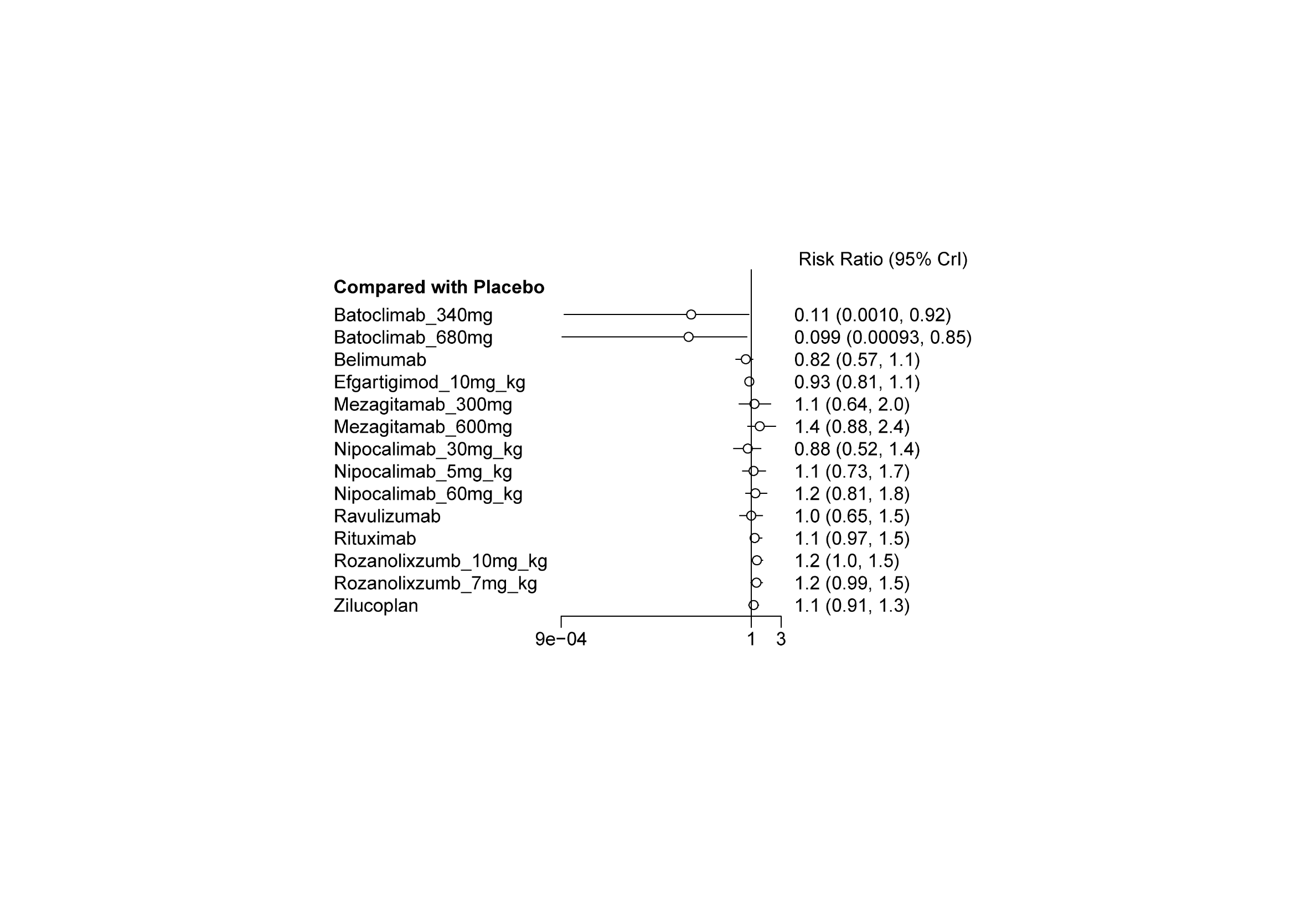
**

**Figure S7: Forest plots of the network meta-analysis: Headache.**

**
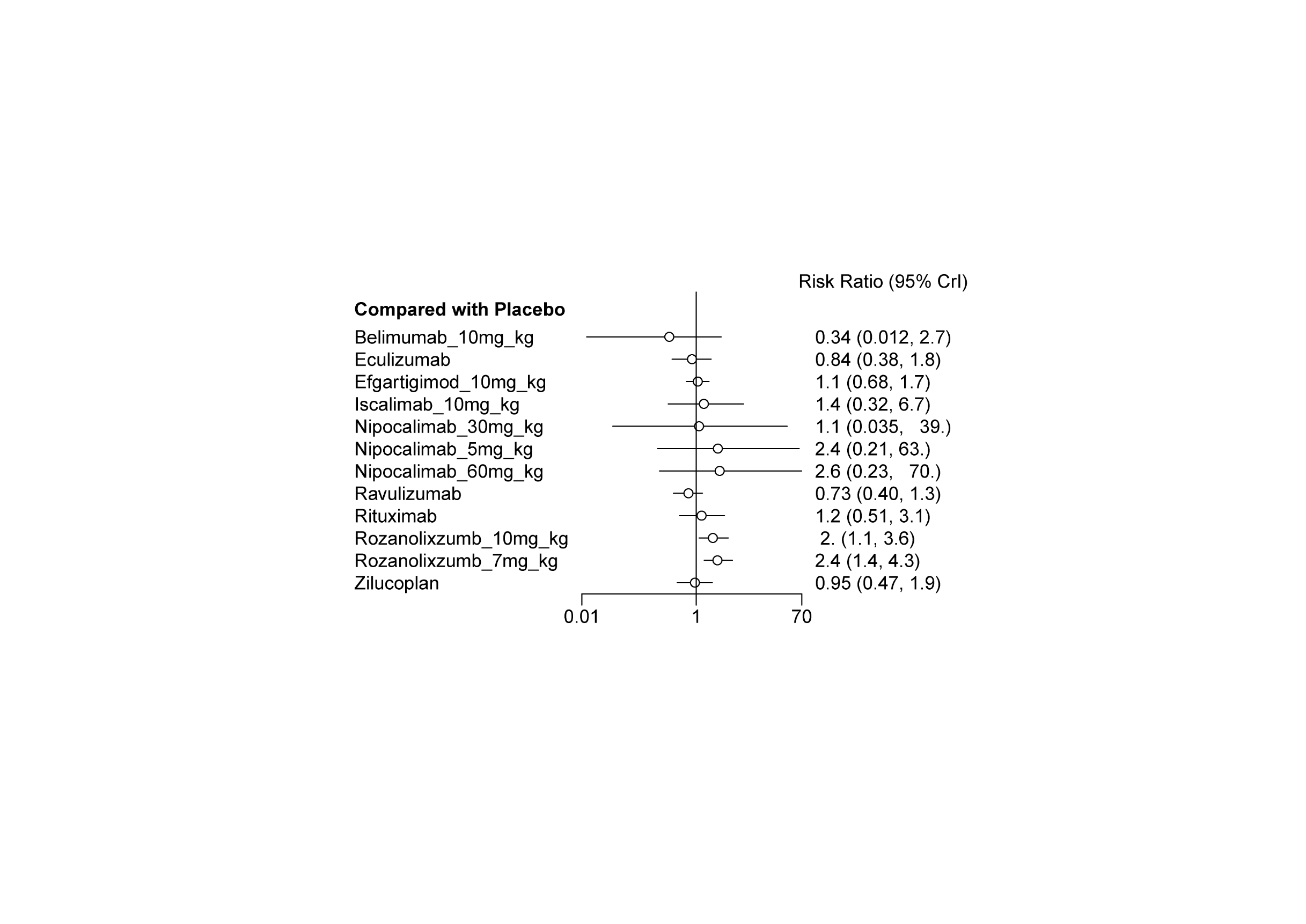
**

**Figure S8: Forest plots of the network meta-analysis: Diarrhea.**

**
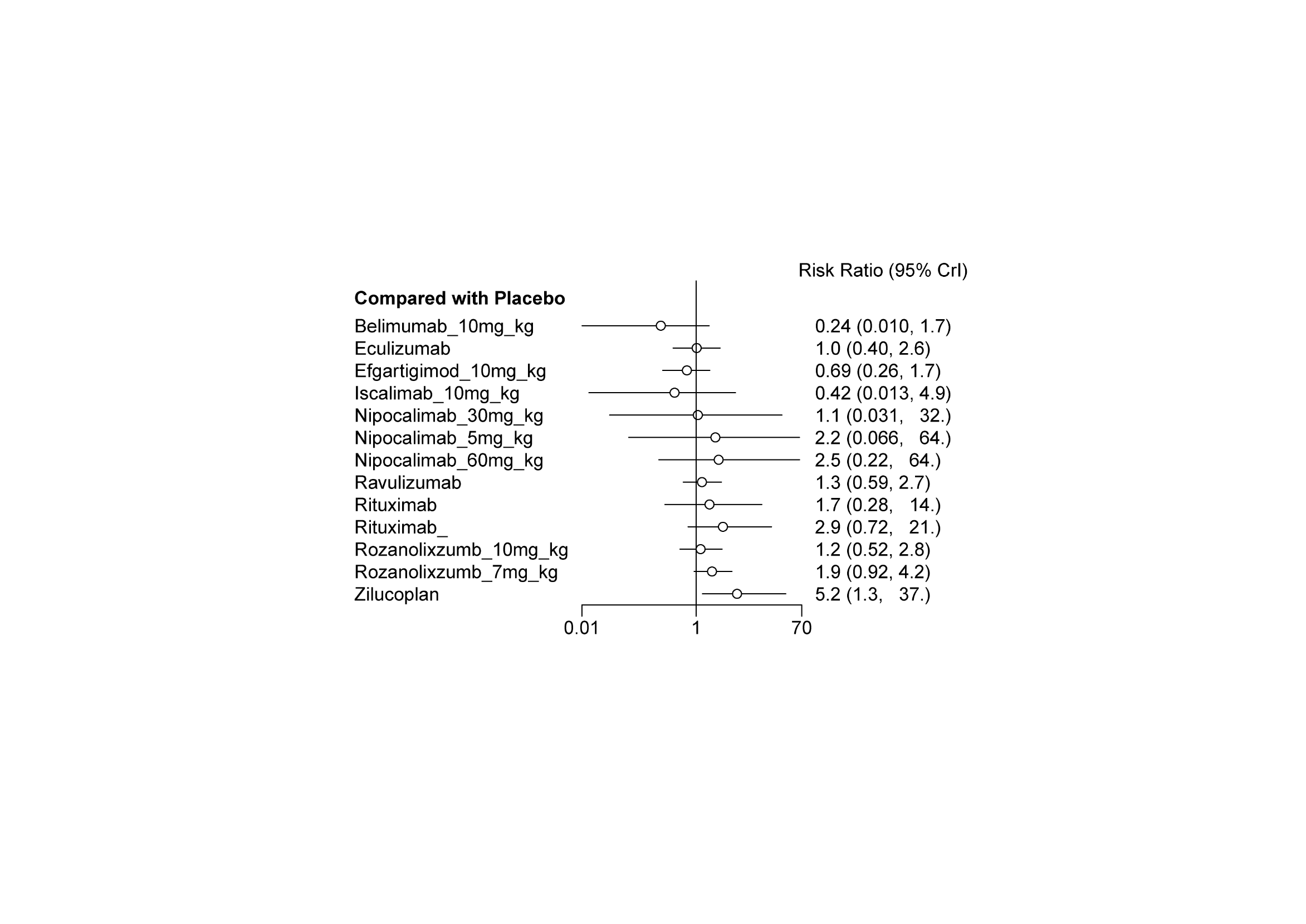
**

**Figure S9: Forest plots of the network meta-analysis: Nausea.**

**
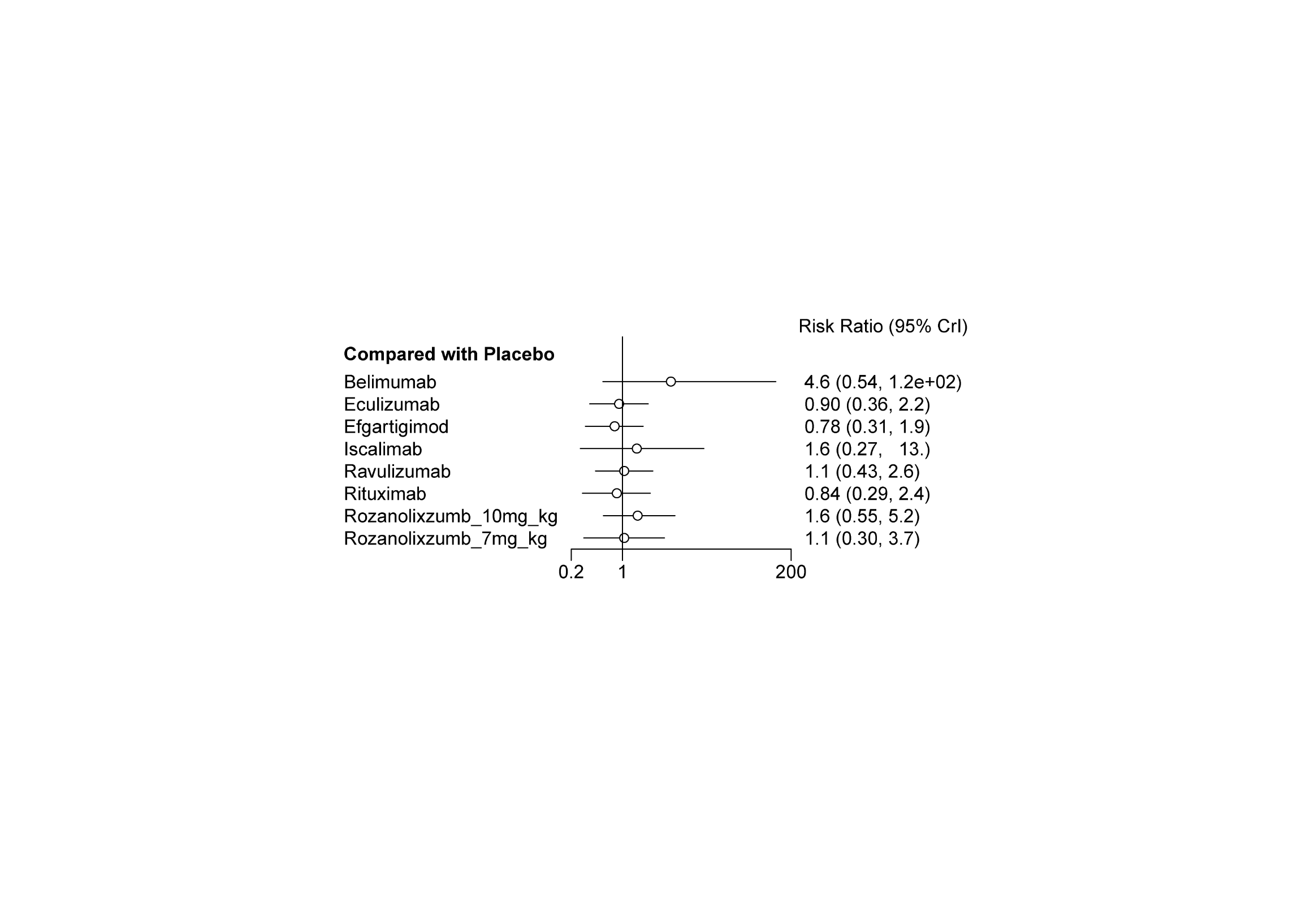
**

**Figure S10: Pairwise meta-analysis of efficacy: MG-ADL.**

**
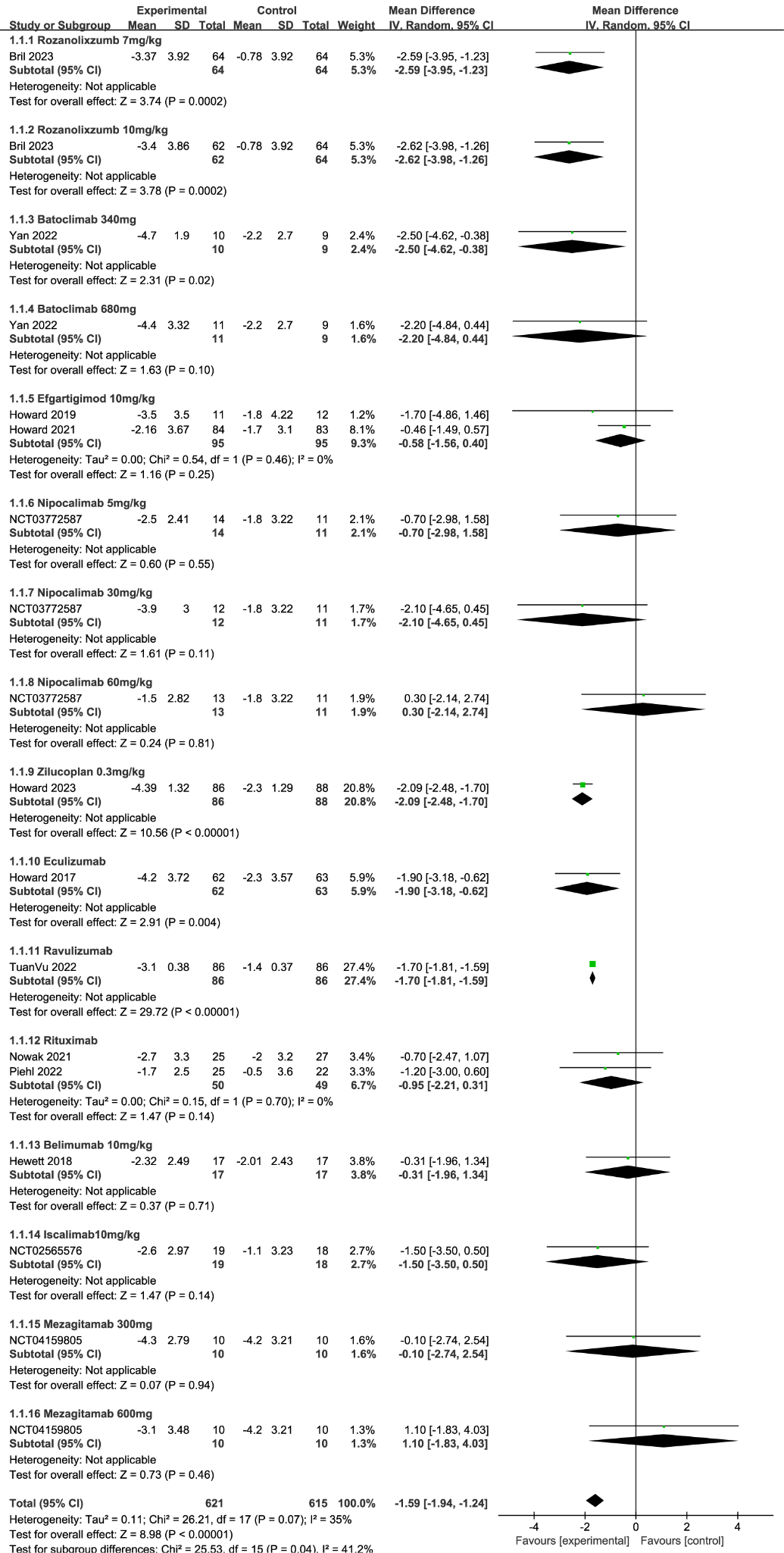
**

**Figure S11: Pairwise meta-analysis of efficacy: QMG.**

**
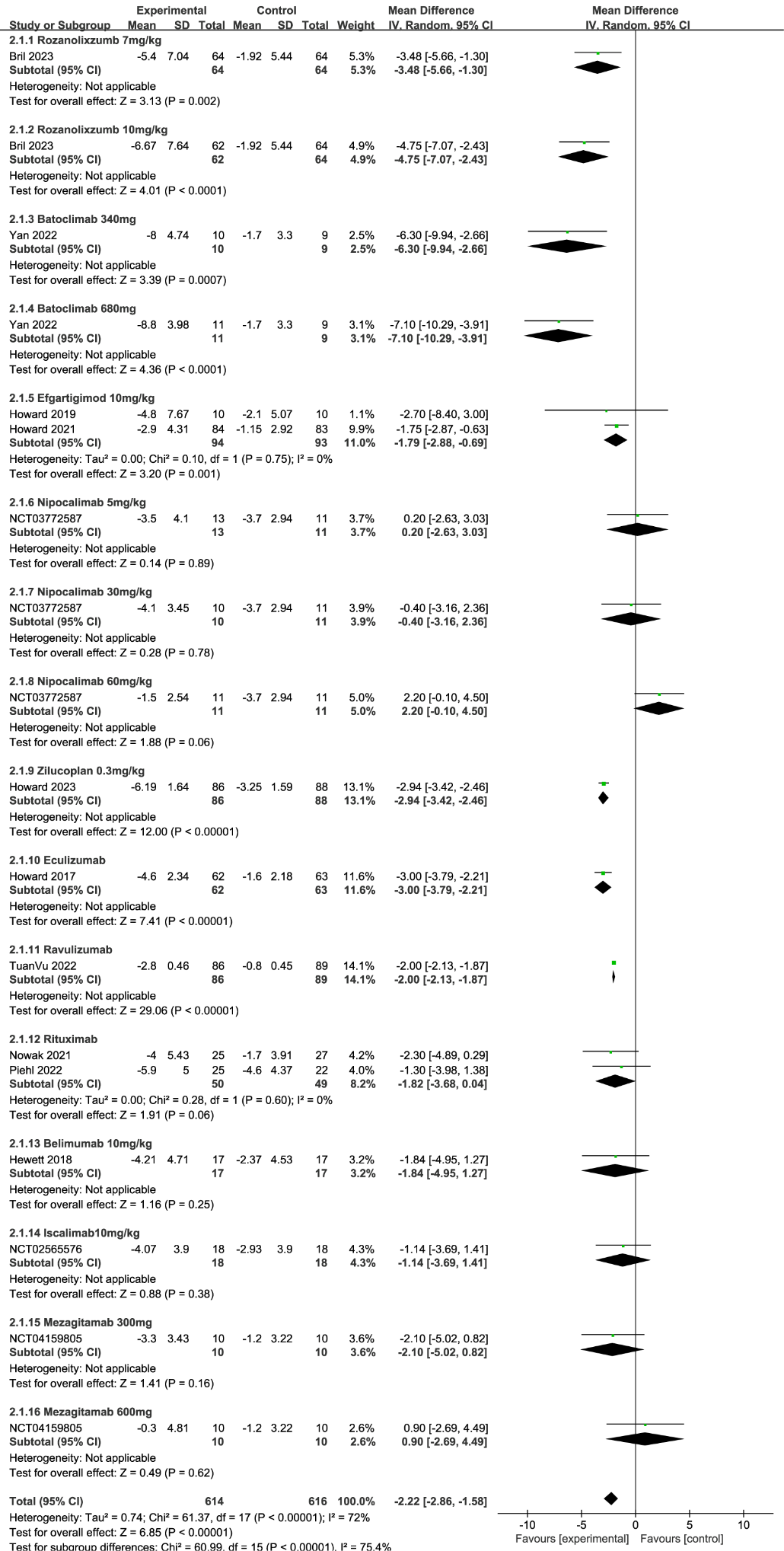
**

**Figure S12: Pairwise meta-analysis of efficacy: MGC.**

**
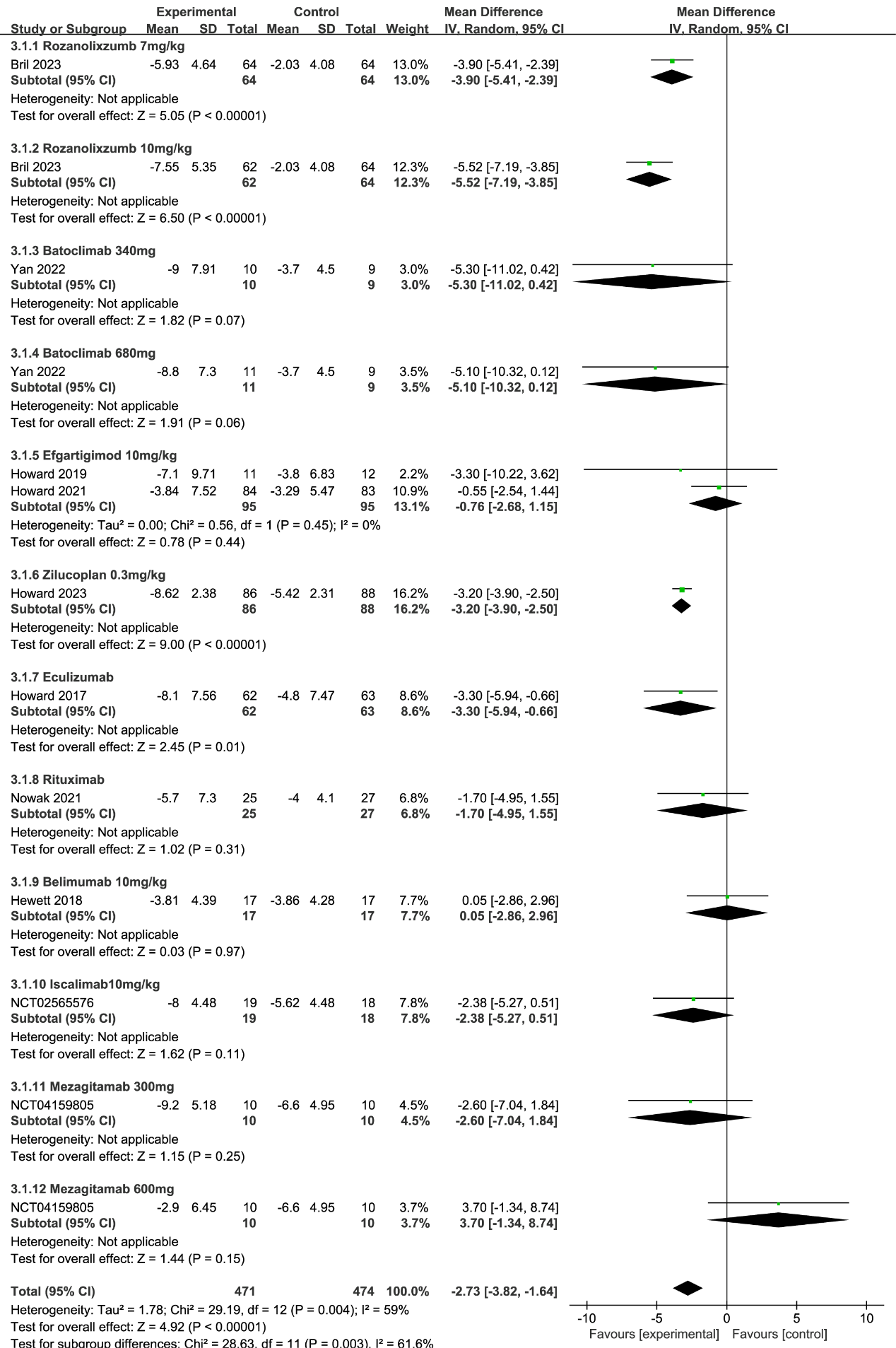
**

**Figure S13: Pairwise meta-analysis of efficacy: MG-QoL 15r.**

**
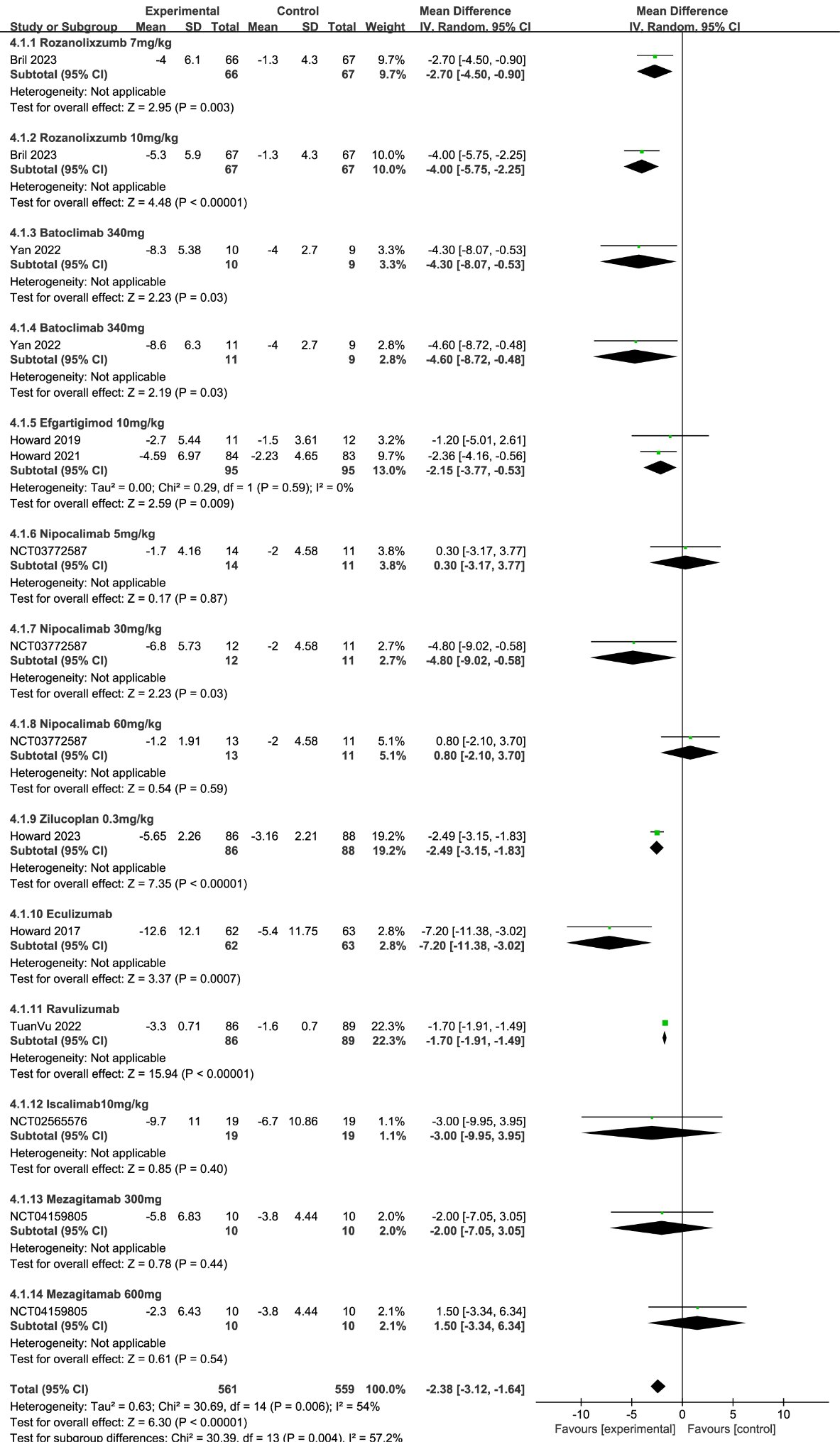
**

**Figure S14: Pairwise meta-analysis of safety: AEs.**

**
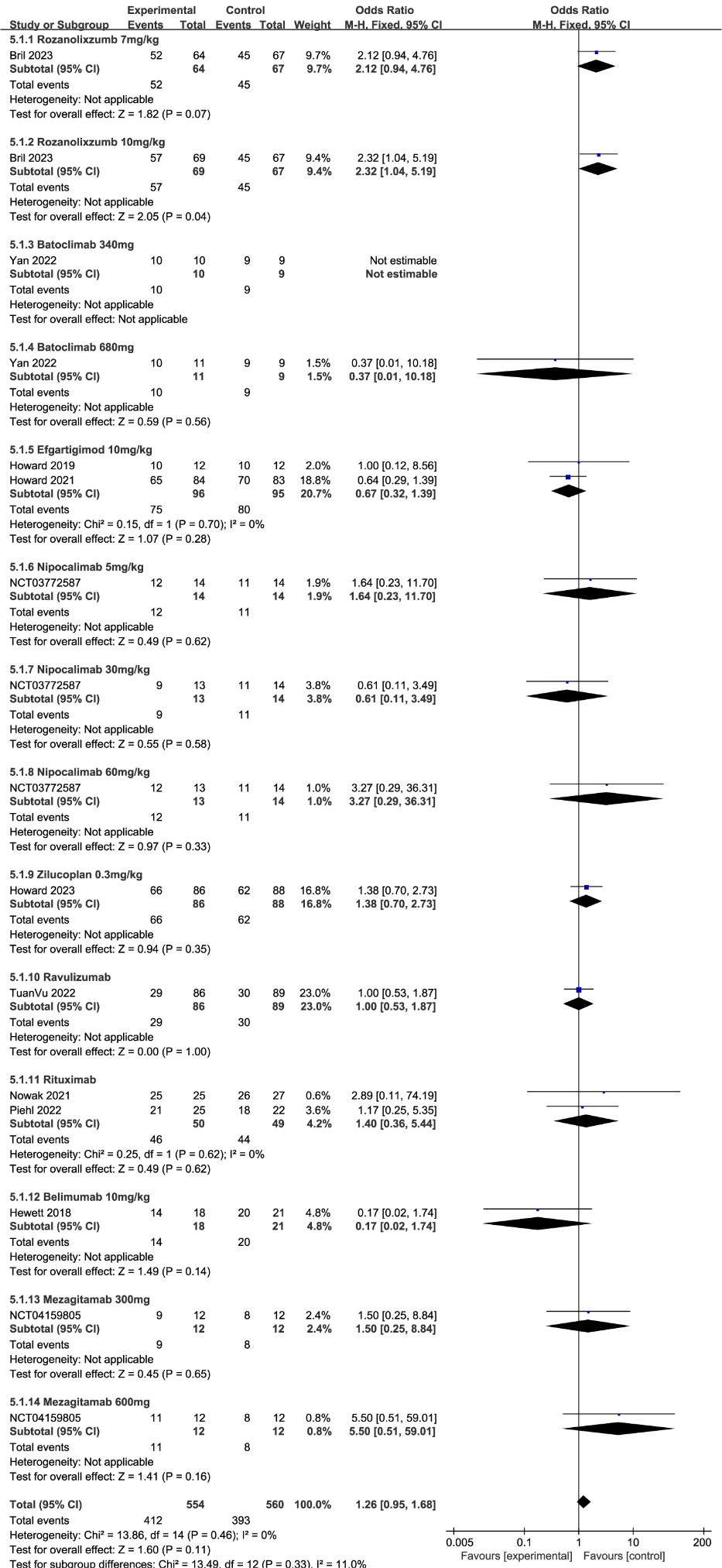
**

**Figure S15: Pairwise meta-analysis of safety: SAEs.**

**
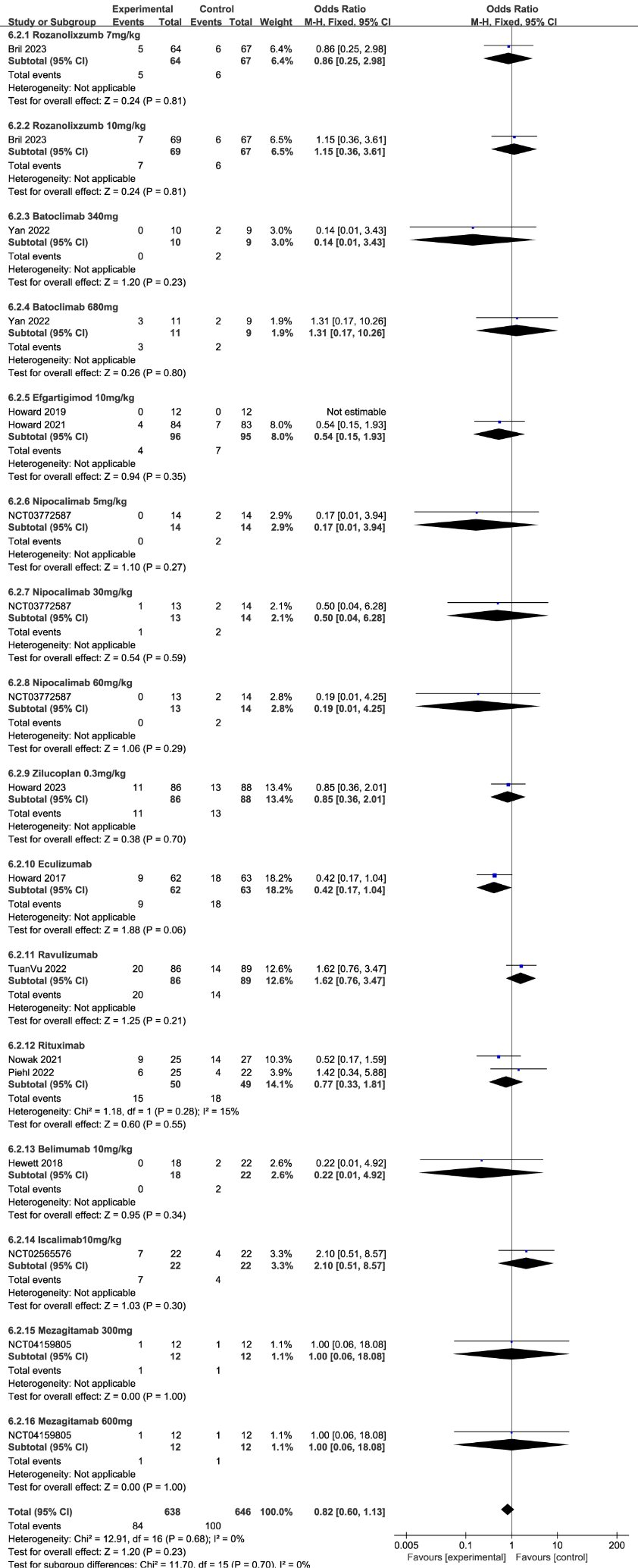
**

**Figure S16: Pairwise meta-analysis of safety: Headache.**

**
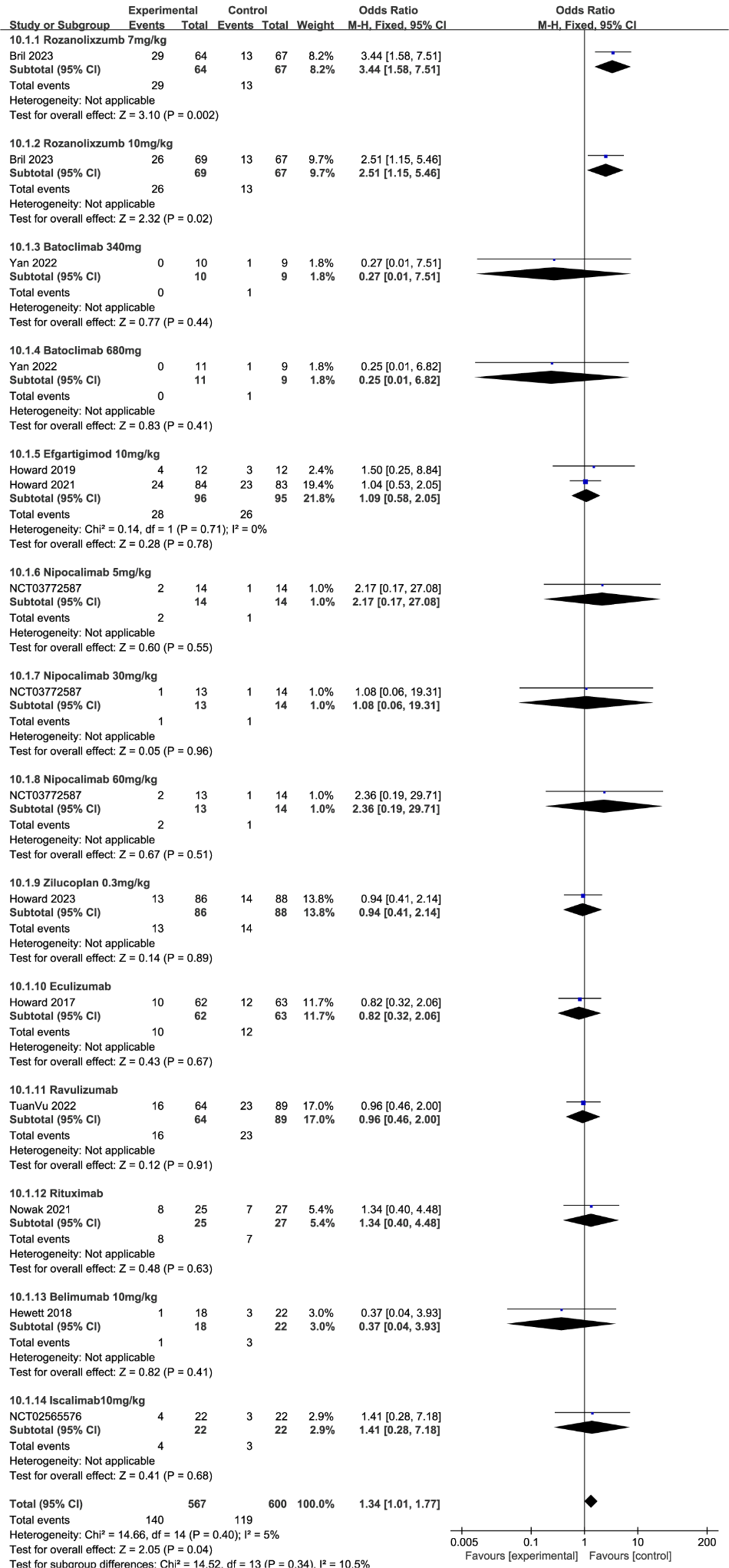
**

**Figure S17: Pairwise meta-analysis of safety: Diarrhea.**

**
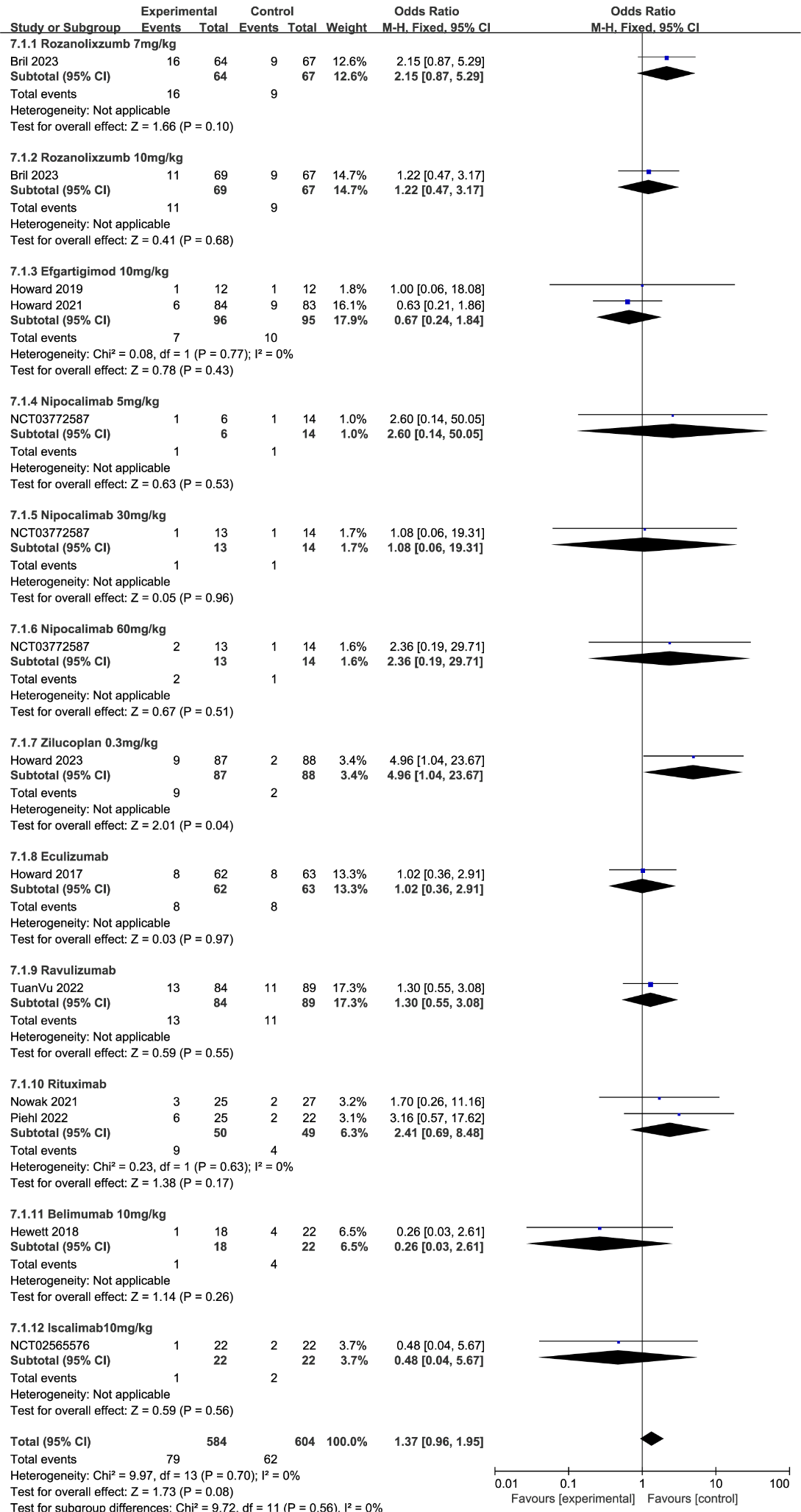
**

**Figure S18: Pairwise meta-analysis of safety: Nausea.**

**
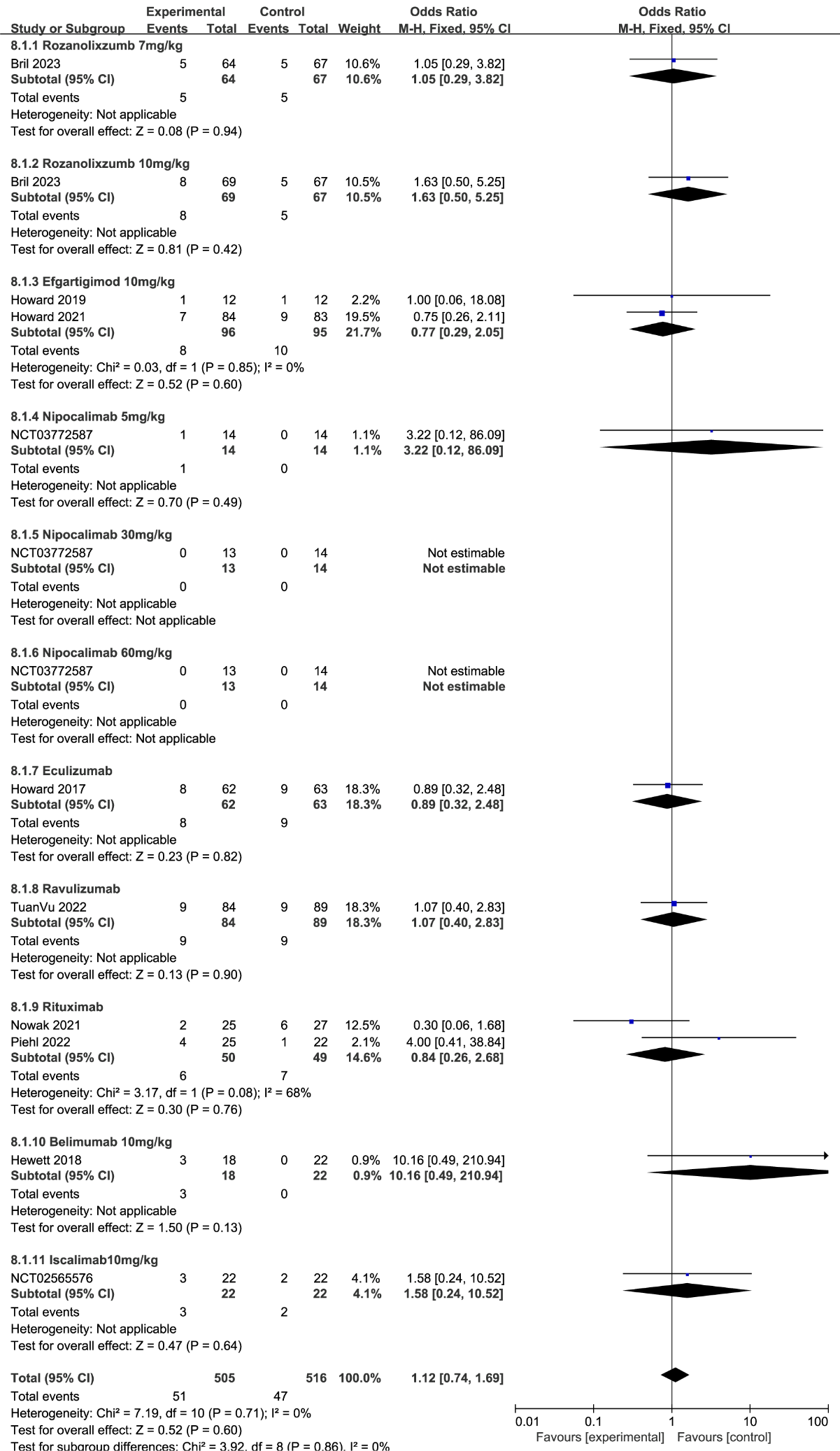
**

**Figure S19: League tables of the headache, diarrhea, and Nausea.**

**
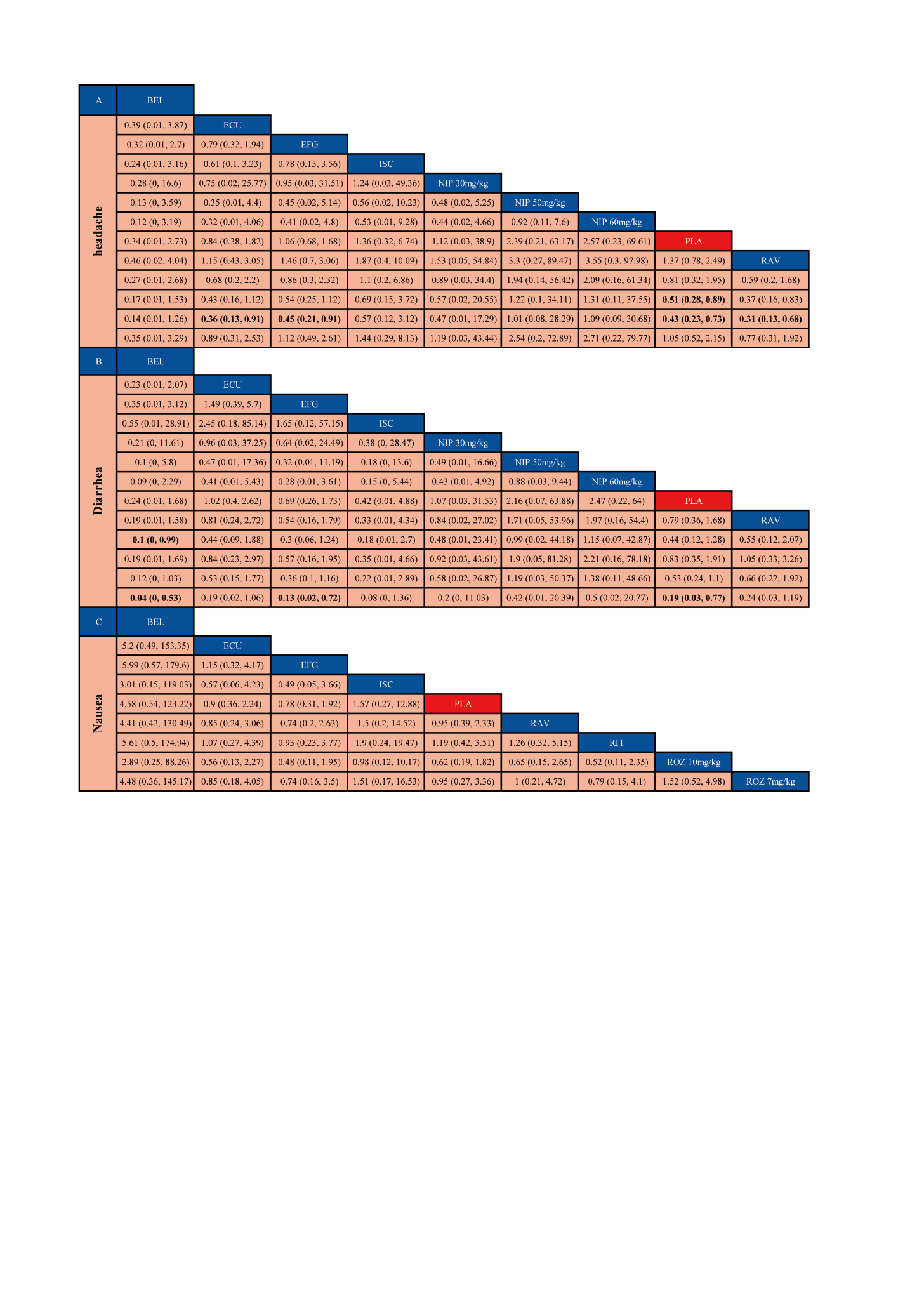
**

**Figure S20: Cumulative probability of different monoclonal antibodies for efficacy outcome.**

**
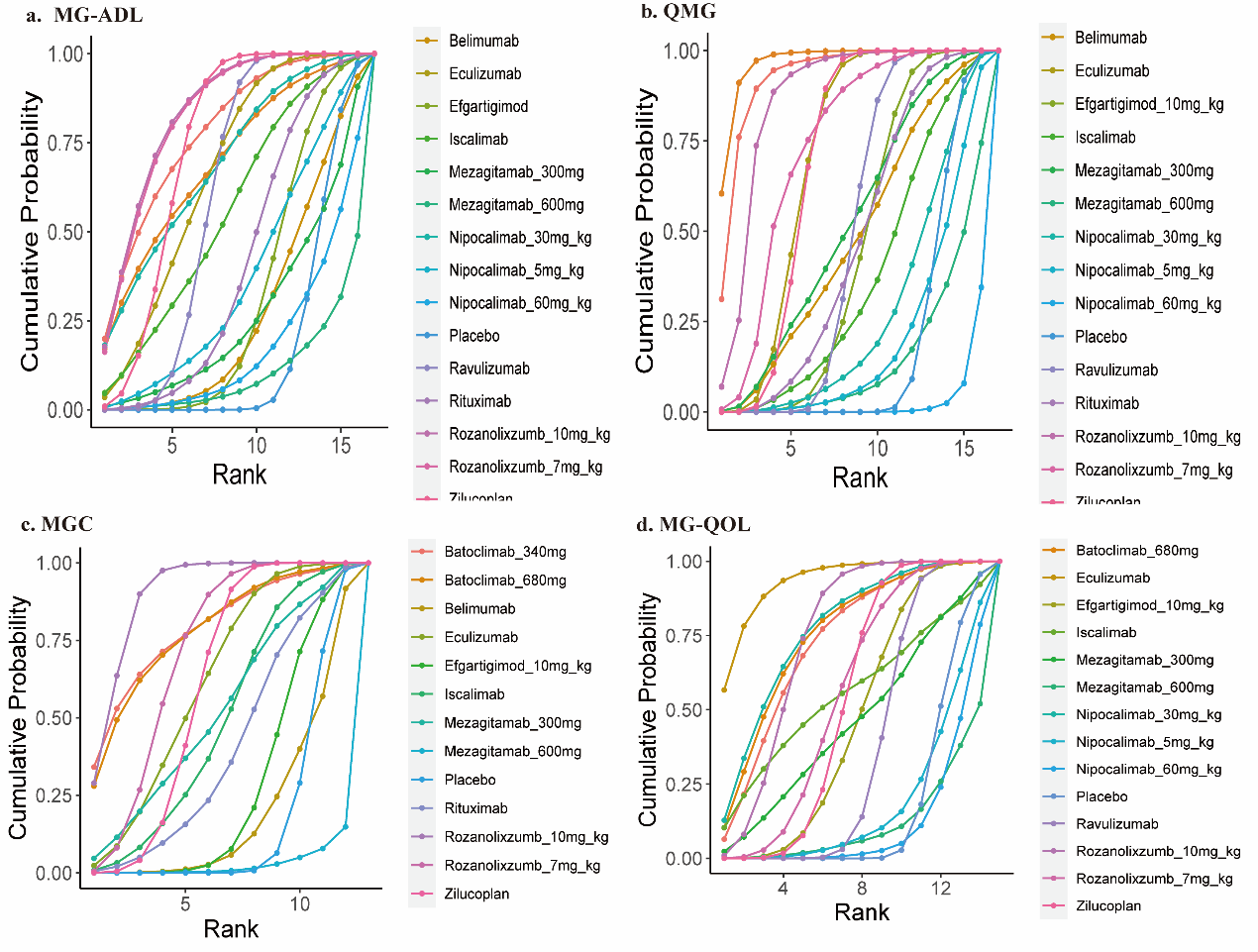
**

**Figure S21: Cumulative probability of different monoclonal antibodies for safety outcome.**

**
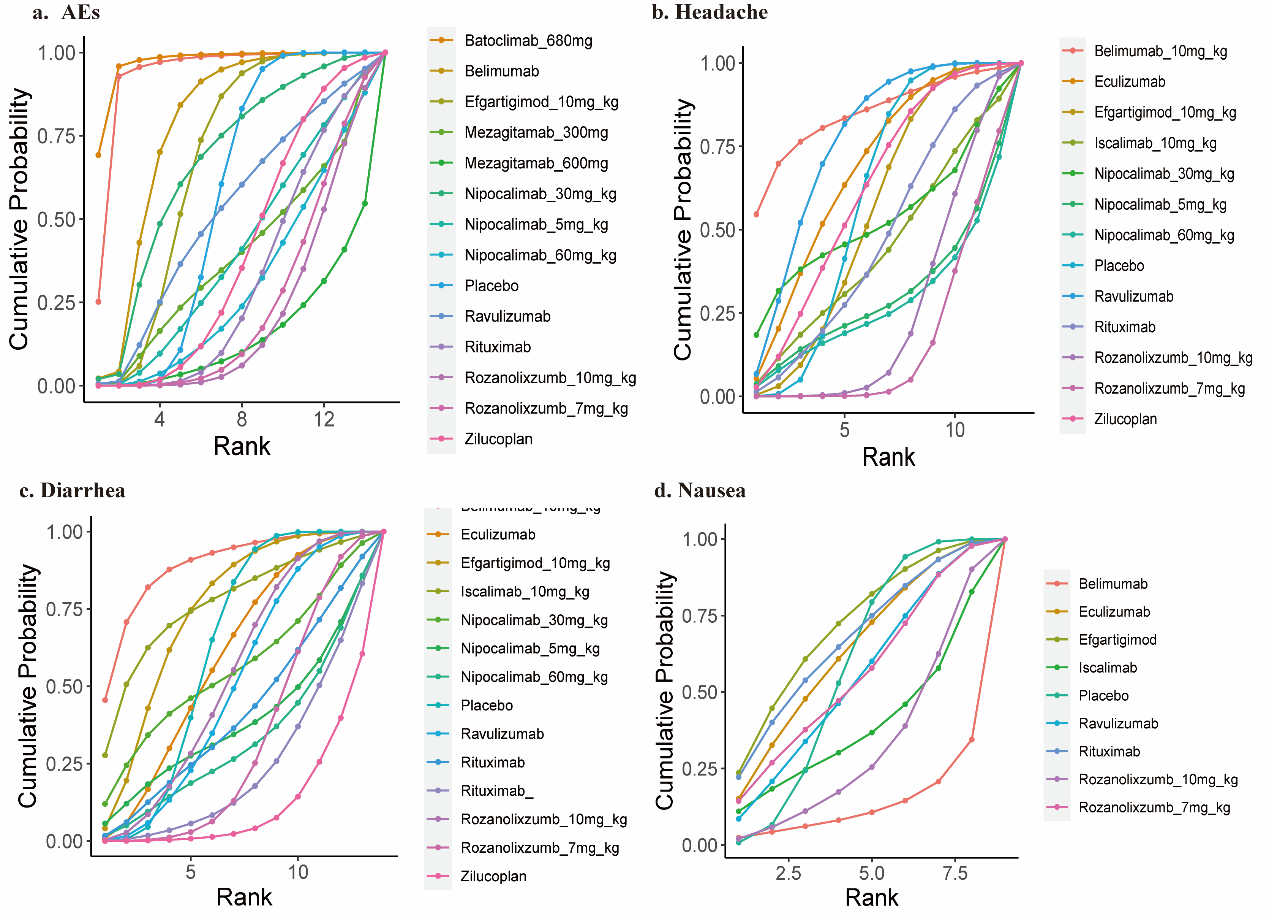
**

**Figure S22: Convergence diagnostics of the network meta-analysis: MG-ADL.**

**
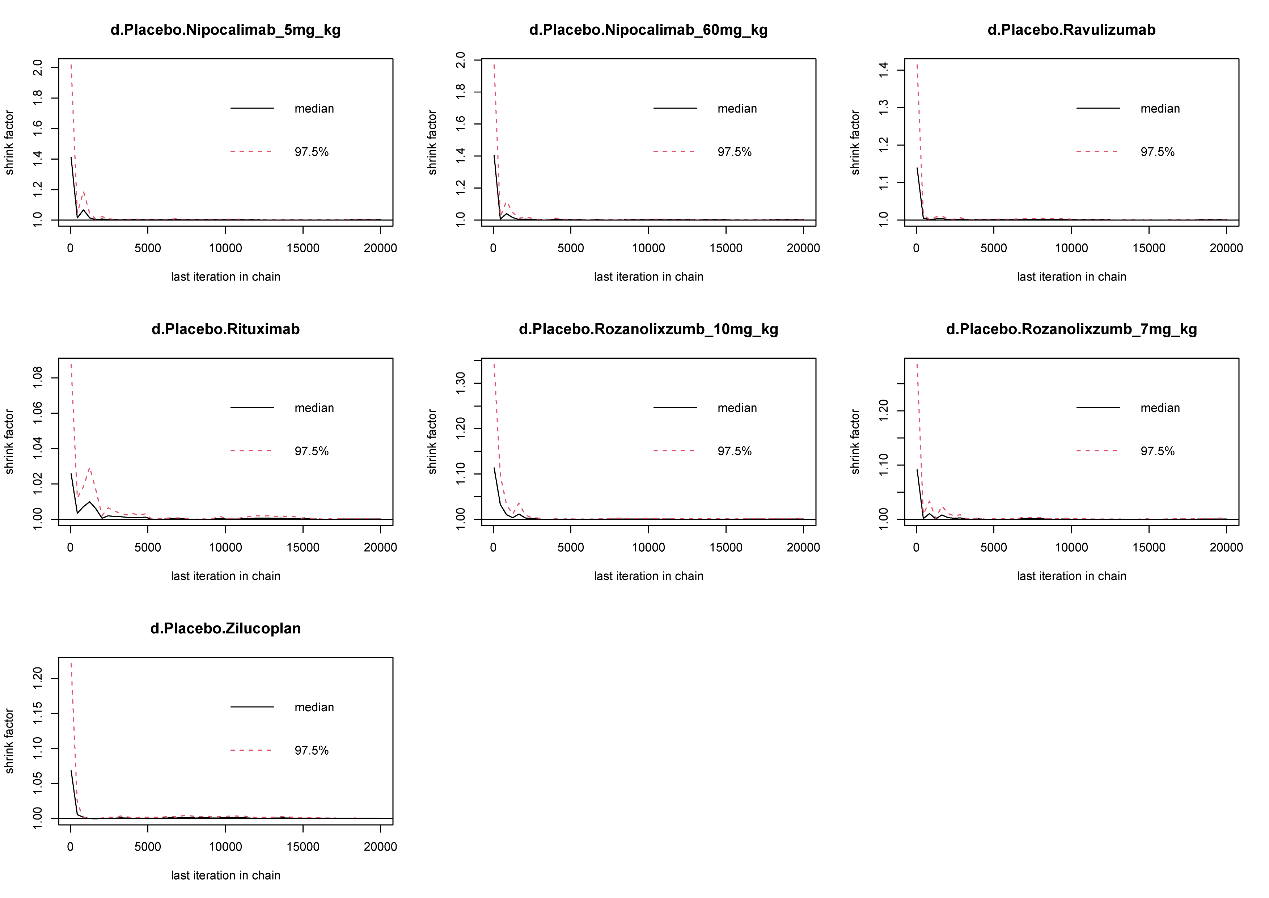
**

**Figure S23: Convergence diagnostics of the network meta-analysis: QMG.**

**
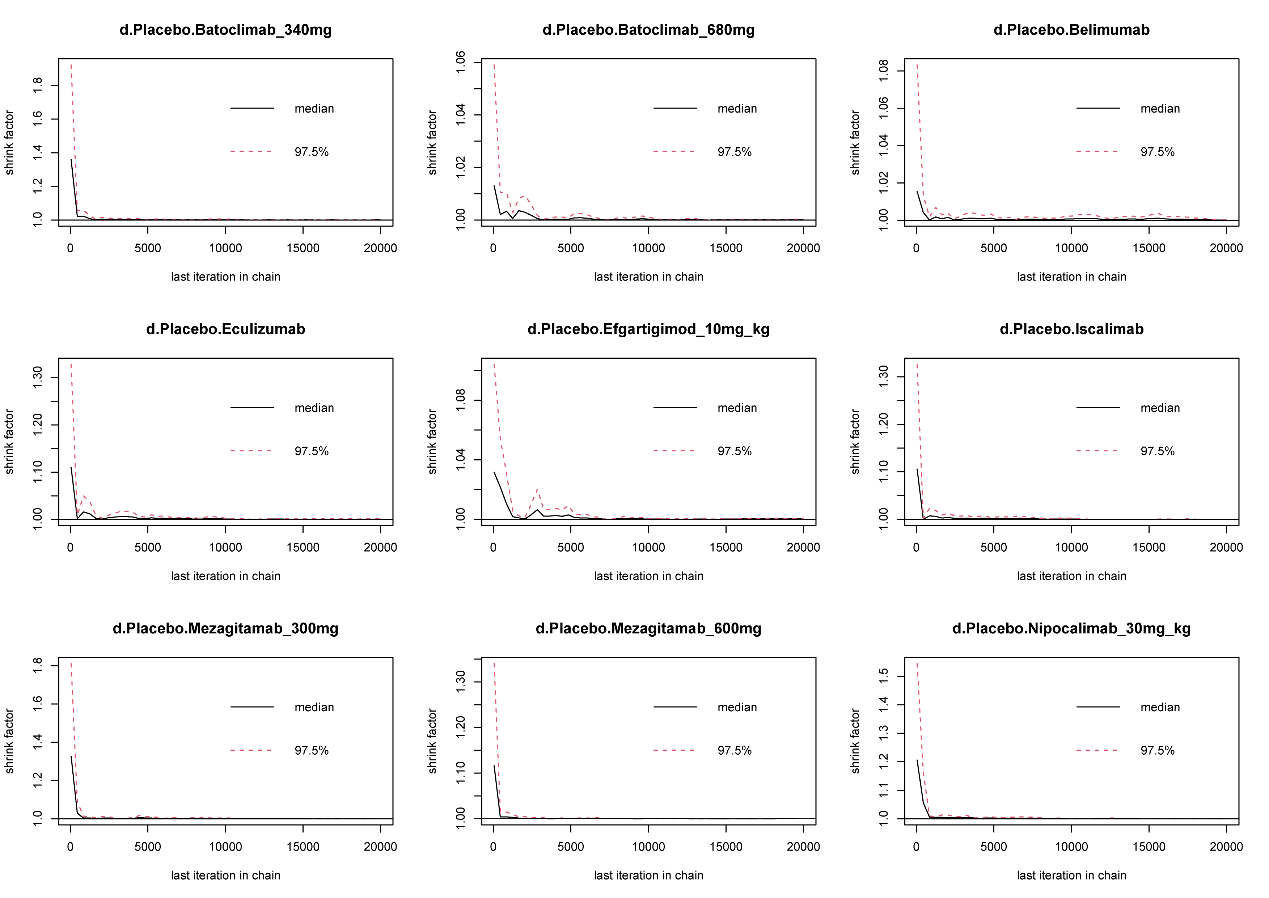
**

**
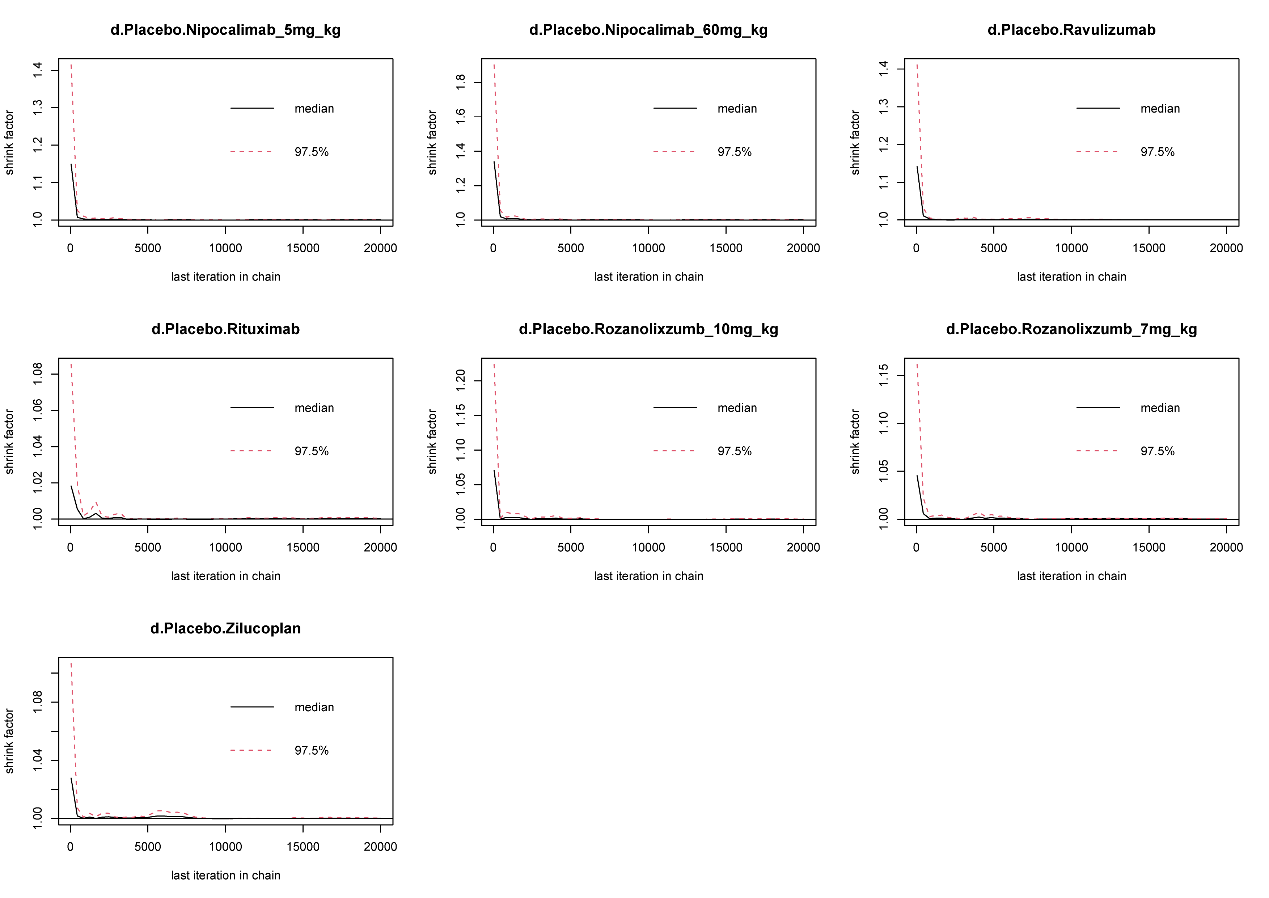
**

**Figure S24: Convergence diagnostics of the network meta-analysis: MGC.**

**
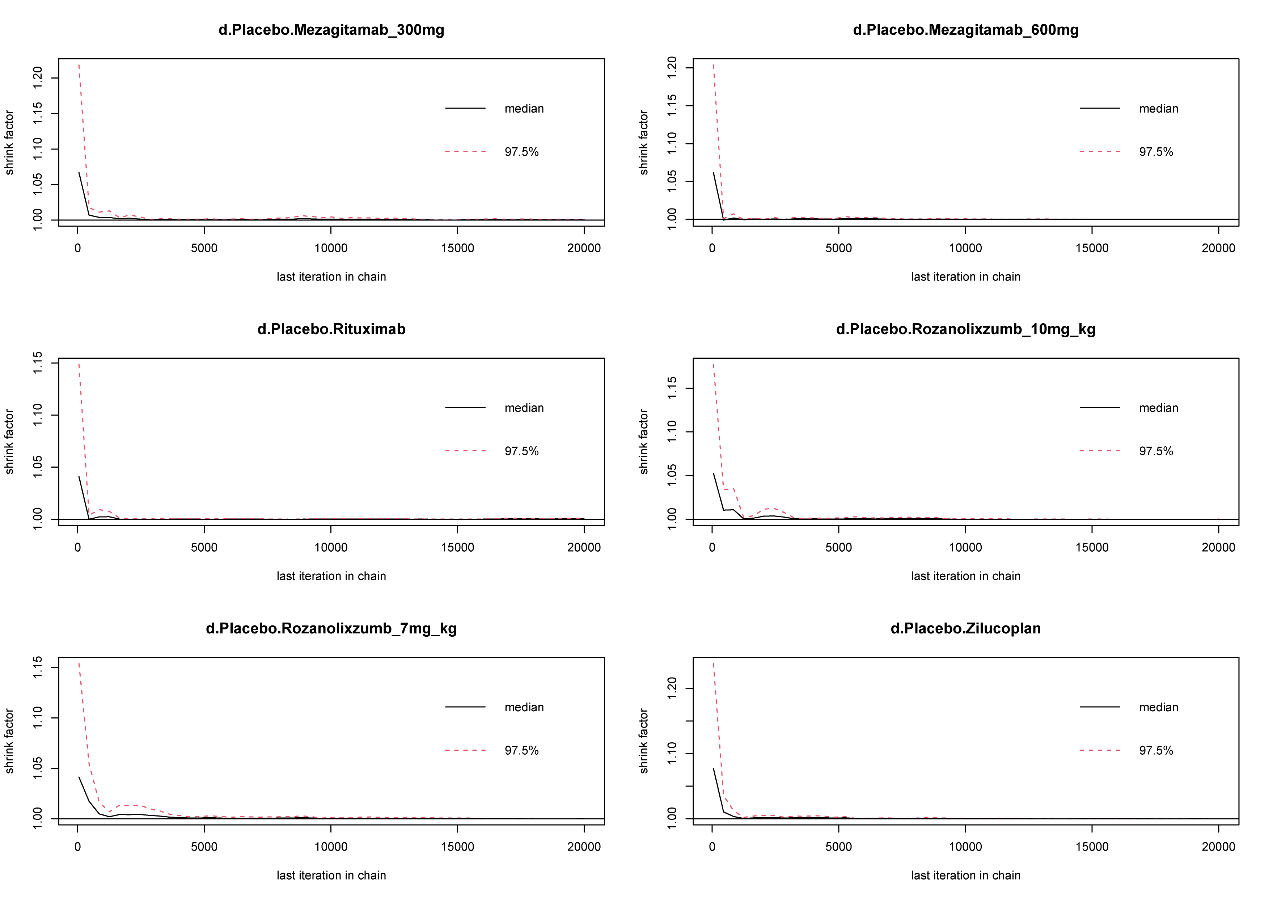
**

**Figure S25: Convergence diagnostics of the network meta-analysis: MG-QoL 15r.**

**
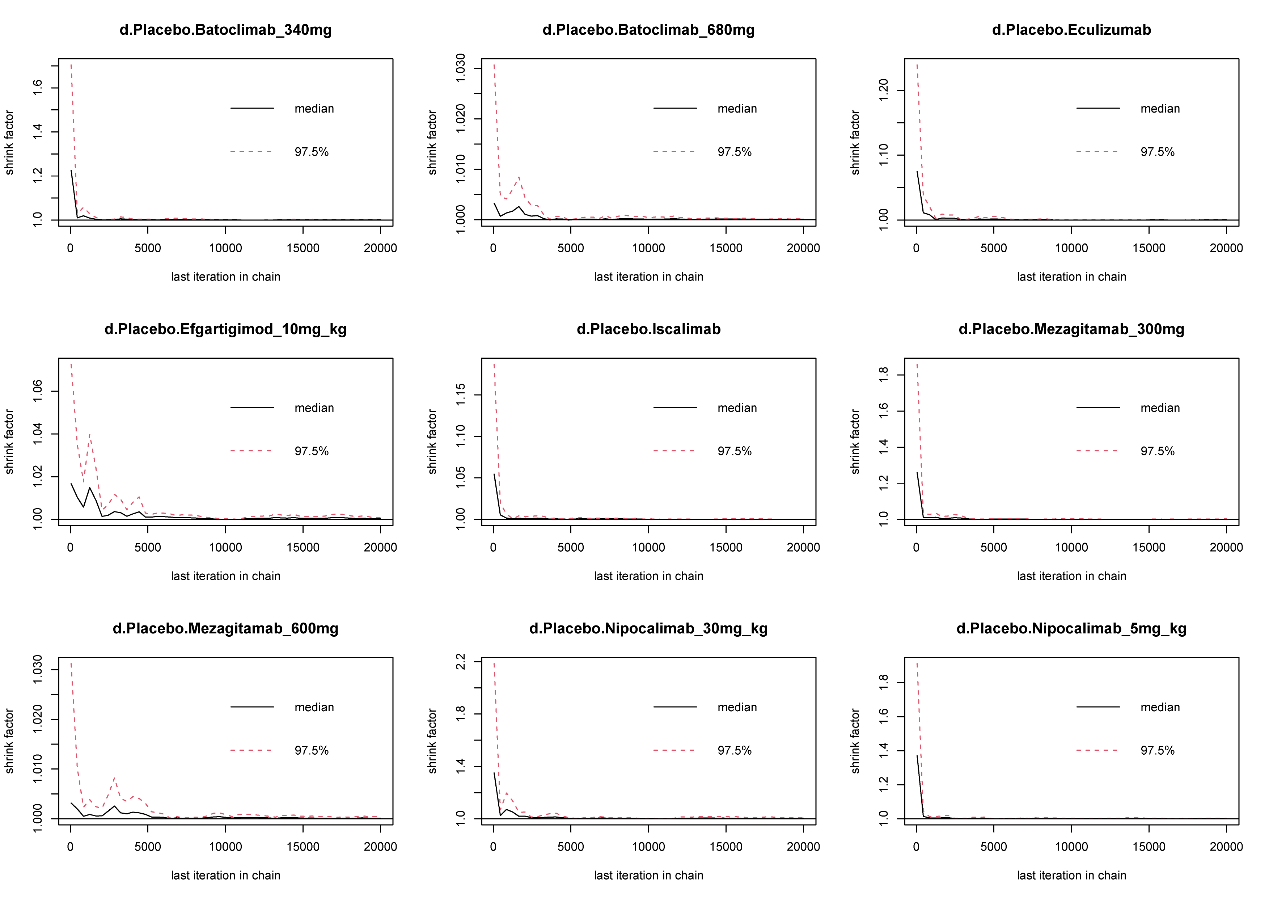
**

**
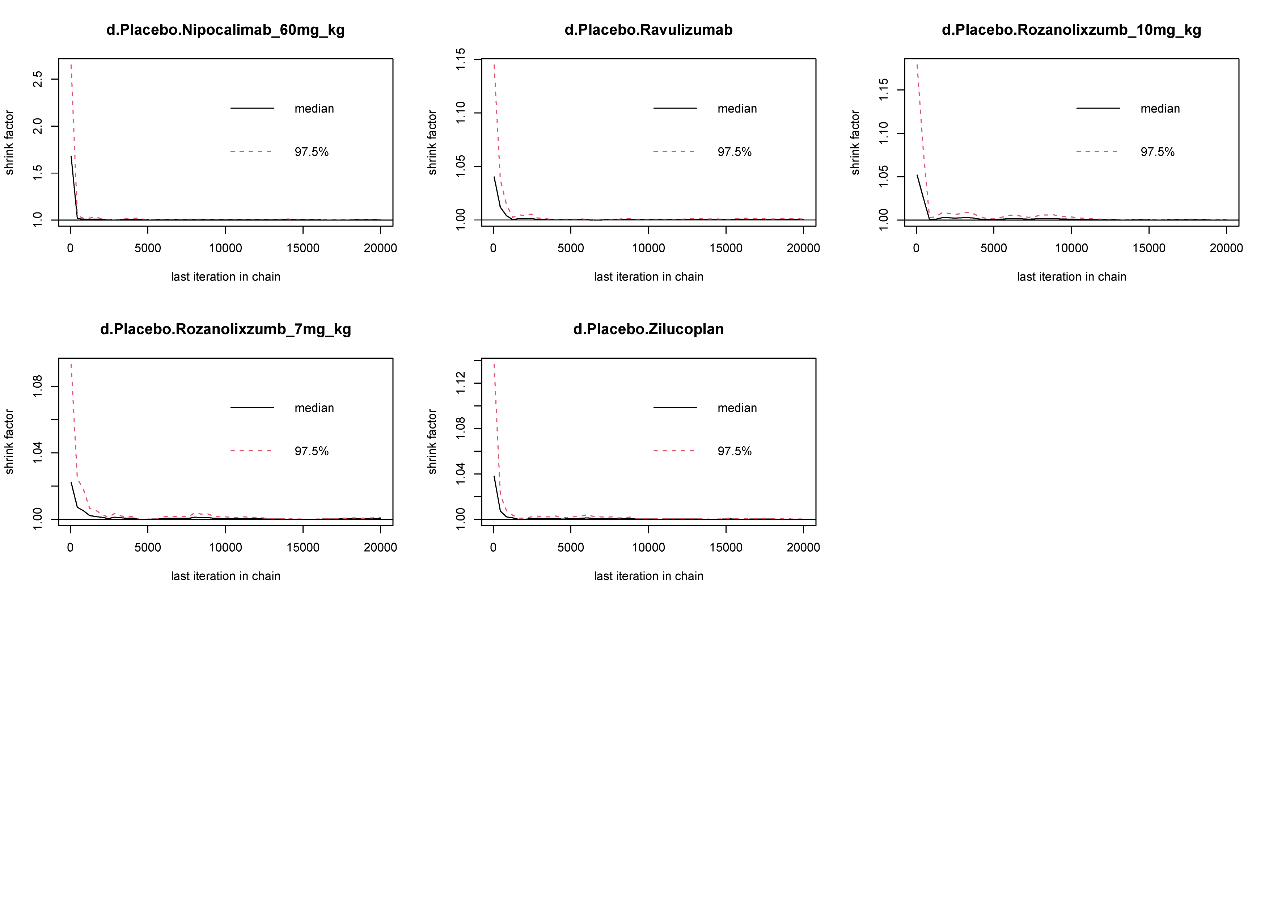
Figure S26: Convergence diagnostics of the network meta-analysis: AEs.**

**
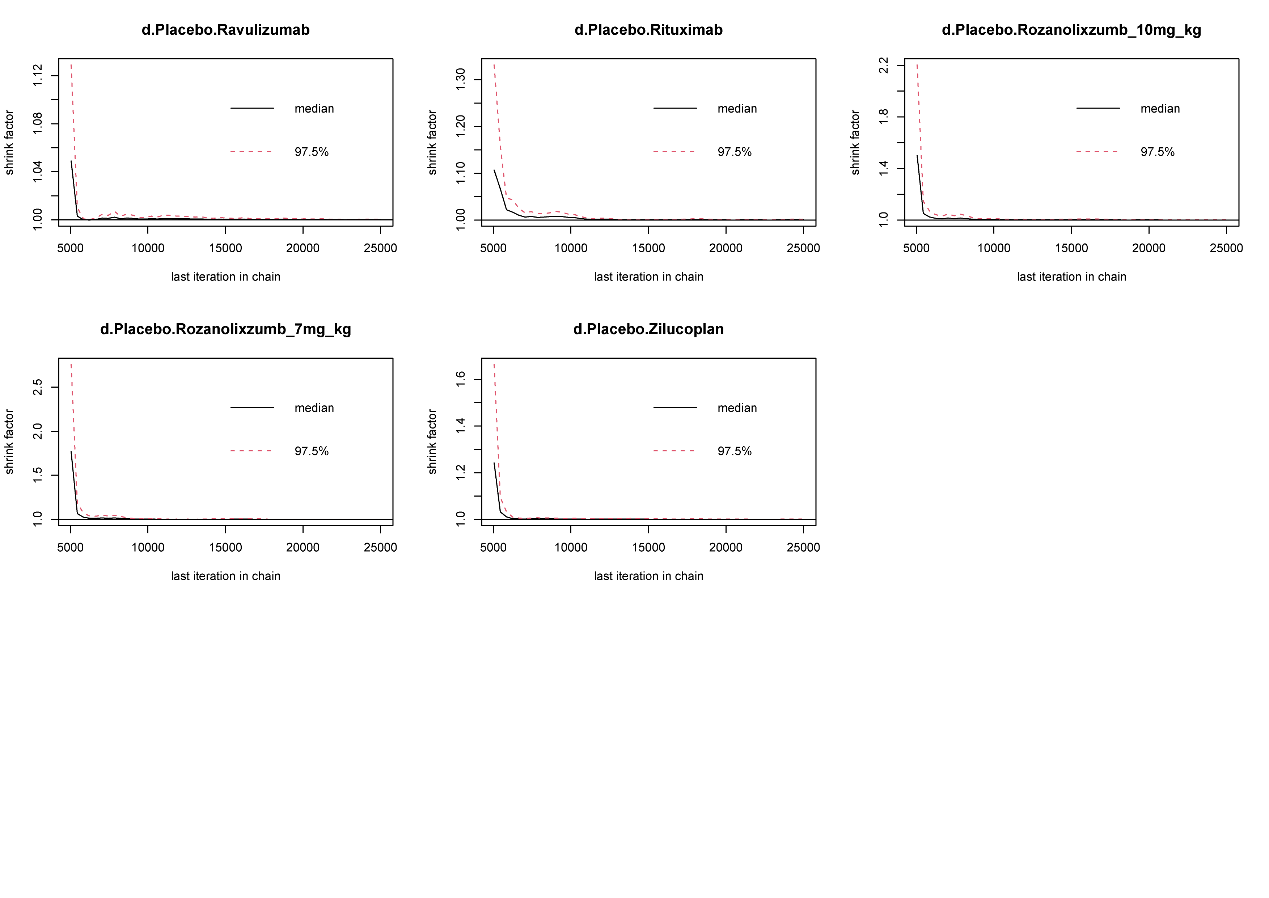
**

**
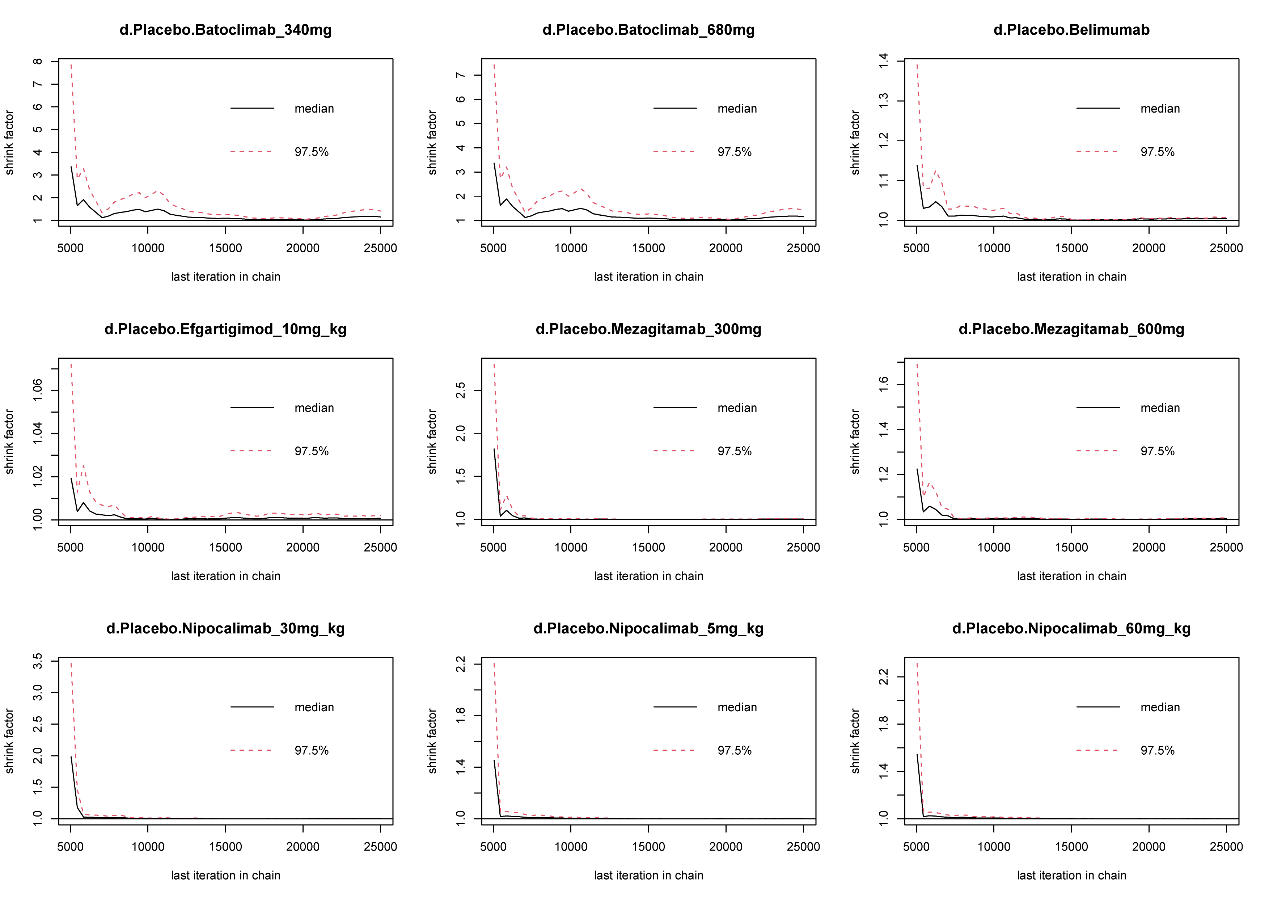
**

**Figure S27: Convergence diagnostics of the network meta-analysis: Headache.**

**
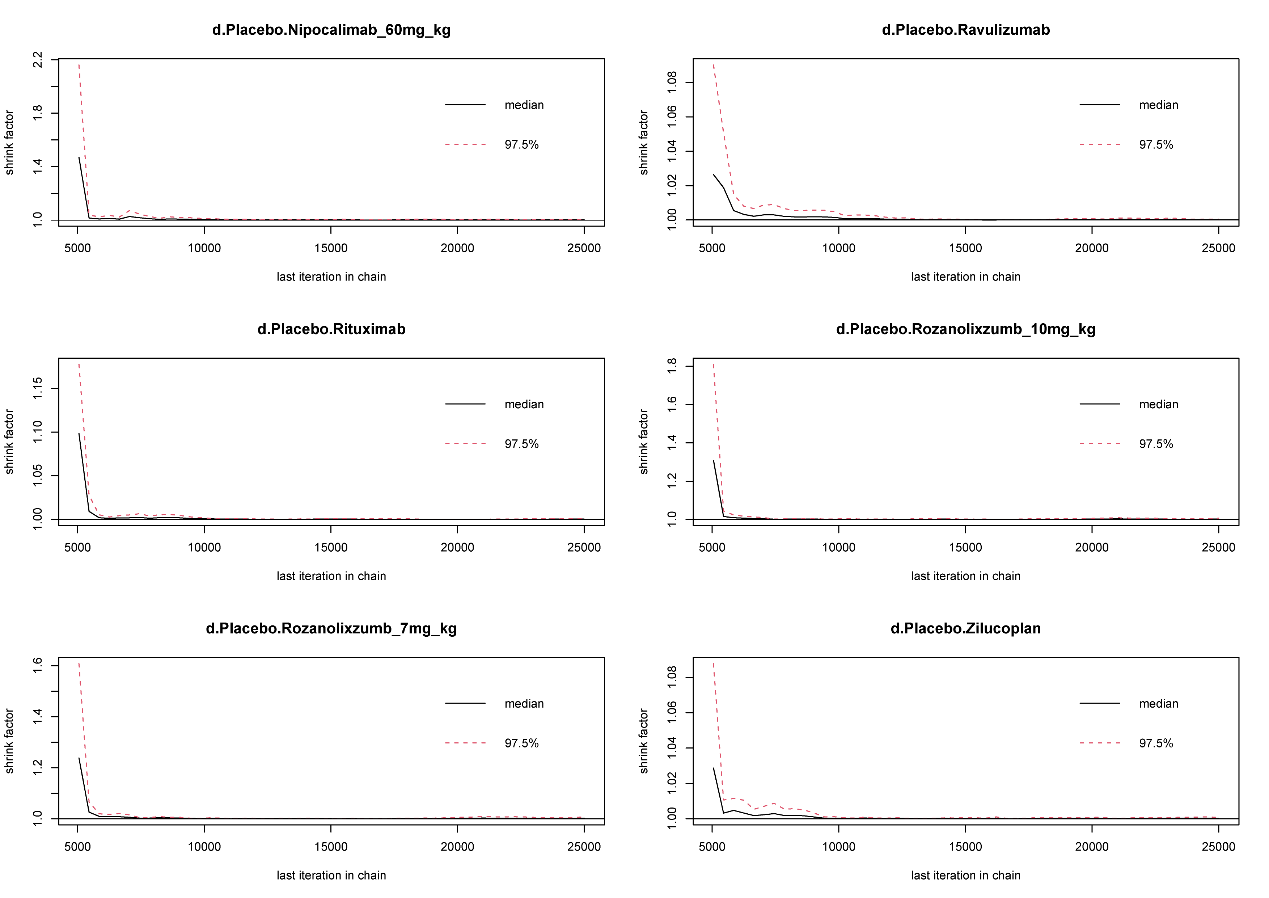
**

**Figure S28: Convergence diagnostics of the network meta-analysis: Diarrhea.**

**
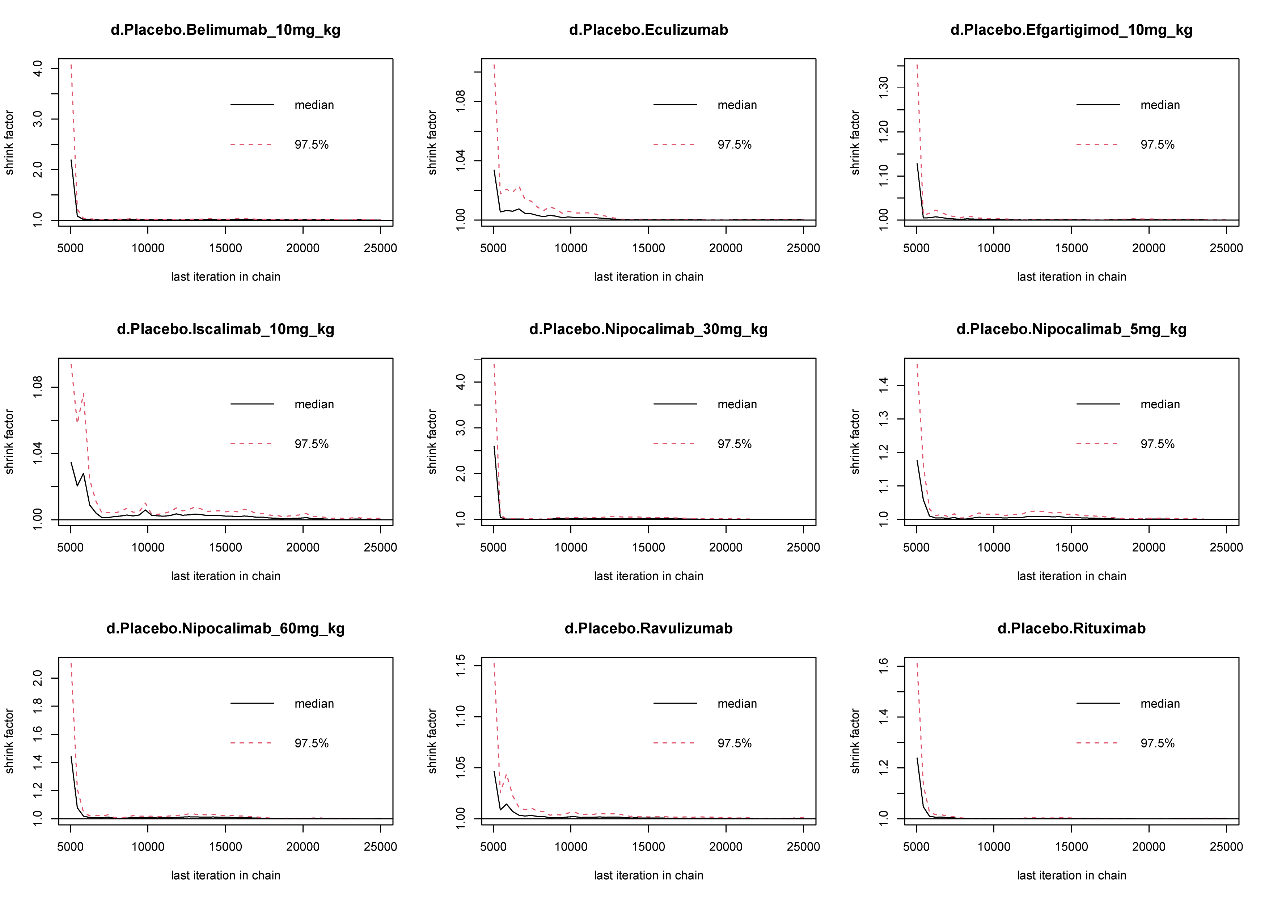
**

**
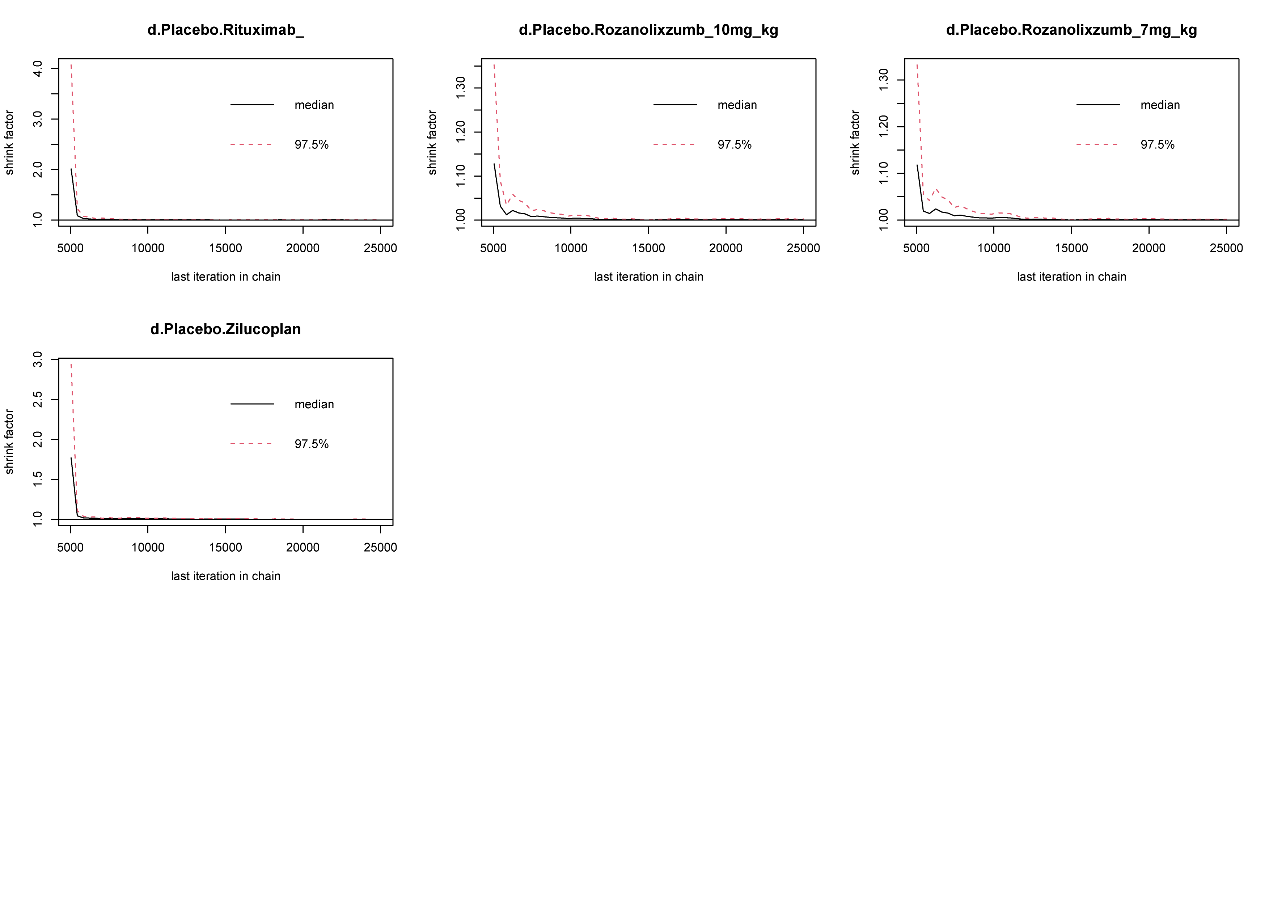
Figure S29: Convergence diagnostics of the network meta-analysis: Nausea.**

**
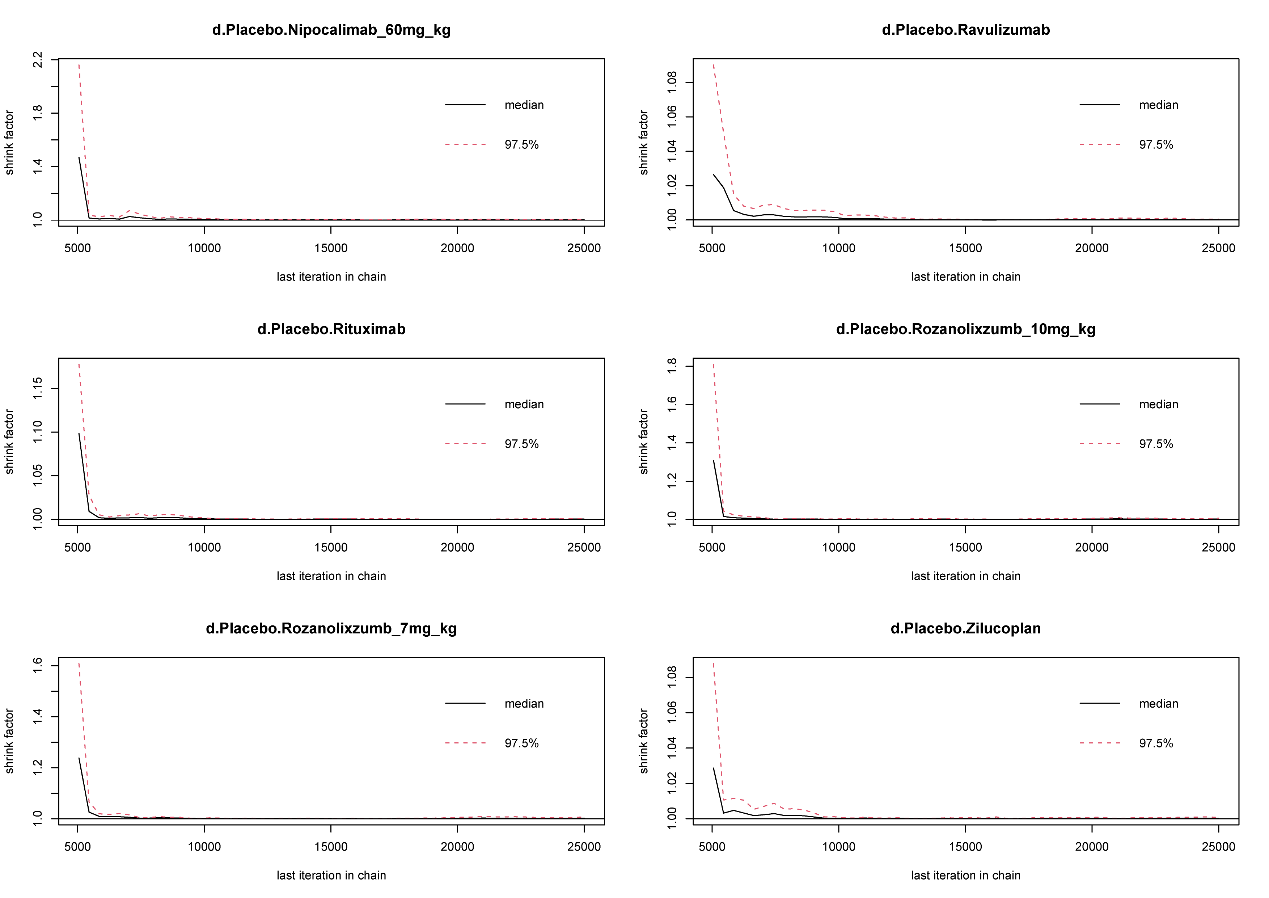
**

**Figure S30: Trance and density of the network meta-analysis: MG-ADL.**

**
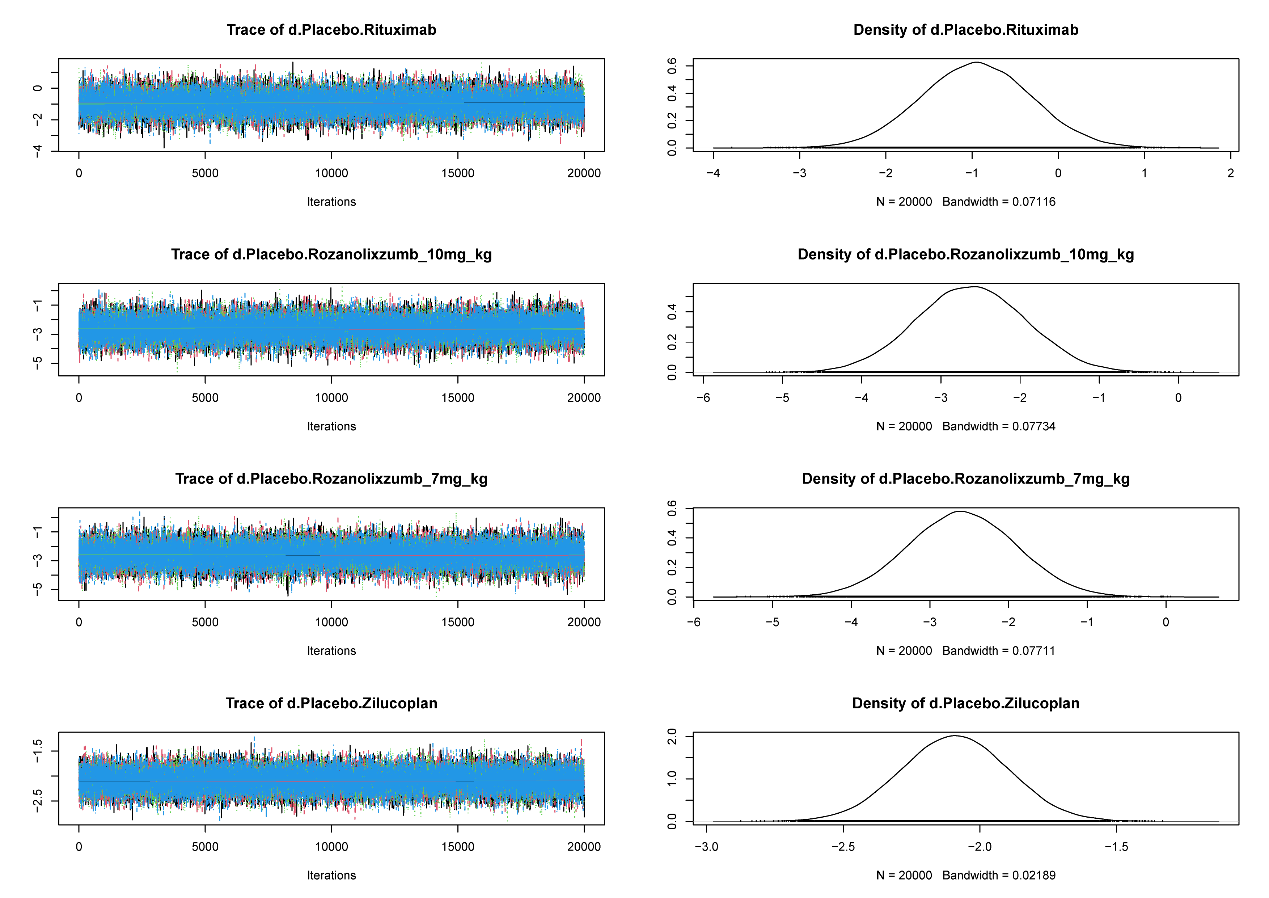
**

**Figure S31: Trance and density of the network meta-analysis: QMG.**

**
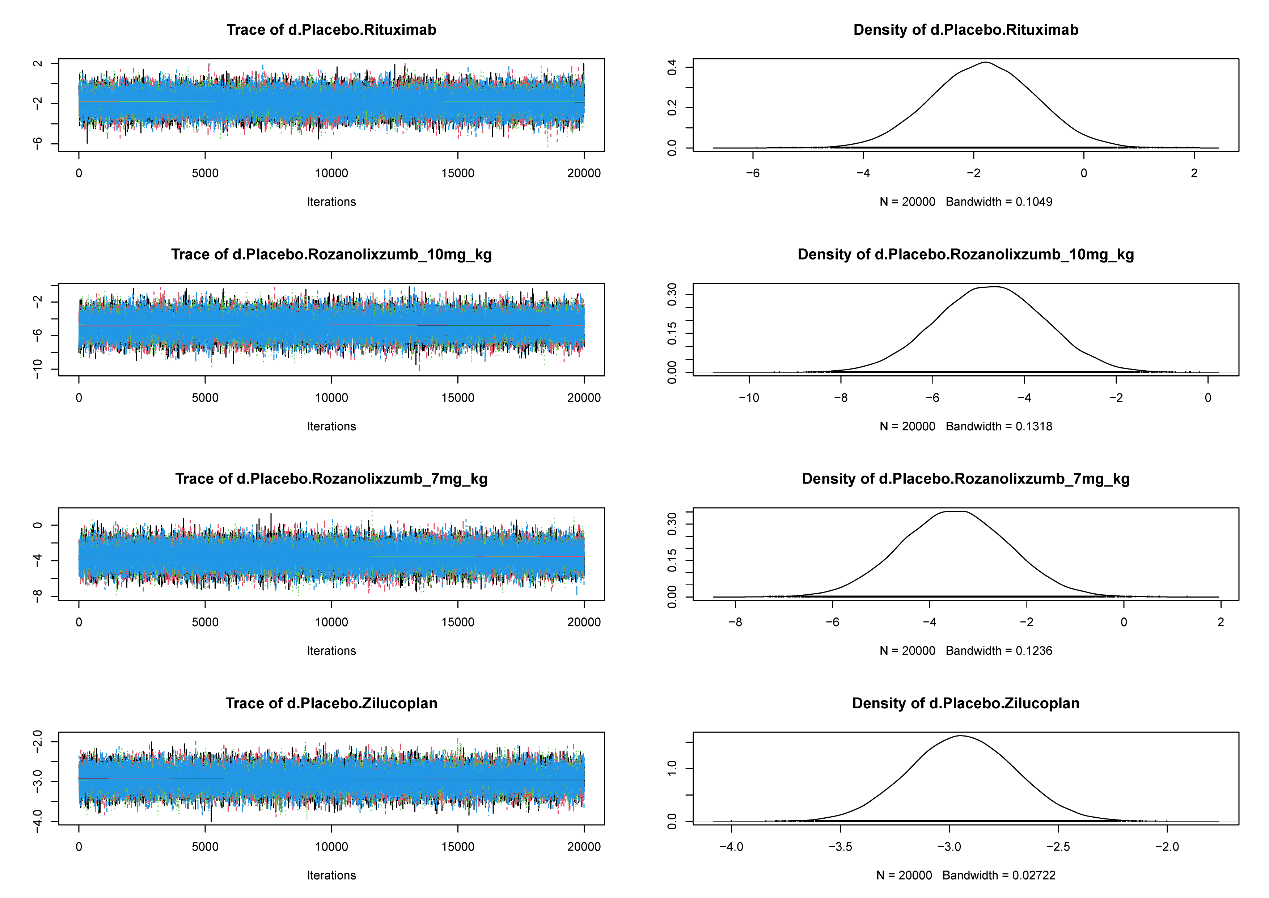
**

**Figure S32: Trance and density of the network meta-analysis: MGC.**

**
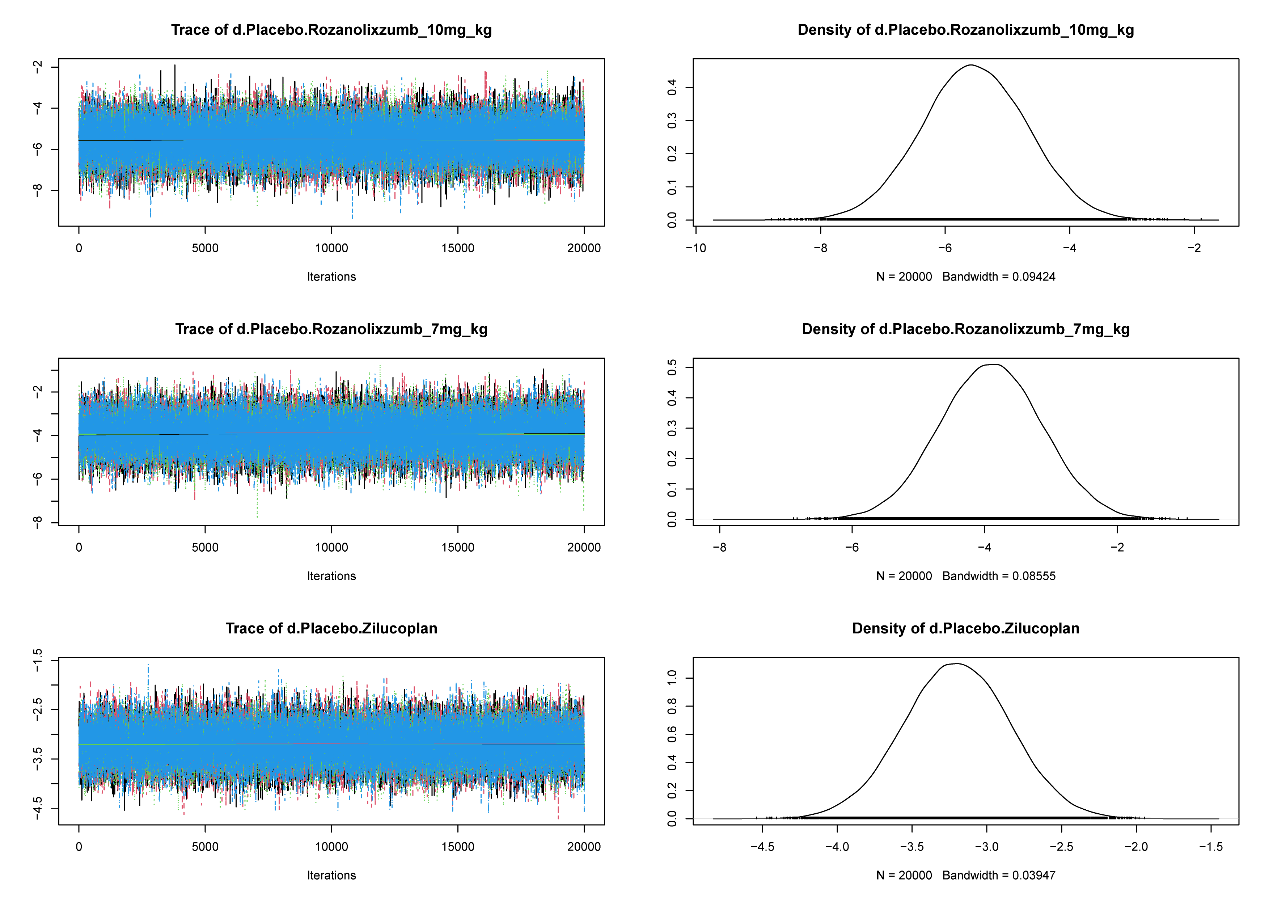
**

**Figure S33: Trance and density of the network meta-analysis: MG-QoL 15r.**

**
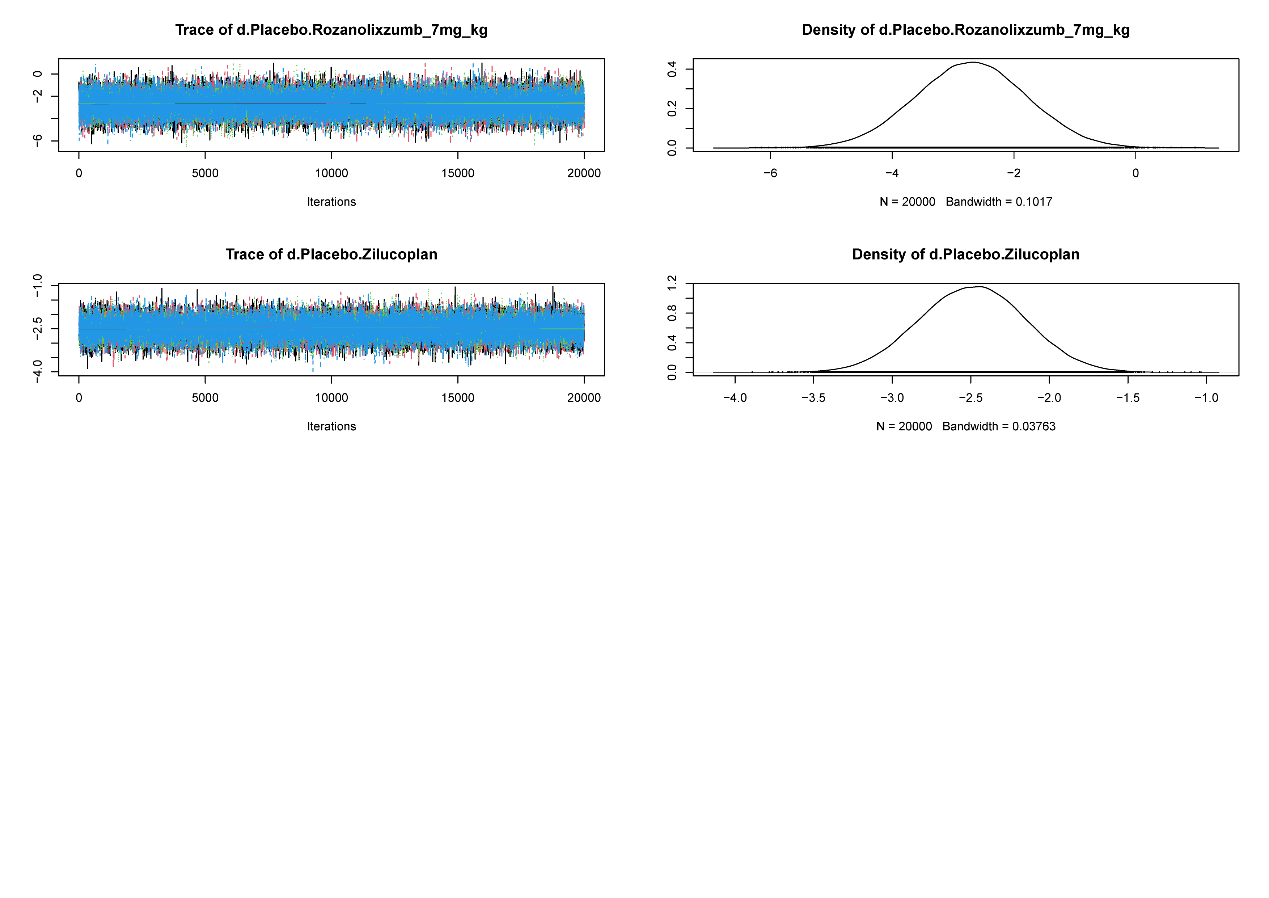
**

**Figure S34: Trance and density of the network meta-analysis: AEs.**

**
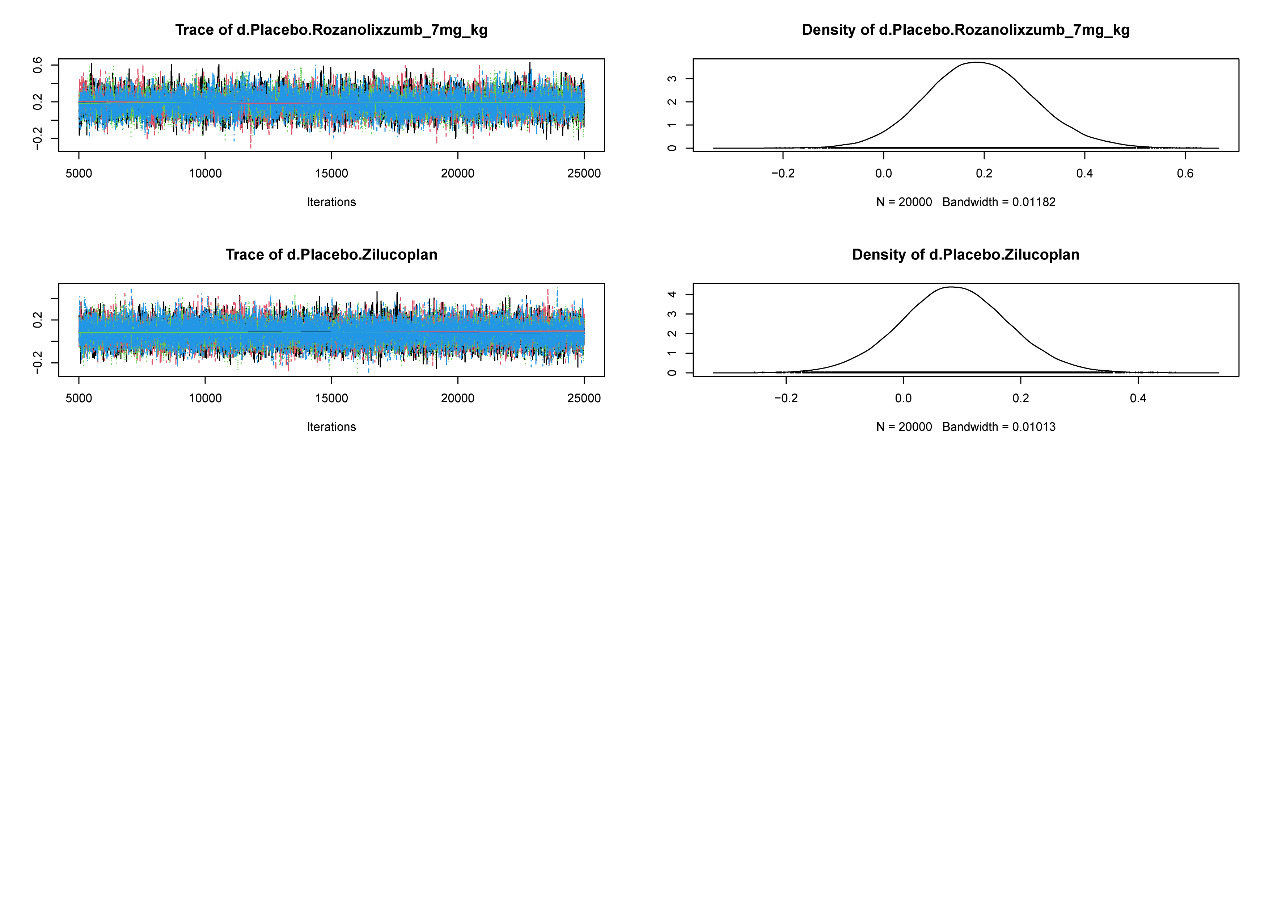
**

**Figure S35: Trance and density of the network meta-analysis: Headache.**

**
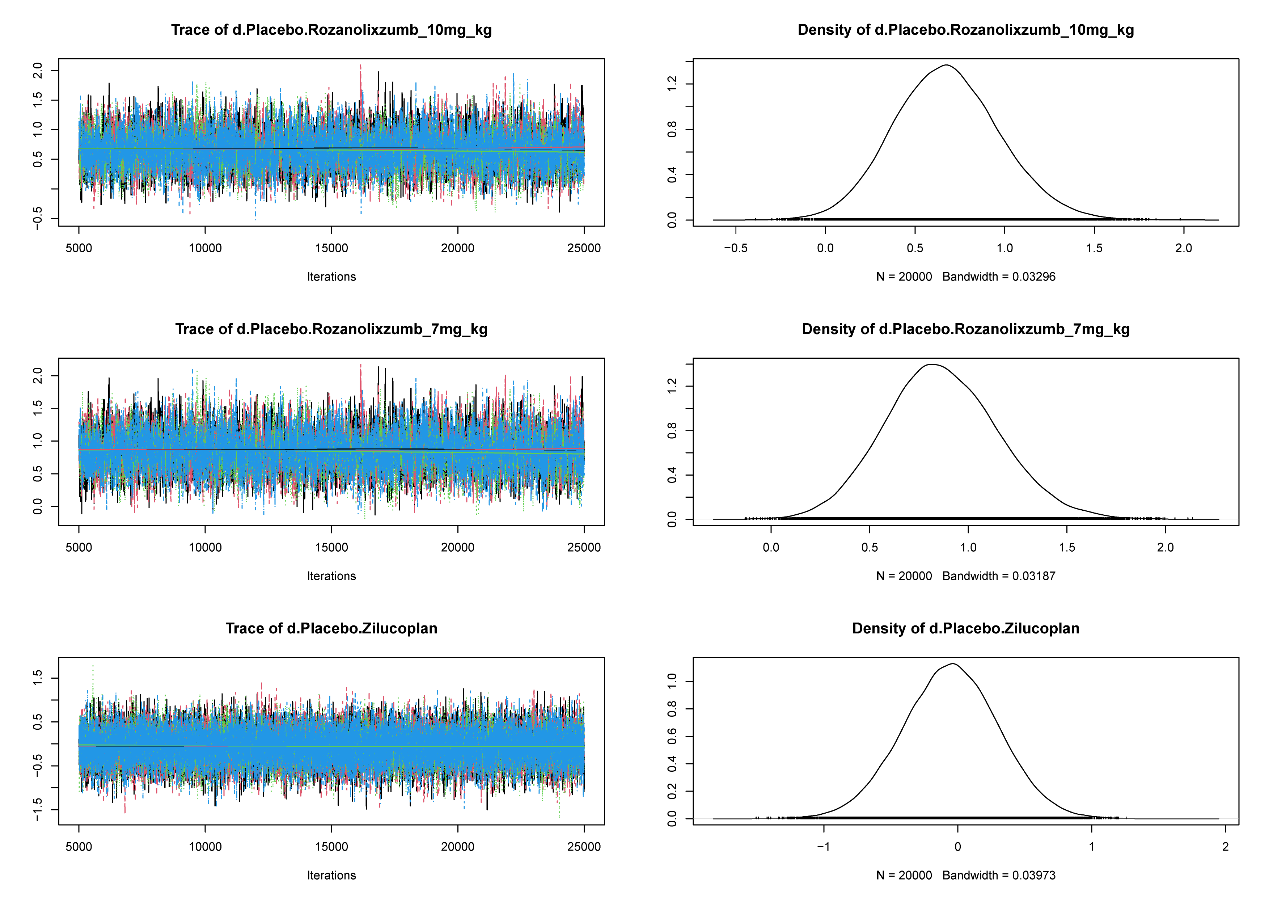
**

**Figure S36: Trance and density of the network meta-analysis: Diarrhea.**

**
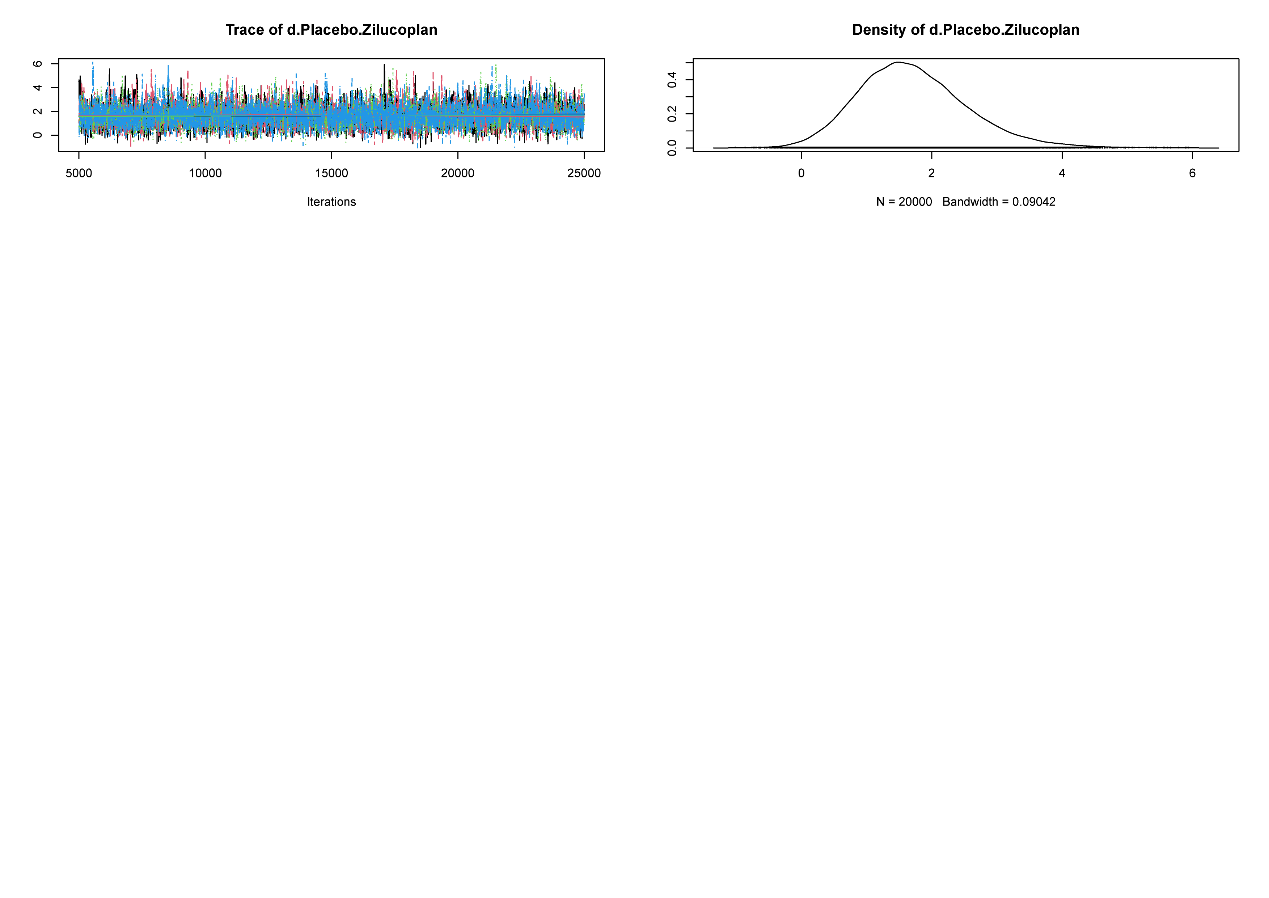
Figure S37: Trance and density of the network meta-analysis: Nausea.**

**
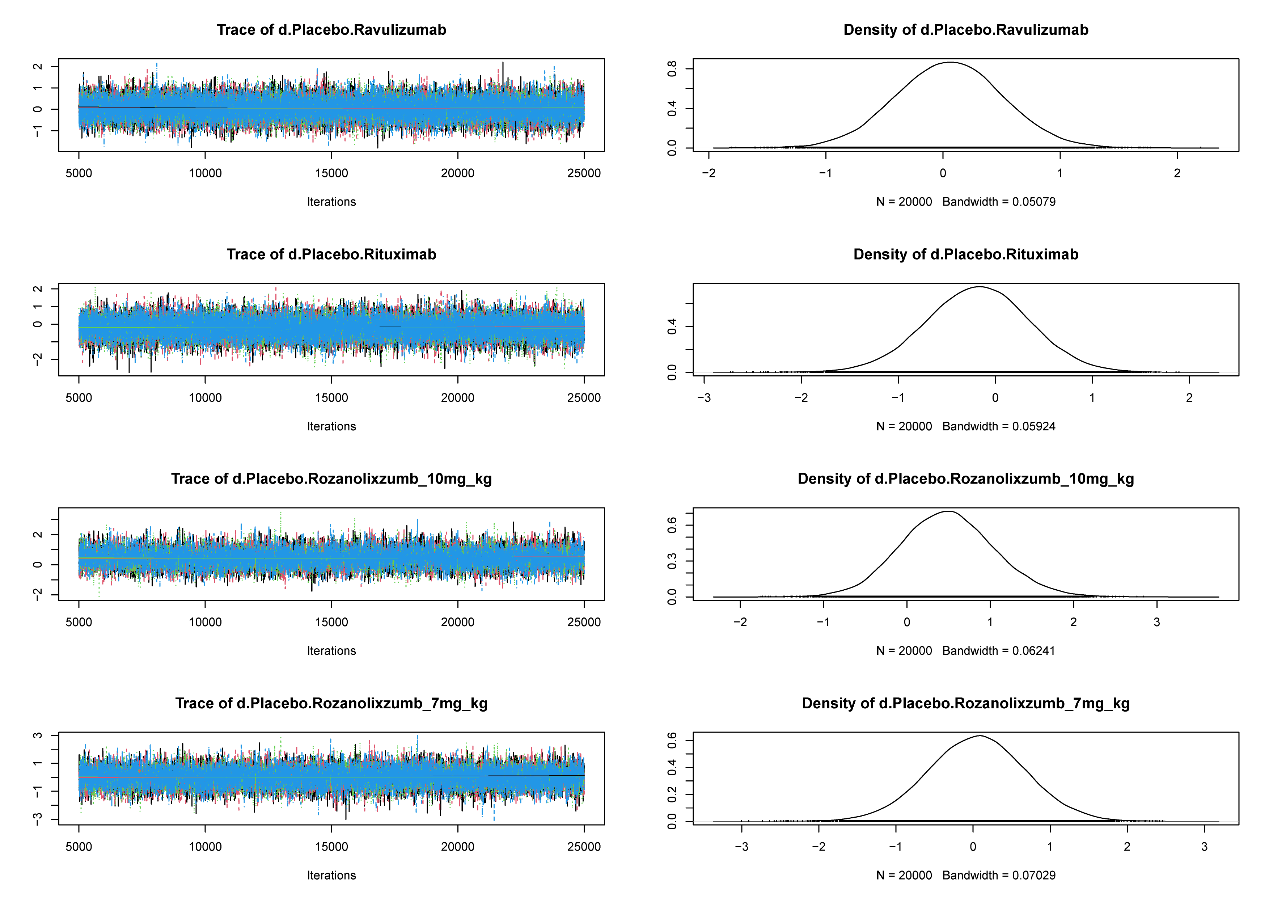
**

**Figure S38: Forest plots for the heterogeneity: MD-ADL**

**
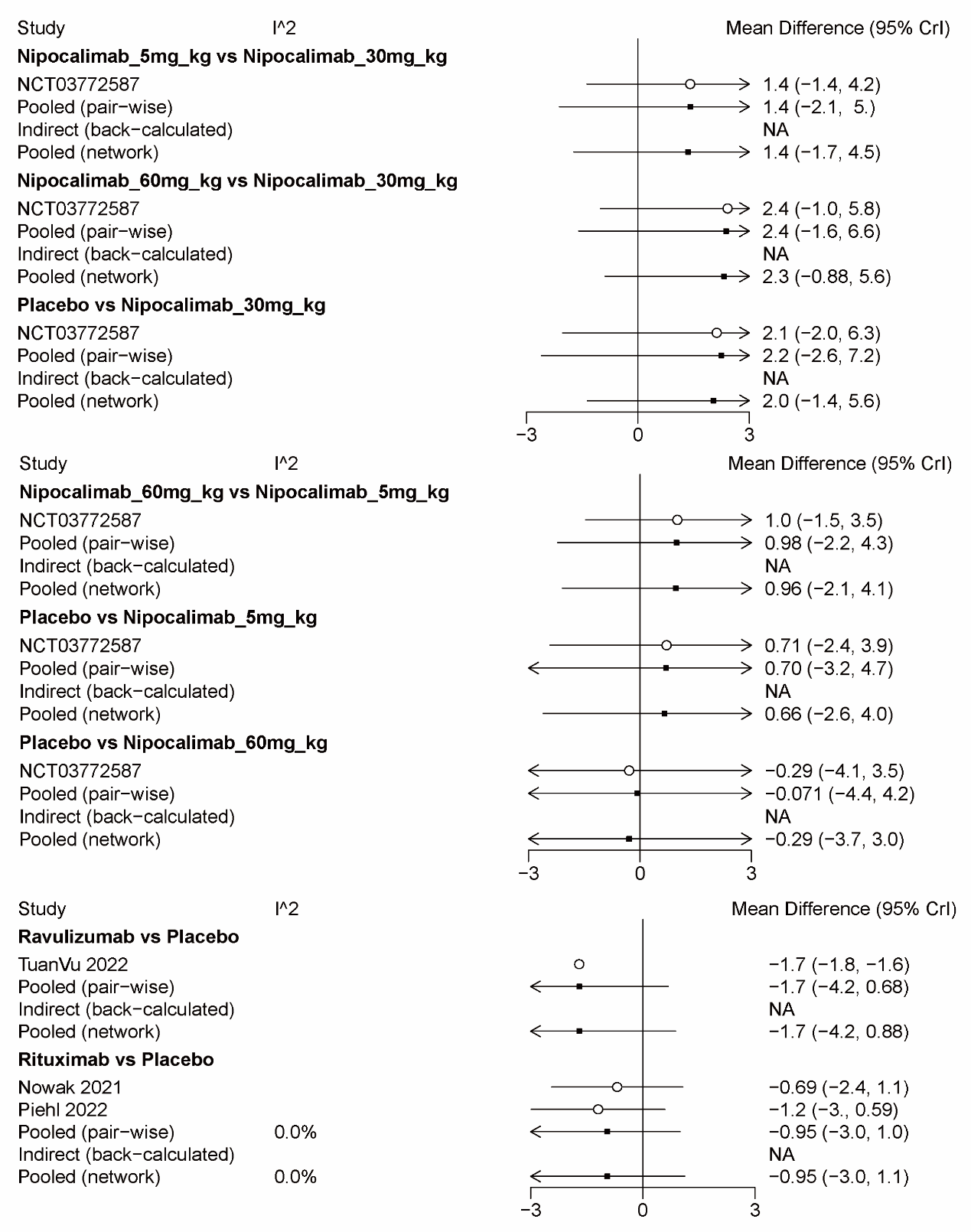
**

**
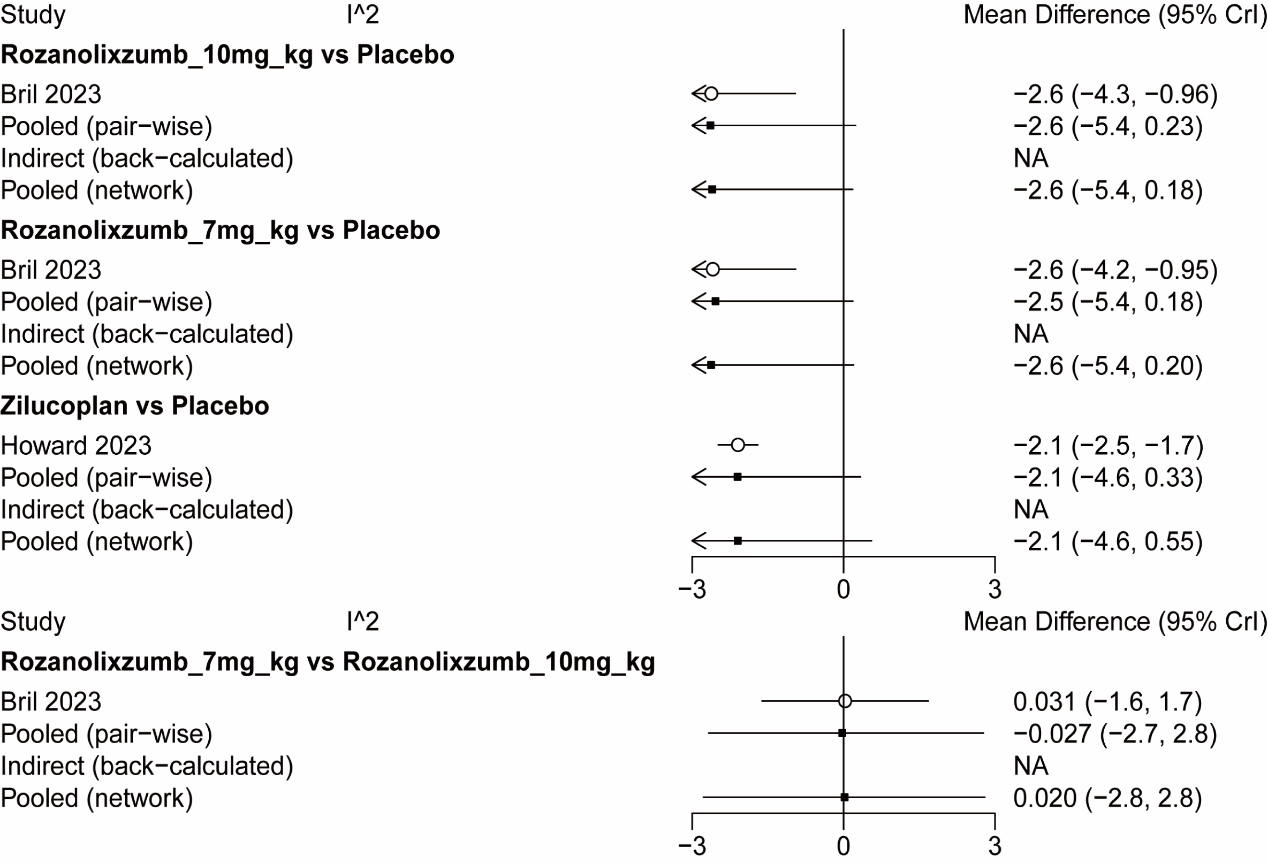
**

**Figure S39: Forest plots for the heterogeneity: QMG**

**
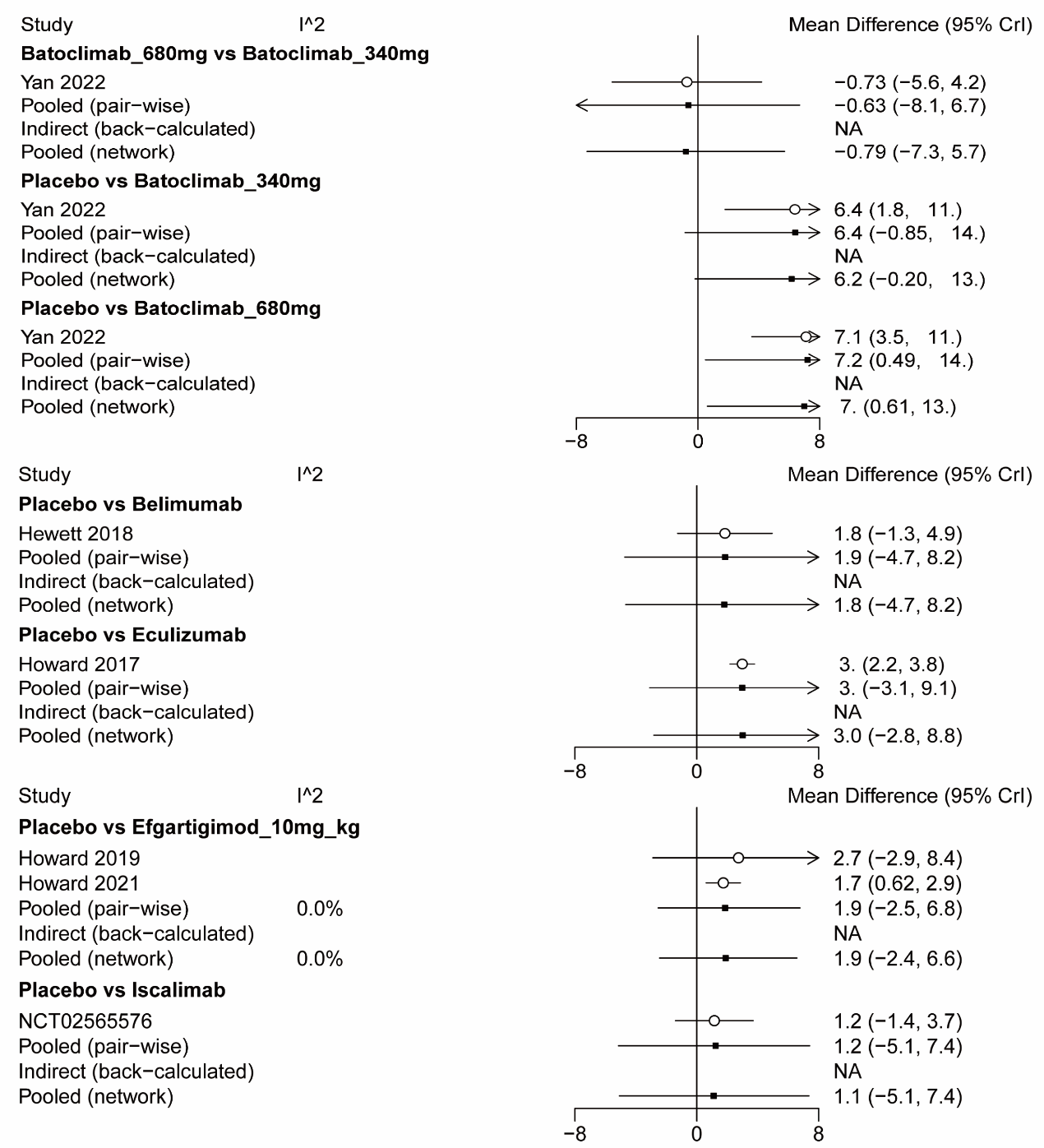
**

**
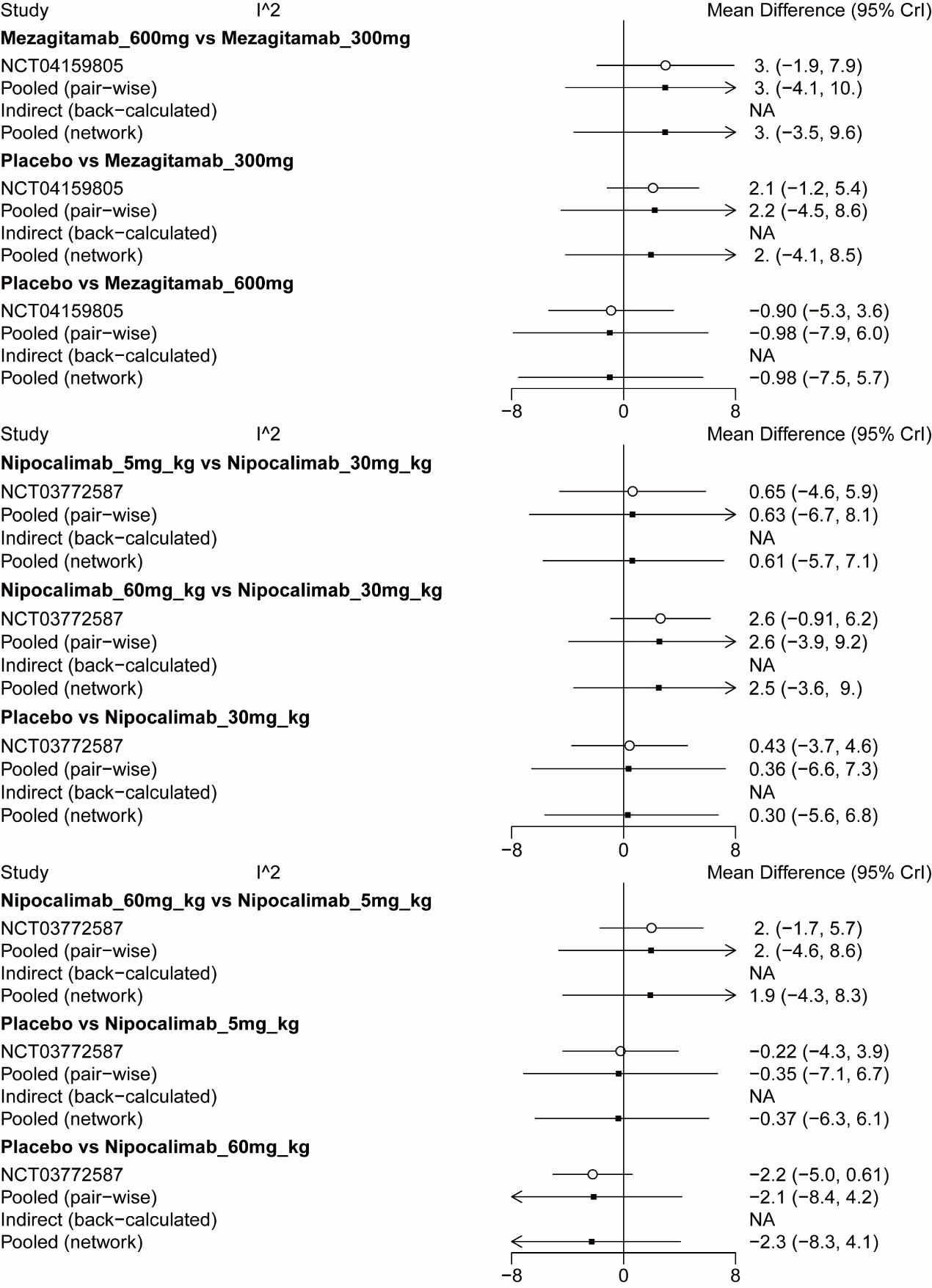
**

**
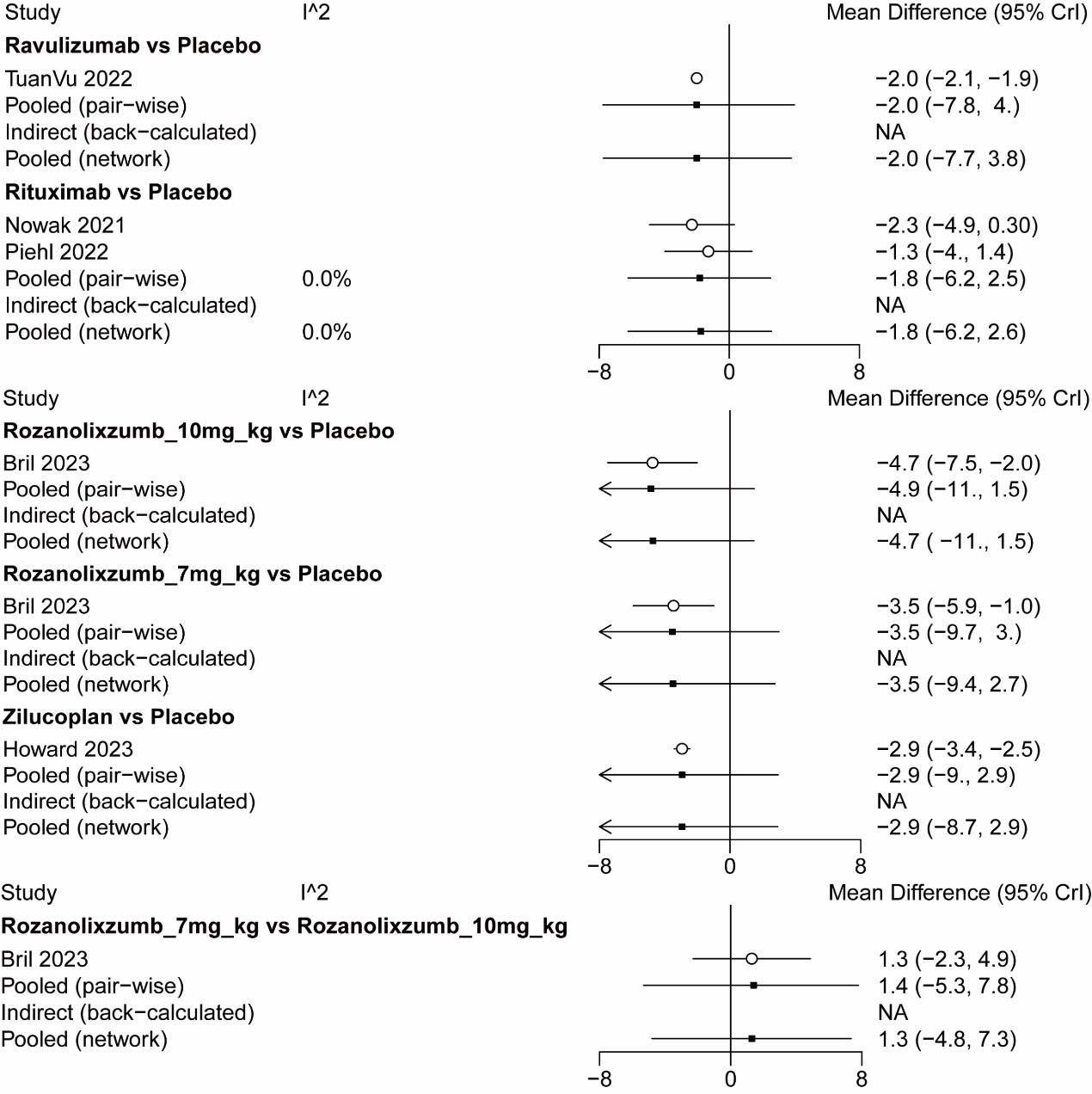
**

**Figure S40: Forest plots for the heterogeneity: MGC**

**
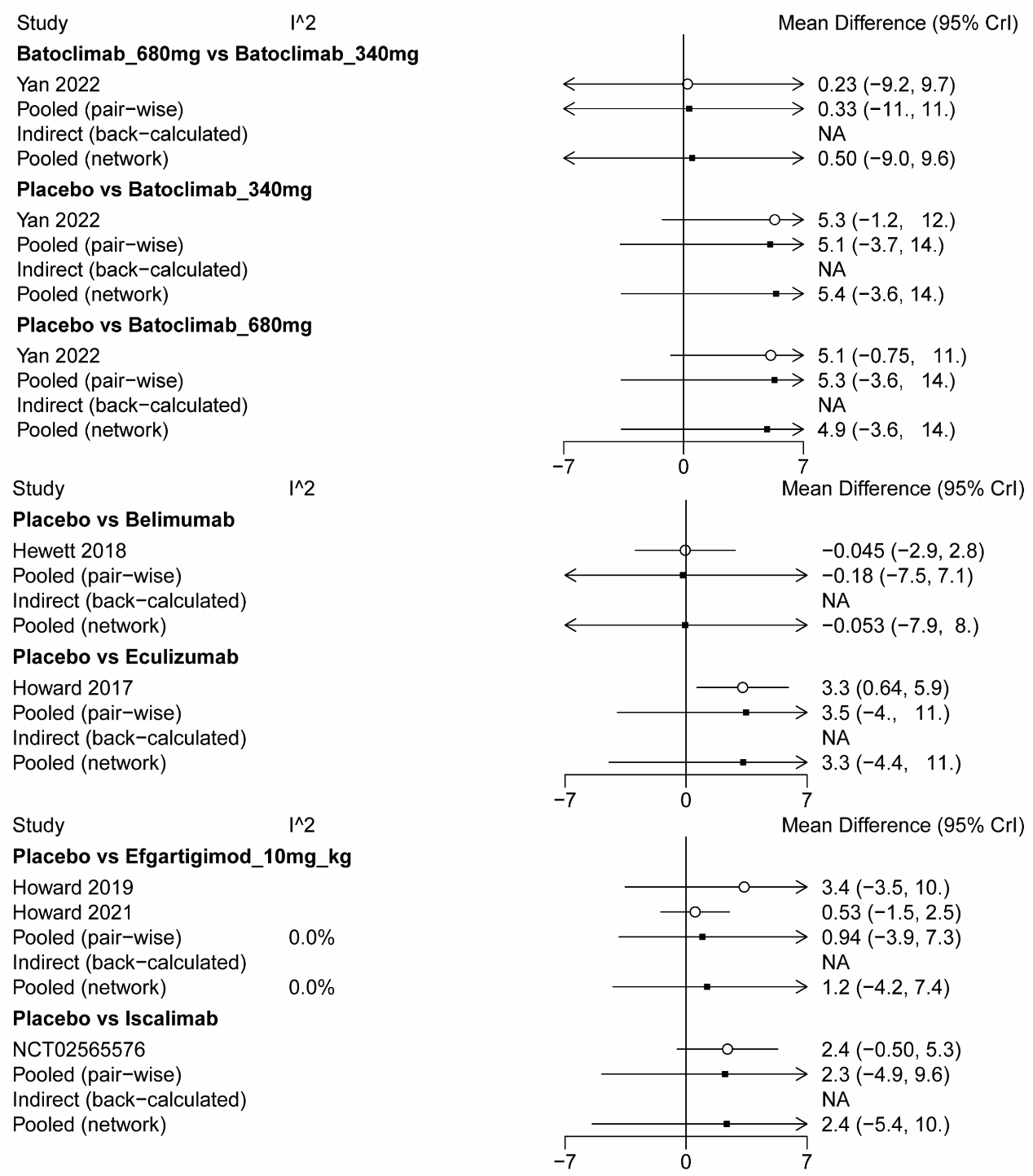
**

**
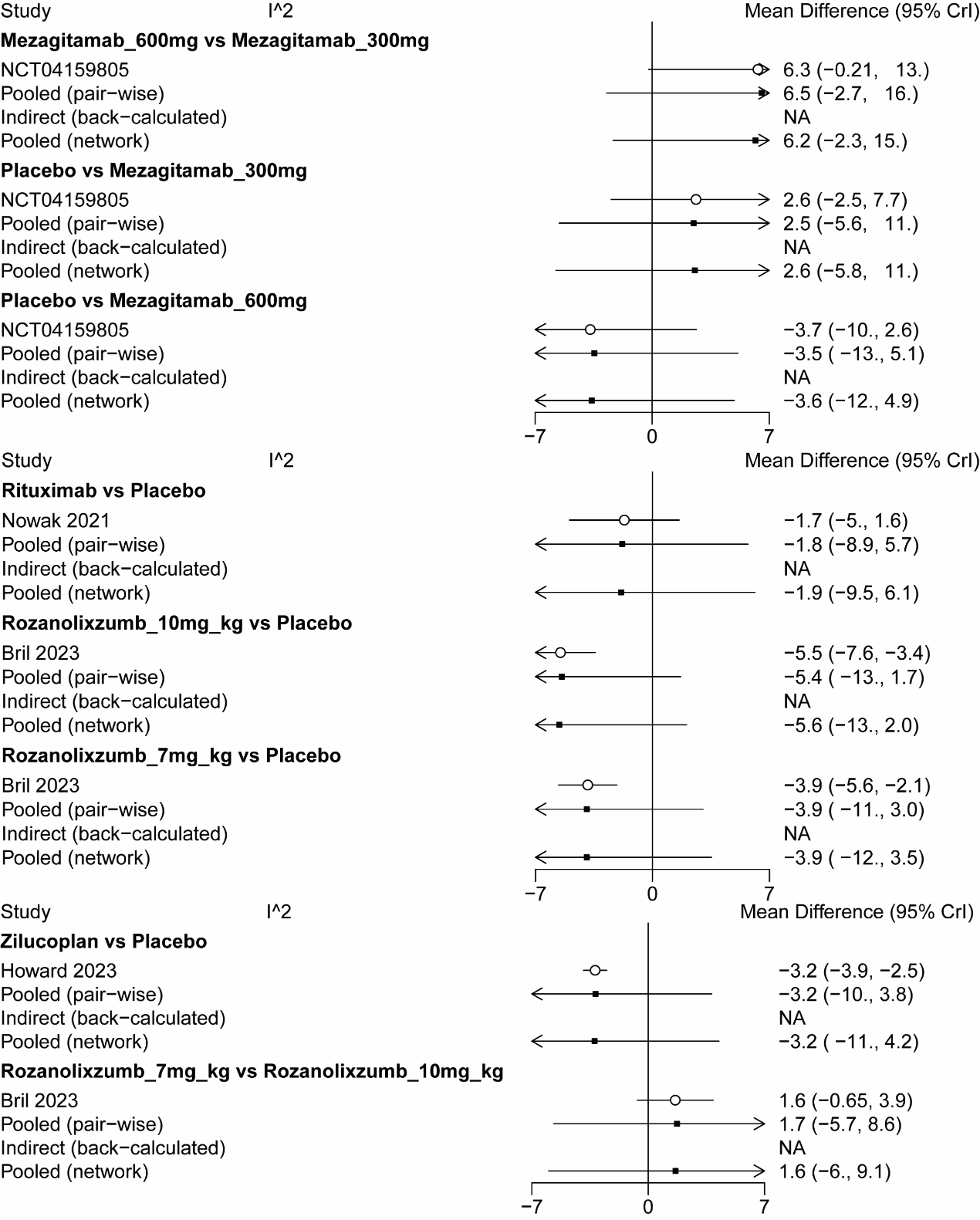
**

**Figure S41: Forest plots for the heterogeneity: MG-QoL 15r.**

**
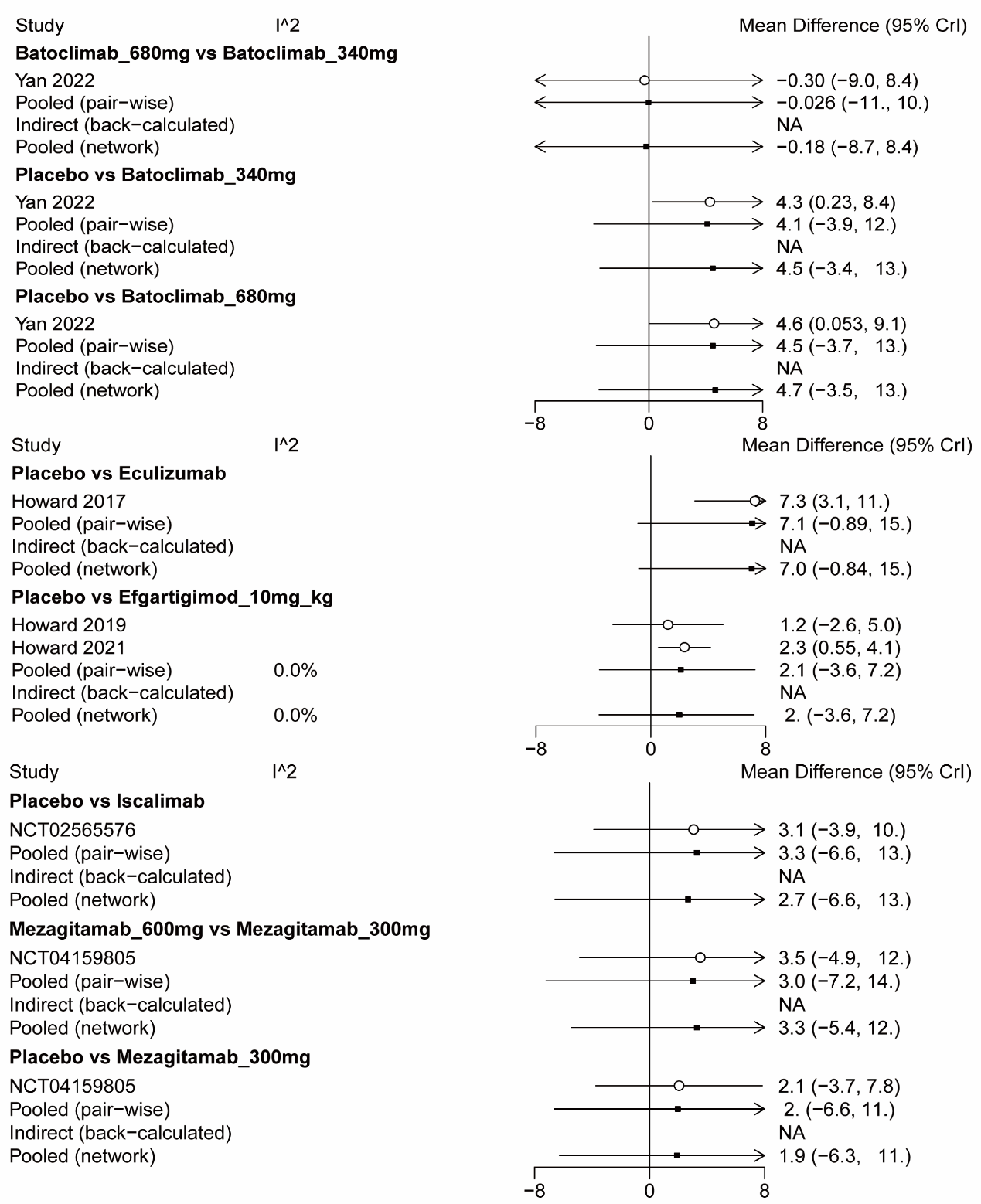
**

**
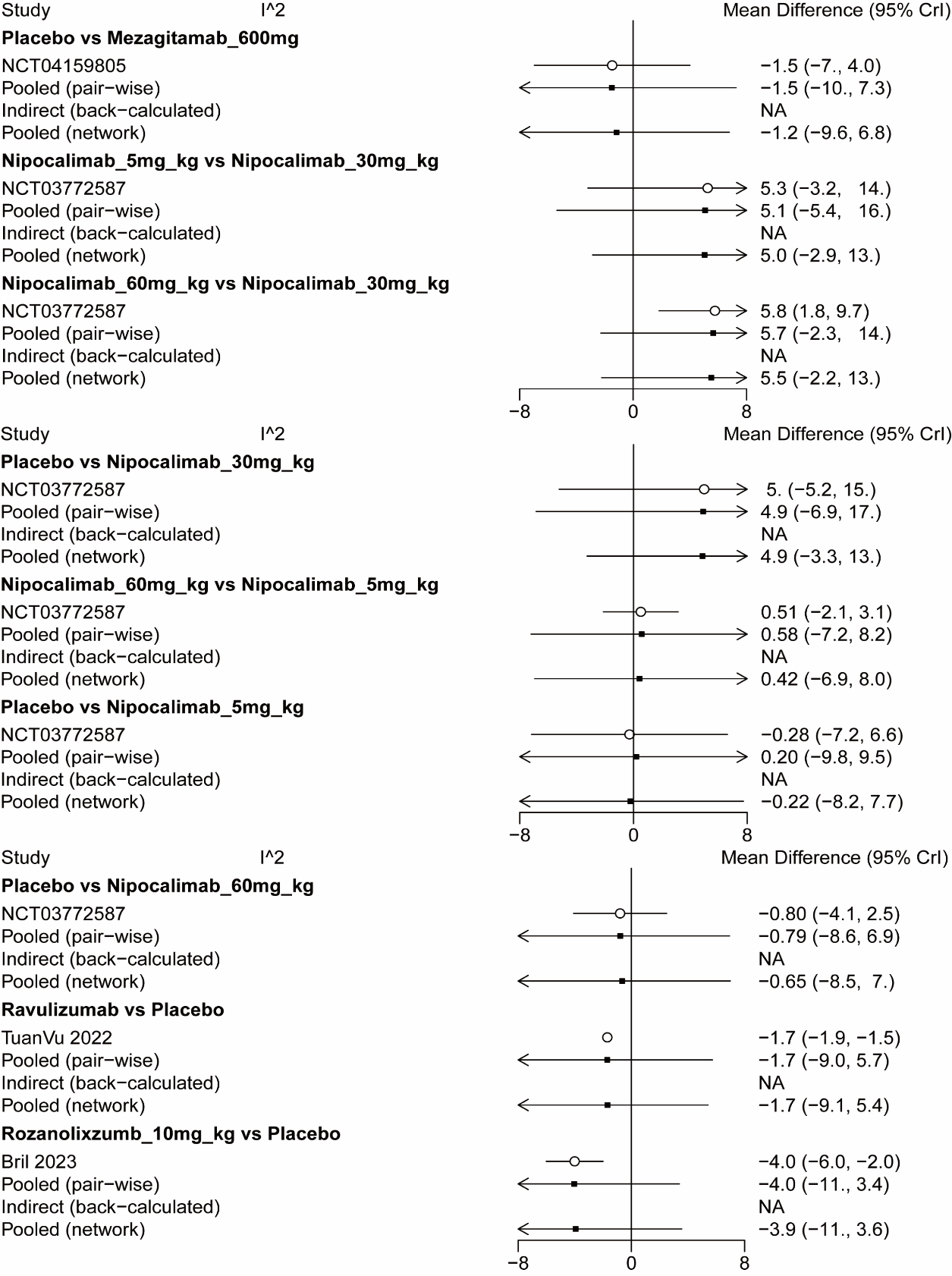
**

**
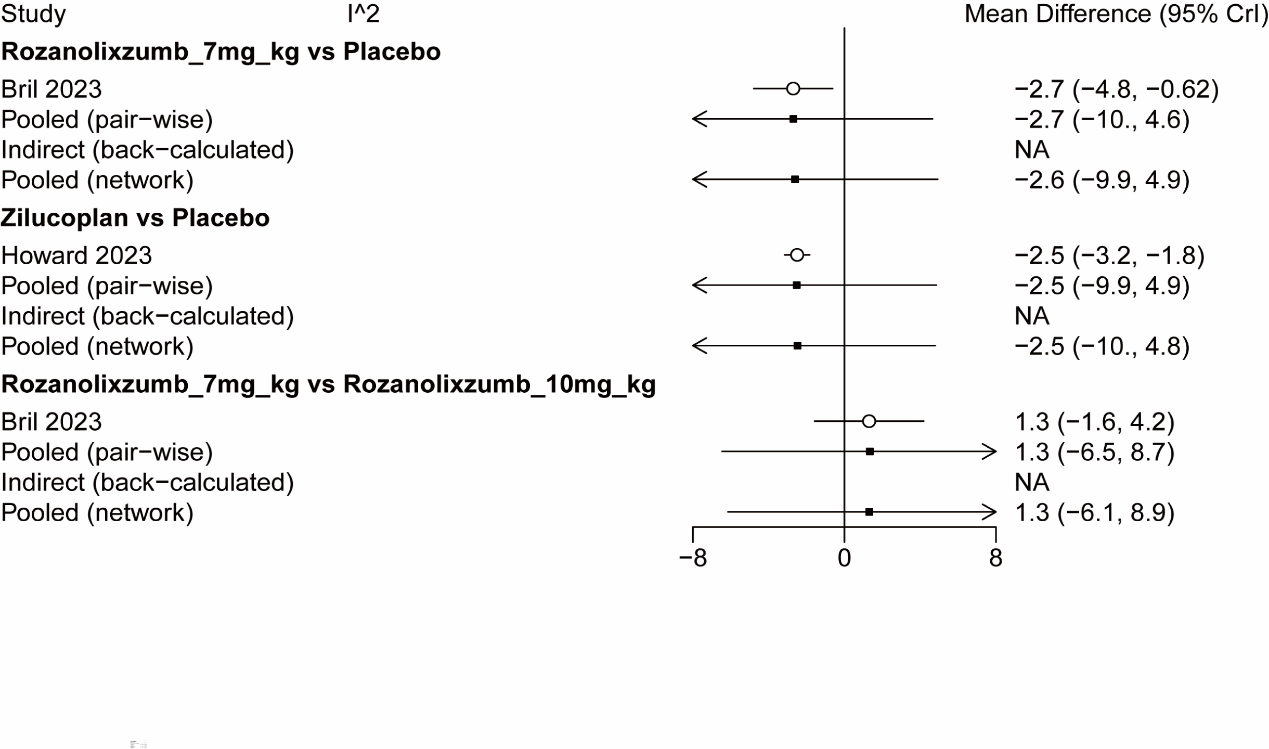
**

**Figure S42: Forest plots for the** **heterogeneity: AEs**

**
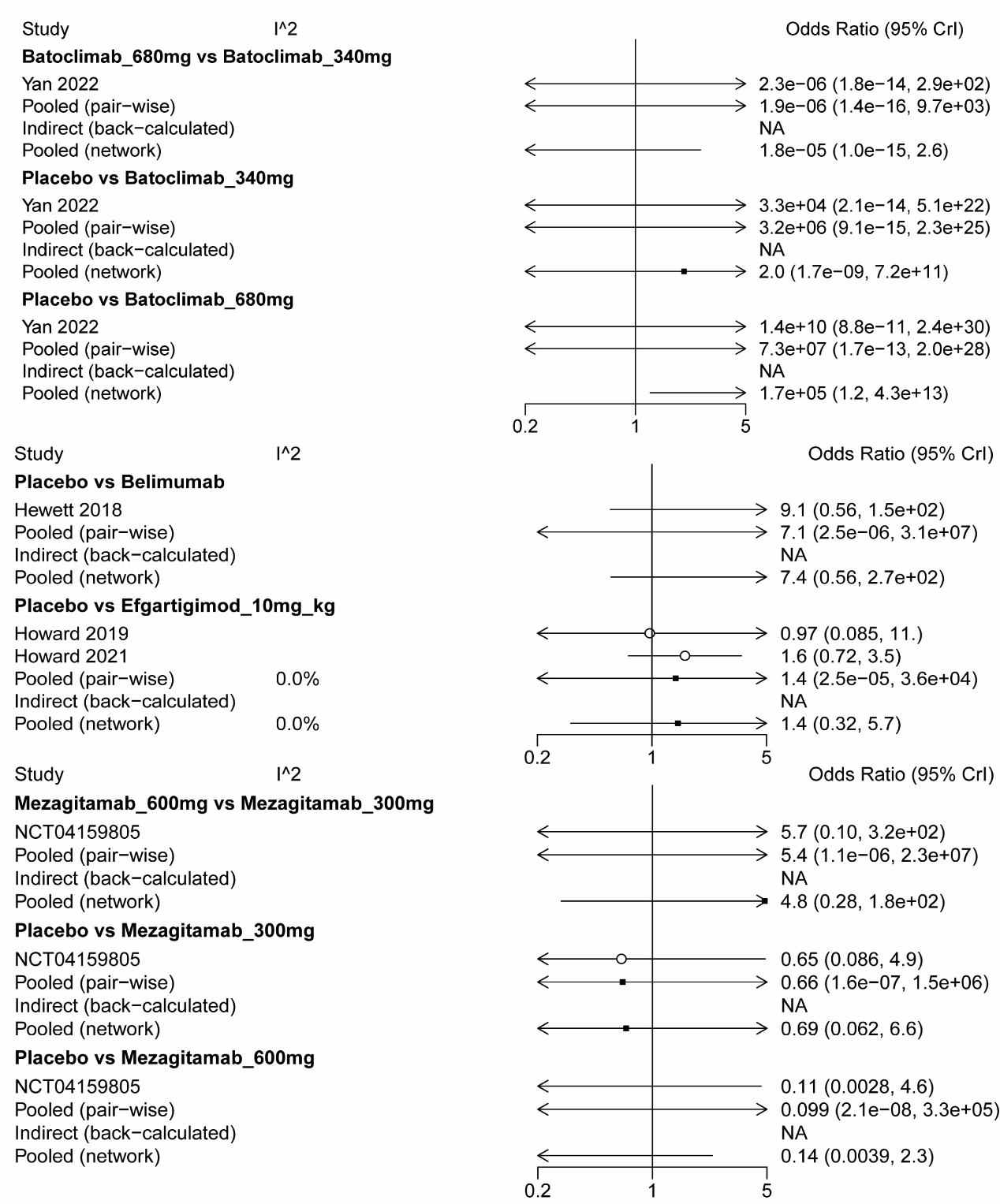
**

**
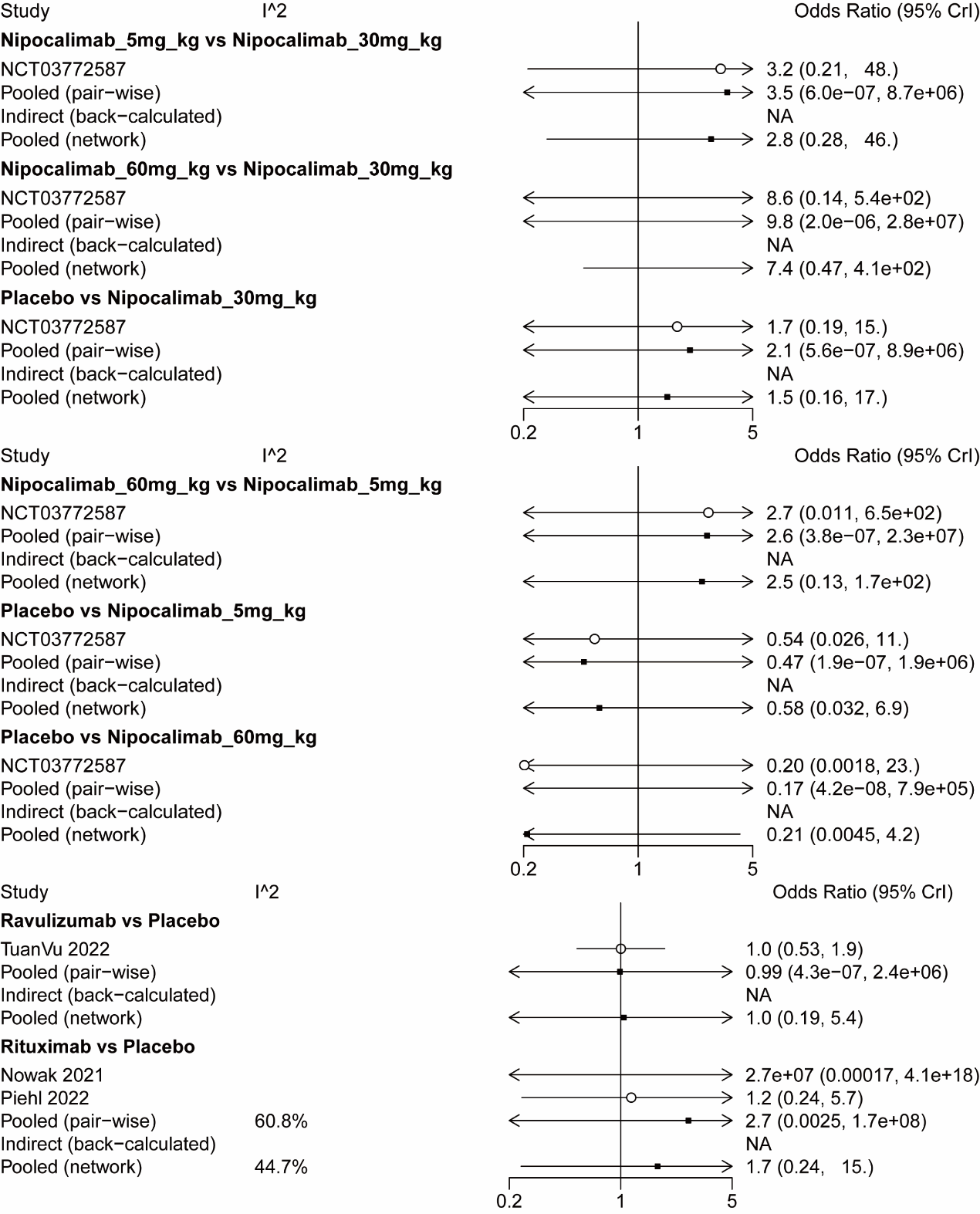
**

**
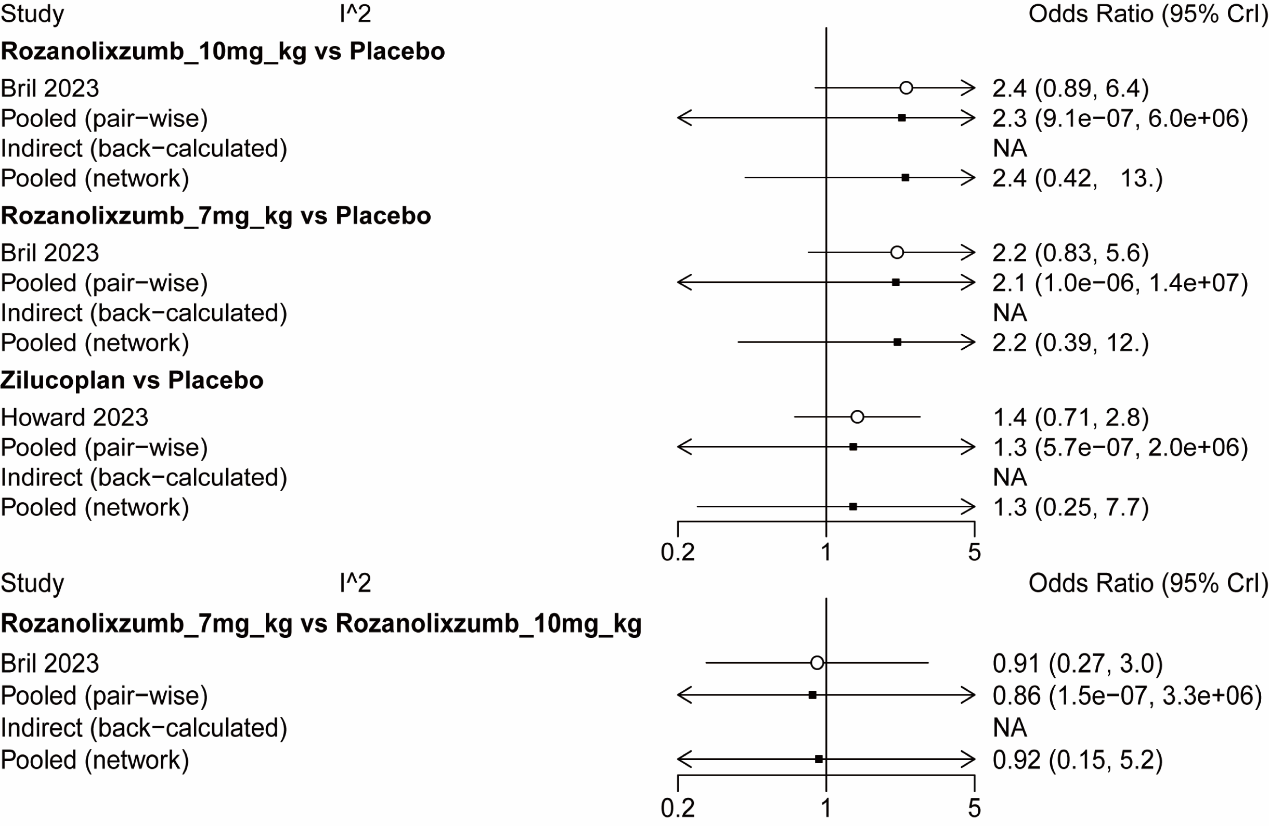
**

**Figure S43: Forest plots for the heterogeneity: Headache.**

**
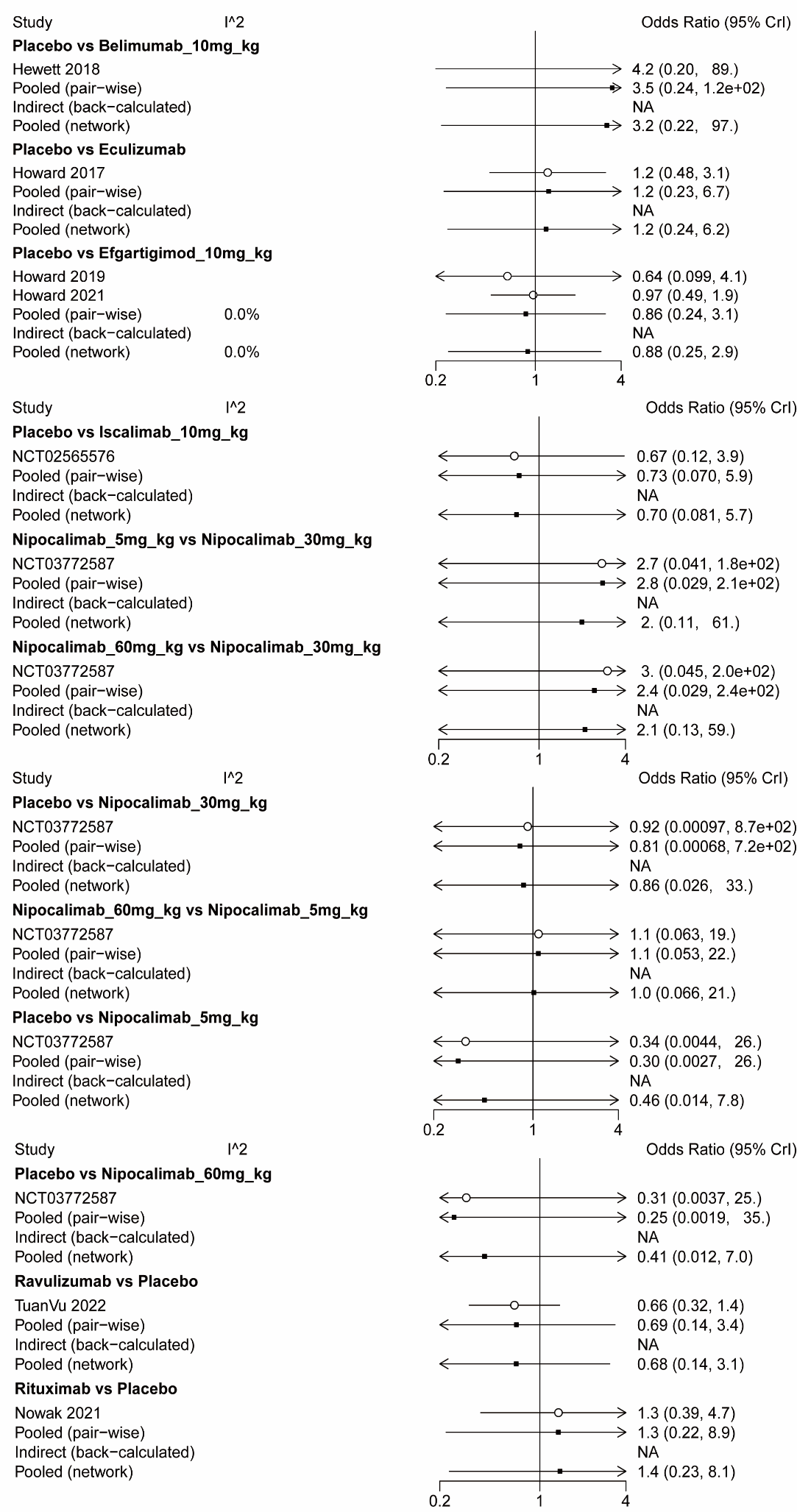
**

**
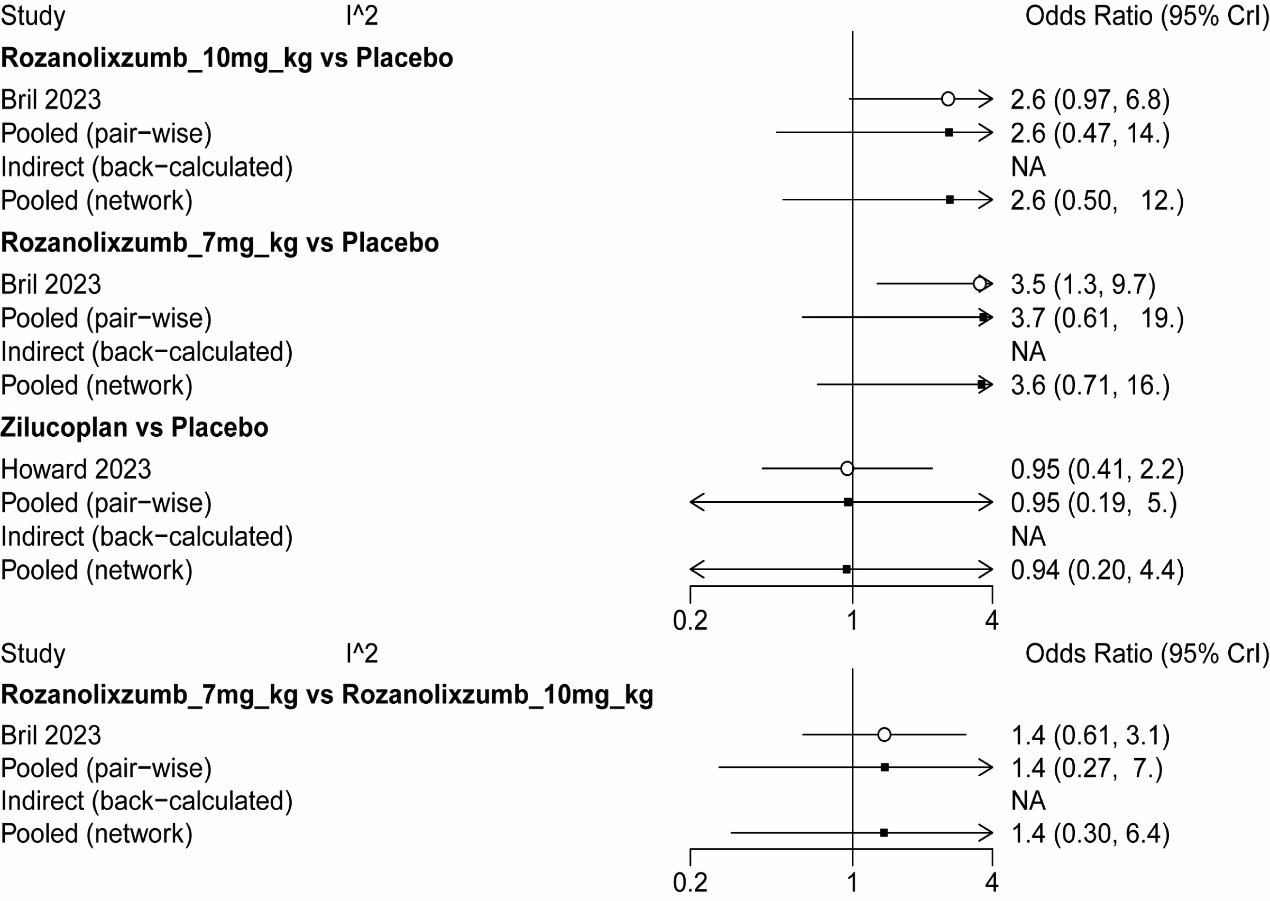
**

**Figure S44: Forest plots for the heterogeneity: Diarrhea.**

**
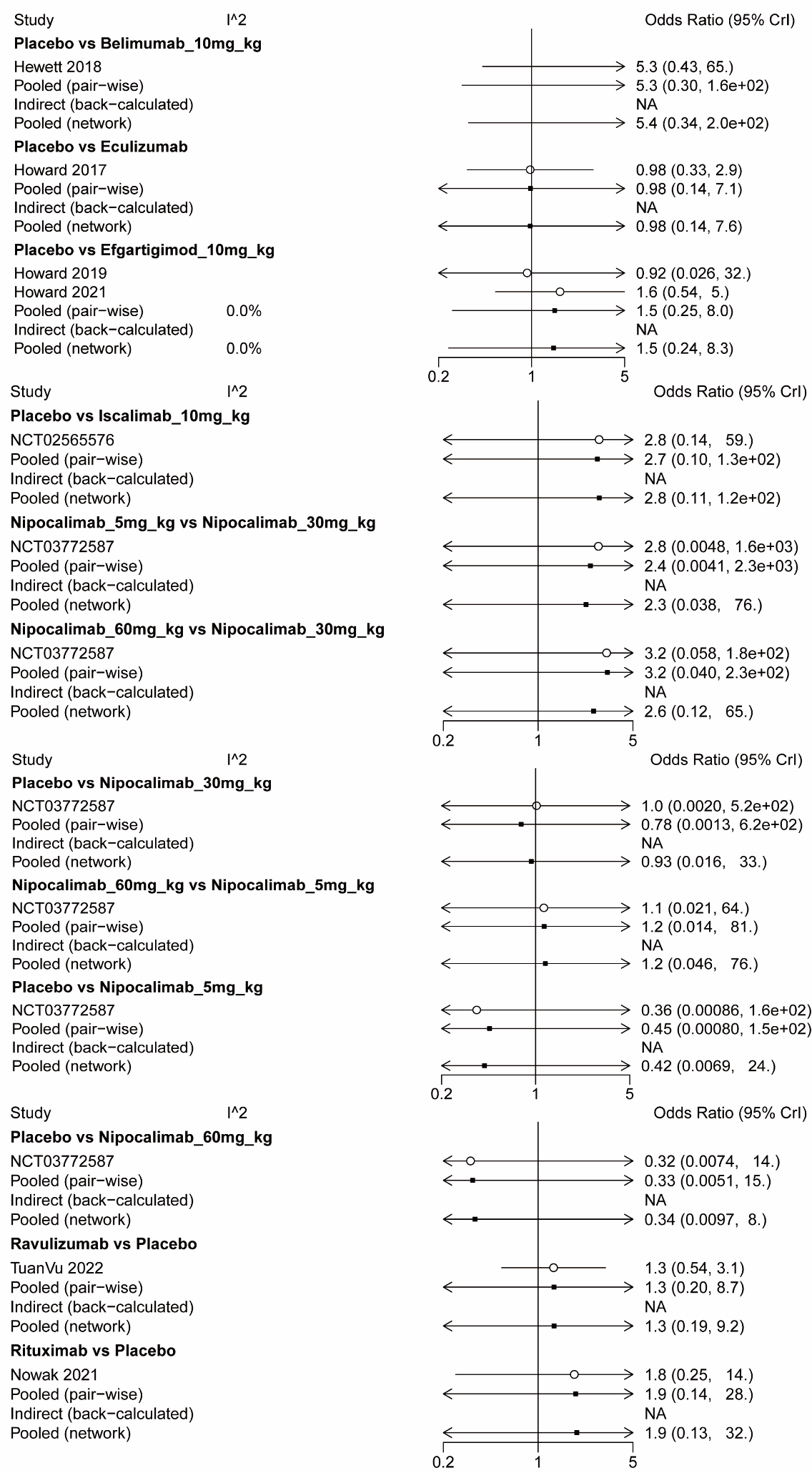
**

**
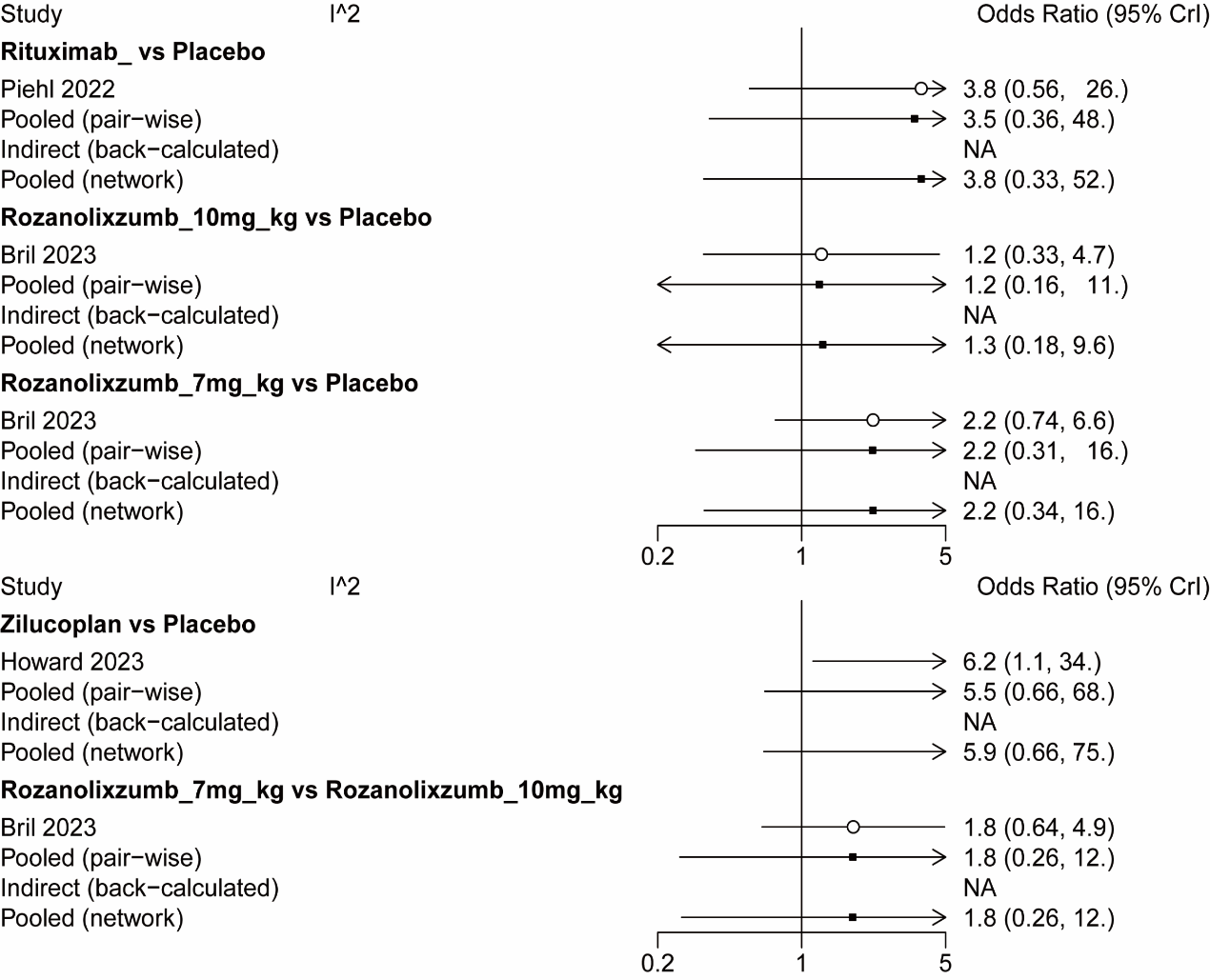
**

**Figure S45: Forest plots for the heterogeneity: Nausea.**

**
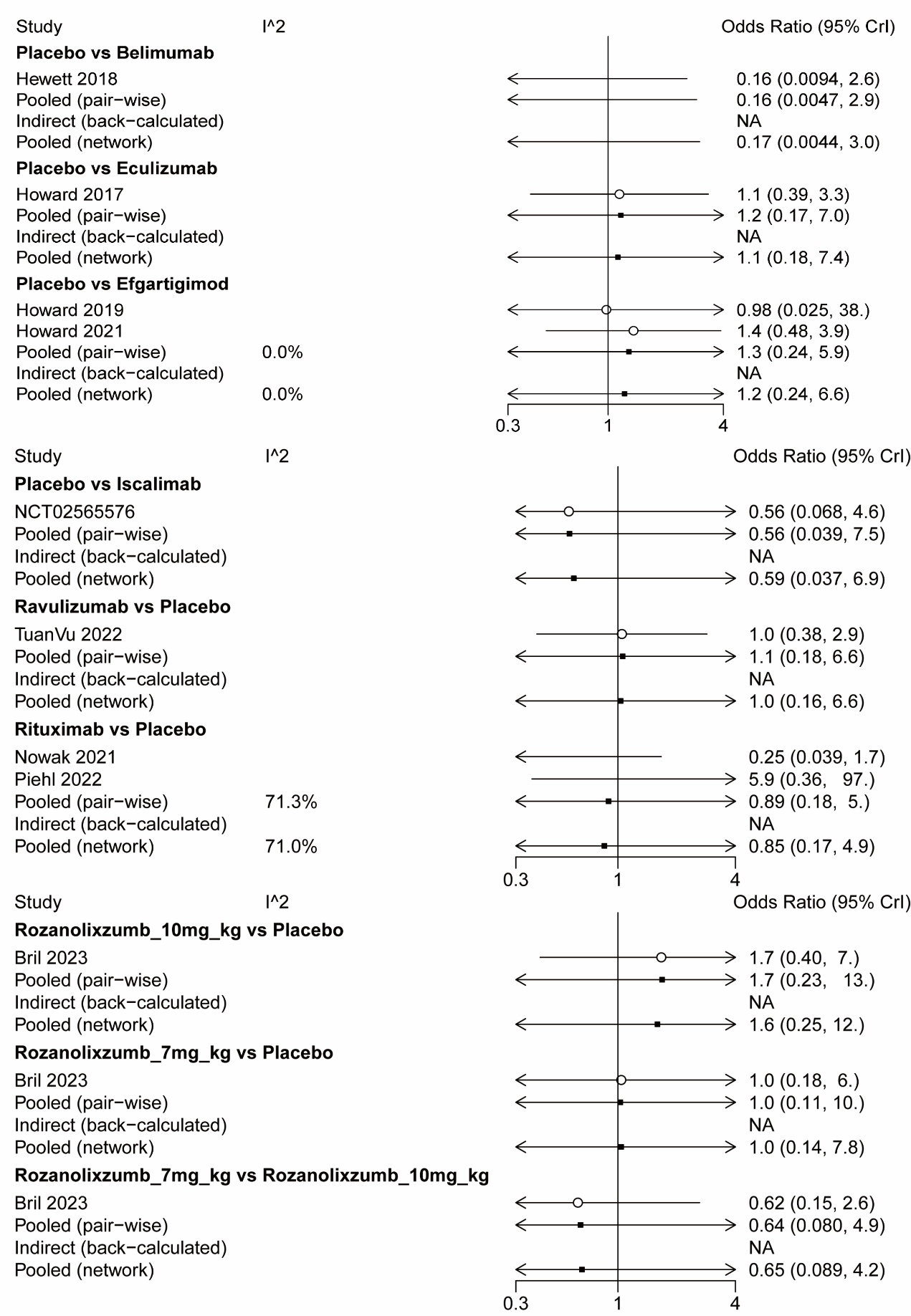
**

**Figure S46: Funnel plot for MG-ADL**

**
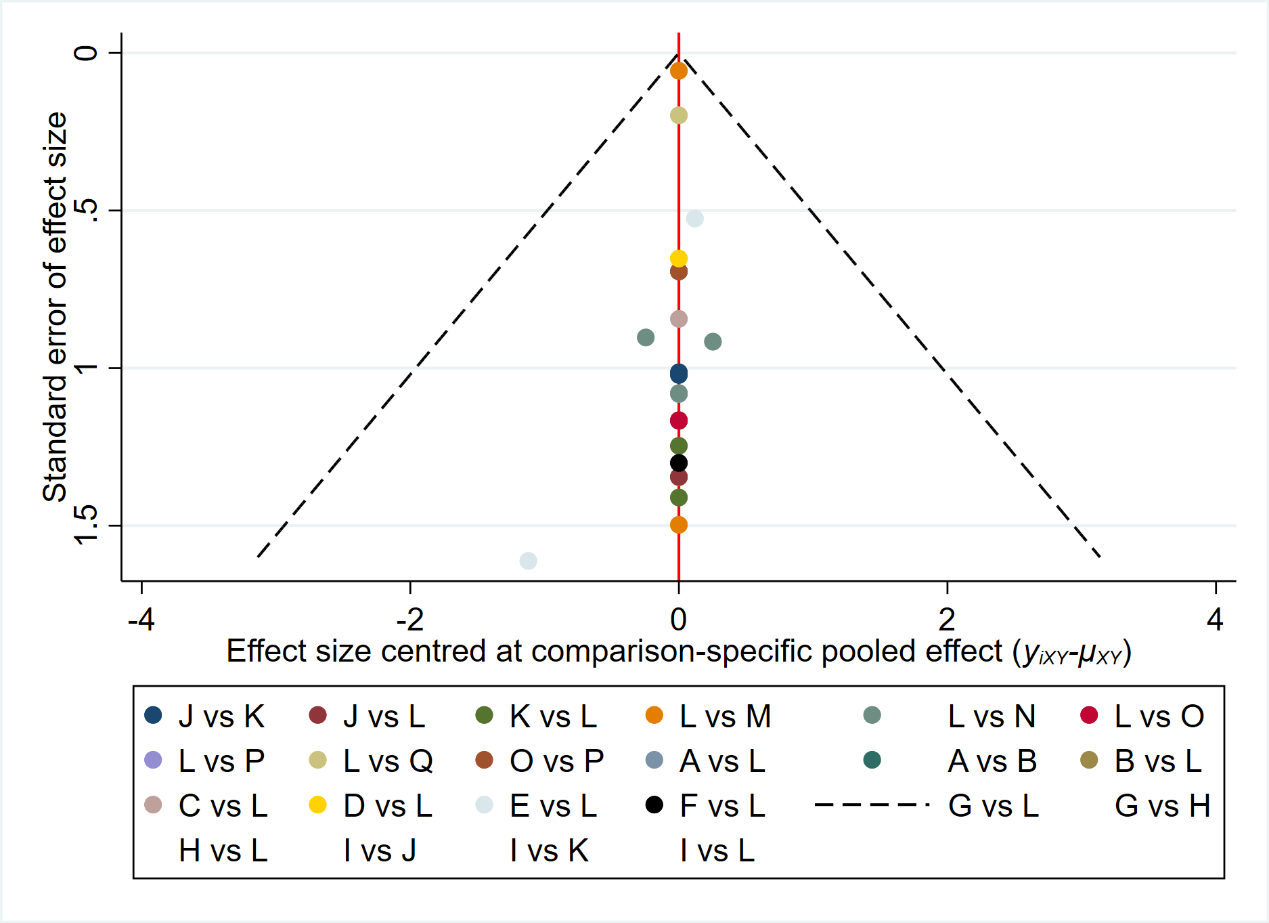
**

**Figure S47: Funnel plot for QMG**

**
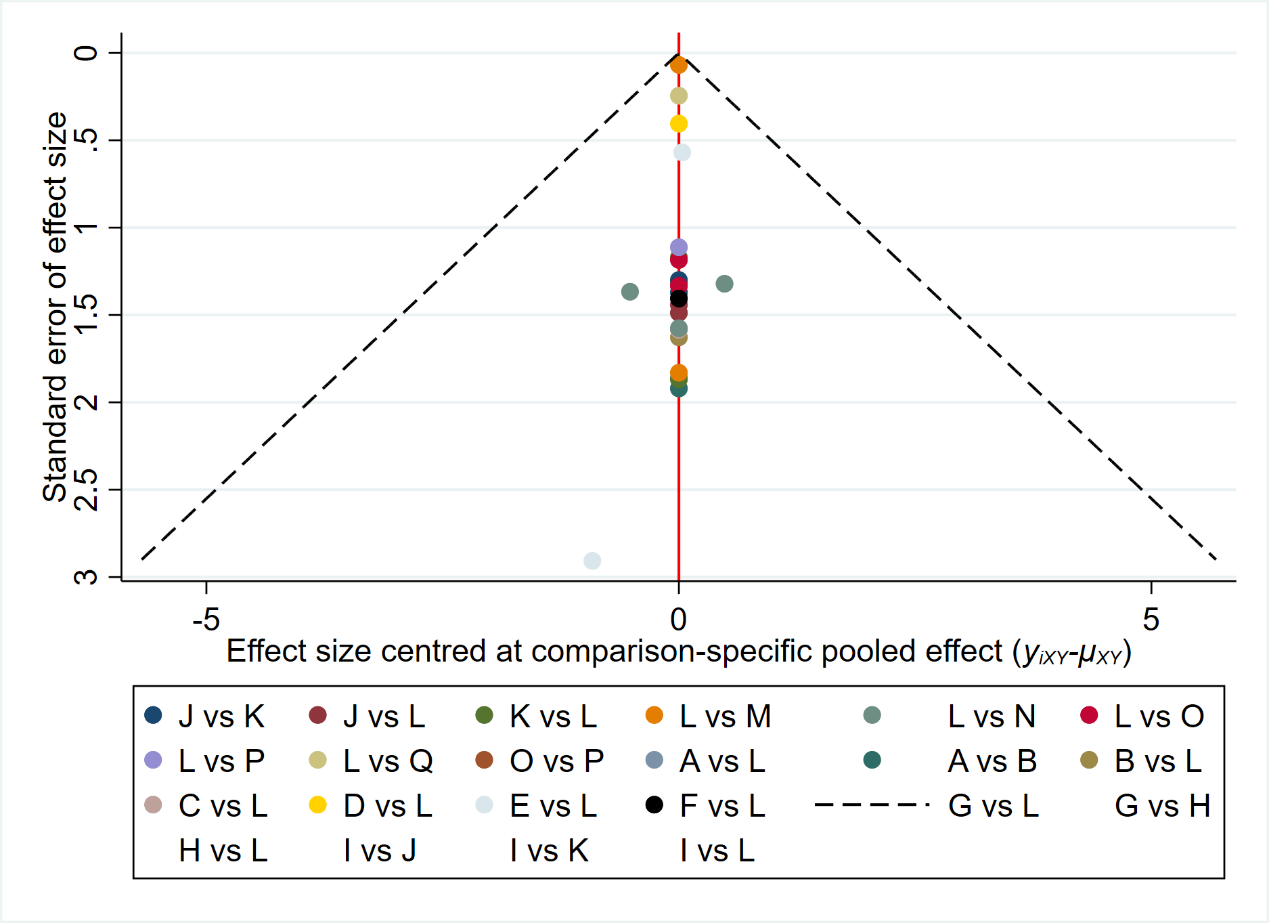
**

**Figure S48: Funnel plot for MGC**

**
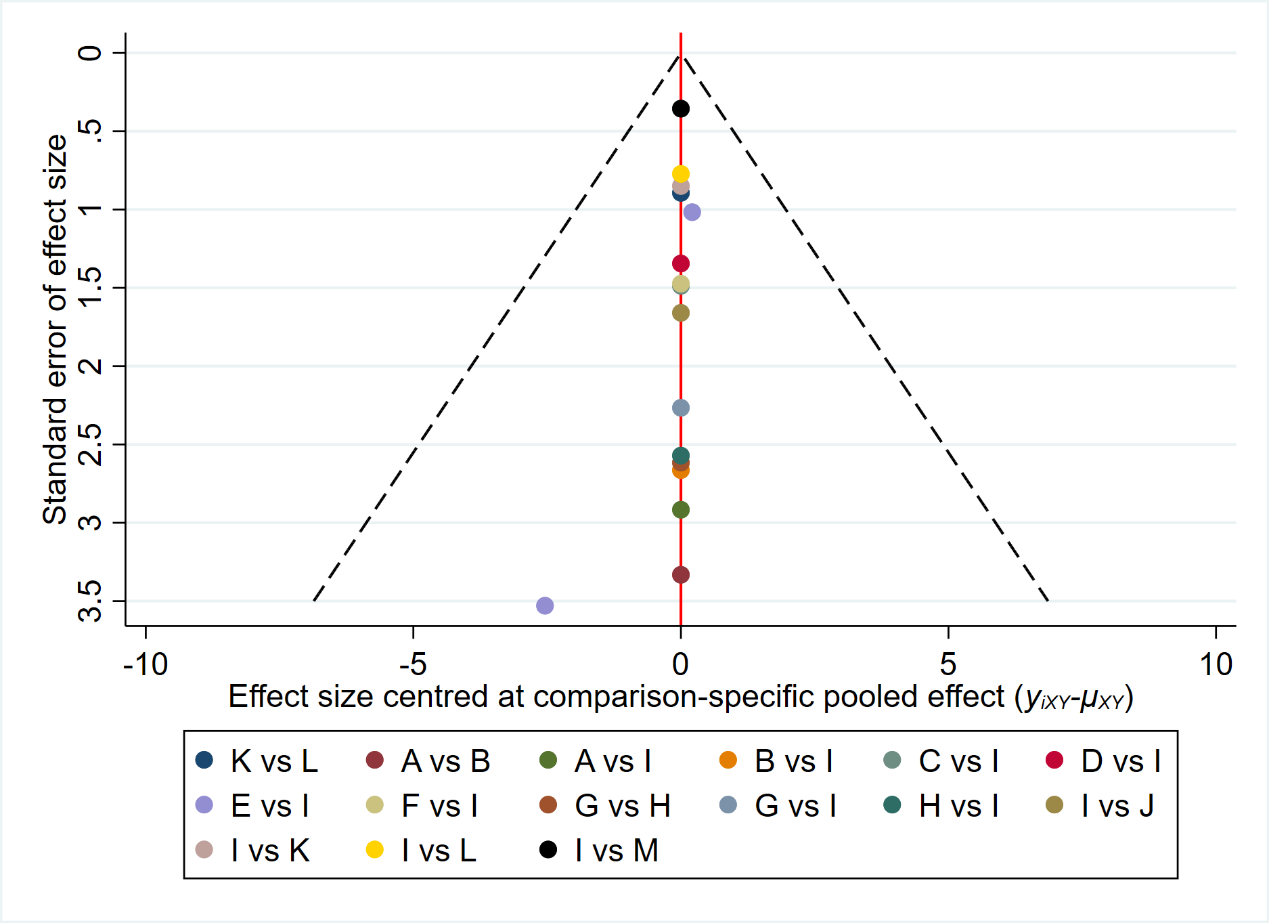
**

**Figure S49: Funnel plot for and MG-QoL 15r**

**
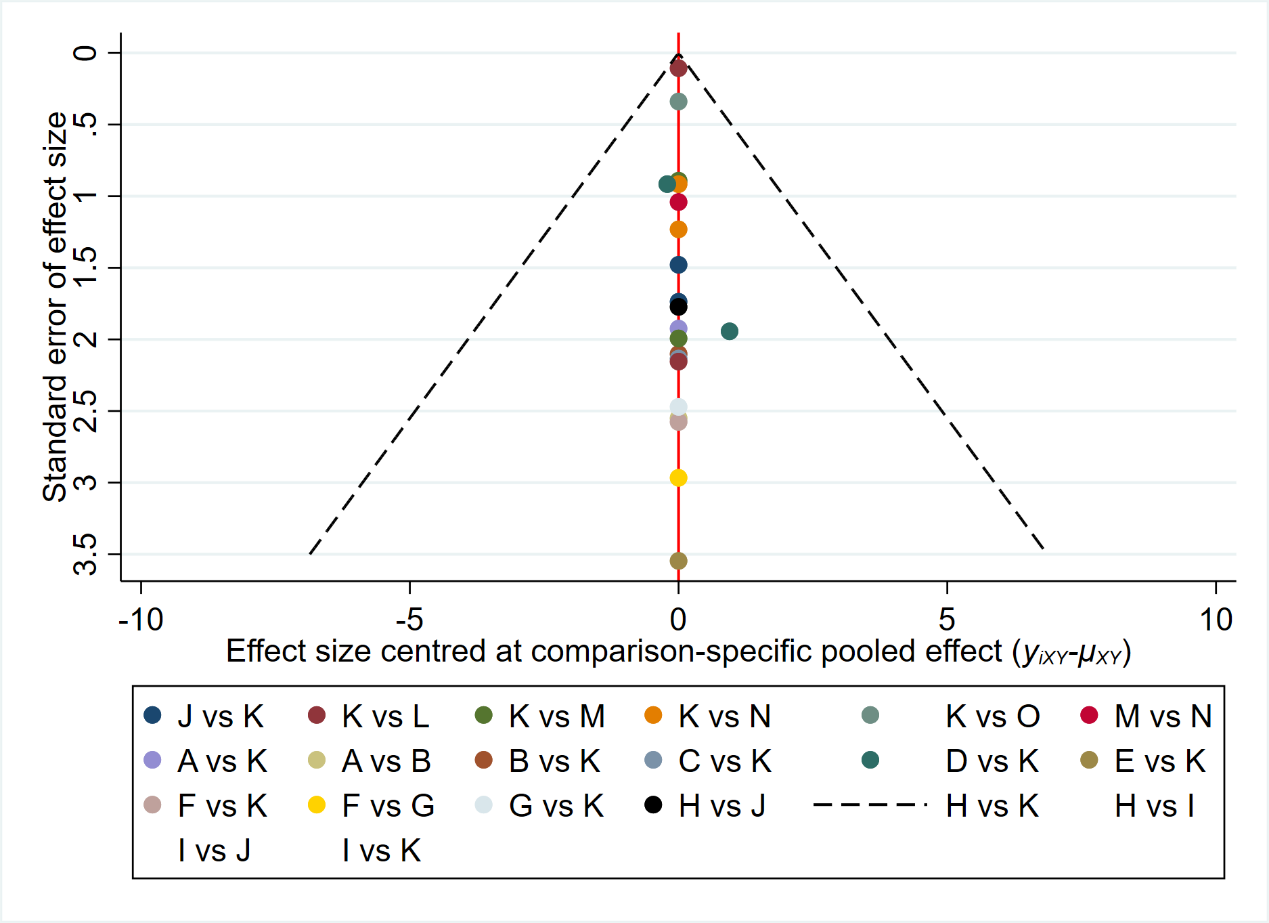
**

**Figure S50: Funnel plot for AEs**

**
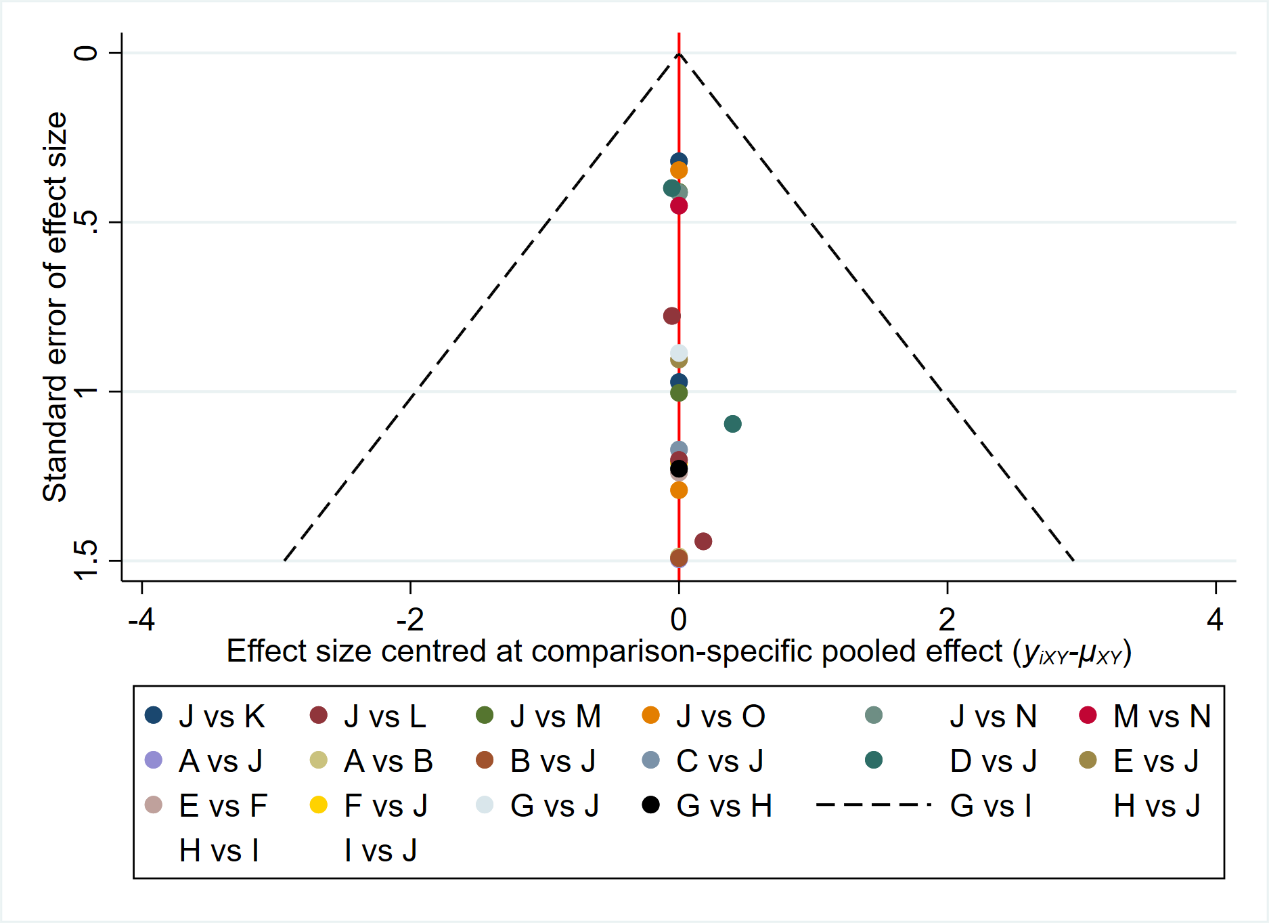
**

**Figure S51: Funnel plot for Headache**

**
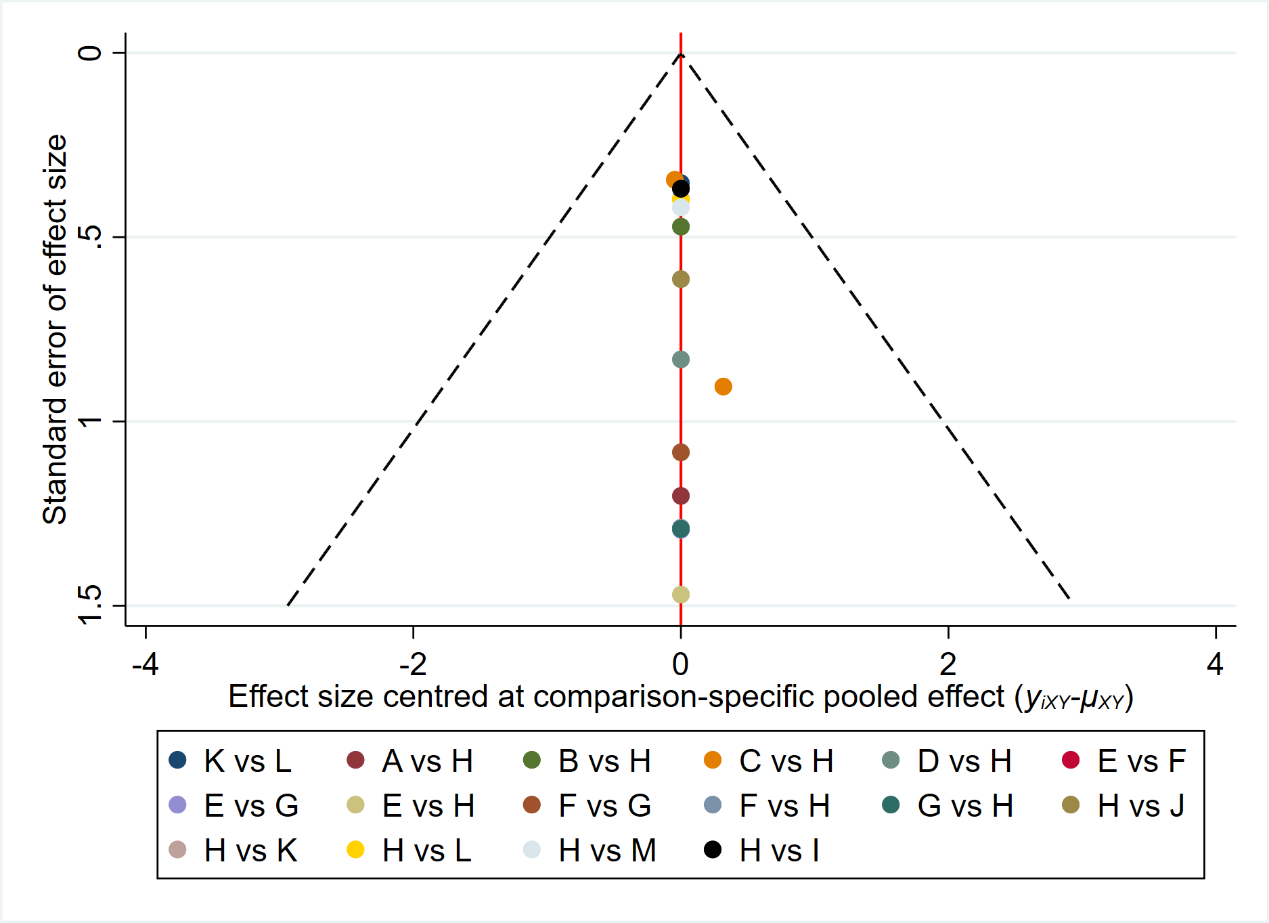
**

**Figure S52: Funnel plot for Diarrhea.**

**
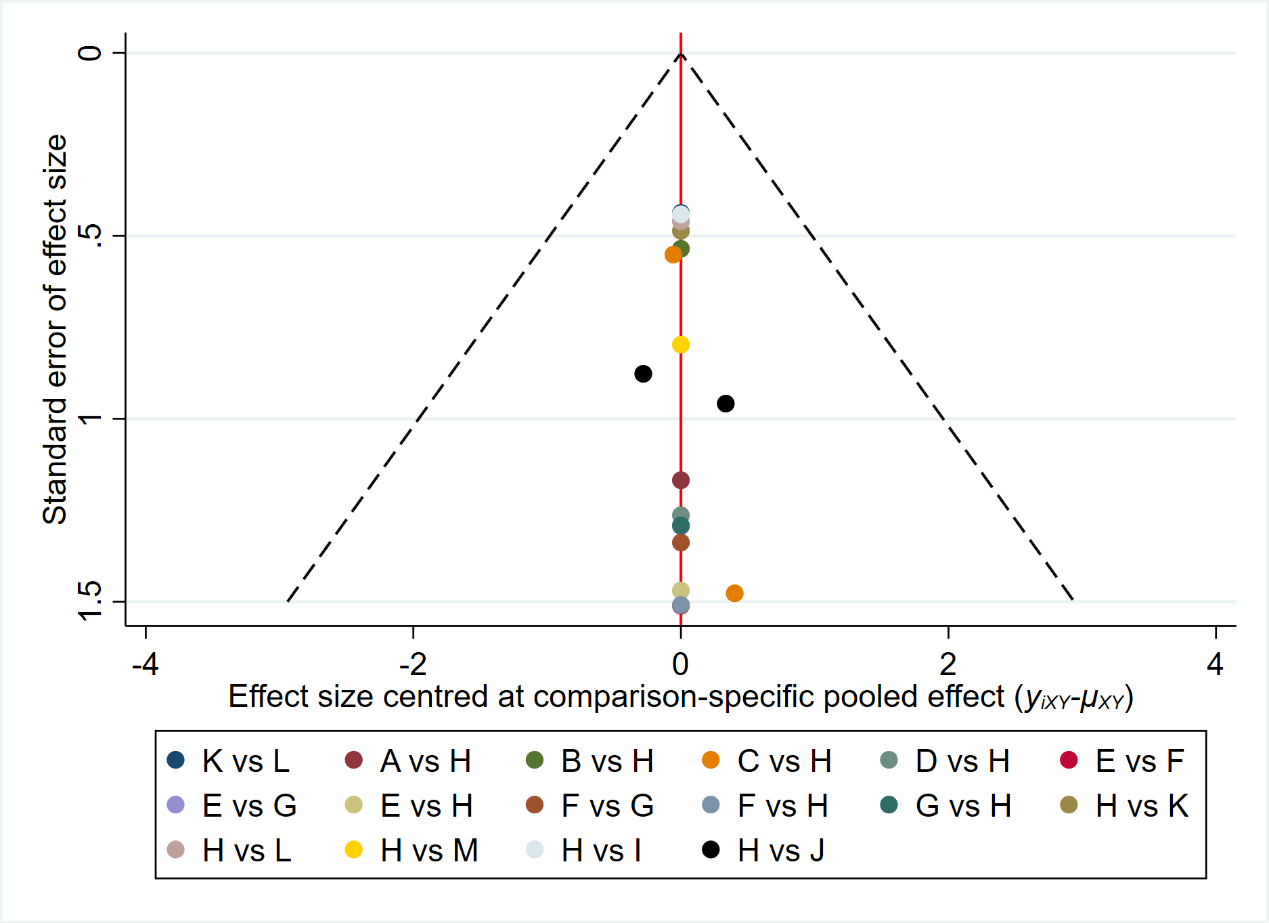
**

**Figure S53: Funnel plot for Nausea.**

**
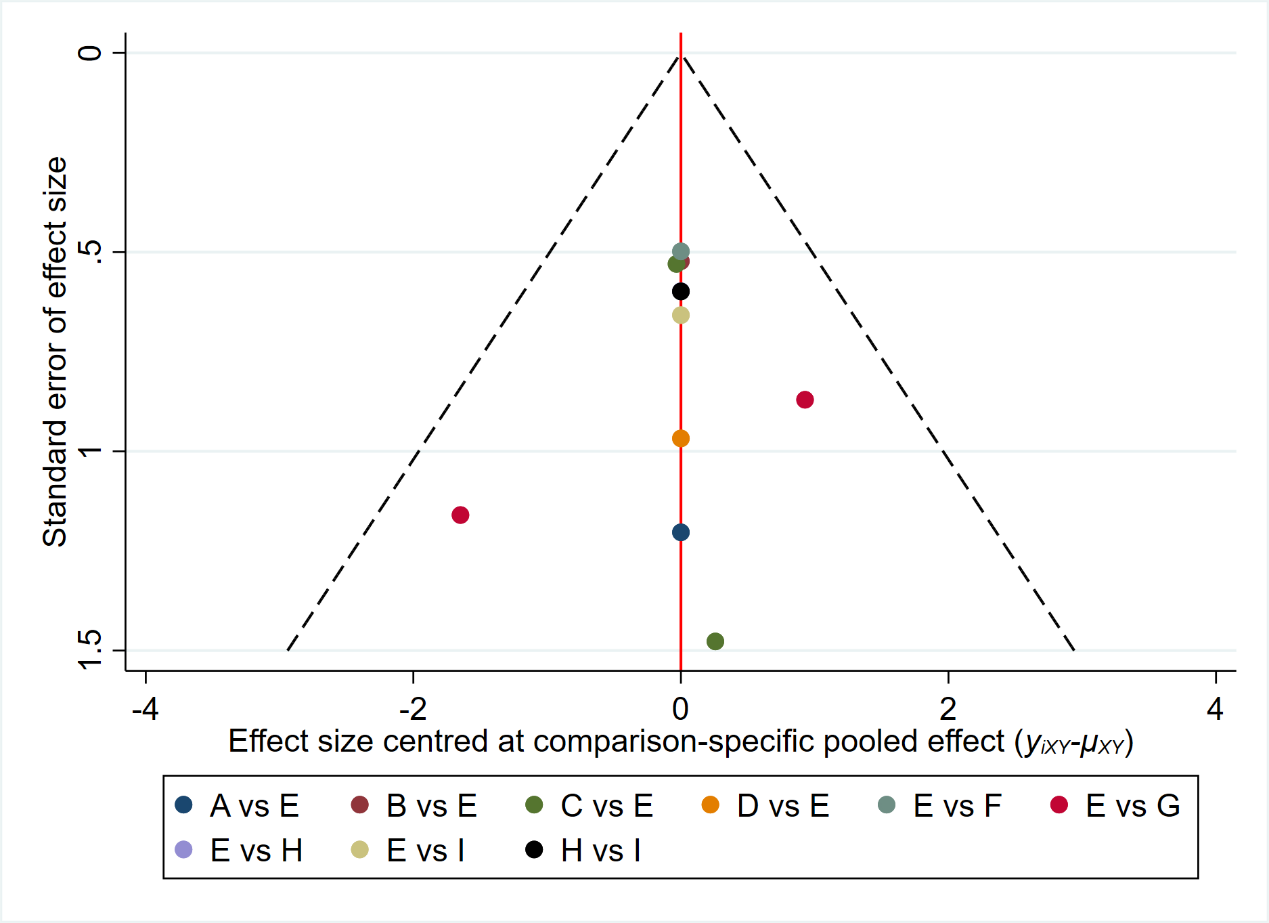
**

**Reference:**

1. Bril V, Drużdż A, Grosskreutz J, Habib AA, Mantegazza R, Sacconi S, et al. Safety and Efficacy of Rozanolixizumab in Patients with Generalised Myasthenia Gravis (Mycaring): A Randomised, Double-Blind, Placebo-Controlled, Adaptive Phase 3 Study. *Lancet Neurol* (2023) 22(5):383-94. Epub 2023/04/15. doi: 10.1016/s1474-4422(23)00077-7.

2. Yan C, Duan RS, Yang H, Li HF, Zou Z, Zhang H, et al. Therapeutic Effects of Batoclimab in Chinese Patients with Generalized Myasthenia Gravis: A Double-Blinded, Randomized, Placebo-Controlled Phase Ii Study. *Neurol Ther* (2022) 11(2):815-34. Epub 2022/04/13. doi: 10.1007/s40120-022-00345-9.

3. Howard JF, Jr., Bril V, Burns TM, Mantegazza R, Bilinska M, Szczudlik A, et al. Randomized Phase 2 Study of Fcrn Antagonist Efgartigimod in Generalized Myasthenia Gravis. *Neurology* (2019) 92(23):e2661-e73. Epub 2019/05/24. doi: 10.1212/wnl.0000000000007600.

4. Howard JF, Jr., Bril V, Vu T, Karam C, Peric S, Margania T, et al. Safety, Efficacy, and Tolerability of Efgartigimod in Patients with Generalised Myasthenia Gravis (Adapt): A Multicentre, Randomised, Placebo-Controlled, Phase 3 Trial. *Lancet Neurol* (2021) 20(7):526-36. Epub 2021/06/20. doi: 10.1016/s1474-4422(21)00159-9.

5. Momenta Pharmaceuticals I. A Study to Evaluate the Safety, Tolerability, Efficacy, Pharmacokinetics and Pharmacodynamics of M281 Administered to Adults with Generalized Myasthenia Gravis (2023). <https://www.clinicaltrials.gov/study/NCT03772587?term=NCT03772587&rank=1> (Accessed July 7, 2023).

6. Howard JF, Jr., Bresch S, Genge A, Hewamadduma C, Hinton J, Hussain Y, et al. Safety and Efficacy of Zilucoplan in Patients with Generalised Myasthenia Gravis (Raise): A Randomised, Double-Blind, Placebo-Controlled, Phase 3 Study. *Lancet Neurol* (2023) 22(5):395-406. Epub 2023/04/15. doi: 10.1016/s1474-4422(23)00080-7.

7. Howard JF, Jr., Utsugisawa K, Benatar M, Murai H, Barohn RJ, Illa I, et al. Safety and Efficacy of Eculizumab in Anti-Acetylcholine Receptor Antibody-Positive Refractory Generalised Myasthenia Gravis (Regain): A Phase 3, Randomised, Double-Blind, Placebo-Controlled, Multicentre Study. *Lancet Neurol* (2017) 16(12):976-86. Epub 2017/10/27. doi: 10.1016/s1474-4422(17)30369-1.

8. Alexion. Safety and Efficacy Study of Ravulizumab in Adults with Generalized Myasthenia Gravis (2022). <https://www.clinicaltrials.gov/study/NCT03920293?term=NCT03920293&rank=1> (Accessed July 7, 2023).

9. Piehl F, Eriksson-Dufva A, Budzianowska A, Feresiadou A, Hansson W, Hietala MA, et al. Efficacy and Safety of Rituximab for New-Onset Generalized Myasthenia Gravis: The Rinomax Randomized Clinical Trial. *JAMA Neurol* (2022) 79(11):1105-12. Epub 2022/09/20. doi: 10.1001/jamaneurol.2022.2887.

10. Nowak RJ, Coffey CS, Goldstein JM, Dimachkie MM, Benatar M, Kissel JT, et al. Phase 2 Trial of Rituximab in Acetylcholine Receptor Antibody-Positive Generalized Myasthenia Gravis: The Beatmg Study. *Neurology* (2022) 98(4):E376-E89. doi: 10.1212/WNL.0000000000013121.

11. Hewett K, Sanders DB, Grove RA, Broderick CL, Rudo TJ, Bassiri A, et al. Randomized Study of Adjunctive Belimumab in Participants with Generalized Myasthenia Gravis. *Neurology* (2018) 90(16):e1425-e34. Epub 2018/04/18. doi: 10.1212/wnl.0000000000005323.

12. Pharmaceuticals N. Safety,Tolerability,Pharmacokinetics and Efficacy of Cfz533 in Moderate to Severe Myasthenia Gravis (2021). <https://www.clinicaltrials.gov/study/NCT02565576?term=NCT02565576&rank=1> (Accessed July 7, 2023).

13. Takeda. A Study of Tak-079 in People with Generalized Myasthenia Gravis (2023). <https://www.clinicaltrials.gov/study/NCT04159805?term=NCT04159805&rank=1> (Accessed July 7, 2023).
